# Flow electrochemistry: a safe tool for fluorine chemistry†

**DOI:** 10.1039/d1sc02123k

**Published:** 2021-06-04

**Authors:** Bethan Winterson, Tim Rennigholtz, Thomas Wirth

**Affiliations:** School of Chemistry, Cardiff University Park Place, Main Building Cardiff CF10 3AT Cymru/Wales UK wirth@cf.ac.uk

## Abstract

The heightened activity of compounds containing fluorine, especially in the field of pharmaceuticals, provides major impetus for the development of new fluorination procedures. A scalable, versatile, and safe electrochemical fluorination protocol is conferred. The strategy proceeds through a transient (difluoroiodo)arene, generated by anodic oxidation of an iodoarene mediator. Even the isolation of iodine(iii) difluorides was facile since electrolysis was performed in the absence of other reagents. A broad range of hypervalent iodine mediated reactions were achieved in high yields by coupling the electrolysis step with downstream reactions in flow, surpassing limitations of batch chemistry. (Difluoroiodo)arenes are toxic and suffer from chemical instability, so the uninterrupted generation and immediate use in flow is highly advantageous. High flow rates facilitated productivities of up to 834 mg h^−1^ with vastly reduced reaction times. Integration into a fully automated machine and in-line quenching was key in reducing the hazards surrounding the use of hydrofluoric acid.

## Abstract 2

A scalable, efficient and safe electrochemical fluorination protocol is conferred. A broad range of iodine(iii) mediated transformations were performed in high yields without exposure to toxic HF.

## Graphical abstract

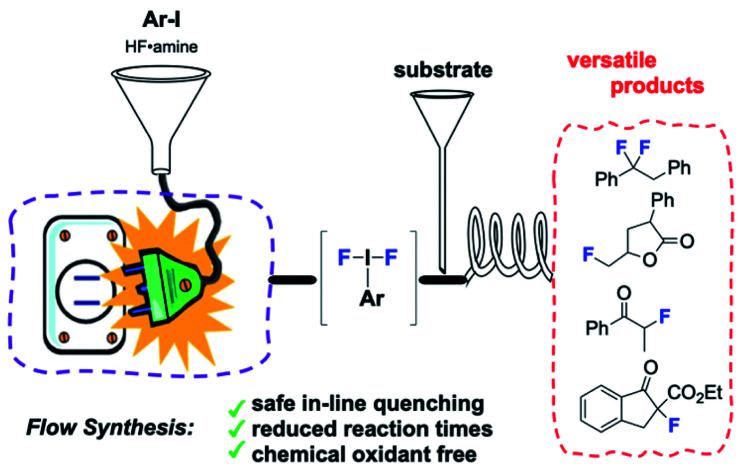

## Introduction

The construction of carbon–fluorine bonds in organic molecules is an upsurging field in chemical synthesis, which still presents ongoing difficulties for organic chemists. Fluorination can enhance the lipophilicity, membrane permeability, metabolism, and therapeutic properties of drug molecules.^1,2^ In recent years, fluoropharmaceuticals have seen exponential growth in the drug market, which provides major impetus for sustained development. Almost one third of the top performing drugs in the market contain a fluorine atom in their structure.^3^ For instance, lansoprazole (Prevacid), regulating stomach acid, and fluoxetine (Prozac), an anti-depressant, are considered among the most successful fluorine containing drugs. Broader incorporation is, nonetheless, largely limited by synthetic accessibility. Naturally occurring organic fluorine compounds are relatively sparse and there are only a few biologically synthesised organofluorides.^4^ Thus, easy routes to C–F bond formation cannot be derived from nature.

The development of aza-fluoro reagents such as Selectfluor®,^5^*N*-fluoropyridinium salts,^6^ and diethylaminosulfur trifluoride^7^ was a major breakthrough, as they are bench stable. Nevertheless, their high price, preparation from fluorine gas and significantly poor atom economy means there is limited potential for industrial application. Hydrofluoric acid (HF) is commercially available and extremely low cost as it is the fluorine source for many organofluorine compounds such as fluoropolymers and refrigerants. It is also used as cleaning reagent in the semiconductor industry.^8^ Though, the utilization of HF, even on a laboratory scale, has been hindered by its toxicity. Henceforth, despite the tremendous progress in organofluorine chemistry, safe and industrially viable fluorination protocols are still challenging.

**Fig. 1:**
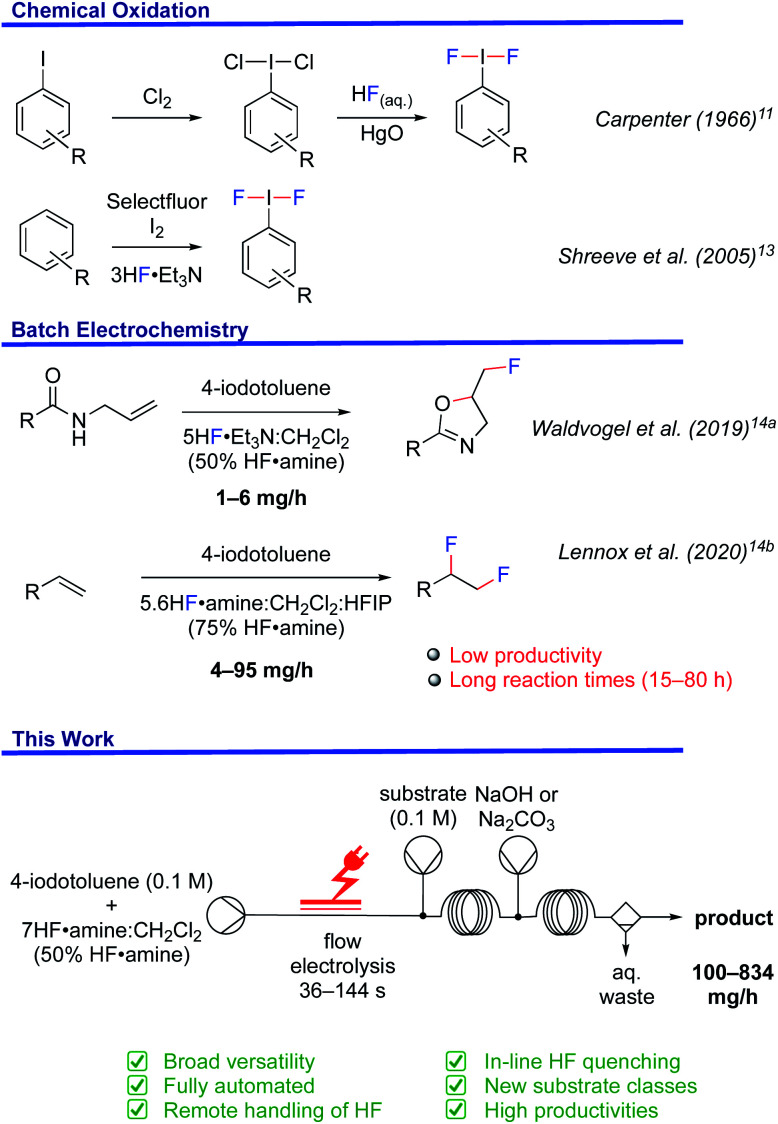
Methods for the preparation and use of (difluoroiodo)arenes.

Hypervalent iodine reagents have emerged as powerful tools for fluorination chemistry.^9^ (Difluoroiodo)arenes are typically prepared with HF and XeF_2_ for oxidations,^10^ ligand exchange of (dichloroiodo)arenes in the presence of HgO,^11^ or the reaction of iodosyl arenes with HF.^12^ Shreeve *et al.* disclosed a straightforward synthesis from arenes using Selectfluor®, Et_3_N·3HF and elemental iodine (Fig. 1).^13^ However, the requirement for stoichiometric amounts of chemical oxidants in these reactions is not sustainable.

In order to foster a sustainable approach, the batch electrochemical synthesis of (difluoroiodo)arenes has been subject to ongoing investigation (Fig. 1).^14^ For example, the electrochemical fluorocyclisation of *N*-allylcarboxamides, fluoro-desulfurisation of dithioacetals and fluorination of α-dicarbonyl compounds was the subject of elegant work by Waldvogel^14*a*^ and Fuchigami.^14*c*,*d*^ While the selectivity and yields of the products were generally high, the substrate types amenable were limited because success was typically contingent on in-cell applications. For instance, *N*-allylcarboxamides bearing nitro functionalities, liable to cathodic reduction, did not give the desired oxazoline in this manner.^14*a*^ Applying an ex-cell approach yielded the desired oxazoline, albeit in a low yield, attributed to the chemical instability of the I(iii)F_2_ mediator.^14*a*^ Even so, Lennox *et al.* demonstrated improved product diversity by successfully using ex-cell methodology, which avoids competitive substrate oxidation or un-selective fluorination.^14*b*^

While these examples represent a significant advancement in electrochemical fluorinations, the low productivity of batch procedures and hazards surrounding the handling of large volumes of HF have yet to be addressed. Flow chemistry can be particularly advantageous. With respect to safety, flow systems can be superior when in-line quenching is realised which minimises the HF exposure risk to the experimentalist. Flow chemistry can even be combined with automated platforms so that unsafe reagents can be completely remotely managed.^15,16^ Especially, when considering the larger volumes of HF necessary for gram-decagram synthesis, remote management of un-safe reagents becomes particularly beneficial. Industrial applications are inherently biased towards synthetic strategies that are safe and reduce constraints in resources. It is, therefore, manifest that a flow procedure would be of significant value. Herein, we disclose our efforts into the development of a safe, scalable, and versatile fluorination protocol for organic molecules.

## Results and discussion

The electrolysis was performed in an undivided, commercially available flow cell^17^ bearing two platinum electrodes with an active surface area of 12 cm^2^, applying galvanostatic conditions. A PFTE recessed channel was fitted in the flow cell giving a 0.6 mL internal volume and a 0.5 mm interelectrode gap. *N*-Allyl-4-methylbenzamide **1a** served as a test substrate for the elaboration of optimal electrolytic conditions for the synthesis of iodine(iii) difluoride reagents. The yield of the hypervalent iodine reagent was inferred by the yield of **2a** (using ^19^F NMR spectroscopy with ethyl fluoroacetate as internal standard, Table 1), given the purification of (difluoroiodo)toluene is more hazardous than **2a**. Subsequently, the electrolysis was investigated in detail through varying the electrode materials, solvent system, flow rate, and current density. Initially, applying reaction conditions similar to the literature^14*a*^ with 4-iodotoluene (0.1 M), an applied charge of 3 F, flow rate of 0.1 mL min^−1^, and stirring for 12 h post electrolysis in CH_2_Cl_2_/5HF·NEt_3_ (1 : 1 v/v), the desired product **2a** was observed in a moderate 51% yield (Table 1, entry 1). Exchanging the expensive 5HF·NEt_3_ (ref. 18) to 5.6HF·amine, made by diluting the cheaper and readily available 9HF·Py (ref. 19) with 3HF·NEt_3_,^20^ led to no reduction in yield (Table 1, entry 2). For the oxidation of iodine(i) to iodine(iii), a theoretical charge amount of 2 F is required. Though, higher current densities promoted product formation to higher yields but an increase from 5 F to 6 F led to no additional increase (Table 1, entries 2–4). Further studies were conducted by screening the solvent system.

**Table 1:** Optimization of the anodic oxidation of 4-iodotoluene under flow conditions^a^

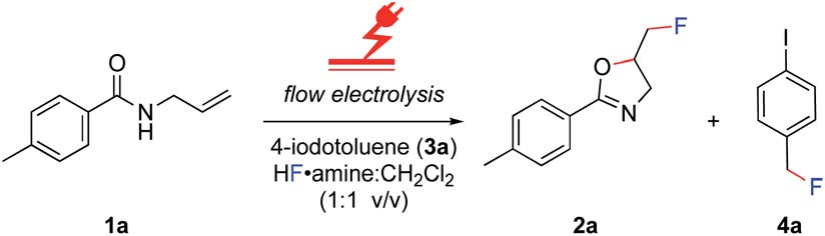						
Entry	Charge (F)	Flow rate (mL min^−1^)	*n*HF·amine	Co-solvent	Yield **2a** (%)^b^	Yield **4a** (%)^b^
1	3	0.1	5HF·NEt_3_	CH_2_Cl_2_	51	50
2	3	0.1	5.6HF·amine	CH_2_Cl_2_	50	12
3	5	0.1	5.6HF·amine	CH_2_Cl_2_	76	12
4	6	0.1	5.6HF·amine	CH_2_Cl_2_	75	12
5	5	0.1	3HF·NEt_3_	CH_2_Cl_2_	0	<5
6	5	0.1	4.5HF·amine	CH_2_Cl_2_	<5	87
7	5	0.1	5.6HF·amine	CH_2_Cl_2_	76	12
8	5	0.1	7HF·amine	CH_2_Cl_2_	>95	<5
9	5	0.1	7HF·amine	CH_2_Cl_2_ : HFIP (3 : 1)	82	0
10^c^	5	0.1	7HF·amine	CH_2_Cl_2_	0	0
11^d^	5	0.1	7HF·amine	CH_2_Cl_2_	0	0
12	5	0.25	7HF·amine	CH_2_Cl_2_	>95	<5
13	5	0.5	7HF·amine	CH_2_Cl_2_	87	<5
14	5	1	7HF·amine	CH_2_Cl_2_	75	<5

a Standard reaction conditions: undivided flow cell, Pt electrodes (active surface area: 12 cm^2^), interelectrode distance: 0.5 mm, **1a** (1 equiv.), 4-iodotoluene (1 equiv.), CH_2_Cl_2_: *n*HF·amine (1 : 1 v/v). HFIP: 1,1,1,3,3,3-hexafluoro-2-propanol.

b Yield determined by ^19^F NMR using ethyl fluoroacetate as internal standard.

c Glassy carbon anode.

d Panasonic carbon anode.

**Fig. 2:**
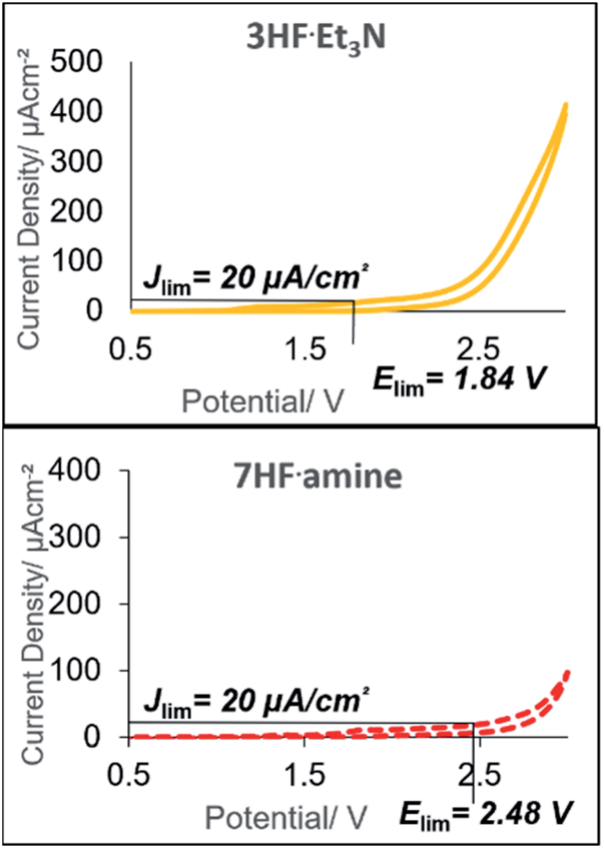
Cyclic voltammetry (CV) studies of HF·amine dilutions; 3HF·NEt_3_ (orange), 7HF·amine (red). CV conditions: *n*HF·amine : CH_2_Cl_2_ (1 : 15 v/v), Pt disk (immersed surface area: 3 mm^2^), Pt wire counter electrode, Ag/0.01 M AgCl reference, 10 mV s^−1^.

Despite reasonable yields of **2a**, the benzylic fluorination of **3a** was apparent. In contrast to the desired fluorocyclisation, benzylic fluorination is non-mediated and results from the direct anodic oxidation of 4-iodotoluene. Compound **4a** itself is a very poor mediator, thus the formation of this product had to be minimised.^14*b*^ Replacement of 5.6HF·amine by 3HF·NEt_3_ or 4.5HF·amine led to hampered or completely abolished fluorocyclisation (Table 1, entries 5 and 6). After electrolysis in 3HF·NEt_3_, the solution had a brown-violet colour consistent with iodine liberation (see ESI†). When 4.5HF·amine was used, benzylic fluorination was highly selective (Table 1, entry 6). Increasing the proportion of HF above 5.6HF·amine resulted in increased yields of **2a** and decreased yields of **4a** (Table 1, entries 7 and 8). After electrolysis, the solutions were pale yellow which is indicative of iodine(III) formation (see ESI†). The anodic oxidation of 4-iodotoluene proceeded with full conversion and a quantitative yield using 7HF·amine. To obtain an insight into the differences between the explored HF solutions, cyclic voltammetry studies were performed in various HF·amine dilutions (Fig. 2). For the electrochemical preparation of TolIF_2_, hydrofluoric acid provides the ligands, is the electrolyte, and contributes the balancing cathodic reaction. Due to the relatively high oxidation potentials of aryl iodides, the electrochemical stability of the solvent can be critical.^21^ The stability of the solvents against anodic oxidation was contrasted using the arbitrary stability criterion defined as *j*_lim_ = 20 μA cm^−2^. The anodic stability of the *n*HF·amine solvents increased with increasing values of *n*. For example, the 7HF·amine solvent is about 0.64 V (*E*_lim_ = 2.48 V, *j*_lim_ = 20 μA cm^−2^) more stable to oxidation compared to 3HF·NEt_3_ (*E*_lim_ = 1.84 V, *j*_lim_ = 20 μA cm^−2^). Although, these solvents should all be sufficiently stable to facilitate the desired iodine(iii) formation. Indeed, these solvents have previously been utilised in the electrochemical generation of (difluoroiodo)arenes.^14^ On the other hand, the cathodic potentials gave more insight. A higher cathodic potential was observed with decreasing values of *n*, *i.e.* 5.6HF·amine *E*_p_ = −1.21 V, and 7HF·amine *E*_p_ = −0.95 V. Hence, the increased yields in the higher acidity solutions can be, in part, attributed to their more facile reduction potential.

Subsequently, the influence of co-solvent was examined. We reasoned that the addition of HFIP could be beneficial for the transformation as it could act as a radical-stabilizing co-solvent, provide a more facile cathodic reaction, and even improve selectivity towards iodine(iii) formation. Yet, despite the complete suppression of benzylic fluorination, a significant reduction in the yield of **2a** was observed (Table 1, entry 9). Also, reduction in the HF dilution was investigated to minimize by-product formation and increase the percentage recovery of the mediator. However, any dilution resulted in decreased yields. Nonetheless, the HF acid requirement (50% v/v) is significantly lower than previous publications (75% v/v).^14*b*^ Varying the anodic material gave no improvement to the electrochemical procedure. Exchanging a platinum anode to a carbon based anode could be beneficial due to their reduced cost and lower overpotential (Table 1, entries 10 and 11). Albeit, both glassy carbon and panasonic carbon did not facilitate the desired reaction.

**Fig. 3:**
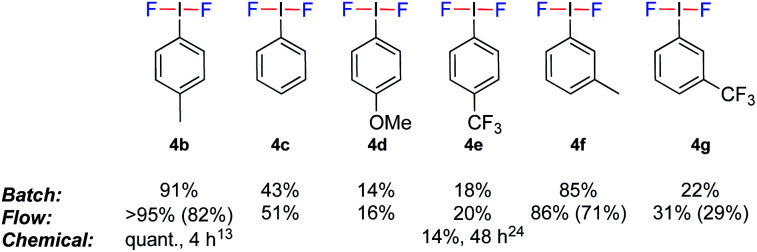
Electrochemically generated (difluoroiodo)arenes. Yields determined by ^19^F NMR spectroscopy using ethyl fluoroacetate as an internal standard. The NMR yields are based on the yield of the oxazoline **2a** (given the 1 : 1 stoichiometry). Yields in parenthesis are isolated yields of the corresponding (difluoroiodo)arenes.

To evaluate the potential of coupling the electrochemical generation of (difluoroiodo)toluene with a second reaction in flow, the stability of the electrolysis step over time was investigated. Using the optimal conditions (Table 1, entry 12), the electrolysis was run for 3.5 h and analysed every 30 min by ^19^F NMR spectroscopy. Pleasingly, no change in yield or voltage, which would be indicative of passivation, was observed. Identification of the mediator was confirmed *via* anodic oxidation of only 4-iodotoluene **3a** in flow. (Difluoroiodo)toluene **4b** was isolated as a pure product in 82% yield. A range of other iodoarenes could also be oxidised in acceptable to excellent yields. Whilst the electron rich arenes **4b** and **4f** could be synthesised in excellent yields, the electron poor arenes **4e** and **4g** were formed in lower yields due to their higher oxidation potential. For **4d**, the un-expected low yield is attributed to decomposition of the starting material during electrolysis. Even though a 4-*t*BuPhI mediator could effectively prevent benzylic fluorination, its high price (5 g = £168)^22^ as opposed to **3a** (25 g = £19.5)^23^ would not be economical. The electrochemical generation of iodine(iii) difluorides is still respectable given the higher yields and/or drastically reduced reaction times compared to chemical oxidants (Fig. 3).^13,24^

To demonstrate the efficiency of the flow electrochemical generation of **4b**, several hypervalent iodine mediated transformations were demonstrated in a coupled manner. Given the relatively high oxidation potentials of iodoarenes, efficient ex-cell protocols are important to prevent possible oxidation/reduction of the substrate. Since the instability of **4b** can make ex-cell procedures inefficient in batch,^14*a*^ we summarized the immediate generation and use in flow (2.4 min) compared to batch (7.2 h)^14*b*^ could be hugely beneficial as it allows little time for mediator degradation. More importantly, the long reaction times in batch could be circumvented by a flow approach, which drastically improves the productivity and industrial viability of these processes. For safety and exposure purposes, a simple in-line quenching process was established by injecting aqueous Na_2_CO_3_ or NaOH using a third pump after the desired reaction time and mixing in a third flow reactor, followed by phase separation (see ESI†).

**Fig. 4:**
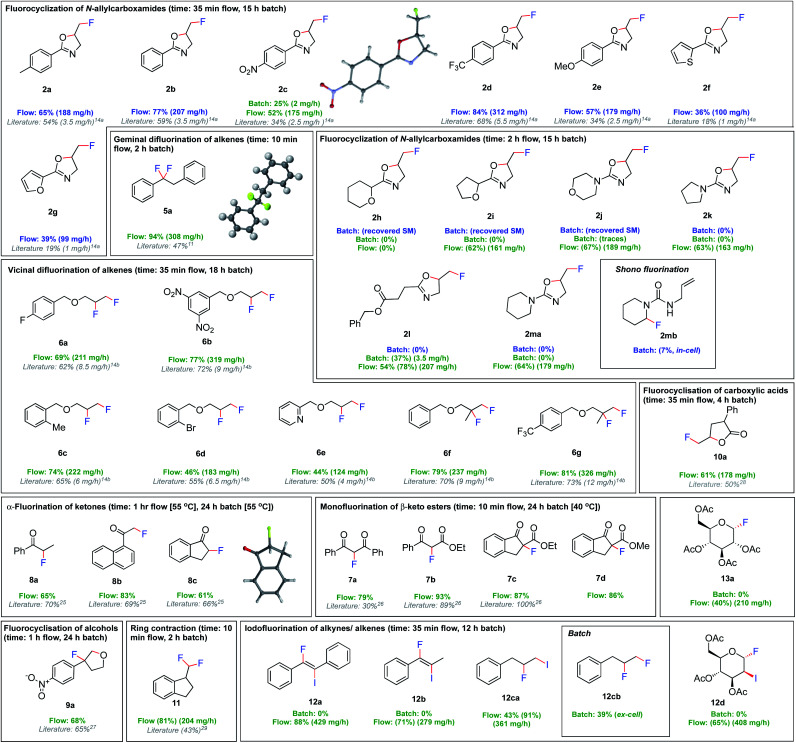
Fluorinations using flow electrolysis. Yields refer to the isolated products, or NMR yields in parentheses. Batch electrolysis is performed in an undivided cell, Pt (anode, active surface area 1.2 cm^2^) and Pt (cathode), 3 F, 50 mA cm^−2^, 5.6HF·amine : CH_2_Cl_2_ (1 : 1 v/v). Flow electrolysis is performed in an undivided cell, Pt (anode, active surface area 12 cm^2^) and Pt (cathode), 5 F, 16.75 cm^2^, 7HF·amine : CH_2_Cl_2_ (1 : 1 v/v), 0.25 mL min^−1^. Blue refers to in-cell procedures, green refers to ex-cell procedures.^30^

We then investigated the versatility of this fluorination procedure (Fig. 4). First, the fluorocyclisation of *N*-allylcarboxamides was targeted. Despite only moderately increased yields compared to batch, a dramatic increase in productivity was observed due to the shortened reaction times, from 1–6 mg h^−1^ in batch, to 100–311 mg h^−1^ in flow. Unsurprisingly, the ex-cell synthesis of **2c** in batch (25%) was much lower yielding than the flow procedure (52%). This is attributed to the chemical instability of (difluoroiodo)toluene, indicating the immediate use in flow is much more effective. Of particular note, electrochemical methods have so far been uniquely limited to aromatic substituents in the α-position to the carbonyl.^14*a*^ Indeed, the fluorocyclisation of **1h**, **1i**, **1j**, **1k** and **1m** did not proceed in batch. The substrates bearing saturated *N*-heterocycles **2j**, **2k** and **2m** were particularly problematic in batch. Their preference for Shono-type fluorination (*E*_p_ = 1.6 V for **1m**) as oppose to iodoarene oxidation (*E*_p_ = 1.7 V), meant in-cell applications were not possible. In fact, applying an in-cell procedure for compound **1m** gave no desired fluorocyclisation, but small quantities of Shono-type fluorination **2mb** (7%) (detected by HRMS and ^19^F NMR spectroscopy). No remaining starting material could be observed due to decomposition. Given their preference for Shono-type fluorination when exposed to electricity, we rationalized that an ex-cell approach was more viable. But, even the ex-cell approach in batch did not lead to product formation, although no decomposition of the starting material was observed. Since extending the reaction time from 15 h to 24 h (electrolysis time + stirring time) gave trace amounts of the products, we concluded that a flow approach may be more successful. With this approach, the non-aromatic oxazolines could be synthesised in good yields in flow, apart from **2h**. It was even possible to generate an aliphatic 2-oxazoline (**2l**) in a modest 54% isolated yield. Given the efficiency of the ex-cell application in flow, the scope of readily oxidised or reduced substrates was extended. A versatile set of reactions including *vic*/*gem* difluorinations, monofluorinations, α-fluorinations of ketones, ring contractions, and fluorocyclizations of alcohols and carboxylic acids could all be performed in modest to excellent yields. In all cases, the productivities were increased from 4–9 mg h^−1^ quantities in batch, to 100–300 mg h^−1^ in flow.

**Fig. 5:**
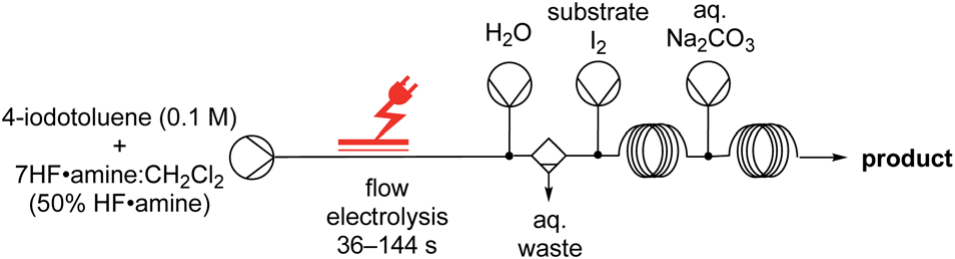
Reaction scheme for iodofluorinations.

Thereafter, we focused our attention on the di-functionalization of unsaturated compounds by two different atoms, such as the iodofluorination of alkenes/alkynes. Intriguingly, our initial baseline reactions in batch were unsuccessful. Adding molecular iodine followed by allylbenzene in an ex-cell manner did not yield **12ca**, and only the difluorinated product **12cb** was obtained in 39% yield. Since previous methods are performed in the absence of a HF·amine solvent,^31^ we thought that removal of excess amounts of nucleophilic fluoride maybe necessary to yield the desired product. Although aqueous-organic extraction to remove the HF·amine solvent was not reasonable in batch, the addition of an in-line liquid–liquid extractor^32^ in flow was simple. Injecting water and applying an in-flow liquid–liquid extractor after the electrochemical reactor facilitated removal of hydrofluoric acid (Fig. 5). Combining the organic solvent stream with a stream of molecular iodine and the alkene/alkyne yielded the iodofluorinated products **12a**, **12b** and **12ca** in excellent yields. Since the difluorinated product was not observed in the absence of HF, we reasoned that the issues experienced in batch are unlikely due to a competitive reaction between the TolIF_2_ and the alkene,^31^ but likely due to substitution of iodide, in the product, by nucleophilic fluoride. This, in turn, is promoted by excess amounts of the nucleophilic fluoride source, HF·amine.^33^ Indeed, treating the iodofluorinated product **12ca** with TolIF_2_ and 5.6HF·amine yielded the difluorinated product **12cb**. The pharmaceutically relevant compounds **12d** and **13a** were also synthesised in good yields, **12d** through the iodofluorination of the alkene and **13a** by the electrochemical fluorination of the corresponding thioacetal. Overall, the yields of the final products are generally high, and the system could be easily manipulated to accompany the different reaction conditions required for each product.

**Fig. 6:**
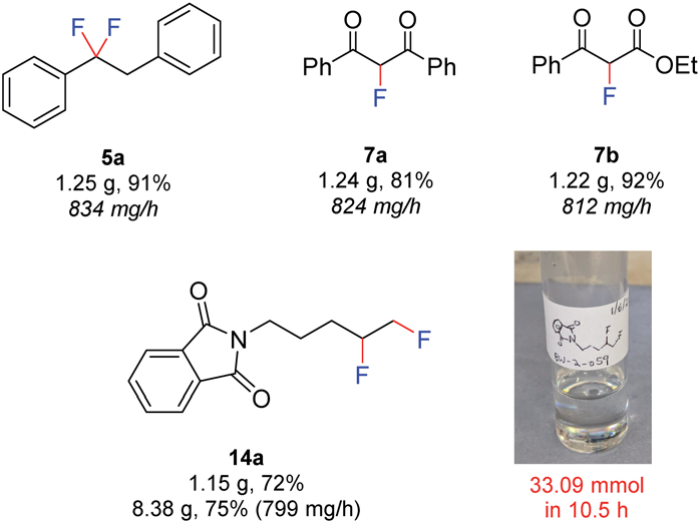
Fluorinations using automated electrolysis.

Finally, in an effort to further increase the safety and scalability prospects, a fully automated approach was developed (Fig. 6). The pumps and potentiostat were computer controlled and the product was collected automatically using a fraction collector. Using this approach, the rapid exercise of multiple experiments and completely remote handling of hydrofluoric acid mixtures was possible. The larger volumes of HF·amine necessary for scale-up procedures could be handled in a safer manner, since no human interaction was needed. In this procedure, a flow rate of 1.0 mL min^−1^ (Table 1, entry 14), a substrate concentration of 0.07 M, and a 4-iodotoluene concentration of 0.1 M was used. The scalability of the method was initially demonstrated by preparing gram-scale quantities of **5a** (1.25 g, 834 mg h^−1^), **7a** (1.24 g, 824 mg h^−1^) and **7b** (1.22 g, 812 mg h^−1^). By this method, the productivity of **7a** was increased from 287 mg h^−1^ at a flow rate of 0.25 mL min^−1^, to 824 mg h^−1^ at 1.0 mL min^−1^ and each 6.3 mmol reaction could be performed in just 1.5 h. Also, the difluorinated product **14a**, derived from the unactivated alkene, could even be produced on an 8 g scale in just 10.5 h. Pleasingly, upon scaling up from a 1 g to 8 g scale for compound **14a**, no decrease in yield was observed. Inspection of the electrodes following all large scale reactions revealed no passivation and a steady voltage was observed throughout the experiments. Since all scale up reactions could be performed with no detrimental effect on the yield of the products, the above results demonstrate the reliability of the flow electrochemical fluorination procedure.

## Conclusions

In summary, a scalable, reliable, and safe fluorination procedure using hypervalent iodine mediators has been developed. (Difluoroiodo)arenes are toxic, suffer from chemical instability and are prone to hydrolysis, so the uninterrupted generation and immediate use in flow exceeds limitations in batch. Since air and moisture can be effectively excluded, the yields of ex-cell applications in flow are often significantly higher yielding. Effectively decoupling iodine(iii) formation with the desired reaction removed any functional group bias of batch procedures. A versatile scope of hypervalent iodine mediated fluorinations such as fluorocyclisations, difluorinations, monofluorinations and ring contractions were achieved in good to excellent yields. The system could be effortlessly manipulated to facilitate a broad range of reactions by changing the residence time, solvent, or temperature of the reaction coil. Most notably, the electrochemical approach for the di-functionalisation of alkenes/alkynes by non-identical atoms could only be envisioned in flow. Integration into an automated electrolysis machine and a simple in-line quenching process facilitated completely remote handling of toxic hydrofluoric acid. Given the emerging importance of organic fluorination procedures for the pharmaceutical industry, and their bias towards procedures that are safe, scalable, and reduce constraints in resources, a flow procedure is invaluable.

## Data availability

All available data are included in the supporting information.

## Author contributions

Experiments: BW and TR, manuscript: BW and TW.

## Conflicts of interest

There are no conflicts to declare.

## Supplementary Material

SC-012-D1SC02123K-s001

SC-012-D1SC02123K-s002
