# Supplementary material for: Flow electrochemistry: a safe tool for fluorine chemistry
Source: Chem Sci. 2021 Jun 4;12(26):9053–9. doi: 10.1039/d1sc02123k (PMC8261735; doi:10.1039/d1sc02123k)

Electronic Supplementary Material (ESI) for Chemical Science.
This journal is © The Royal Society of Chemistry 2021

Table of Contents:

General Information S2
General Procedures for the Synthesis of the Starting Materials S4
General Procedure A for the Synthesis of Allyl Amides: S4
General Procedure B for the Synthesis of Allyl Alcohols: S4
General Procedure C for the Synthesis of Esters: S4
Characterisation of the Substrates: S5
General Electrolysis Procedures S13
General Electrolysis Protocol D for Screening and Optimization Studies: S13
General Electrolysis Procedures for the Synthesis of (Difluoroiodo)arenes: S13
General Batch Electrolysis Procedure E: S13
General Flow Electrolysis Procedure F: S13
General Electrolysis Procedures for Fluorination: S14
General Batch Electrolysis Procedure (in-cell) G: S14
General Batch Electrolysis Procedure (ex-cell) H: S14
General Flow Electrolysis Procedure (in-cell) I: S14
General Flow Electrolysis Procedure (ex-cell) J: S15
General Automated Flow Electrolysis Procedure K: S15
General Flow Electrolysis Procedure L: S15
Electrochemical Batch Reaction Equipment: S16
Electrochemical Flow Reactor: S17
Automated Electrochemical Apparatus: S18
Zaiput Liquid-Liquid Extractor: S19
Electrolysis Optimisation Studies: S20
Optimisation Reaction Scheme: S20
^{19}F NMR of the fluorinated oxazoline product and ethyl fluoroacetate S20
^{19}F NMR of the 1-(fluoromethyl)-4-iodobenzene and ethyl fluoroacetate S20
Table S1: Optimisation of Applied Charge S21
Table S2: Optimisation of HF.amine ratio S21
Table S3: Optimisation of Solvent Composition S22
Table S4: Optimisation of Anodic Material S22
Table S5: Optimisation of Flow Rate S22
Table S6: Measuring the Yield with Time S23
nHF•amine Anodic Potentials S24
nHF•amine Cathodic Potentials S24
Substrate Potentials S24
Characterization of the Products: S25
References: S43
NMR Spectra for the Fluorinated Products: S68

General Information

General:

Reactions involving air and moisture sensitive reagents were carried out in flame dried glass-ware under a dry argon atmosphere using a Schlenk technique. Room temperature (rt) refers to 20 – 25 °C. Temperatures of 0 °C were obtained using ice/water baths. Thin-layer chromatography (TLC) was performed on pre-coated aluminium sheets of Merck silica gel 60 F254 (0.20 mm) and visualized by UV radiation (254 nm). Automated column chromatography was performed on a Biotage® Isolera Four using Biotage® cartridges SNAP Ultra 10 g, 25 g, or 50 g. The progress of all reactions was monitored by thin-layer chromatography (TLC) using aluminium plates coated with silica (Kieselgel 60 F254 silica).

Reagents:

All chemicals were purchased from Acros Organics, Alfa Aesar GmbH & Co. KG, Fisher Scientific, FluoroChem, Merck KGAA and Sigma Aldrich GmbH. All reagents obtained from commercial sources were used as received. Dry solvents such THF, diethyl ether and toluene were obtained after passing these previously degassed solvents through activated alumina columns (Mbraun, SPS-800). Dry dichloromethane was obtained using phosphorous pentoxide, and then distilling when needed. Deuterated solvents for NMR analysis were purchased from Sigma Aldrich.

NMR:

^{1}H NMR, ^{13}C NMR and ^{19}F NMR spectra were measured on Bruker DPX 300, 400 or 500 apparatus and were referenced to the residual solvent peak (^{1}H: $CDCl_3$, δ 7.26 ppm; DMSO-d_6, δ 2.50 ppm; MeOH-d_4, δ 3.31 ppm) and solvent ^{13}C signal ($CDCl_3$, δ 77.2 ppm, DMSO-d_6, δ 39.5, MeOH-d_4, δ 49.0). For the ^{19}F spectra, ethyl fluoroacetate served as external standard (δ = −231.1ppm). [1] Chemical shifts (δ) were reported in ppm, multiplicity of the signals was declared as followed: s = singlet, d = doublet, t = triplet, q = quartet, quin = quintet, sex = sextet, hep = septet, dd = doublet of doublets, m = multiplet, b = broad; and coupling constants (J) in Hertz.

High resolution mass spectrometry (HRMS, m/z):

Data was obtained from Cardiff University on a Water LCR Premier XE- TOF. Ions were generated by the Atmospheric Pressure Ionisation Techniques (APCI): Electrospray (ESI), Electron Ionisation (EI).

Infrared spectra:

IR spectra were recorded on a Shimadzu FTIR Affinity-1S apparatus. Wavenumbers are quoted in cm^{-1}. All compounds were measured neat directly on the crystal of the IR machine. Melting points were measured using a Gallenkamp variable heater with samples in open capillary tubes.

Electrolysis:

The electrochemical reactions were carried out in a galvanostatic mode using a GWINSTEK GPR-30H10D galvanostat. Electrolysis experiments were carried out using a Vapourtec Ion Electrochemical Reactor. Electrode materials: Platinum, Glassy Carbon (GC), Graphite, Nickel, Boron doped diamond (BDD) were sourced from Goodfellow (http://www.goodfellow.com/). The electrodes (5 x 5 cm^2) are separated by a 0.5 mm spacer with a channel volume of 0.6 mL and an exposed electrode surface area of 12 cm^2 (each electrode). The syringe pumps that were used were KR Analytical Ltd Fusion 100 Touch syringe pumps.

Cyclic Voltammetry:

The cyclic voltammogram studies were either performed in a Orygalys OGF500 Potentiostat / Galvanostat with OGFPWR power supply or using the IKA Electrasyn 2.0.

Warning: Use of HF reagents:

The hazards of hydrogen fluoride solutions are well categorised and can be viewed in this SDS (https://www.sigmaaldrich.com/catalog/product/aldrich/184225). Personal protection is seriously important. It is advised to wear two pairs of thick nitrile gloves when handling. If the gloves come into contact with HF, they should be removed immediately, the area affected washed thoroughly, treated with calcium gluconate gel and medical attention sought.

Preparation of HF.amine stock solutions HF:

Amine stock solutions were made prior to every reaction and stirred for 30 mins before use. For example:

1 mL of a 5.6HF.amine stock solution by diluting Py.9HF (Olah's reagent) with 3HF•NEt_3 (0.44 mL of 9HF•Py and 0.56 mL of 3HF•NEt_3).

1 mL of 7HF.amine stock solution made by diluting Py.9HF (Olah's reagent) with NEt_3.3HF (0.68 mL of 9HF•Py and 0.32 mL of 3HF•NEt_3).

General Procedures for the Synthesis of the Starting Materials

General Procedure A for the Synthesis of Allyl Amides:

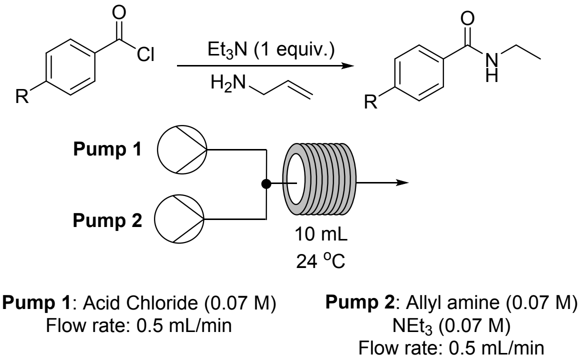

The procedure is analogous to the literature procedure but performed in flow rather than batch.[1] A solution of the carboxylic acid chloride derivative (1 equiv.) in dried dichloromethane and a solution of NEt_3 (1 equiv.) and allylamine (1 equiv.) in dried dichloromethane were pumped through a 10 mL (1 mm internal diameter) flow reactor. The reaction mixture was collected for the desired period of time. The reaction solution was washed with water (50 mL) and brine (50 mL) and the organic layer dried over $MgSO_4$ and filtered. The solvent was concentrated *in-vacuo* to afford the pure product.

General Procedure B for the Synthesis of Allyl Alcohols:

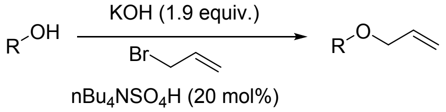

The procedure is analogous to the literature procedure.[2] To a solution of the alcohol (1 equiv.) in allyl bromide (1 equiv.) was added KOH (1.9 equiv.), and tetrabutylammonium bisulfate (20 mol%). The mixture was stirred at room temperature until the reaction was shown to be complete by TLC analysis. Water (20 mL) was then added, and the aqueous layer was extracted with diethyl ether (20 mL, 3-5 times). The combined organic layers were washed once with water (30 mL), and once with brine (30 mL) before being dried over magnesium sulfate, filtered, and concentrated *in-vacuo* to afford the crude product. The crude product was purified by silica gel flash column chromatography.

General Procedure C for the Synthesis of Esters:

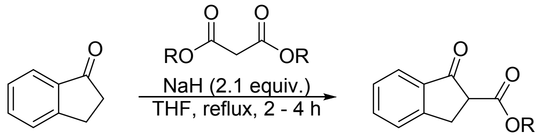

The procedure is analogous to the literature procedure.[3] A solution of indanone (1.0 equiv.) in abs. THF (1 L mol^{-1}) was added dropwise to a stirred suspension of $CH_2(COOR)_2$ (5.0 equiv.) and NaH (60 % dispersion in mineral oil, 2.1 equiv.) in abs. THF (2 L mol^{-1}) under a nitrogen atmosphere. The mixture was heated to reflux or stirred at ambient temperature (2 – 4 h), cooled in an ice bath and then acidified with hydrochloric acid. The residue was then extracted with Et_2O (3 x 30 mL). The combined organic layers were dried over $MgSO_4$, filtered and the solvent was removed *in vacuo*. The crude product was purified by silica gel flash column chromatography.

Characterisation of the Substrates:

_N_-Allyl-4-methylbenzamide (1a)

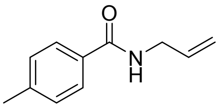

Title compound was prepared according to general procedure **A** and purified by column chromatography (SiO_2; gradient starting with 100% cyclohexane and increasing up to 75% ethyl acetate) affording a colourless solid (1.20 g, 98%).

^{1}H NMR (400 MHz, $CDCl_3$) δ 7.68 (d, *J* = 8.2 Hz, 2H), 7.20 (d, *J* = 8.5 Hz, 2H), 6.45 (bs, 1H), 5.91 (ddt, *J* = 17.3, 10.3, 5.6 Hz, 1H), 5.20 (dq, *J* = 17.2, 1.5 Hz, 1H), 5.14 (dq, *J* = 10.2, 1.5 Hz, 1H) 4.05 (t, *J* = 5.7 Hz, 2H), 2.37 (s, 3H).

^{13}C NMR (101 MHz, $CDCl_3$) δ 167.5, 142.0, 134.4, 131.7, 129.3, 127.1, 116.6, 42.5, 21.5.

MP: 75 – 76 ^{o}C.

NMR data is identical to the literature data.[1]

_N_-Allylbenzamide (1b)

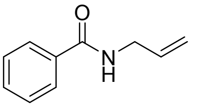

Title compound was prepared according to general procedure **A** and purified by column chromatography (SiO_2; hexanes: ethyl acetate, 3:2) affording a colourless oil (1.09 g, 97%).

^{1}H NMR (500 MHz, $CDCl_3$) δ 7.81 – 7.76 (m, 2H), 7.52 – 7.43 (m, 1H), 7.43 – 7.37 (m, 2H), 5.83 (ddt, *J* = 17.1, 10.3, 5.6 Hz, 1H), 5.15 (dq, *J* = 17.2, 1.6 Hz, 1H), 5.06 (dq, *J* = 10.2, 1.4 Hz, 1H), 3.96 (tt, *J* = 5.7, 1.6 Hz, 2H).

^{13}C NMR (101 MHz, $CDCl_3$) δ 167.7, 134.3, 134.2, 131.3, 128.3, 127.1, 116.1, 42.3.

NMR data is identical to the literature data.[1]

_N_-Allyl-4-nitrobenzamide (1c)

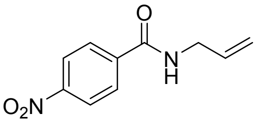

Title compound was prepared according to general procedure **A** and purified by column chromatography (SiO_2; gradient starting with 100% cyclohexane and increasing up to 75% ethyl acetate) affording a colourless solid (1.37 g, 95%).

^{1}H NMR (400 MHz, $CDCl_3$) δ 8.30 (d, *J* = 8.7 Hz, 2H), 7.95 (d, *J* = 8.7 Hz, 2H), 6.21 (bs, 1H), 5.95 (ddt, *J* = 17.1, 10.2, 5.9 Hz, 1H), 5.30 (dq, *J* = 17.1, 1.5 Hz, 1H), 5.24 (dq, *J* = 10.2, 1.2 Hz, 1H), 4.12 (tt, *J* = 5.8, 1.3 Hz, 2H).

^{13}C NMR (126 MHz, $CDCl_3$) δ 165.4, 149.8, 140.2, 133.6, 128.4, 124.1, 117.7, 43.1.

MP: 120 – 121 ^{o}C.

NMR data is identical to the literature data.[1]

***N*-Allyl-4-(trifluoromethyl)benzamide (1d)**

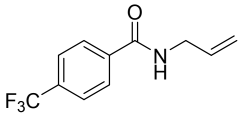

Title compound was prepared according to general procedure **A** and purified by column chromatography (SiO_2; gradient starting with 100% cyclohexane and increasing up to 75% ethyl acetate) affording a colourless solid (1.56 g, 97%).

^{1}H NMR (500 MHz, $CDCl_3$) δ 7.89 (d, *J* = 8.6 Hz, 2H), 7.71 (d, *J* = 8.7 Hz, 2H), 6.19 (bs, 1H), 5.95 (ddt, *J* = 17.1, 10.2, 5.8 Hz, 1H), 5.29 (dq, *J* = 17.2, 1.5 Hz, 1H), 5.22 (dq, *J* = 10.2, 1.3 Hz, 1H), 4.12 (tt, *J* = 5.8, 1.5 Hz, 2H).

^{19}F NMR (376 MHz, $CDCl_3$) δ -62.96 (s, 3F).

^{13}C NMR (126 MHz, $CDCl_3$) δ 166.2, 137.9, 133.9, 133.5 (d, *J* = 32.7 Hz), 127.6, 126.0 (q, *J* = 3.76 Hz), 123.9 (d, *J* = 272.55 Hz), 117.5, 42.8.

MP: 103 – 104 °C.

NMR data is identical to the literature data.[1]

***N*-Allyl-4-methoxybenzamide (1e)**

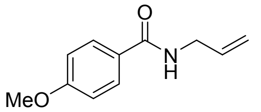

Title compound was prepared according to general procedure **A** and purified by column chromatography (SiO_2; gradient starting with 100% cyclohexane and increasing up to 75% ethyl acetate) affording a colourless oil (1.33 g, 99%).

^{1}H NMR (500 MHz, $CDCl_3$) δ 7.79 – 7.72 (m, 2H), 6.92 – 6.86 (m, 2H), 6.33 (bs, 1H), 5.91 (ddt, *J* = 17.1, 10.2, 5.7 Hz, 1H), 5.23 (dq, *J* = 17.1, 1.7 Hz, 1H), 5.15 (dq, *J* = 10.2, 1.4 Hz, 1H), 4.05 (tt, *J* = 5.7, 1.6 Hz, 2H), 3.83 (s, 3H).

^{13}C NMR (126 MHz, $CDCl_3$) δ 167.0, 162.3, 134.5, 128.9, 126.9, 116.6, 113.9, 55.5, 42.5.

NMR data is identical to the literature data.[1]

***N*-Allyltetrahydrofuran-2-carboxamide (1i)**

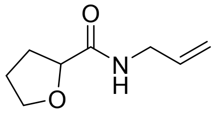

Title compound was prepared according to general procedure **A** and purified by column chromatography (SiO_2; gradient starting with 100% cyclohexane and increasing up to 100% ethyl acetate) affording a reddish oil (1.34 g, 92%).

^{1}H NMR (500 MHz, $CDCl_3$) δ 6.75 (broad, 1H), 5.77 (ddt, *J* = 17.1, 10.3, 5.6 Hz, 1H), 5.09 (ddq, *J* = 17.0, 10.3, 1.5 Hz, 2H), 4.30 (dd, *J* = 8.4, 5.8 Hz, 1H), 3.93 – 3.78 (m, 4H), 2.28 – 2.18 (m, 1H), 2.13 – 1.63 (m, 3H).

^{13}C NMR (126 MHz, $CDCl_3$) δ 173.7 (s), 133.9 (s), 116.6 (s), 78.5 (s), 69.5 (s), 41.2 (s), 30.3 (s), 25.6 (s).

N-allylmorpholine-4-carboxamide (1j)

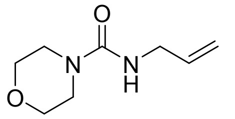

Title compound was prepared according to general procedure **A** and purified by column chromatography (SiO_2; gradient starting with 100% cyclohexane and increasing up to 100% ethyl acetate) affording a white solid (1.19 g, 96%).

^{1}H NMR (400 MHz, $CDCl_3$) δ 5.86 (ddt, *J* = 15.9, 10.4, 5.7 Hz, 1H), 5.17 (dd, *J* = 17.2, 1.5 Hz, 1H), 5.10 (dd, *J* = 10.2, 1.3 Hz, 1H), 3.86 (dt, *J* = 5.6, 1.3 Hz, 2H), 3.72 – 3.65 (m, 4H), 3.38 – 3.33 (m, 4H).

^{13}C NMR (101 MHz, $CDCl_3$) δ 157.7 (s), 135.4 (s), 116.2 (s), 66.6 (s), 44.3 (s), 43.6 (s).

MP: 112 – 114 °C

N-allylpyrrolidine-1-carboxamide (1k)

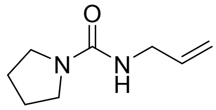

Title compound was prepared according to general procedure **A** and purified by column chromatography (SiO_2; gradient starting with 100% cyclohexane and increasing up to 100% ethyl acetate) affording a pale yellow oil (0.63 g, 90%).

^{1}H NMR (300 MHz, $CDCl_3$) δ 5.87 (ddd, *J* = 15.8, 10.7, 5.6 Hz, 1H), 5.11 (dd, *J* = 25.3, 13.7 Hz, 2H), 4.50 (broad, 1H), 3.86 (dd, *J* = 5.6, 1.3 Hz, 2H), 3.46 – 3.26 (m, 4H), 2.01 – 1.72 (m, 4H).

^{13}C NMR (75 MHz, $CDCl_3$) δ 156.7 (s), 135.8 (s), 115.8 (s), 46.0 (s), 43.4 (s), 25.7 (s).

Benzyl 4-(allylamino)-4-oxobutanoate (1l)

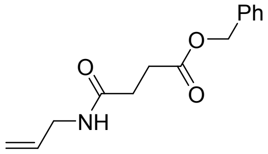

To a solution of allylamine (0.8 mL, 10.5 mmol, 2.1 equiv.) in THF (5 mL) at 0 °C was added succinic anhydride (500 mg, 5.0 mmol, 1.0 equiv.) in three portions. The reaction mixture was stirred for 3 h after slowly warming to ambient temperature. The volatile substances were evaporated *in vacuo* and the residue was dissolved in THF (5 mL). Benzyl bromide (1.5 mL, 12.5 mmol, 2.5 equiv.) and triethylamine (0.8 mL, 6.0 mmol, 1.2 equiv.) were added to the solution and the reaction mixture was refluxed for 8 h before being allowed to cool to ambient temperature. The mixture was poured into aqueous HCl (1 M) and the aqueous phase was extracted with dichloromethane (3 × 20 mL). The combined organic phases were dried over MgSO4 and the solvent was removed in vacuo. The residue was purified by column chromatography (cyclohexane : ethyl acetate, 1:1). The product (430 mg, 1.7 mmol, 34%) was obtained as a colourless solid.

^{1}H NMR (300 MHz, $CDCl_3$) δ 7.42 – 7.30 (m, 5H), 5.82 (t, *J* = 5.6 Hz, 1H) 5.80 (ddt, *J* = 17.1, 10.3, 5.6 Hz, 1H), 5.15 (dq, *J* = 30.3, 1.5 Hz, 1H), 5.15-5.12 (m, 3H), 3.86 (tt, *J* = 5.7, 1.5 Hz, 2H), 2.74 (t, *J* = 5.7 Hz, 2H), 2.50 (t, *J* = 6.8 Hz, 2H).

^{13}C NMR (75 MHz, $CDCl_3$) δ 173.0 (s), 171.4 (s), 135.9 (s), 134.2 (s), 128.7 (s), 128.5 (s), 128.4 (s), 116.5 (s), 66.7 (s), 42.2 (s), 31.1 (s), 29.8 (s).

MP: 42 – 44 °C

NMR data is identical to the literature data.[21]

***N*-Allylpiperidine-1-carboxamide (1m)**

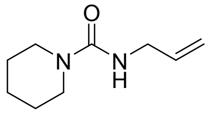

Title compound was prepared according to general procedure **A** and purified by column chromatography (SiO_2; gradient starting with 100% cyclohexane and increasing up to 100% ethyl acetate) affording a pale-yellow oil (0.72 g, 91%).

^{1}H NMR (400 MHz, $CDCl_3$) δ 5.90 – 5.77 (m, 1H), 5.15 (dd, *J* = 31.8, 13.6 Hz, 2H), 3.84 (d, *J* = 4.1 Hz, 2H), 3.32 (t, *J* = 4.8 Hz, 4H), 1.78 – 1.55 (m, 6H).

^{13}C NMR (101 MHz, $CDCl_3$) δ 156.0 (s), 134.2 (s), 116.7 (s), 47.5 (s), 43.7 (s), 24.6 (s), 23.5 (s).

1-((Allyloxy)methyl)-4-fluorobenzene (6aa)

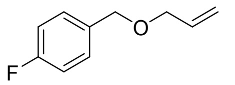

Title compound was prepared according to general procedure **B** and purified by column chromatography (SiO_2; hexanes: ethyl acetate, 95:5) affording a colourless oil (448 mg, 27%).

^{1}H NMR (500 MHz, $CDCl_3$) δ 7.37 – 7.27 (m, 2H), 7.03 – 6.95 (m, 2H), 5.96 (ddt, *J* = 16.1 10.4, 5.6 Hz, 1H), 5.31 (dq, *J* = 17.2, 1.6 Hz, 1H), 5.22 (dq, *J* = 10.4, 1.3 Hz, 1H), 4.49 (s, 2H), 4.03 (dt, *J* = 5.8, 1.3 Hz, 2H).

^{13}C NMR (101 MHz, $CDCl_3$) δ 164.5 (d, *J* = 238.8 Hz) 134.7, 134.2 (d, *J* = 3.1 Hz), 129.6 (d, *J* = 8.1 Hz), 117.5, 115.4 (d, *J* = 21.4 Hz), 71.5, 71.3.

NMR data is identical to the literature data.[2]

1-((Allyloxy)methyl)-3,5-dinitrobenzene (6ba)

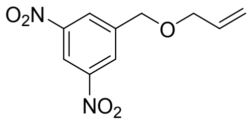

Title compound was prepared according to general procedure **B** and purified by column chromatography (SiO_2; hexanes: ethyl acetate, 8:2) affording a dark orange oil (857 mg, 36%).

^{1}H NMR (400 MHz, $CDCl_3$) δ 8.96 (t, *J* = 2.1 Hz, 1H), 8.55 (d, *J* = 2.1 Hz, 2H), 5.97 (ddt, *J* = 17.2, 10.4, 5.7 Hz, 1H), 5.37 (dq, *J* = 17.2, 1.6 Hz, 1H), 5.30 (dq, *J* = 10.2, 1.2 Hz, 1H), 4.70 (m, 2H), 4.16 (dt, *J* = 5.7, 1.4 Hz, 2H).

^{13}C NMR (101 MHz, $CDCl_3$) δ 148.7, 143.5, 133.8, 127.2, 118.6, 118.0, 72.4, 69.8.

NMR data is identical to the literature data.[2]

1-((Allyloxy)methyl)-2-methylbenzene (6ca)

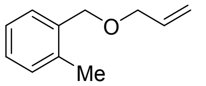

Title compound was prepared according to general procedure **B** and purified by column chromatography (SiO_2; hexanes: diethyl ether, 9:1) affording a colourless oil (292 mg, 18%).

^{1}H NMR (400 MHz, $CDCl_3$) δ 7.34 (d, *J* = 5.2 Hz, 2H), 7.25 – 7.14 (m, 3H), 5.98 (ddt, *J* = 16.0, 11.2, 5.6 Hz, 1H), 5.33 (dq, *J* = 17.2, 1.6 Hz, 1H), 5.26 – 5.20 (m, 1H), 4.53 (s, 2H), 4.06 (d, *J* = 5.6 Hz, 2H), 2.35 (s, 3H).

^{13}C NMR (101 MHz, $CDCl_3$) δ 136.8, 136.4, 135.1, 130.4, 128.7, 127.9, 126.0, 117.2, 71.4, 70.7, 19.0.

NMR data is identical to the literature data.[2]

1-((Allyloxy)methyl)-2-bromobenzene (6da)

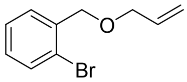

Title compound was prepared according to general procedure **B** and purified by column chromatography (SiO_2; hexanes 100%) affording a colourless oil (1.32 g, 58%).

^{1}H NMR (400 MHz, $CDCl_3$) δ 7.54 (dd, *J* = 8.0, 1.1 Hz, 1H), 7.52 – 7.49 (m, 1H), 7.36 – 7.29 (m, 1H), 7.16 – 7.10 (m, 1H), 5.99 (ddt, *J* = 17.2, 10.4, 5.6 Hz, 1H), 5.36 (dq, *J* = 17.2, 1.6 Hz, 1H), 5.24 (ddt, *J* = 10.4, 1.6, 1.2 Hz, 1H), 4.59 (s, 2H), 4.12 (dt, *J* = 5.6, 1.4 Hz, 2H).

^{13}C NMR (101 MHz, $CDCl_3$) δ 137.9, 134.7, 132.7, 129.2, 129.0, 127.6, 122.8, 117.5, 71.9, 71.6.

NMR data is identical to the literature data.[2]

2-((Allyloxy)methyl)pyridine (6ea)

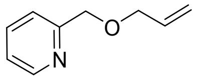

Title compound was prepared according to general procedure **B** and purified by column chromatography (SiO_2; hexanes: diethyl ether, 7:3) affording a dark orange oil (478 mg, 32%).

^{1}H NMR (300 MHz, $CDCl_3$) δ 8.54 (d, *J* = 4.4 Hz, 1H), 7.68 (td, *J* = 7.7, 1.8 Hz, 1H), 7.45 (d, *J* = 7.8 Hz, 1H), 7.17 (dd, *J* = 7.2, 5.2 Hz, 1H), 5.97 (ddt, *J* = 17.2, 10.5, 5.6 Hz, 1H), 5.33 (dq, *J* = 17.2, 1.6 Hz, 1H), 5.22 (dq, *J* = 10.4, 1.3 Hz, 1H), 4.64 (s, 2H), 4.11 (dt, *J* = 5.6, 1.4 Hz, 2H).

^{13}C NMR (126 MHz, $CDCl_3$) δ 158.7, 149.3, 136.8, 134.6, 122.5, 121.5, 117.6, 73.1, 72.0.

NMR data is identical to the literature data.[2]

((2-Methylallyloxy)methyl)benzene (6fa)

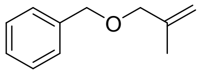

Title compound was prepared from 2-methylprop-2-en-1-ol (10 mmol) and benzyl bromide (15 mmol) according to general procedure **B** and purified by column chromatography (SiO_2; hexanes 100%) affording a colourless oil (356 mg, 22%).

^{1}H NMR (300 MHz, $CDCl_3$) δ 7.40 – 7.27 (m, 5H), 4.98 (d, *J* = 24.2 Hz, 2H), 4.51 (s, 2H), 3.97 – 3.93 (m, 2H), 1.78 (s, 3H).

^{13}C NMR (75 MHz, $CDCl_3$) δ 142.4, 138.6, 128.5, 127.9, 127.7, 112.5, 74.3, 72.0, 19.8.

NMR data is identical to the literature data.[2]

1-(((2-Methylallyl)oxy)methyl)-4-(trifluoromethyl)benzene (6ga)

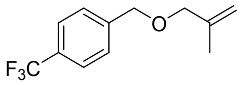

Title compound was prepared from 2-methylprop-2-en-1-ol (10 mmol) and 1- (bromomethyl)-4-(trifluoromethyl)benzene (15 mmol) according to general procedure **B** and purified by column chromatography (SiO_2; hexanes 100%)) affording a colourless oil (1.52 g, 66%).

^{1}H NMR (300 MHz, $CDCl_3$) δ 7.61 (d, *J* = 8.1 Hz, 2H), 7.47 (d, *J* = 8.0 Hz, 2H), 4.98 (d, *J* = 18.5 Hz, 2H), 4.55 (s, 2H), 3.96 (s, 2H), 1.78 (s, 3H)

^{13}C NMR (75 MHz, $CDCl_3$) δ 142.8, 142.0, 129.7 (q, *J* = 32.3 Hz), 127.7, 125.5, 124.3, 112.8, 74.6, 71.1, 19.7.

NMR data is identical to the literature data.[2]

Ethyl 1-oxo-2,3-dihydro-1H-indene-2-carboxylate (7ca)

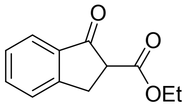

Title compound was prepared according to general procedure **C** and purified by column chromatography (SiO_2; hexanes / EtOAc, 9:1) affording a purple oil (1.63 g, 80%).

Isolated as a mixture of the keto and enol form (4.5:1)

^{1}H NMR (400 MHz, $CDCl_3$) δ 7.77 (d, *J* = 7.7 Hz, 1H; ArH), 7.62 (t, *J* = 8.0 Hz, 1H; ArH), 7.50 (d, *J* = 6.9 Hz, 1H, ArH), 7.39 (t, *J* = 7.5 Hz, 1H; ArH), 4.32 (q, *J* = 7.1 Hz, 0.46H; CH_2O - enol), 4.25 (q, *J* = 7.1 Hz, 2H; CH_2O), 3.71 (dd, *J* = 8.3, 4.1 Hz, 1H; CHC=O), 3.58 (s, 0.46H; CH_2 - enol), 3.53 (dd, *J* = 17.2, 4.1 Hz, 1H; CH_2), 3.37 (dd, *J* = 17.2, 8.3 Hz, 1H; CHC=O), 1.36 (t, *J* = 7.1 Hz, 0.68H; CH_3 - enol), 1.31 (t, *J* = 7.1 Hz, 3H; CH_3).

^{13}C NMR (126 MHz, $CDCl_3$) δ 199.7, 169.3, 153.8, 135.5, 135.4, 127.9, 126.7, 124.8, 61.9, 53.5, 30.4, 14.3. Minor peaks due to enol observed.

NMR data is identical to the literature data.[3]

Methyl 1-oxo-2,3-dihydro-1H-indene-2-carboxylate (7da)

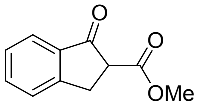

Title compound was prepared according to general procedure **C** and purified by column chromatography (SiO_2; hexanes / EtOAc, 9:1) affording an orange solid (1.37 g, 72%).

Isolated as a mixture of the keto and enol form (6:1).

^{1}H NMR (400 MHz, $CDCl_3$) δ 10.37 (bs, 0.16 H; OH -enol) 7.77 (d, *J* = 7.7 Hz, 1H; ArH), 7.62 (t, *J* = 7.5 Hz, 1H; ArH), 7.50 (d, *J* = 7.7 Hz, 1H; ArH), 7.39 (t, *J* = 7.4 Hz, 1H; ArH), 3.85 (s, 0.52 H; OCH_3 - enol), 3.79 (s, 3H; OCH_3), 3.74 (dd, *J* = 8.3, 4.0 Hz, 1H; CHCO), 3.56 (dd, *J* = 17.2, 3.9 Hz, 1H; CH_2), 3.51 (s, 0.3 H; CH_2 - enol), 3.37 (dd, *J* = 17.3, 8.2 Hz, 1H; CH_2).

^{13}C NMR (101 MHz, $CDCl_3$) δ 199.6, 169.8, 153.8, 135.6, 128.0, 126.7, 124.9, 53.3, 53.0, 30.4. Minor peaks due to enol observed.

MP: 139 – 145 °C (decomposition).

NMR data is identical to the literature data.[4]

3-(4-Nitrophenyl)but-3-en-1-ol (9aa)

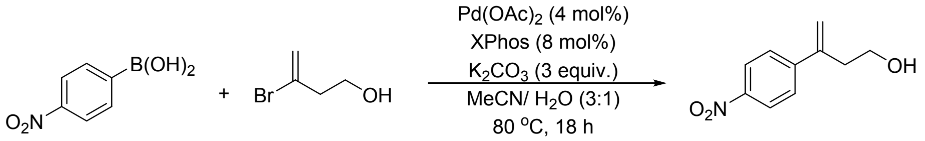

4-Nitroboronic acid (1.5 equiv.), K_2CO_3 (3 equiv.), $Pd(OAc)_2$ (4 mol%) and XPhos (8 mol%) were weighted in a round bottom flask. The flask was equipped with a reflux condenser, evacuated, and backfilled with inert gas. The solvent was added, followed by the 3-bromobut-3-en-1-ol (1.0 equiv.). The reaction mixture was stirred at 80 °C for 18 h. After cooling to room temperature, the reaction mixture was filtered using EtOAc as the rinsing solvent. The filtrate was washed twice with brine, dried over $MgSO_4$, filtered, and concentrated under reduced pressure. The product was purified by column chromatography (SiO_2; hexanes : ethyl acetate, 3:2) affording an orange oil (0.42 g, 72%).

^{1}H NMR (300 MHz, $CDCl_3$) δ 8.18 (d, *J* = 8.9 Hz, 2H), 7.56 (d, *J* = 8.9 Hz, 2H), 5.55 – 5.54 (m, 1H), 5.36 – 5.34 (m, 1H), 3.75 (t, *J* = 6.5 Hz, 2H), 2.80 (t, *J* = 6.5 Hz, 2H), 1.66 (sbr, 1H).

^{13}C NMR (75 MHz, $CDCl_3$) δ 147.4, 147.3, 143.5, 127.1, 123.9, 118.1, 61.0, 38.2.

HRMS (CI+): calc: $[M+H]^+$ ($C_{10}H_{12}NO_3$) 194.08117; measured: 194.0815.

NMR data is identical to the literature data.[5]

2-Phenylpent-4-enoic acid (10aa)

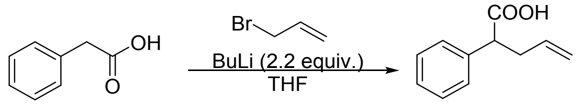

A solution of n-butyllithium (2.5 M in hexanes, 32.3 mL, 80.8 mmol, 2.2 equiv.) was added dropwise to a solution of phenylacetic acid (5.00 g, 36.72 mmol, 1.0 equiv.) in THF (150 mL) at –78 °C under nitrogen atmosphere. The reaction mixture was allowed to warm to room temperature and stirred for 45 minutes. Neat allyl bromide (12.7 mL, 146.9 mmol, 4.0 equiv.) was added and the resulting solution was stirred at room temperature for 16 h. The reaction mixture was quenched with aqueous 1M HCl solution and extracted with Et_2O (3 x 30 mL). The combined organic layers were washed with brine, dried over $MgSO_4$, filtered, and concentrated under reduced pressure to affording a pale-yellow oil (6.30 g, 97%).

^{1}H NMR (500 MHz, $CDCl_3$) δ 7.57 – 7.05 (m, 5H), 5.65 (ddt, *J* = 17.0, 10.2, 6.8 Hz, 1H), 5.01 (dq, *J* = 17.1, 1.5 Hz, 1H), 4.94 (dd, *J* = 10.2, 1.5 Hz, 1H), 3.57 (dd, *J* = 8.3, 7.1 Hz, 1H), 2.91 – 2.60 (m, 1H), 2.60 – 2.28 (m, 1H).

^{13}C NMR (126 MHz, $CDCl_3$) δ 179.9, 138.0, 135.0, 128.9, 128.3, 127.8, 117.5, 51.6, 37.2.

NMR data is identical to the literature data.[6]

2-(Pent-4-en-1-yl)isoindoline-1,3-dione (14a)

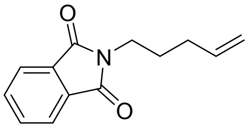

5 Bromopent-1-ene (9.5 mL, 80 mmol) and potassium phthalimide (16.3 g, 88 mmol) was added to 160 mL of dry DMF in a flame-dried Schlenk tube containing a magnetic stir bar. The flask was purged with argon (3 times) and the solution heated to 60 ^{o}C for 24 h with stirring. After cooling, the solution was filtered, washed with H_2O (3 x 200 mL), extracted with CH_2Cl_2 (3 x 200 mL) and dried over magnesium sulfate. The resulting solution was dried *in vacuo* and purified by silica gel column chromatography (EtOAc: cyclohexane 1:15) to obtain the pure product as a colourless oil (15.84 g, 92%)

^{1}H NMR (400 MHz, $CDCl_3$) δ 7.83 (dd, *J* = 5.4, 3.1 Hz, 2H), 7.70 (dd, *J* = 5.5, 3.0 Hz, 2H), 5.81 (ddt, *J* = 16.9, 10.2, 6.6 Hz, 1H), 5.01 (dddd, *J* = 26.7, 10.2, 3.3, 1.4 Hz, 2H), 3.69 (t, *J* = 7.2 Hz, 2H), 2.17 – 2.06 (m, 2H), 1.83 – 1.74 (m, 2H).

^{13}C NMR (126 MHz, $CDCl_3$) δ 168.6, 137.5, 134.1, 132.3, 123.3, 115.5, 37.7, 31.1, 27.8.

NMR data is identical to the literature data.[22]

General Electrolysis Procedures

General Electrolysis Protocol D for Screening and Optimization Studies:

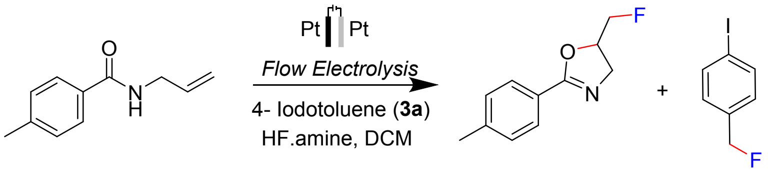

The electrolysis was performed in an undivided cell using a Vapourtec Ion Electrochemical Flow Reactor[15] (reactor volume = 0.6 mL, spacer 0.5 mm) using platinum electrodes (active surface area: A = 12 cm^2) as the anode and as the cathode. *N*-Allyl-4-methylbenzamide (0.1 M) and 4-iodotoluene (0.1 M) were dissolved in a mixture of dry dichloromethane and nHF.amine (1:1 v/v) and were electrolysed under constant current conditions (201 mA, 5 F, 0.25 mL/min). The exact electrolysis conditions depend on the parameter screened. The first one and a half reactor volumes of each run were discarded to ensure that a steady state had been reached and the result of the run was an accurate representation of the system. After collection for a known period of time, the mixture was stirred for a further 12 h in batch, and subsequently quenched with an ice-cooled saturated solution of Na_2CO_3. The organic phase is washed with water (30 mL) and brine (30 mL) and extracted with dichloromethane (3 x 30 mL). The organic phase was dried over $MgSO_4$, filtered, and the solvent removed under reduced pressure. Ethyl fluoroacetate (of a known amount) and 2 mL of $CDCl_3$ were added to the crude product and a ^{19}F NMR spectrum was measured. The amount of formed 5-(fluoromethyl)-2-(4-methylphenyl)-oxazoline was determined by the ratio of the fluorine signal of the standard ethyl fluoroacetate (δ = −231.1, 1F) to the oxazoline signal (δ = −228.7, 1F).

*Due to the more difficult purification of the (difluoroiodo)toluene, i.e. some of the I(III) is degraded (hydrolysis when exposed to moisture and air) upon isolation, emphasized by the difference in yield of the stable oxazoline (<95%) compared to 82% of the less stable I(III) upon isolation, we concluded that yield based on the stable oxazoline gave a better representation of the system.

General Electrolysis Procedures for the Synthesis of (Difluoroiodo)arenes:

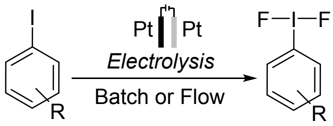

General Batch Electrolysis Procedure E:
The procedure is analogous to the literature procedure.[1] Batch electrolysis was performed using the IKA Electrasyn 2.0.[16] A solution of 0.5 mmol iodoarene in 2.5 mL dichloromethane and 2.5 mL amine·5.6HF, employing a platinum anode and a platinum cathode, was electrolyzed. The electrolysis was performed under constant current conditions (j = 50 mA·cm^{-2}, active surface area 1.2 cm^2, 3 F). After completion of electrolysis, the aqueous layer was extracted three times with 30 mL of dichloromethane, dried over $MgSO_4$ and filtered. The solvent was removed by N_2 bubbling at 25 ^oC, and the product washed 4-5 times with pentane. The remaining solvent was once again removed by N_2 bubbling at 25 ^oC to give the pure product.

General Flow Electrolysis Procedure F:
Flow electrolysis was performed using a Vapourtec Ion Electrochemical Flow Reactor (reactor volume = 0.6 mL, spacer 0.5 mm).[15] A solution of 4-iodotoluene in dichloromethane and amine·7HF (1:1 v/v) employing a platinum anode and a platinum cathode was electrolyzed. The electrolysis was performed under constant current conditions (j = 16.75 mA·cm^{-2}, active surface area 12 cm^2 for each electrode, 5 F) at a flow rate of 0.25 mL/min. The first one and a half reactor volumes of each run were discarded to ensure that a steady state had been reached and the result of the run was an accurate representation of the system. After completion of the electrolysis and collection of the solution for the desired period of time, the aqueous layer was extracted three times with dichloromethane, dried over $MgSO_4$, and filtered. The solvent was removed by N_2 bubbling at 25 ^oC,

and the product washed thoroughly with pentane. The remaining solvent was once again removed by N_2 bubbling at 25 °C to give the pure product.

General Electrolysis Procedures for Fluorination:

General Batch Electrolysis Procedure (in-cell) G:

The procedure is analogous to the literature procedure.[1] Batch electrolysis was performed using the IKA Electrasyn 2.0. [16] A solution of 0.5 mmol starting material and 0.5 mmol 4-iodotoluene in 2.5 mL dichloromethane and 2.5 mL amine·5.6HF, employing a platinum anode and a platinum cathode, was electrolyzed. The electrolysis was performed under constant current conditions (j = 50 mA cm^{-2}, active surface area 1.2 cm^2, 3 F). After completion of the electrolysis, the reaction mixture was allowed to stir for the recommended amount of time. The mixture was poured into 50 mL of an ice-cooled saturated solution of Na_2CO_3 and stirred for 20 minutes. The aqueous layer was extracted two times with 30 mL of dichloromethane, dried over $MgSO_4$ and filtered. After removal of the solvent, the crude product was further isolated by column chromatography.

General Batch Electrolysis Procedure (ex-cell) H:

The procedure is analogous to the literature procedure.[1] The synthesis is identical to the above procedure, except for the substrate being added to the reaction mixture after electrolysis took place.

General Flow Electrolysis Procedure (in-cell) I:

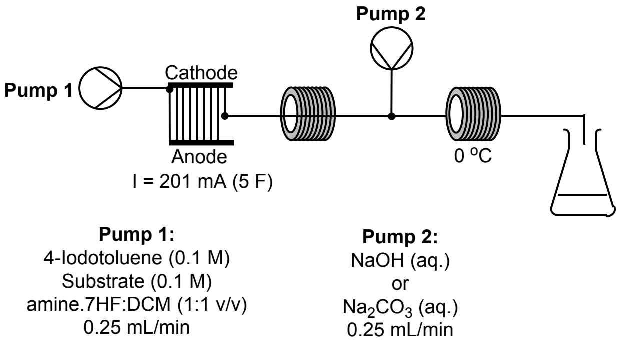

The procedure is analogous to the literature procedure.[7] Flow electrolysis was performed using a Vapourtec Ion Electrochemical Flow Reactor (reactor volume = 0.6 mL, spacer 0.5 mm).[15] The flow electrochemical reactor applying the general procedure **F** was joined to a PTFE coil (1 mm internal diameter) and a second syringe pump *via* a T-piece. A solution of starting material (1 equiv.) and 4-iodotoluene (1 equiv.) in dried dichloromethane and amine·7HF (1:1 v/v), employing a platinum anode and a platinum cathode (active surface area = 12 cm^2 for each electrode), was electrolyzed (j = 16.75 mA·cm^{-2}, 5 F) and allowed to flow through a PFTE coil for the corresponding residence time. A second pump and coil, connected *via* a T-piece, was employed for quenching purposes. NaOH/Na_2CO_3 was injected post reaction, and the reaction mixture allowed to stir for a further 15 minutes in at 0 °C flow. After reaching a steady state (1.5 reactor volumes) the solution was collected into a flask. DCM (30 mL) was added, the phases were separated, and the aqueous phase was extracted with DCM (3 x 20 mL). The combined organic layers were washed with water (2 x 20 mL) and dried over $MgSO_4$, filtered, and the solvent was removed under vacuum. The crude product was purified by column chromatography.

General Flow Electrolysis Procedure (ex-cell) J:

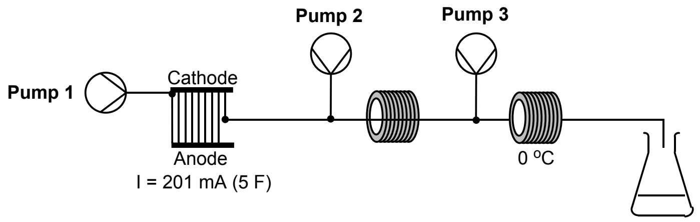

Pump 1:
4-Iodotoluene (0.1 M)
amine.7HF:DCM (1:1 v/v)
0.25 mL/min

Pump 2:
Substrate (0.1 M)
DCM
0.25 mL/min

Pump 3:
NaOH (aq.)
or
Na_2CO_3 (aq.)
0.5 mL/min

The procedure is analogous to the literature procedure.[7] The synthesis is identical to the above procedure, except for the substrate being injected into the reaction mixture after electrolysis took place.

General Automated Flow Electrolysis Procedure K:

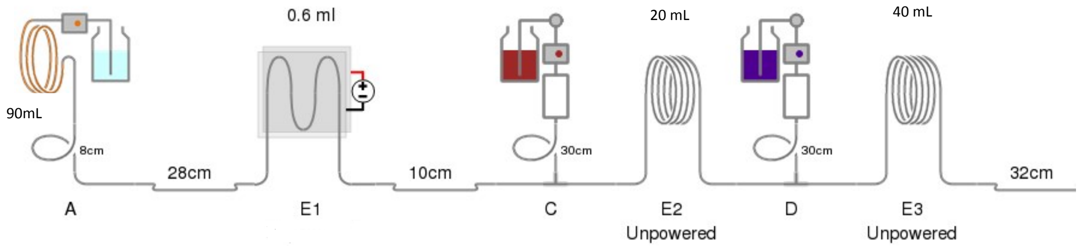

Pump A:
4-Iodotoluene (0.1 M)
7HF.amine:DCM (1:1 v/v)
1.0 mL/min

Pump C:
Substrate (0.07 M)
DCM
1.0 mL/min

Pump D:
$NaOH_{(aq.)}$ (3 M)
2.0 mL/min

The synthesis is identical to the above procedure **J**, except for the procedure being carried out in an automated electrolysis machine. 1.0 mL/min was used for the first 2 pumps (**Pump A and C)**, and 2.0 mL/min for **Pump D**. The substrate concentration was reduced to 0.07 M to account for the lower yield in the electrolysis step.

General Flow Electrolysis Procedure L:

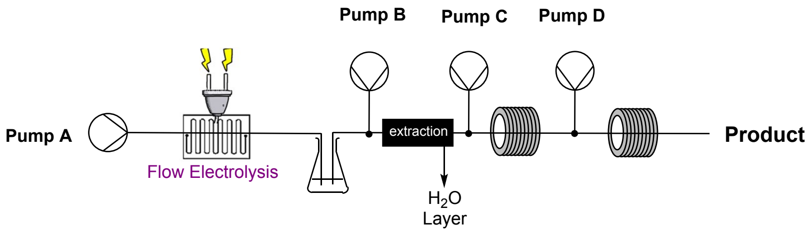

Pump A:
4-Iodotoluene (0.1 M)
7HF.amine:DCM (1:1 v/v)
0.25 mL/min

Pump B:
Water
0.25 mL/min

Pump C:
Substrate (0.07 M)
MeCN
Iodine
0.5 mL/min

Pump D:
$Na_2CO_{3(aq.)}$
1.0 mL/min

The procedure is analogous to the literature procedure.[7] The synthesis is identical to the ex-cell synthesis **J**, except for the in-line-liquid liquid extractor[23] and injection of water.

*In general gas production in flow can be a challenge whenever hydrogen evolution at the cathode is used as a balancing reaction. Back pressure regulators can ensure efficient gas-liquid mixing and minimise large gas bubble accumulations, or in-line gas liquid extractors are efficient. However, due to the high internal pressures within the developed system, no back pressure regulatorwas needed.

Electrochemical Batch Reaction Equipment:

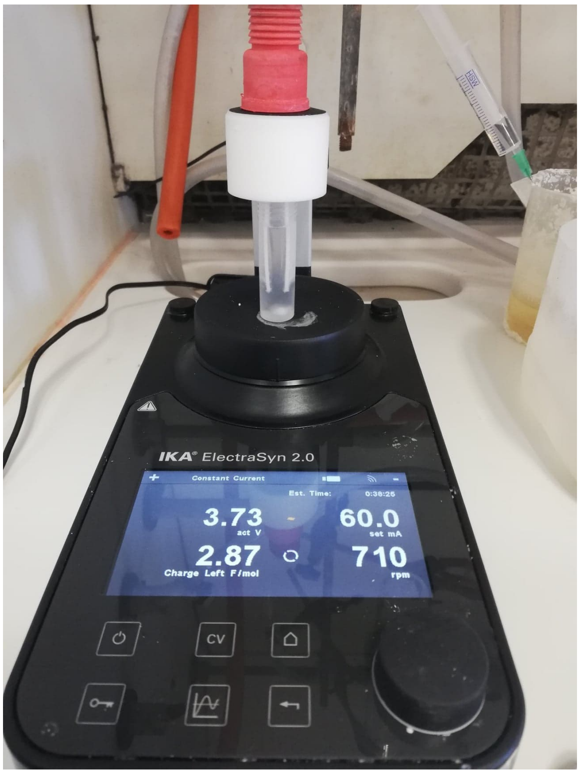

Figure S1: Batch electrolysis was performed using the IKA Electrasyn 2.0, utilizing two platinum electrodes, in a FEP vial.

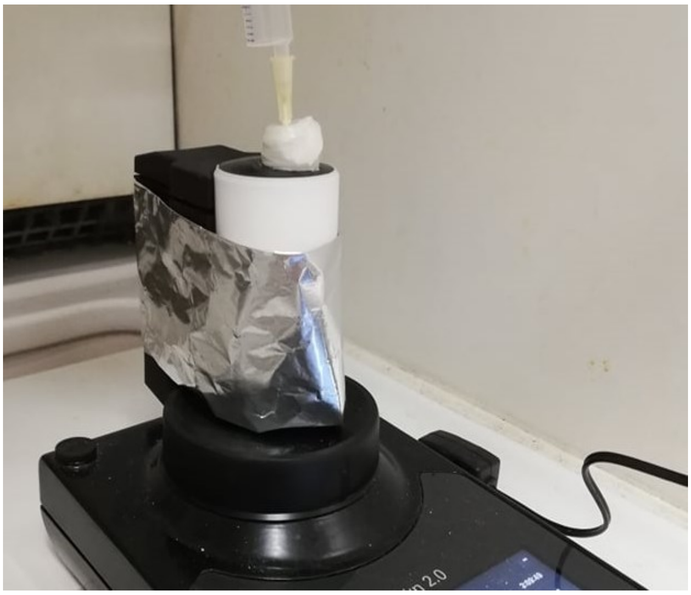

Figure S2: The electrochemical vial was shaded from light by a foil wrap. The fume cupboard light was switched off, and the vial purged with argon throughout.

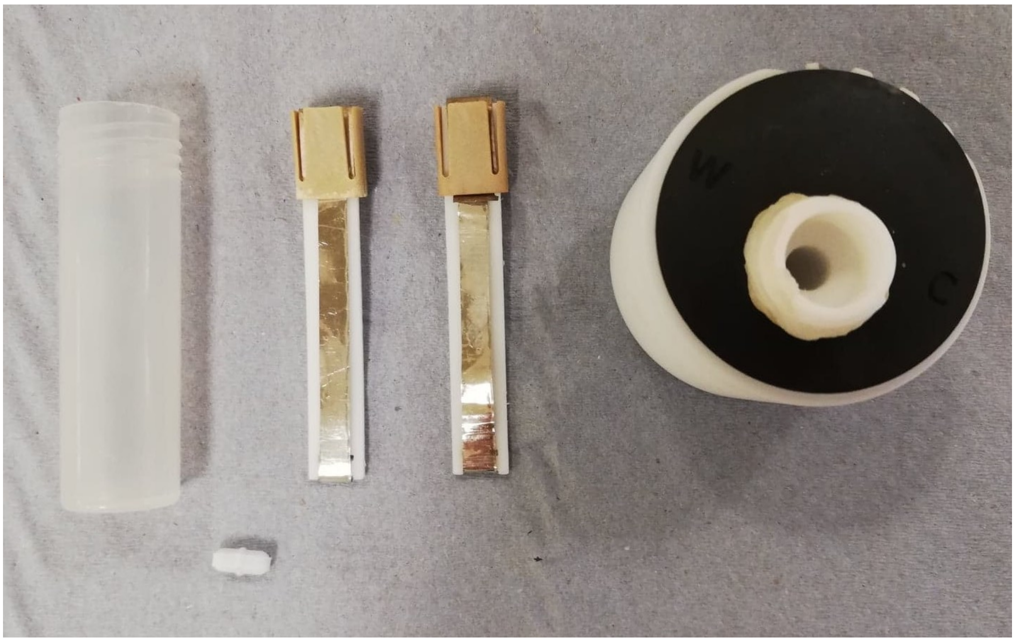

Figure S3: Batch electrolysis equipment; FEP vial (left), Pt electrodes (centre, submerged surface area 1.2 cm^2), stirrer bar (bottom) and IKA Electrasyn 2.0 lid (right).

Electrochemical Flow Reactor:

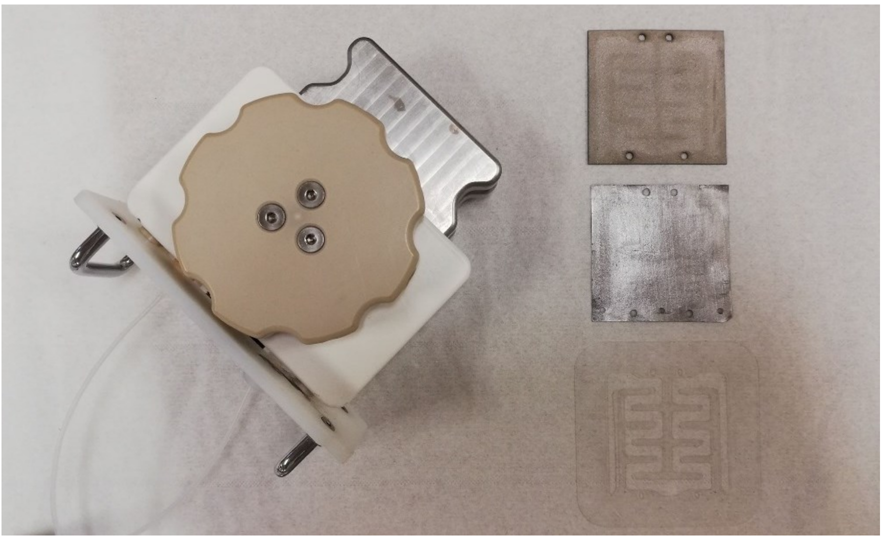

Figure S4: The Vapourtec Ion Electrochemical Flow Reactor (reactor volume = 0.6 mL, spacer 0.5 mm) – closed reactor.

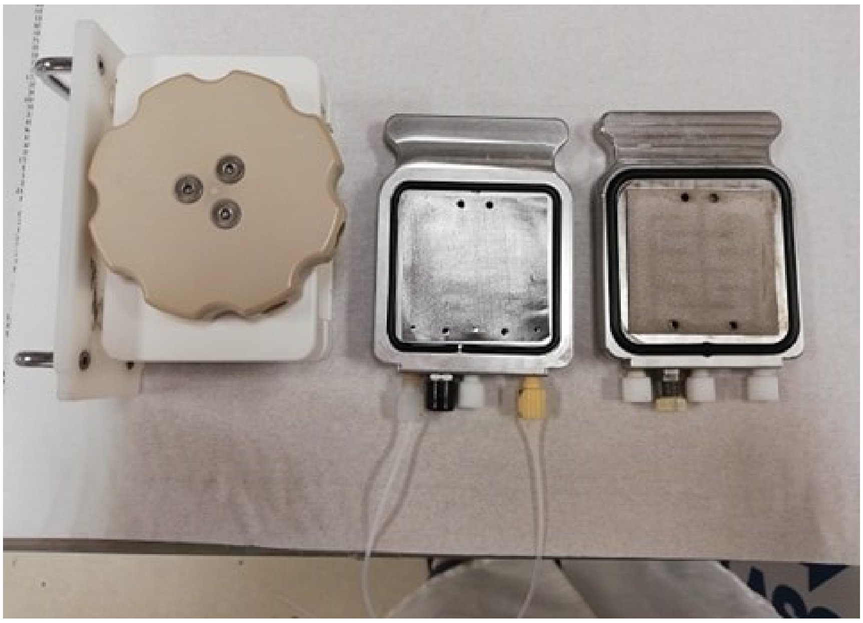

Figure S5: The Vapourtec Ion Electrochemical Flow Reactor – open reactor.

Automated Electrochemical Apparatus:

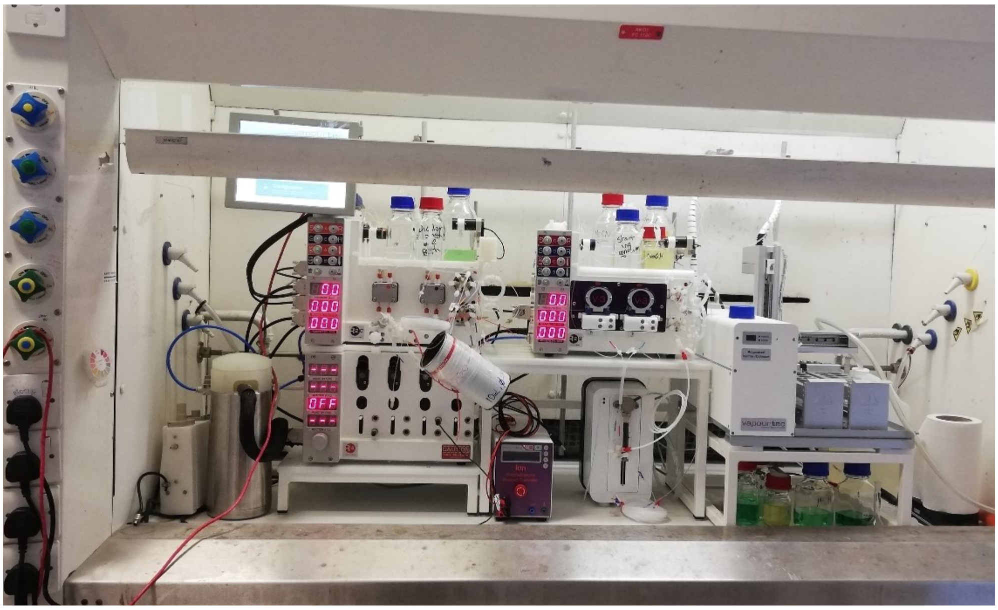

Figure S6: Picture of the automated electrolysis machine. The machine has 4 pumps (HPLC/peristaltic), an autosampler/fraction collector, galvanostat, backpressure regulators etc. that can all be remotely controlled via a PC.

Zaiput Liquid-Liquid Extractor:

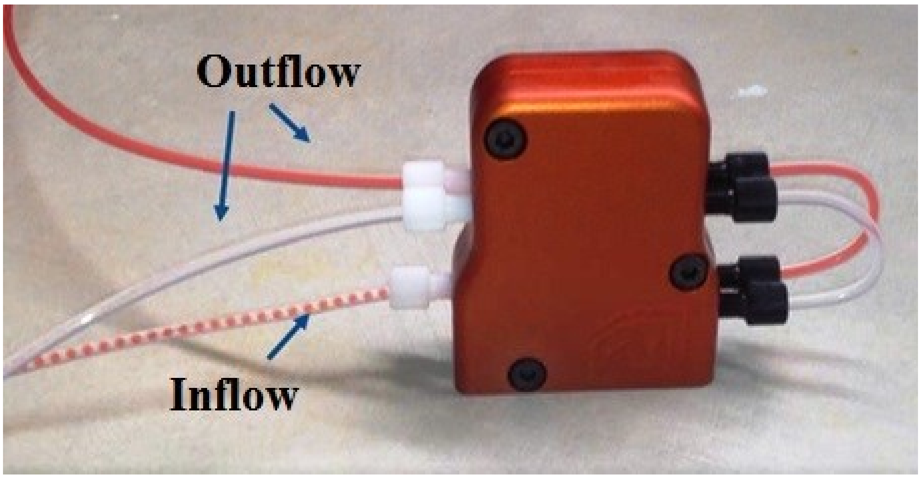

Electrolysis Optimisation Studies:

Optimisation Reaction Scheme:

Electrolysis optimisation studies were performed *via* the general procedure **D.**

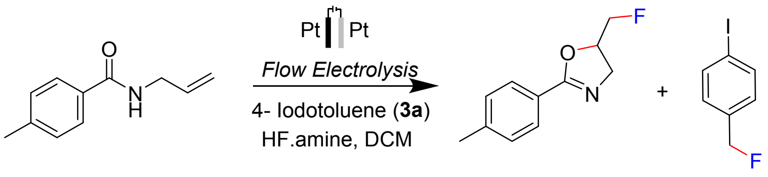

^{19}F NMR of the fluorinated oxazoline product and ethyl fluoroacetate

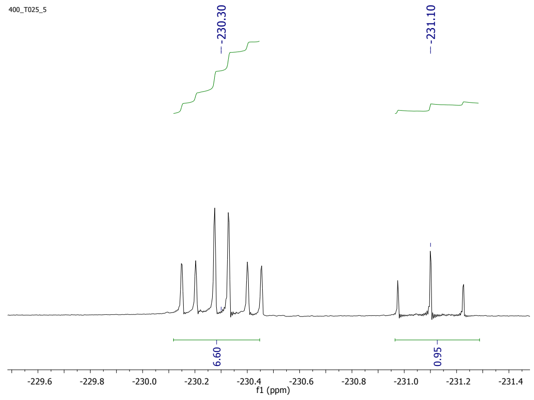

Figure S7: The alkyl range of the ^{19}F NMR spectrum of the electrolysis mixture after electrolysis; 5-(fluoromethyl)-2-(4-methylphenyl)-oxazoline (td, 1F, left) and ethyl fluoroacetate (t, 1F, right).

^{19}F NMR of the 1-(fluoromethyl)-4-iodobenzene and ethyl fluoroacetate

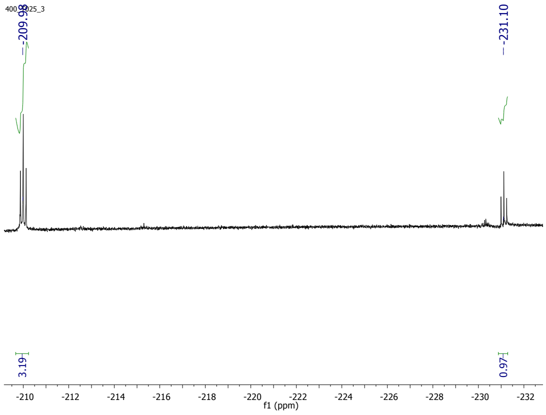

Figure S8: The alkyl range of the ^{19}F NMR spectrum of the electrolysis mixture after electrolysis; 1-(fluoromethyl)-4-iodobenzene (t, 1F, left) and ethyl fluoroacetate (t, 1F, right).

Table S1: Optimisation of Applied Charge

Entry	Charge	Yield of 2a /%	Yield of 3b /%
**1**	3	50	12%
**2**	3	51[b]	50
**3**	4	68	12%
**4**	5	76	12%
**5**	6	75	12%

Reaction conditions: 1 eq. of 4-iodotoluene, 1 eq. of *N*-allyl-4-methylbenzamide, solvent: CH_2Cl_2: 5.6HF•amine (prepared by diluting 9HF•Py with 3HF•NEt_3) (1:1 v/v), undivided cell, Pt(anode) and Pt(cathode), 0.1 mL/min, 12 h of stirring in batch post electrolysis. [b] 5HF•NEt_3used. Yield determined by ^{19}F NMR spectroscopy using ethyl fluoroacetate as an internal standard.

Table S2: Optimisation of HF.amine ratio

Entry	HF.amine	Yield 2a /%	Yield 3b /%
**1**	3	0	<5
**2**	4.5	<5	87
**3**	5	74	50
**4**	5.6	76	12
**5**	5.6[b]	0	<5
**6**	6	90	13
**7**	7	>95%	<5

Reaction conditions: 1 eq. of 4-iodotoluene, 1 eq. of *N*- allyl-4-methylbenzamide, solvent: CH_2Cl_2: xHF•amine (prepared by diluting 9HF•Py with 3HF•NEt_3) (1:1 v/v), 80 mA: 6.7 mAcm^{-2}: 5 F, undivided cell, Pt(anode) and Pt(cathode), 0.1 mL/min, 12 h of stirring in batch post electrolysis. [b] HF•amine stock solution prepared using 9HF•Py and diluting with pyridine. Yield determined by ^{19}F NMR spectroscopy using ethyl fluoroacetate (δ = −231.1, 1F) as an internal standard.

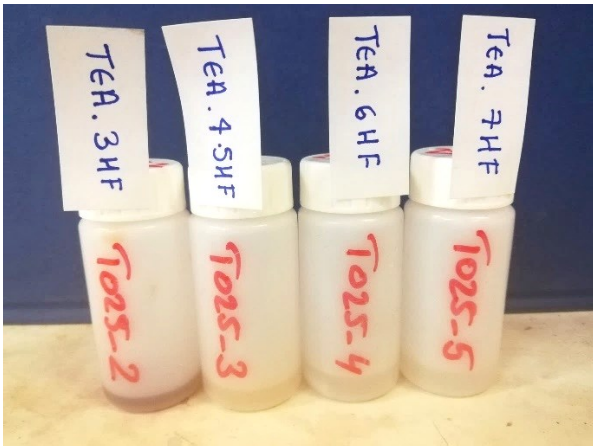

Figure S9: Image shows the colour of reaction solutions after electrolysis was performed in different HF.amine ratios. Electrolysis performed in 3HF•NEt_3. (far left) resulted in a dark purple solution and iodine precipitation. Electrolysis performed in HF•amine ratios of >5 resulted in a pale yellow solution, indicative of iodine(III) formation.

Table S3: Optimisation of Solvent Composition

Entry	Co-solvent	Yield of 2a /%	Yield of 3b /%	HF.amine ratio/ %
**1**	Dichloromethane	>95	<5	50
**2**	Dichloroethane	53	<5	50
**3**	Acetonitrile	18	0	50
**4**	Dichloromethane: Hexafluoro-isopropanol (3:1 v/v)	82	0	50
**5**	Dichloromethane	87	<5	40

Reaction conditions: 1 eq. of 4-iodotoluene, 1 eq. of *N*-allyl-4-methylbenzamide, solvent: Co-solvent: 7HF•amine (prepared by diluting 9HF•Py with $3HF•NEt_3$) (1:1 v/v), 5 F, undivided cell, Pt(anode) and Pt(cathode), 0.1 mL/min, 12 h of stirring in batch post electrolysis. Yield determined by ^{19}F NMR spectroscopy using ethyl fluoroacetate (δ = −231.1, 1F) as an internal standard.

Table S4: Optimisation of Anodic Material

Entry	Anodic Material	Yield of 2a [%]	Yield of 3b [%]
**1**	Glassy Carbon	0	0
**2**	Panasonic Carbon	0	0
**3**	Platinum	>95	<5%

Reaction conditions: 1 eq. of 4-iodotoluene, 1 eq. of *N*-allyl-4-methylbenzamide, solvent: CH_2Cl_2: 7HF•amine (prepared by diluting 9HF•Py with $3HF•NEt_3$) (1:1 v/v), 5 F, undivided cell, Pt(anode) and Pt(cathode), 0.1 mL/min, 12 h of stirring in batch post electrolysis. Yield determined by ^{19}F NMR spectroscopy using ethyl fluoroacetate (δ = −231.1, 1F) as an internal standard.

Table S5: Optimisation of Flow Rate

Entry	Flow Rate [mL min^{-1}]	Yield of 2a [%]	Yield of 3b [%]
**1**	0.05	82	<5
**2**	0.10	>95	<5
**3**	0.25	>95	<5
**4**	0.50	87	<5
**5**	0.75	77	<5
**6**	1.00	75	<5

Reaction conditions: 1 eq. of 4-iodotoluene, 1 eq. of *N*-allyl-4-methylbenzamide, solvent: CH_2Cl_2: 7HF•amine (prepared by diluting 9HF•Py with $3HF•NEt_3$) (1:1 v/v), 5 F, undivided cell, Pt(anode) and Pt(cathode), 12 h of stirring in batch post electrolysis. Yield determined by ^{19}F NMR spectroscopy using ethyl fluoroacetate (δ = −231.1, 1F) as an internal standard.

Table S6: Measuring the Yield with Time

Entry	Time [min]	Yield of 2a [%]	Voltage [V]
**1**	30	>95	5 – 6
**2**	63	>95	5 – 6
**3**	97	>95	5 – 6
**4**	120	>95	5 – 6
**5**	210	>95	5 – 6

Reaction conditions: 1 eq. of 4-iodotoluene, 1 eq. of *N*-allyl-4-methylbenzamide, solvent: CH_2Cl_2: 7HF•amine (prepared by diluting 9HF•Py with 3HF•NEt_3) (1:1 v/v), 5 F, undivided cell, Pt(anode) and Pt(cathode), 0.25 mL/min, 12 h of stirring in batch post electrolysis. Yield determined by ^{19}F NMR spectroscopy using ethyl fluoroacetate (δ = −231.1, 1F) as an internal standard.

Cyclic Voltammetry:

nHF•amine Anodic Potentials

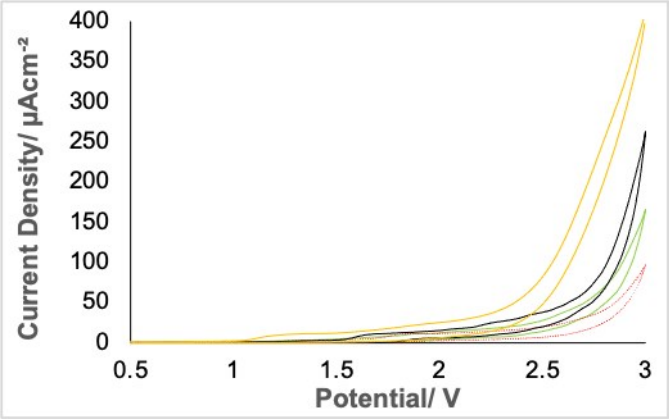

Figure S10: CV of 3HF•NEt_3 (yellow), 4.5HF•amine (black), 5.6HF•amine (green) and 7HF•amine (red). CV conditions: nHF•amine: CH_2Cl_2 (1:15 v/v), Pt disk (immersed surface area: 3 mm^2), Pt wire counter electrode, Ag/ 0.01 M AgCl reference, 10 mV/s.

nHF•amine Cathodic Potentials

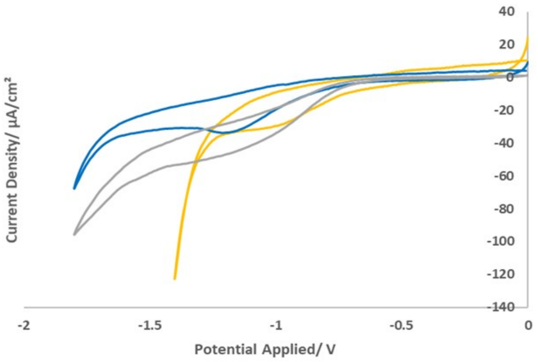

Figure S11: CV of 3HF•NEt_3 (blue), 5.6HF•amine (grey), 7HF•amine (orange). CV conditions are the same as the reaction conditions: nHF•amine: CH_2Cl_2 (1:1 v/v), GC carbon disk (immersed surface area: 3 mm^2), Pt wire counter electrode, Ag/ 0.01 M AgCl reference, 30 mV/s.

Substrate Potentials

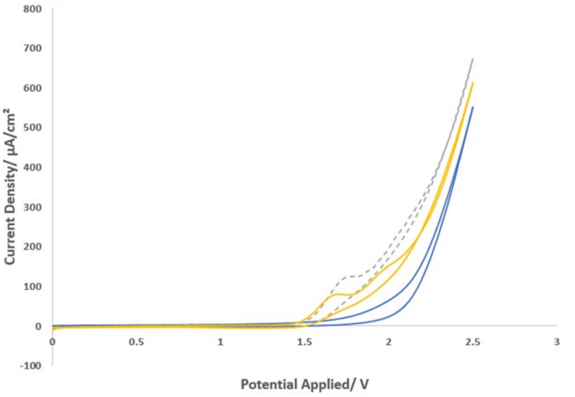

CV of *N*-allylpiperidine-1-carboxamide (5 mM, orange) and 4-iodotoluene (5 mM, grey) in 5.6HF•amine (blue). CV conditions are the same as the reaction conditions: nHF•amine: DCM (1:1 v/v), GC carbon disk (immersed surface area: 3 mm^2), Pt wire counter electrode, Ag/ 0.01 M AgCl reference, 30 mV/s.

Characterization of the Products:

(Difluoroiodo)toluene (4b)

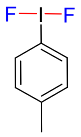

Obtained from 4-iodotoluene (170 mg, 0.78 mmol) using the general procedure **F**. The product was obtained as a colourless solid (164 mg, 0.64 mmol, 82%).

^{1}H NMR (500 MHz, CDCl$_3$): δ 7.84 (d, *J* = 8.5 Hz, 2H), 7.39 (d, *J* = 8.3 Hz, 2H), 2.47 (s, 3H).

^{19}F NMR (471 MHz, CDCl$_3$): δ -176.75 (s, 2F).

^{13}C NMR (126 MHz, CDCl$_3$): δ 142.5, 132.3, 130.4 (t, *J* = 3.3 Hz), 121.1 (t, *J* = 11.2 Hz), 21.3.

NMR data is identical to the literature data.[8]

Difluoro(*m*-tolyl)-λ^3-iodane (4f)

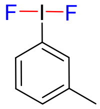

Obtained from 3-iodotoluene (181 mg, 0.83 mmol) using the general procedure **F**. The product was obtained as a yellow oil (151 mg, 0.59 mmol, 71%).

^{1}H NMR (300 MHz, CDCl$_3$) δ 7.82 – 7.70 (m, 2H), 7.46 (t, *J* = 7.8 Hz, 1H), 7.31 (d, *J* = 7.6 Hz, 1H), 2.43 (s, 3H).

^{19}F NMR (471 MHz, CDCl$_3$) δ -176.95 (s, 2F).

^{13}C NMR (126 MHz, CDCl$_3$) δ 142.1, 132.5, 131.2, 130.5 (t, *J* = 3.3 Hz), 127.3 (t, *J* = 3.5 Hz), 124.2 (t, *J* = 10.5 Hz), 21.6.

Difluoro(3-(trifluoromethyl)phenyl)-λ^3-iodane (4g)

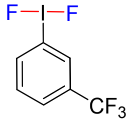

Obtained from 1-iodo-3-(trifluoromethyl)benzene (193 mg, 0.71 mmol) using the general procedure **F**. The product was obtained as a colourless solid (64 mg, 0.21 mmol, 29%).

^{1}H NMR (500 MHz, CDCl$_3$) δ 8.19 (s, 1H), 8.12 (d, *J* = 8.1 Hz, 1H), 7.79 (d, *J* = 7.7 Hz, 1H), 7.75 (t, *J* = 7.9 Hz, 1H).

^{19}F NMR (471 MHz, CDCl$_3$) δ -62.76 (s, 3F), -176.92 (s, 2F).

^{13}C NMR (126 MHz, CDCl$_3$) δ 134.2 (q, *J* = 33.6 Hz), 132.8 (t, *J* = 4.6 Hz), 132.0 (s), 128.5 (q, *J* = 3.4 Hz), 126.7 (q, *J* = 5.2 Hz), 123.1 (q, *J* = 273.2 Hz), 123.0 (t, *J* = 10.4 Hz).

5-(Fluoromethyl)-2-phenyl-oxazoline (2b)

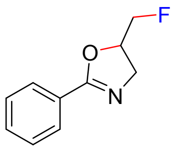

Prepared according to the general procedure **G** from *N*-allylbenzamide (81 mg, 0.5 mmol) over 15 h. The product was purified by column chromatography (SiO_2; cyclohexane: ethyl acetate 1:99) and obtained as a light yellow, clear oil (56 mg, 0.31 mmol, 63%).

Prepared according to the general procedure **I** using an 8.75 mL reactor coil, from *N*-allylbenzamide (242 mg, 1.5 mmol). The product was purified by column chromatography (SiO_2; cyclohexane: ethyl acetate 1:99) and obtained as a light yellow, clear oil (207 mg, 1.16 mmol, 77%).

^{1}H NMR (400 MHz, $CDCl_3$) δ 7.98 - 7.91 (m, 2H), 7.52 - 7.45 (m, 1H), 7.45 – 7.35 (m, 2H), 5.00 – 4.86 (m, 1H), 4.61 (ddd, *J* = 47.2, 10.4, 3.1 Hz, 1H), 4.47 (ddd, *J* = 47.3, 10.4, 5.7 Hz, 1H), 4.14 (dd, *J* = 14.9, 10.2 Hz, 1H), 3.86 (dd, *J* = 14.9, 7.5 Hz, 1H).

^{19}F NMR (376 MHz, $CDCl_3$) δ -230.49 (td, *J* = 47.2, 19.6 Hz, 1F).

^{13}C NMR (101 MHz, $CDCl_3$) δ 164.2, 131.7, 128.5, 128.4, 127.4, 83.5 (d, *J* = 175.2 Hz), 77.7 (d, *J* = 19.6 Hz), 55.9 (d, *J* = 5.9 Hz).

NMR data is identical to the literature data.[1]

5-(Fluoromethyl)-2-(4-methylphenyl)-oxazoline (2a)

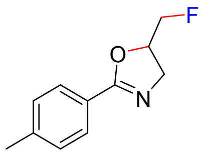

Prepared according to the general procedure **G** from *N*-allyl-4-methylbenzamide (88 mg, 0.5 mmol) over 15 h. The product was purified by column chromatography (SiO_2; cyclohexane: ethyl acetate 1:99) and obtained as a colourless oil (52 mg, 0.27 mmol, 54%).

Prepared according to the general procedure **I** using an 8.75 mL reactor coil (internal diameter = 1 mm), from *N*-allyl-4-methylbenzamide (263 mg, 1.5 mmol). The product was purified by column chromatography (SiO_2; cyclohexane: ethyl acetate 1:99) and obtained as a colourless oil (188 mg, 0.97 mmol, 65%).

^{1}H NMR (400 MHz, $CDCl_3$) δ 7.84 (d, *J* = 8.2 Hz, 2H), 7.22 (d, *J* = 7.9 Hz, 2H), 4.91 (ddddd, *J* = 19.1, 10.3, 7.6, 5.7, 3.1 Hz, 1H), 4.59 (ddd, *J* = 43.7, 10.4, 3.1 Hz, 1H), 4.47 (ddd, *J* = 47.3, 10.3, 5.6, 1H), 4.13 (ddd, *J* = 14.7, 10.2, 1.1 Hz, 1H), 3.84 (dd, *J* = 14.8, 7.5 Hz, 1H), 2.38 (s, 3H).

^{19}F NMR (376 MHz, $CDCl_3$) δ -228.67 (td, *J* = 47.2, 19.2 Hz, 1F).

^{13}C NMR (101 MHz, $CDCl_3$) δ 164.4, 142.1, 129.3, 128.4, 124.7, 83.6 (d, *J* = 175.1 Hz), 77.6 (d, *J* = 18.5 Hz), 55.8 (d, *J* = 5.89 Hz), 21.7.

NMR data is identical to the literature data.[1]

5-(Fluoromethyl)-2-(4-nitrophenyl)-oxazoline (2c)

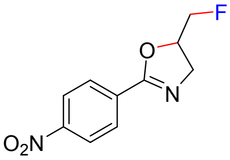

Prepared according to the general procedure **H** from *N*-allyl-4- nitrobenzamide (103 mg, 0.5 mmol) over 15 h. The product was purified by column chromatography (SiO_2; cyclohexane: ethyl acetate 7:3) and obtained as a colourless solid (28 mg, 0.12 mmol, 25%).

Prepared according to the general procedure **J** using a 17.5 mL reactor coil (internal diameter = 1 mm), from *N*-allyl-4- nitrobenzamide (309 mg, 1.5 mmol). The product was purified by column chromatography (SiO_2; cyclohexane: ethyl acetate 7:3) and obtained as a colourless solid (175 mg, 0.78 mmol, 52%).

^{1}H NMR (400 MHz, $CDCl_3$) δ 8.29 (d, *J* = 8.7 Hz, 2H), 8.18 (d, *J* = 8.7 Hz, 2H), 5.11-4.98 (m, 1H), 4.66 (ddd, *J* = 47.4, 10.6, 2.7 Hz, 1H), 4.58 (ddd, *J* = 47.1, 10.9, 5.3 Hz, 1H), 4.25 (dd, *J* = 14.8, 10.4 Hz, 1H), 3.99 (dd, *J* = 15.1, 7.6 Hz, 1H).

^{19}F NMR (376 MHz, $CDCl_3$) δ -230.67 (td, *J* = 47.7 Hz, 21.1 Hz, 1F).

^{13}C NMR (126 MHz, $CDCl_3$) δ 162.4, 149.7, 133.0, 129.4, 123.6, 83.1 (d, *J* = 175.9 Hz), 78.4 (d, *J* = 19.5 Hz), 55.9 (d, *J* = 6.1 Hz).

MP: 199 – 121 °C.

NMR data is identical to the literature data.[1]

5-(Fluoromethyl)-2-(4-(trifluoromethyl)phenyl)-oxazoline (2d)

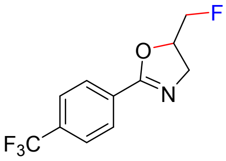

Prepared according to the general procedure **G** from *N*-allyl-4-(trifluoromethyl)benzamide (115 mg, 0.5 mmol) over 15 h. The product was purified by column chromatography (SiO_2; cyclohexane: ethyl acetate 1:3) and obtained as a colourless oil (93 mg, 0.38 mmol, 75%).

Prepared according to the general procedure **I** using an 8.75 mL reactor coil (internal diameter = 1 mm), from *N*-allyl-4-(trifluoromethyl)benzamide (344 mg, 1.5 mmol). The product was purified by column chromatography (SiO_2; cyclohexane: ethyl acetate 1:3) and obtained as a colourless oil (311 mg, 1.26 mmol, 84%).

^{1}H NMR (400 MHz, $CDCl_3$) δ 8.07 (d, *J* = 8.1 Hz, 2H), 7.67 (d, *J* = 8.2 Hz, 2H), 5.07 – 4.82 (m, 1H), 4.61 (ddd, *J* = 47.3, 10.7, 2.7 Hz, 1H), 4.48 (ddd, *J* = 47.3, 10.6, 5.3 Hz, 1H) 4.18 (ddd, *J* = 15.1, 10.3, 1.2 Hz, 1H), 3.91 (dd, *J* = 15.1, 7.6 Hz, 1H).

^{19}F NMR (376 MHz, $CDCl_3$) δ –63.01 (s, 3F), –229.81 (td, *J* = 47.2, 20.2 Hz, 1F).

^{13}C NMR (101 MHz, $CDCl_3$) δ 163.1, 133.3 (q, *J* = 32.6 Hz), 130.9, 128.8, 125.5 (q, *J* = 3.8 Hz), 112.5, 83.4 (d, *J* = 175.6 Hz), 78.2 (d, *J* = 19.5 Hz), 56.0 (d, *J* = 6.0 Hz).

NMR data is identical to the literature data.[1]

5-(Fluoromethyl)-2-(4-methoxyphenyl)-oxazoline (2e)

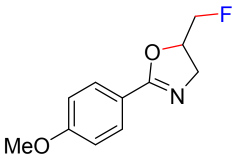

Prepared according to the general procedure **G** from *N*-allyl-4-methoxybenzamide (96 mg, 0.5 mmol) over 15 h. The product was purified by column chromatography SiO_2; (cyclohexane: ethyl acetate 1:3) and obtained as a yellow oil (44 mg, 0.21 mmol, 42%).

Prepared according to the general procedure **I** using an 8.75 mL reactor coil (internal diameter = 1 mm), from *N*-allyl-4-methoxybenzamide (287 mg, 1.5 mmol). The product was purified by column chromatography (SiO_2; cyclohexane: ethyl acetate 1:3) and obtained as a yellow oil (179 mg, 0.86 mmol, 57%).

^{1}H NMR (400 MHz, $CDCl_3$) δ 7.91 (d, *J* = 8.7 Hz, 2H), 6.92 (d, *J* = 8.7 Hz, 2H), 5.00 – 4.86 (m, 1H), 4.59 (ddd, *J* = 47.6, 10.3, 3.0 Hz, 1H), 4.46 (ddd, *J* = 47.6, 10.5, 5.8 Hz, 1H) 4.13 (dd, *J* = 14.5, 10.2 Hz, 1H), 3.86 – 3.78 (m, 4H).

^{19}F NMR (376 MHz, $CDCl_3$) δ -228.74 (td, *J* = 47.2, 19.3 Hz, 1F).

^{13}C NMR (101 MHz, $CDCl_3$) δ 164.3, 162.6, 130.4, 119.6, 114.0, 83.6 (d, *J* = 175.2 Hz), 77.8 (d, *J* = 19.5 Hz), 55.6, 55.5 (d, *J* = 6.1 Hz).

NMR data is identical to the literature data.[1]

5-(Fluoromethyl)-2-(thiophen-2-yl)-oxazoline (2f)

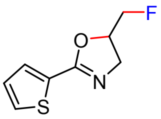

Prepared according to the general procedure **I** using an 8.75 mL reactor coil (internal diameter = 1 mm), from N-Allylthiophen-2-carboxamide (251 mg, 1.5 mmol). The product was purified by column chromatography (SiO_2; cyclohexane: ethyl acetate 1:3) and obtained as a colourless oil (100 mg, 0.54 mmol, 36%).

^{1}H NMR (500 MHz, $CDCl_3$) δ 7.62 (dd, *J* = 3.7, 1.2 Hz, 1H), 7.46 (dd, *J* = 5.0, 1.2 Hz, 1H), 7.07 (dd, *J* = 5.0, 3.7 Hz, 1H), 4.97 – 4.86 (m, 1H), 4.66 – 4.43 (m, 2H), 4.12 (ddd, *J* = 14.7, 10.1, 1.5 Hz, 1H), 3.85 (dd, *J* = 14.8, 7.5 Hz, 1H).

^{13}C NMR (126 MHz, $CDCl_3$) δ 160.45 (s), 130.77 (s), 130.13 (d, *J* = 39.3 Hz), 129.97 (s), 127.79 (s), 83.35 (d, *J* = 175.4 Hz), 78.28 (d, *J* = 19.6 Hz), 55.91 (s).

NMR data is identical to the literature data.[1]

5-(Fluoromethyl)-2-(furan-2-yl)-4,5-dihydrooxazole (2g)

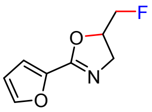

Prepared according to the general procedure **I** using an 8.75 mL reactor coil (internal diameter = 1 mm), from N-Allylfuran-2-carboxamide (227 mg, 1.5 mmol). The product was purified by column chromatography (SiO_2; cyclohexane: ethyl acetate 1:3) and obtained as a colourless oil (99 mg, 0.59 mmol, 39%).

^{1}H NMR (400 MHz, CDCl$_3$) δ 7.55 (dd, *J* = 1.7, 0.7 Hz, 1H), 6.99 (d, *J* = 3.4 Hz, 1H), 6.49 (dd, *J* = 3.5, 1.8 Hz, 1H), 5.00 – 4.81 (m, 1H), 4.68 – 4.38 (m, 2H), 4.15 (dd, *J* = 14.8, 10.1 Hz, 1H), 3.87 (dd, *J* = 14.9, 7.5 Hz, 1H).

^{19}F NMR (376 MHz, CDCl$_3$) δ -229.41 (td, *J* = 47.1, 19.7 Hz).

^{13}C NMR (101 MHz, CDCl$_3$) δ 156.53 (s), 145.61 (s), 142.71 (s), 114.94 (s), 111.84 (s), 83.24 (d, *J* = 175.6 Hz), 78.04 (d, *J* = 19.6 Hz), 55.79 (d, *J* = 5.9 Hz).

5-(Fluoromethyl)-2-(tetrahydrofuran-2-yl)-4,5-dihydrooxazole (2i)

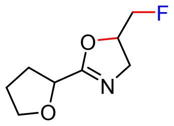

Prepared according to the general procedure **J** using a 60 mL reactor coil (internal diameter = 1 mm) from *N*-allyltetrahydrofuran-2-carboxamide (233 mg, 1.5 mmol). The yield was determined by ^{19}F NMR spectroscopy using ethyl fluoroacetate as an internal standard (62%)

Product could not be isolated due to the extremely volatile nature. A crude NMR is provided.

^{1}H NMR (400 MHz, CDCl$_3$) δ 4.69 (dddd, *J* = 20.7, 7.6, 5.0, 2.8 Hz, 1H), 4.56 – 4.22 (m, 3H), 3.98 – 3.72 (m, 3H), 3.72 – 3.55 (m, 1H), 2.16 – 1.66 (m, 4H).

^{19}F NMR (376 MHz, CDCl$_3$) δ -230.73 (tdd, *J* = 47.1, 37.5, 20.8 Hz).

^{13}C NMR (101 MHz, CDCl$_3$) δ 167.83 (s), 83.27 (d, *J* = 174.1 Hz), 73.55 (d, *J* = 15.1 Hz), 69.21 (d, *J* = 7.5 Hz), 55.24 (s), 30.00 (s), 25.66 (s).

HRMS (EI+): calc: $[M+H]^+$ ($C_8H_{13}NO_2F$) 174.0928; measured: 174.0930.

4-(5-(Fluoromethyl)-4,5-dihydrooxazol-2-yl)morpholine (2j)

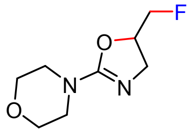

Prepared according to the general procedure **J** using a 60 mL reactor coil (internal diameter = 1 mm) from *N*-allylmorpholine-4-carboxamide (255 mg, 1.5 mmol). The yield was determined by ^{19}F NMR spectroscopy using ethyl fluoroacetate as an internal standard (67%)

Product could not be isolated due to the extremely volatile nature. A crude NMR is provided.

^{1}H NMR (400 MHz, CDCl$_3$) δ 4.86 – 4.74 (m, 1H), 4.58 – 4.36 (m, 2H), 3.90 (ddd, *J* = 12.6, 9.4, 1.6 Hz, 1H), 3.71 (dd, *J* = 6.1, 3.7 Hz, 4H), 3.57 (dd, *J* = 12.7, 7.1 Hz, 1H), 3.45 – 3.37 (m, 4H).

^{19}F NMR (376 MHz, CDCl$_3$) δ -227.09 (td, *J* = 47.4, 18.6 Hz).

^{13}C NMR (101 MHz, CDCl$_3$) δ 161.19 (s), 83.41 (d, *J* = 175.1 Hz), 66.49 (s), 66.29 (s), 55.92 (s), 45.04 (s).

HRMS (ES+): calc: $[M+H]^+$ ($C_8H_{14}N_2O_2F$) 189.1045; measured: 189.1039.

5-(Fluoromethyl)-2-(pyrrolidin-1-yl)-4,5-dihydrooxazole (2k)

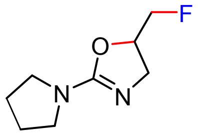

Prepared according to the general procedure **J** using a 60 mL reactor coil (internal diameter = 1 mm) from *N*-allylpyrrolidine-1-carboxamide (231 mg, 1.5 mmol). The yield was determined by ^{19}F NMR spectroscopy using ethyl fluoroacetate as an internal standard (63%).

Product could not be isolated due to the extremely volatile nature. A crude NMR is provided.

^{1}H NMR (400 MHz, $CDCl_3$) δ 4.81 – 4.62 (m, 1H), 4.49 – 4.27 (m, 2H), 3.87 – 3.74 (m, 1H), 3.46 (dd, *J* = 12.3, 7.1 Hz, 1H), 3.40 – 3.22 (m, 4H), 1.92 – 1.70 (m, 4H).

^{19}F NMR (376 MHz, $CDCl_3$) δ -226.46 (td, *J* = 47.5, 18.2 Hz).

^{13}C NMR (101 MHz, $CDCl_3$) δ 160.10 (s), 83.77 (d, *J* = 174.7 Hz), 78.36 (d, *J* = 18.0 Hz), 47.08 (s), 46.01 (s), 25.82 (s).

HRMS (EI+): calc: $[M]^+$ ($C_8H_{13}ON_2F$) 172.10064; measured: 172.1006.

5-(fluoromethyl)-2-(piperidin-1-yl)-4,5-dihydrooxazole (2ma)

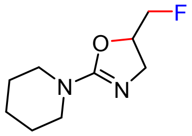

Prepared according to the general procedure **J** using a 60 mL reactor coil (internal diameter = 1 mm) from *N*-allylpiperidine-1-carboxamide (252 mg, 1.5 mmol). The yield was determined by ^{19}F NMR spectroscopy using ethyl fluoroacetate as an internal standard (64%)

Product could not be isolated due to the extremely volatile nature. A crude NMR is provided.

^{1}H NMR (400 MHz, $CDCl_3$) δ 4.70 (dddd, *J* = 9.5, 6.7, 3.2 Hz, 1H), 4.52 – 4.25 (m, 2H), 3.81 (ddd, *J* = 12.4, 9.4, 1.6 Hz, 1H), 3.46 (dd, *J* = 12.5, 7.1 Hz, 1H), 3.35 – 3.25 (m, 4H), 1.59 – 1.41 (m, 6H).

^{19}F NMR (376 MHz, $CDCl_3$) δ -226.45 (td, *J* = 47.4, 18.0 Hz).

^{13}C NMR (101 MHz, $CDCl_3$) δ 161.52 (s), 83.71 (d, *J* = 179.1 Hz), 77.98 (d, *J* = 18.8 Hz), 53.58 (s), 53.53 (s), 25.32 (s), 24.22 (s).

HRMS (EI+): calc: $[M]^+$ ($C_9H_{15}ON_2F$) 186.11629; measured: 186.1163.

Benzyl 3-(5-(fluoromethyl)-4,5-dihydrooxazol-2-yl)propanoate (2l)

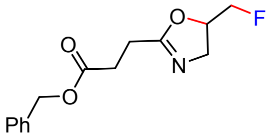

Prepared according to the general procedure **J** using a 60 mL reactor coil (internal diameter = 1 mm) from benzyl 4-(allylamino)-4-oxobutanoate (371 mg, 1.5 mmol) over 2 hr. The product was purified by column

chromatography (SiO_2; cyclohexane: ethyl acetate 1:2) and obtained as a colourless oil (215 mg, 0.81 mmol, 54%).

^{1}H NMR (400 MHz, CDCl$_3$) δ 7.47 – 7.28 (m, 5H), 5.12 (s, 2H), 4.69 (ddddd, *J* = 19.9, 10.3, 7.5, 5.5, 3.0 Hz, 1H), 4.39 (ddd, *J* = 47.2, 10.4, 3.0 Hz, 1H), 4.29 (ddd, *J* = 47.2, 10.3, 5.1 Hz, 1H), 3.87 (dd, *J* = 14.7, 10.2 Hz, 1H), 3.59 (ddd, *J* = 14.7, 7.3, 1.5 Hz, 1H), 2.77 – 2.68 (m, 2H), 2.67 – 2.59 (m, 2H).

^{19}F NMR (376 MHz, CDCl$_3$) δ -229.41 (td, *J* = 47.2, 19.8 Hz).

^{13}C NMR (101 MHz, CDCl$_3$) δ 171.95 (s), 166.43 (s), 135.79 (d, *J* = 16.1 Hz), 128.40 (s), 83.40 (d, *J* = 174.9 Hz), 77.57 (s), 66.48 (s), 55.22 (s), 30.27 (s), 23.16 (s).

NMR data is identical to the literature data.[21]

_N_-allyl-2-fluoropiperidine-1-carboxamide (2mb)

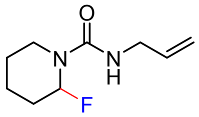

^{19}F NMR (471 MHz, CDCl$_3$) δ -124.02 (ddd, *J* = 283.9, 55.8, 3.1 Hz).

HRMS (ES+): calc: $[M+H]^+$ ($C_9H_{16}ON_2F$) 187.1247; measured: 187.1256.

1,1-Difluoro-1,2-diphenylethane (5a)

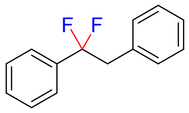

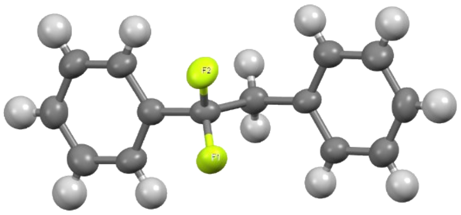

Prepared according to the general procedure **H** from 1,1-diphenylethene (90 mg, 0.5 mmol) over 2 hr. The product was purified by column chromatography (SiO_2; petroleum ether 100%) and obtained as a white crystalline solid (102 mg, 0.47 mmol, 94%).

Prepared according to the general procedure **J** using a 5 mL reactor coil (internal diameter = 1 mm), from 1,1-diphenylethene (270 mg, 1.5 mmol). The product was purified by column chromatography (SiO_2; petroleum ether 100%) and obtained as a white crystalline solid (309 mg, 1.42 mmol, 94%).

Prepared according to the general procedure **K** using a 20 mL reactor coil (internal diameter = 1 mm), from 1,1-diphenylethene (1.14 g, 6.3 mmol). The product was purified by column chromatography (SiO_2; petroleum ether 100%) and obtained as a white crystalline solid (1.25 g, 5.72 mmol, 91%).

^{1}H NMR (500 MHz, CDCl$_3$) δ 7.46 – 6.96 (m, 10H), 3.41 (t, *J* = 15.8 Hz, 2H).

^{19}F NMR (471 MHz, CDCl$_3$) δ -94.91 (t, *J* = 15.9 Hz, 2F).

^{13}C NMR (126 MHz, CDCl$_3$) δ 137.0 (t, *J* = 27.0 Hz), 132.8, 130.8, 129.8, 128.3 (d, *J* = 2.7 Hz), 127.4, 125.4 (t, *J* = 6.2 Hz), 122.1 (t, *J* = 244.1 Hz), 46.0 (t, *J* = 28.6 Hz).

NMR data is identical to the literature data.[13]

1-((2,3-Difluoropropoxy)methyl)-4-fluorobenzene (6a)

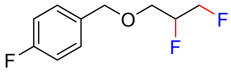

Prepared according to the general procedure **H** from 1-((allyloxy)methyl)-4-fluorobenzene (83 mg, 0.5 mmol) over 15 hr. The product was purified by column chromatography (SiO_2; cyclohexane: diethyl ether 9:1) and obtained as a volatile, colourless oil (62 mg, 0.30 mmol, 61%).

Prepared according to the general procedure **J** using a 17.5 mL reactor coil (internal diameter = 1 mm), from 1-((allyloxy)methyl)-4-fluorobenzene (249 mg, 1.5 mmol). The product was purified by column chromatography (SiO_2; cyclohexane: diethyl ether 9:1) and obtained as a volatile, colourless oil (211 mg, 1.03 mmol, 69%).

^{1}H NMR (400 MHz, $CDCl_3$) δ 7.34 – 7.27 (m, 2H), 7.08 – 7.01 (m, 2H), 4.94 – 4.72 (m, 1H), 4.72 – 4.52 (m, 2H), 4.54 (s, 2H), 3.71 (ddd, *J* = 20.2, 4.9, 1.3 Hz, 2H).

^{19}F NMR (376 MHz, $CDCl_3$) δ -114.21 – -114.33 (m, 1F), -195.63 – -196.54 (m, 1F), -233.69 (tdd, *J* = 47.2, 21.1, 13.2 Hz, 1F).

^{13}C NMR (101 MHz, $CDCl_3$) δ 162.7 (d, *J* = 246.0 Hz), 133.4 (d, *J* = 3.1 Hz), 129.7 (d, *J* = 8.2 Hz), 115.6 (d, *J* = 21.5 Hz), 90.5 (dd, *J* = 175.7, 19.9 Hz), 82.2 (dd, *J* = 172.4, 23.4 Hz), 73.2, 68.0 (dd, *J* = 24.2, 7.9 Hz).

NMR data is identical to the literature data.[2]

1-((2,3-Difluoropropoxy)methyl)-3,5-dinitrobenzene (6b)

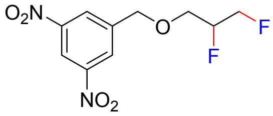

Prepared according to the general procedure **H** from 1-((allyloxy)methyl)-3,5-dinitrobenzene (83 mg, 0.5 mmol) over 15 hr. The product was purified by column chromatography (SiO_2; cyclohexane: ethyl acetate 7:3) and obtained as a dark orange oil (88 mg, 0.32 mmol, 64%).

Prepared according to the general procedure **J** using a 17.5 mL reactor coil (internal diameter = 1 mm), from 1-((allyloxy)methyl)-3,5-dinitrobenzene (237 mg, 1.5 mmol). The product was purified by column chromatography (SiO_2; cyclohexane: ethyl acetate 7:3) and obtained as a dark orange oil (319 mg, 1.15 mmol, 77%).

^{1}H NMR (500 MHz, $CDCl_3$) δ 8.98 (t, *J* = 2.1 Hz, 1H), 8.53 (dt, *J* = 2.0, 0.7 Hz, 2H), 5.06 – 4.79 (m, 1H), 4.81 (s, 2H), 4.77 – 4.50 (m, 2H), 3.94 – 3.91 (m, 1H), 3.89 – 3.87 (m, 1H).

^{19}F NMR (376 MHz, $CDCl_3$) δ -195.76 – -196.21 (m, 1F), -234.35 (tdd, *J* = 47.1, 21.5, 13.4 Hz, 1F).

^{13}C NMR (126 MHz, $CDCl_3$) δ 148.8, 142.6, 127.2, 118.3, 90.3 (dd, *J* = 176.5, 20.2 Hz), 81.8 (dd, *J* = 173.1, 23.9 Hz), 71.6, 69.4 (dd, *J* = 23.7, 7.6 Hz).

NMR data is identical to the literature data.[2]

1-((2,3-Difluoropropoxy)methyl)-2-methylbenzene (6c)

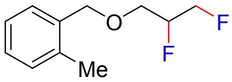

Prepared according to the general procedure **H** from 1-((allyloxy)methyl)-2-methylbenzene (81 mg, 0.5 mmol) over 15 hr. The product was purified by column chromatography (SiO_2; cyclohexane: diethyl ether 9.5:0.5) and obtained as a volatile colourless oil (72 mg, 0.36 mmol, 72%).

Prepared according to the general procedure **J** using a 17.5 mL reactor coil (internal diameter = 1 mm), from 1-((allyloxy)methyl)-2-methylbenzene (243 mg, 1.5 mmol). The product was purified by column chromatography (SiO_2; cyclohexane: diethyl ether 9.5:0.5) and obtained as a volatile colourless oil (222 mg, 1.11 mmol, 74%).

^{1}H NMR (500 MHz, CDCl$_3$) δ 7.35 – 7.27 (m, 1H), 7.25 – 7.14 (m, 3H), 4.93 – 4.74 (m, 1H), 4.72 – 4.50 (m, 4H), 3.73 (ddd, *J* = 19.8, 5.0, 1.3 Hz, 2H), 2.34 (s, 3H).

^{19}F NMR (376 MHz, CDCl$_3$) δ -195.78 – -196.26 (m, 1F), -233.70 (tdd, *J* = 46.7, 19.9, 12.5 Hz, 1F).

^{13}C NMR (126 MHz, CDCl$_3$) δ 137.0, 135.5, 130.6, 128.9, 128.4, 126.0, 90.5 (dd, *J* = 175.6, 19.7 Hz), 82.4 (dd, *J* = 172.3, 23.2 Hz), 72.4, 68.0 (dd, *J* = 24.4, 8.0 Hz), 18.9.

NMR data is identical to the literature data.[2]

1-Bromo-2-((2,3-difluoropropoxy)methyl)benzene (6d)

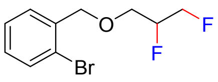

Prepared according to the general procedure **H** from 1-((allyloxy)methyl)-2-bromobenzene (113 mg, 0.5 mmol) over 15 hr. The product was purified by column chromatography (SiO_2; cyclohexane: ethyl acetate 97.5: 2.5) and obtained as a colourless oil (59 mg, 0.22 mmol, 45%).

Prepared according to the general procedure **J** using a 17.5 mL reactor coil (internal diameter = 1 mm), from 1-((allyloxy)methyl)-2-bromobenzene (340 mg, 1.5 mmol). The product was purified by column chromatography (SiO_2; cyclohexane: ethyl acetate 97.5: 2.5) and obtained as a colourless oil (183 mg, 0.69 mmol, 46%).

^{1}H NMR (400 MHz, CDCl$_3$) δ 7.54 (dd, *J* = 8.0, 1.0 Hz, 1H), 7.43 (dd, *J* = 8.0, 1.0 Hz, 1H), 7.32 (td, *J* = 7.6, 1.1 Hz, 1H), 7.17 (t, *J* = 7.7 Hz, 1H), 4.98 – 4.79 (m, 1H), 4.76 – 4.58 (m, 4H), 3.81 (ddd, *J* = 19.8, 4.9, 1.2 Hz, 2H).

^{19}F NMR (376 MHz, CDCl$_3$) δ -195.96 – -196.49 (m, 1F), -233.68 (tdd, *J* = 47.2, 20.9, 13.2 Hz, 1F).

^{13}C NMR (101 MHz, CDCl$_3$) δ 137.0, 132.9, 129.5, 129.3, 127.7, 123.0, 90.4 (dd, *J* = 177.5, 18.2 Hz), 82.3 (dd, *J* = 172.4, 23.3 Hz), 73.2, 68.6 (dd, *J* = 24.4, 8.0 Hz).

NMR data is identical to the literature data.[2]

2-((2,3-Difluoropropoxy)methyl)pyridine (6e)

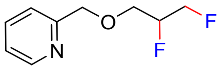

Prepared according to the general procedure **H** from 2-((allyloxy)methyl)pyridine (74 mg, 0.5 mmol) over 15 hr. The product was purified by column chromatography (SiO_2; cyclohexane: ethyl acetate 6:4) and obtained as an orange oil (44 mg, 0.24 mmol, 47%).

Prepared according to the general procedure **J** using a 17.5 mL reactor coil (internal diameter = 1 mm), from 2-((allyloxy)methyl)pyridine (224 mg, 1.5 mmol). The product was purified by column chromatography (SiO_2; cyclohexane: ethyl acetate 6:4) and obtained as an orange oil (124 mg, 0.66 mmol, 44%).

^{1}**H NMR (400 MHz, $CDCl_3$)** δ 8.54 (d, *J* = 4.2, 1H), 7.70 (td, *J* = 7.7, 1.7 Hz, 1H), 7.41 (d, *J* = 7.8 Hz, 1H), 7.23 – 7.18 (m, 1H), 4.99 – 4.76 (m, 1H), 4.76 – 4.54 (m, 4H), 3.82 (ddd, *J* = 20.4, 4.9, 1.2 Hz, 2H).

^{19}**F NMR (376 MHz, $CDCl_3$)** δ = -195.94 - -196.34 (m, 1F), -233.58 (tdd, J = 46.7, 21.4, 12.3 Hz, 1F).

^{13}**C NMR (101 MHz, $CDCl_3$)** δ 157.8, 149.4, 137.0, 122.8, 121.6, 90.4 (dd, *J* = 175.8, 19.9 Hz), 82.2 (dd, *J* = 172.4, 23.4 Hz), 74.6, 68.8 (dd, *J* = 24.1, 7.9 Hz).

NMR data is identical to the literature data.[2]

((2,3-Difluoro-2-methylpropoxy)methyl)benzene (6f)

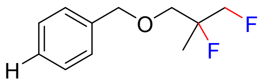

Prepared according to the general procedure **H** from ((2-methylallyloxy)methyl)benzene (81 mg, 0.5 mmol) over 15 hr. The product was purified by column chromatography (SiO_2; cyclohexane: ethyl acetate 9:1) and obtained as a volatile colourless oil (73 mg, 0.36 mmol, 73%).

Prepared according to the general procedure **J** using a 17.5 mL reactor coil (internal diameter = 1 mm), from ((2-methylallyloxy)methyl)benzene (243 mg, 1.5 mmol). The product was purified by column chromatography (SiO_2; cyclohexane: ethyl acetate 9:1) and obtained as a volatile colourless oil (237 mg, 1.18 mmol, 79%).

^{1}**H NMR (400 MHz, $CDCl_3$)** δ 7.38 – 7.28 (m, 5H), 4.60 (d, *J* = 3.6 Hz, 2H), 4.49 (ddd, *J* = 23.2, 19.3, 4.4 Hz, 2H), 3.60 (dddd, *J* = 39.8, 17.0, 10.3, 2.0 Hz, 2H), 1.40 (dd, *J* = 21.9, 2.2 Hz, 2H).

^{19}**F NMR (376 MHz, $CDCl_3$)** δ -157.87 – -166.23 (m, 1F), -232.54 (td, *J* = 48.2, 12.55 Hz, 1F).

^{13}**C NMR (101 MHz, $CDCl_3$)** δ 137.9, 128.6, 128.0, 127.8, 94.7 (dd, *J* = 172.7, 18.3 Hz), 84.6 (dd, *J* = 175.7, 27.0 Hz), 73.9, 71.6 (dd, *J* = 27.4, 4.9 Hz), 18.6 (dd, *J* = 23.2, 5.0 Hz).

NMR data is identical to the literature data.[2]

1-((2,3-Difluoro-2-methylpropoxy)methyl)-4-(trifluoromethyl)benzene (6g)

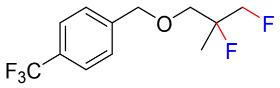

Prepared according to the general procedure **H** from 1-(((2-methylallyl)oxy)methyl)-4-(trifluoromethyl)benzene (115 mg, 0.5 mmol) over 15 hr. The product was purified by column chromatography (SiO_2; cyclohexane: diethyl ether 9:1) and obtained as a volatile colourless oil (103 mg, 0.38 mmol, 77%).

Prepared according to the general procedure **J** using a 17.5 mL reactor coil (internal diameter = 1 mm), from 1-(((2-methylallyl)oxy)methyl)-4-(trifluoromethyl)benzene (245 mg, 1.5 mmol). The product was purified by column chromatography (SiO_2; cyclohexane: diethyl ether 9:1) and obtained as a volatile colourless oil (326 mg, 1.22 mmol, 81%).

^{1}**H NMR (400 MHz, $CDCl_3$)** δ 7.62 (d, *J* = 8.1 Hz, 2H), 7.44 (d, *J* = 8.0 Hz, 2H), 4.64 (s, *J* = 6.5 Hz, 2H), 4.49 (dd, *J* = 47.3, 18.5 Hz, 2H), 3.71 – 3.46 (m, 2H), 1.41 (dd, *J* = 21.8, 2.2 Hz, 3H).

^{19}**F NMR (376 MHz, $CDCl_3$)** δ -62.54 (s, 3F), -162.30 – -162.76 (m, 1F), -232.32 (td, *J* = 47.0, 12.6 Hz, 1F).

^{13}**C NMR (101 MHz, $CDCl_3$)** δ 142.1, 130.2 (d, *J* = 32.4 Hz), 127.6, 125.6, 123.0, 94.6 (dd, *J* = 173.0, 18.4 Hz), 84.4 (dd, *J* = 175.9, 27.5 Hz), 73.0, 72.0 (dd, *J* = 27.2, 4.8 Hz), 18.7 (dd, *J* = 23.2, 4.8 Hz).

NMR data is identical to the literature data.[2]

2-Fluoro-1,3-diphenylpropane-1,3-dione (7a)

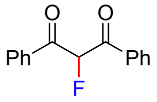

Prepared according to the general procedure **H** from 1,3-diphenylpropane-1,3-dione (112 mg, 0.5 mmol) over 2 hr. The product was purified by column chromatography (SiO_2; cyclohexane: DCM 9:1) and obtained as a pale yellow solid (87 mg, 0.36 mmol, 72%).

Prepared according to the general procedure **J** using a 5 mL reactor coil (internal diameter = 1 mm), from 1,3-diphenylpropane-1,3-dione (336 mg, 1.5 mmol). The product was purified by column chromatography (SiO_2; cyclohexane: DCM 9:1) and obtained as a pale yellow solid (287 mg, 1.18 mmol, 79%).

Prepared according to the general procedure **K** using a 20 mL reactor coil (internal diameter = 1 mm), from 1,3-diphenylpropane-1,3-dione (1.41 g, 6.3 mmol). The product was purified by column chromatography (SiO_2; cyclohexane:CH_2Cl_2 9:1) and obtained as a pale yellow solid (1.24 mg, 5.12 mmol, 81%).

^{1}H NMR (400 MHz, $CDCl_3$) δ 8.10 (d, *J* = 7.7 Hz, 4H), 7.62 (t, *J* = 7.4 Hz, 2H), 7.49 (t, *J* = 7.7 Hz, 4H), 6.54 (d, *J* = 49.2 Hz, 1H).

^{19}F NMR (376 MHz, $CDCl_3$) δ -186.84 (d, *J* = 49.1 Hz, 1F).

^{13}C NMR (101 MHz, $CDCl_3$) δ 191.4 (d, *J* = 20.2 Hz), 134.7, 133.7 (d, *J* = 2.0 Hz), 130.0 (d, *J* = 3.5 Hz), 129.0, 96.8 (d, *J* = 206.4 Hz).

MP: 66 – 68 ^{o}C.

NMR data is identical to the literature data.[9]

Ethyl 2-fluoro-3-oxo-3-phenylpropionate (7b)

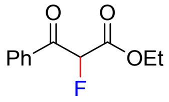

Prepared according to the general procedure **H** from ethyl 3-oxo-3-phenylpropanoate (96 mg, 0.5 mmol) over 2 hr. The product was purified by column chromatography (SiO_2; cyclohexane: diethyl ether 9:1) and obtained as a very pale yellow oil (90 mg, 0.43 mmol, 86%).

Prepared according to the general procedure **J** using a 5 mL reactor coil (internal diameter = 1 mm), from ethyl 3-oxo-3-phenylpropanoate (288 mg, 1.5 mmol). The product was purified by column chromatography (SiO_2; cyclohexane: diethyl ether 9:1) and obtained as a very pale yellow oil (294 mg, 1.40 mmol, 93%).

Prepared according to the general procedure **K** using a 20 mL reactor coil (internal diameter = 1 mm), from ethyl 3-oxo-3-phenylpropanoate (1.21 g, 6.3 mmol). The product was purified by column chromatography (SiO_2; cyclohexane: diethyl ether 9:1) and obtained as a very pale yellow oil (1.22 g, 5.8 mmol, 92%).

^{1}H NMR (500 MHz, $CDCl_3$): δ 8.06 – 8.02 (m, 2H), 7.67 – 7.60 (m, 1H), 7.56 – 7.46 (m, 2H), 5.86 (d, *J* = 48.8 Hz, 1H), 4.33 – 4.26 (m, 2H), 1.26 (t, *J* = 7.1 Hz, 3H).

^{19}F NMR (471 Hz, $CDCl_3$): δ -190.3 (d, J = 49.1 Hz, 1F).

^{13}C NMR (126 MHz, CDCl$_3$): δ 189.7 (d, *J* = 20.2 Hz), 165.1 (d, *J* = 24.1 Hz), 134.7, 133.5 (d, *J* = 2.1 Hz), 129.7 (d, *J* = 3.4 Hz), 129.0, 90.3 (d, *J* = 197.6 Hz), 62.9, 14.1.

NMR data is identical to the literature data.[9]

Ethyl 2-fluoro-1-oxo-2,3-dihydro-1H-indene-2-carboxylate (7c)

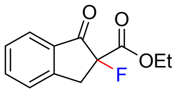

Prepared according to the general procedure **H** from ethyl 1-oxo-2,3-dihydro-1H-indene-2-carboxylate (102 mg, 0.5 mmol) over 2 hr. The product was purified by column chromatography (SiO_2; cyclohexane: ethyl acetate 9:1) and obtained as a colourless solid (83 mg, 0.37 mmol, 75%).

Prepared according to the general procedure **J** using a 5 mL reactor coil (internal diameter = 1 mm), from ethyl 1-oxo-2,3-dihydro-1H-indene-2-carboxylate (306 mg, 0.5 mmol). The product was purified by column chromatography (SiO_2; cyclohexane: ethyl acetate 9:1) and obtained as a colourless solid (290 mg, 1.31 mmol, 87%).

^{1}H NMR (400 MHz, CDCl$_3$) δ 7.85 (d, *J* = 7.7 Hz, 1H), 7.71 (t, *J* = 7.5 Hz, 1H), 7.54 – 7.44 (m, 2H), 4.29 (q, *J* = 7.1 Hz, 2H), 3.79 (dd, *J* = 17.6, 11.6 Hz, 1H), 3.44 (dd, *J* = 23.3, 17.6 Hz, 1H), 1.27 (t, *J* = 7.1 Hz, 3H).

^{19}F NMR (376 MHz, CDCl$_3$) δ -164.44 (dd, *J* = 23.2, 11.5 Hz, 1F).

^{13}C NMR (126 MHz, CDCl$_3$) δ 195.5 (d, *J* = 18.2 Hz), 167.6 (d, *J* = 27.9 Hz), 151.2 (d, J = 3.5 Hz), 136.9, 133.5, 128.8, 126.8, 125.9, 94.5 (d, J = 201.6 Hz), 62.8, 38.5 (d, *J* = 23.9 Hz), 14.2.

MP: 27 – 28 ^{o}C.

NMR data is identical to the literature data.[10]

Methyl 2-fluoro-1-oxo-2,3-dihydro-1H-indene-2-carboxylate (7d)

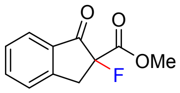

Prepared according to the general procedure **H** from methyl 1-oxo-2,3-dihydro-1H-indene-2-carboxylate (95 mg, 0.5 mmol) over 2 hr. The product was purified by column chromatography (SiO_2; cyclohexane: ethyl acetate 9:1) and obtained as a very pale yellow oil (80 mg, 0.38 mmol, 77%).

Prepared according to the general procedure **J** using a 5 mL reactor coil (internal diameter = 1 mm), from methyl 1-oxo-2,3-dihydro-1H-indene-2-carboxylate (285 mg, 1.5 mmol). The product was purified by column chromatography (SiO_2; cyclohexane: ethyl acetate 9:1) and obtained as a very pale yellow oil (269 mg, 1.29 mmol, 86%).

^{1}H NMR (400 MHz, CDCl$_3$) δ 7.85 (d, *J* = 7.7 Hz, 1H), 7.71 (td, *J* = 7.6, 1.2 Hz, 1H), 7.52 – 7.45 (m, 2H), 3.81 (s, 3H), 3.84-3.77 (m, 1H), 3.45 (dd, *J* = 23.3, 17.7 Hz, 1H).

^{19}F NMR (376 MHz, CDCl$_3$) δ -164.54 (dd, *J* = 23.4, 11.2 Hz, 1F).

^{13}C NMR (101 MHz, CDCl$_3$) δ 195.3 (d, *J* = 18.2 Hz), 167.9 (d, *J* = 27.9 Hz), 150.8 (d, *J* = 4.1 Hz), 137.0, 133.4, 128.9, 126.8 (d, *J* = 1.7 Hz), 125.9, 94.9 (d, *J* = 201 Hz), 53.5, 38.5 (d, *J* = 23.9 Hz).

NMR data is identical to the literature data.[11]

2-Fluoro-1-phenyl-1-propanone (8a)

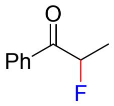

Both batch and flow procedures were carried out using DCE instead of DCM and a substrate concentration of 0.05 M, to account for the reduced yield in the electrolysis step.

Prepared according to the general procedure **H** from propiophenone (67 mg, 0.5 mmol) over 24 hr at 55 ^oC. The product was purified by column chromatography (SiO_2; cyclohexane: ethyl acetate 98:2) and obtained as a colourless oil (55 mg, 0.36 mmol, 72%).

Prepared according to the general procedure **J** using a 30 mL reactor coil (internal diameter = 1 mm, heated to 55 ^oC), from propiophenone (201 mg, 1.5 mmol). The product was purified by column chromatography (SiO_2; cyclohexane: ethyl acetate 98:2) and obtained as a colourless oil (148 mg, 0.97 mmol, 65%).

^{1}H NMR (500 MHz, $CDCl_3$) δ 8.01 – 7.96 (m, 2H), 7.64 – 7.58 (m, 1H), 7.52 – 7.47 (m, 2H), 5.71 (dq, *J* = 48.6, 6.8 Hz, 1H), 1.67 (dd, *J* = 24.0, 6.8 Hz, 3H).

^{19}F NMR (471 MHz, $CDCl_3$) δ -181.43 (dq, *J* = 48.4, 24.2 Hz, 1F).

^{13}C NMR (126 MHz, $CDCl_3$) δ 197.1 (d, *J* = 19.5 Hz), 134.2, 134.0, 129.2 (d, *J* = 3.6 Hz), 128.9, 90.5 (d, *J* = 180.1 Hz), 18.6 (d, *J* = 22.8 Hz).

NMR data is identical to the literature data.[12]

2-Fluoro-1-(1-naphthyl)ethenone (8b)

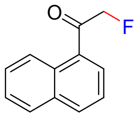

Both batch and flow procedures were carried out using DCE instead of DCM and a substrate concentration of 0.05 M, to account for the reduced yield in the electrolysis step.

Prepared according to the general procedure **H** from 1-(naphthalen-1-yl)ethan-1-one (85 mg, 0.5 mmol) over 24 hr at 55 ^oC. The product was purified by column chromatography (SiO_2; cyclohexane: ethyl acetate 98:2) and obtained as a pale yellow oil (86 mg, 0.46 mmol, 91%).

Prepared according to the general procedure **J** using a 30 mL reactor coil (internal diameter = 1 mm, heated to 55 ^oC), from 1-(naphthalen-1-yl)ethan-1-one (255 mg, 1.5 mmol). The product was purified by column chromatography (SiO_2; cyclohexane: ethyl acetate 98:2) and obtained as a pale yellow oil (234 mg, 1.24 mmol, 83%).

^{1}H NMR (500 MHz, $CDCl_3$) δ 8.70 (d, *J* = 8.6 Hz, 1H), 8.07 (d, *J* = 8.2 Hz, 1H), 7.90 (d, *J* = 9.5 Hz, 1H), 7.81 (d, *J* = 9.3 Hz, 1H), 7.61 (dt, *J* = 13.8, 8.3 Hz, 2H), 7.56 – 7.50 (m, 1H), 5.52 (d, *J* = 47.2 Hz, 2H).

^{19}F NMR (376 MHz, $CDCl_3$) δ –224.91 (t, *J* = 47.2 Hz, 1F).

^{13}C NMR (126 MHz, $CDCl_3$) δ 197.1 (d, *J* = 16.4 Hz), 134.2, 131.4, 131.4, 130.5, 128.8, 128.3, 128.3, 127.1, 125.6, 124.4, 84.1 (d, *J* = 185.0 Hz).

NMR data is identical to the literature data.[12]

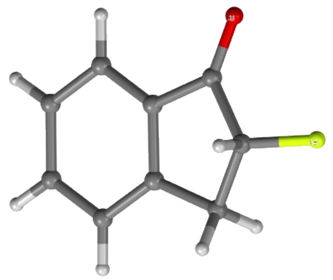

2-Fluoro-1-indanone (8c)

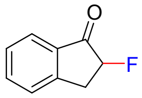

Both batch and flow procedures were carried out using DCE instead of CH_2Cl_2 and a substrate concentration of 0.05 M, to account for the reduced yield in the electrolysis step.

Prepared according to the general procedure **H** from 1-indanone (66 mg, 0.5 mmol) over 24 hr at 55 ^{o}C. The product was purified by column chromatography (SiO_2; cyclohexane:CH_2Cl_2 6:4) and obtained as a white crystals (53 mg, 0.35 mmol, 71%).

Prepared according to the general procedure **J** using a 30 mL reactor coil (internal diameter = 1 mm, heated to 55 ^{o}C), from 1-indanone (198 mg, 1.5 mmol). The product was purified by column chromatography (SiO_2; cyclohexane:CH_2Cl_2 6:4) and obtained as a white crystals (137 mg, 0.91 mmol, 61%).

^{1}H NMR (500 MHz, $CDCl_3$) δ 7.78 (d, *J* = 7.7 Hz, 1H), 7.66 (td, *J* = 7.5, 1.2 Hz, 1H), 7.47 – 7.40 (m, 2H), 5.26 (ddd, *J* = 51.0, 7.8, 4.4 Hz, 1H), 3.62 (dt, *J* = 17.4, 7.7 Hz, 1H), 3.35 – 3.01 (m, 1H).

^{19}F NMR (471 MHz, $CDCl_3$) δ -194.02 (ddd, *J* = 51.1, 23.4, 7.2 Hz).

^{13}C NMR (126 MHz, $CDCl_3$) δ 200.1 (d, *J* = 14.8 Hz), 149.8, 136.5, 134.0, 128.6, 127.0, 124.9, 90.7 (d, *J* = 190.3 Hz), 33.6 (d, *J* = 21.4 Hz).

MP: 54 – 56 ^{o}C.

NMR data is identical to the literature data.[12]

3-Fluoro-3-(4-nitrophenyl)tetrahydrofuran (9a)

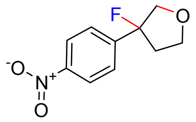

Prepared according to the general procedure **H** from 3-(4-nitrophenyl)but-3-en-1-ol (96 mg, 0.5 mmol) over 15 hr. The product was purified by column chromatography (SiO_2; cyclohexane: ethyl acetate 8:2) and obtained as a yellow oil (73 mg, 0.35 mmol, 69%).

Prepared according to the general procedure **J** using a 30 mL reactor coil (internal diameter = 1 mm, cooled to 0 ^{o}C), from 3-(4-nitrophenyl)but-3-en-1-ol (290 mg, 1.5 mmol). The product was purified by column chromatography (SiO_2; cyclohexane: ethyl acetate 8:2) and obtained as a yellow oil (216 mg, 1.02 mmol, 68%).

^{1}H NMR (500 MHz, $CDCl_3$) δ 8.25 (d, *J* = 8.4 Hz, 2H), 7.60 (d, *J* = 8.6 Hz, 2H), 4.28 – 4.17 (m, 3H), 3.99 (dd, *J* = 32.2, 10.8 Hz, 1H), 2.62 – 2.51 (m, 1H), 2.47 – 2.32 (m, 1H).

^{19}F NMR (471 MHz, $CDCl_3$) δ -148.47 (dddd, *J* = 33.1, 32.9, 23.0, 23.0 Hz, 1F).

^{13}C NMR (126 MHz, CDCl$_3$) δ 147.8, 146.6 (d, *J* = 24.0 Hz), 125.7 (d, *J* = 9.4 Hz), 124.0 (d, *J* = 1.4 Hz), 103.4 (d, *J* = 183.4 Hz), 79.5 (d, *J* = 26.8 Hz), 68.6, 41.8 (d, *J* = 23.7 Hz).

HRMS (ASAP+): calc: [M+H]$^+$ ($C_{10}H_{11}NO_3F$) 212.0723; found: 212.0731.

IR v$_{max}$/cm^{-1} (neat): 3078, 2954, 2873, 1602, 1517, 1435, 1338, 1128, 1109, 1070, 1001, 898, 850, 750, 696.

5-(Fluoromethyl)-3-phenyldihydrofuran-2(3H)-one (10a)

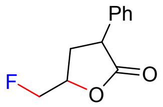

Prepared according to the general procedure **H** from 3-(4-nitrophenyl)but-3-en-1-ol (88 mg, 0.5 mmol) over 4 hr. The product was purified by column chromatography (SiO_2; cyclohexane: ethyl acetate 7:3) and obtained as a colourless oil (54 mg, 0.28 mmol, 56%).

Prepared according to the general procedure **J** using a 17.5 mL reactor coil (internal diameter = 1 mm), from 3-(4-nitrophenyl)but-3-en-1-ol (264 mg, 1.5 mmol). The product was purified by column chromatography (SiO_2; cyclohexane: ethyl acetate 7:3) and obtained as a colourless oil (178 mg, 0.92 mmol, 61%).

Isolated as a mixture of the two stereoisomers (2:1)

^{1}H NMR (400 MHz, CDCl$_3$) (major stereoisomer) δ 7.41 – 7.22 (m, 5H), 4.85 – 4.65 (m, 2H), 4.52 – 4.43 (m, 1H), 3.94 (t, *J* = 11.8 Hz, 1H), 2.78 – 2.66 (m, 1H), 2.43 – 2.29 (m, 1H).

^{19}F NMR (376 MHz, CDCl$_3$)(major stereoisomer) δ -230.60 (td, *J* = 47.2, 21.6 Hz).

^{1}H NMR (400 MHz, CDCl$_3$)(minor stereoisomer) δ 7.41 – 7.22 (m, 5H), 4.87 – 4.43 (m, 3H), 4.00 (t, *J* = 9.4 Hz, 1H), 2.87 – 2.77 (m, 1H), 2.60 – 2.47 (m, 1H).

^{19}F NMR (376 MHz, CDCl$_3$) (minor stereoisomer) δ -233.14 (td, *J* = 46.8, 28.7 Hz).

HRMS (ES+): calc: [M+H]$^+$ ($C_{11}H_{12}FO_2$) 195.0821; found: 195.0826.

NMR data is identical to the literature data.[14]

1-(Difluoromethyl)-2,3-dihydro-1H-indene (11)

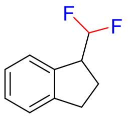

Due to the volatile nature of this product the yield was determined by ^{19}F NMR. A small amount (20 mg) was purified by preparative TLC (pentane 100%) to give the product as a yellow oil. Therefore, a pure NMR is still provided to allow true elucidation of the product.

Prepared according to the general procedure **H** from 1,2-dihydronaphthalene (65 mg, 0.5 mmol) over 2 hr. The yield was calculated by ^{19}F NMR spectroscopy using ethyl fluoroacetate as an internal standard (66 mg, 0.39 mmol, 78%).

Prepared according to the general procedure **J** using a 5 mL reactor coil (internal diameter = 1 mm), from 1,2-dihydronaphthalene (195 mg, 1.5 mmol). The yield was calculated by ^{19}F NMR spectroscopy using ethyl fluoroacetate as an internal standard (204 mg, 1.21 mmol, 81%).

^{1}H NMR (500 MHz, CDCl$_3$) δ 7.46 – 7.11 (m, 4H), 5.79 (td, *J* = 56.9, 5.5 Hz, 1H), 3.69-3.49 (m, 1H), 3.09 – 2.82 (m, 2H), 2.39 – 2.21 (m, 1H), 2.16 – 2.04 (m, 1H).

^{19}F NMR (471 MHz, CDCl$_3$) δ -118.9 - -119.3 (m, 2H).

^{13}C NMR (126 MHz, CDCl$_3$) δ 145.1, 139.0, 128.1, 126.7, 125.6, 125.0, 118.4 (t, *J* = 244.1 Hz), 49.2 (t, *J* = 20.6 Hz), 31.6, 25.6 (t, *J* = 4.3 Hz).

NMR data is identical to the literature data.[13]

(E)-(1-Fluoro-2-iodoethene-1,2-diyl)dibenzene (12a)

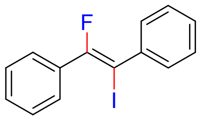

Prepared according to the general procedure **L** using a 35 mL reactor coil (internal diameter = 1 mm), from 1,2-diphenylethyne (267 mg, 1.5 mmol). The product was purified by column chromatography (SiO$_2$; cyclohexane) and obtained as a colourless oil (428 mg, 1.32 mmol, 88%).

^{1}H NMR (500 MHz, CDCl$_3$) δ 7.85 – 7.75 (m, 1H), 7.65 – 7.30 (m, 1H).

^{19}F NMR (471 MHz, CDCl$_3$) δ -76.30 (s).

^{13}C NMR (126 MHz, CDCl$_3$) δ 154.93 (d, *J* = 250.8 Hz), 131.79, 130.23 (d, *J* = 1.9 Hz), 129.88 (d, *J* = 2.7 Hz), 128.56, 128.52, 128.43, 128.38, 128.26, 89.56.

HRMS (EI+): calc: [M+H]$^+$ ($C_{14}H_{10}IF^{127}$) 323.98057; measured: 323.9804.

NMR data is identical to the literature data.[17]

(E)-(1-Fluoro-2-iodoprop-1-en-1-yl)benzene (12b)

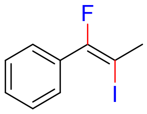

Prepared according to the general procedure **L** using a 35 mL reactor coil (internal diameter = 1 mm), from prop-1-yn-1-ylbenzene (174 mg, 1.5 mmol). The yield was determined by ^{19}F NMR Spectroscopy using ethyl fluoroacetate as an internal standard (71%).

^{1}H NMR (500 MHz, CDCl$_3$) δ 7.66 – 7.27 (m, 5H), 2.59 (d, *J* = 3.6 Hz, 3H).

^{19}F NMR (471 MHz, CDCl$_3$) δ -83.14 (s).

HRMS (EI+): calc: [M+H]$^+$ ($C_9H_8IF^{127}$) 261.96492; measured: 261.9648.

NMR data is identical to the literature data.[17]

(2-Fluoro-3-iodopropyl)benzene (12ca)

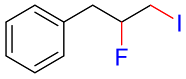

Prepared according to the general procedure **L** using a 35 mL reactor coil (internal diameter = 1 mm), from allylbenzene (177 mg, 1.5 mmol). The product was purified by column chromatography (SiO_2; cyclohexane) and obtained as a colourless oil (170 mg, 0.65 mmol, 43%).

^{1}H NMR (500 MHz, CDCl$_3$) δ 7.35 – 7.31 (m, 2H), 7.29 – 7.24 (m, 3H), 5.15 – 4.27 (m, 1H), 3.35 – 3.22 (m, 2H), 3.09 (dd, *J* = 20.9, 6.0 Hz, 2H) (trace amounts of 4-iodotoluene present)

^{19}F NMR (471 MHz, CDCl$_3$) δ -168.13 – -168.43 (m).

^{13}C NMR (126 MHz, CDCl$_3$) δ 135.76 (d, *J* = 5.2 Hz), 129.61, 128.87, 127.20, 92.31 (d, *J* = 178.3 Hz), 40.91 (d, *J* = 21.1 Hz), 6.48 (d, *J* = 24.3 Hz).

NMR data is identical to the literature data.[18]

(2,3-Difluoropropyl)benzene (12cb)

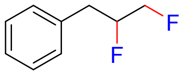

Prepared according to the general procedure **H** from allyl benzene (59 mg, 0.5 mmol) and I_2 (127 mg, 0.5 mmol) over 12 hr. The product was purified by column chromatography (SiO_2; cyclohexane) and obtained as a colourless oil (30 mg, 0.19 mmol, 39%).

^{1}H NMR (400 MHz, CDCl$_3$) δ 7.35 – 7.30 (m, 1H), 7.29 – 7.21 (m, 2H), 4.97 – 4.73 (m, 1H), 4.55 (dddd, *J* = 44.2, 23.1, 10.5, 2.1 Hz, 1H), 4.45 (dddd, *J* = 43.4, 24.7, 11.0, 4.9 Hz, 1H), 3.13 – 2.92 (m, 1H).

^{19}F NMR (376 MHz, CDCl$_3$) δ -186.27 – -186.71 (m), -231.90 – -232.30 (m).

NMR data is identical to the literature data.[2]

(2*R*,3*R*,4*S*,5*S*,6*R*)-2-(Acetoxymethyl)-6-fluoro-5-iodotetrahydro-2*H*-pyran-3,4-diyl diacetate (12d)

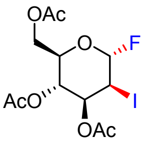

Prepared according to the general procedure **L** using a 35 mL reactor coil (internal diameter = 1 mm), from (2*R*,3*S*,4*R*)-2-(acetoxymethyl)-3,4-dihydro-2H-pyran-3,4-diyl diacetate (408 mg, 1.5 mmol). The yield was determined by ^{19}F NMR Spectroscopy using ethyl fluoroacetate as an internal standard (65%).

^{1}H NMR (500 MHz, CDCl$_3$) δ 5.93 (dd, *J* = 51.0, 1.3 Hz, 1H), 5.53 (dd, *J* = 36.3, 2.2 Hz, 1H), 4.65 (td, *J* = 4.2, 1.3 Hz, 1H), 4.59 (dd, *J* = 9.6, 4.5 Hz, 1H), 4.37 – 4.14 (m, 3H), 2.13 – 2.08 (m, 9H).

^{19}F NMR (471 MHz, CDCl$_3$) δ -116.34 (dd, *J* = 51.1, 3.9 Hz).

NMR data is identical to the literature data.[19]

(2*R*,3*R*,4*S*,5*R*,6*R*)-2-(Acetoxymethyl)-6-fluorotetrahydro-2*H*-pyran-3,4,5-triyl triacetate (13a)

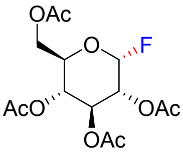

Prepared according to the general procedure **L** using a 35 mL reactor coil (internal diameter = 1 mm), from (2*R*,3*R*,4*S*,5*R*,6*S*)-2-(acetoxymethyl)-6-((4-chlorophenyl)thio)tetrahydro-2*H*-pyran-3,4,5-triyl triacetate (712 mg, 1.5 mmol). The yield was determined by ^{19}F NMR Spectroscopy using ethyl fluoroacetate as an internal standard (40%).

^{1}H NMR (500 MHz, CDCl$_3$) δ 5.68 (dd, *J* = 52.8, 2.8 Hz, 1H), 5.43 (t, *J* = 9.9 Hz, 1H), 5.09 (t, *J* = 9.9 Hz, 1H), 4.94 – 4.83 (m, 1H), 4.28 – 4.15 (m, 3H), 2.07 (3H, s), 2.07 (3H, s), 2.01 (3H, s), 2.0 (3H, s)

^{19}F NMR (471 MHz, CDCl$_3$) δ -149.68 (dd, *J* = 53.0, 24.3 Hz).

NMR data is identical to the literature data.[20]

2-(4,5-Difluoropentyl)isoindoline-1,3-dione (14a)

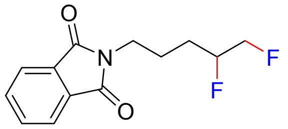

Prepared according to the general procedure **K** using a 70 mL reactor coil (internal diameter = 1 mm), from 2-(pent-4-en-1-yl)isoindoline-1,3-dione (9.49 g, 44.1 mmol). The product was purified by column chromatography (SiO$_2$; cyclohexane: diethyl ether 9:1) and obtained as a colourless oil (8.38 g, 33.09 mmol, 75%).

^{1}H NMR (400 MHz, CDCl$_3$) δ 7.82 (dd, *J* = 5.5, 3.0 Hz, 2H), 7.70 (dd, *J* = 5.4, 3.1 Hz, 2H), 4.84 – 4.27 (m, 3H), 3.72 (t, *J* = 7.0 Hz, 2H), 2.00 – 1.48 (m, 4H).

^{19}F NMR (376 MHz, CDCl$_3$) δ -189.72 – -190.20 (m, 1F), -230.25 (tdd, *J* = 47.4, 20.8, 13.3 Hz, 1F).

^{13}C NMR (101 MHz, CDCl$_3$) δ 168.48 (s), 134.15 (s), 132.14 (s), 123.39 (s), 91.20 (dd, *J* = 173.4, 19.4 Hz), 84.01 (dd, *J* = 174.0, 22.9 Hz), 37.44 (s), 27.49 (dd, *J* = 21.3, 6.7 Hz), 24.22 (d, *J* = 4.1 Hz).

HRMS (CI+): calc: [M+H]$^+$ ($C_{13}H_{13}O_2NF_2$) 253.09089; found: 253.0911.

IR v_{max}/cm^{-1} (neat): 2953, 1770.65, 1770, 1616, 1467, 1396, 1361, 1029, 962, 918, 854, 717, 528.

References:

1 J. D. Haupt, M. Berger and S. R. Waldvogel, *Org. Lett.,* 2019, **21**, 242.
2 S. Doobary, A. T. Sedikides, H. P. Caldora, D. L. Poole and A. J. J. Lennox*, Angew. Chem. Int. Ed.,* 2020, **59**, 1155.
3 A. Dierks, M. Schmidtmann and J. Christoffers, *Chem. Eur. J.,* 2019, **25**, 5451.
4 S. Companys, P. A. Peixoto, C. Bosset, S. Chassaing, K. Miqueu, J. -M. Sotiropoulos, L. Pouységu and S. Quideau, *Chem. Eur. J.,* 2017, **23**, 13309.
5 P. Gan, M. W. Smith, N. R. Braffman and S. A. Snyder, *Angew. Chem. Int. Ed.,* 2016, **55**, 3625.
6 N. Duguet, A. M. Z. Slawin and A. D. Smith, *Org. Lett.,* 2009, **11**, 3858.
7 M. Elsherbini , B. Winterson, H. Alharbi, A. A. Folgueiras-Amador, C. Génot and T. Wirth, *Angew. Chem. Int. Ed.,* 2019, **58**, 9811.
8 C. Ye, B. Twamley and J. M. Shreeve, *Org. Lett.,* 2005, **7**, 3961.
9 T. Kitamura, S. Kuriki and M. H. Morshed, Y. Hori, *Org. Lett.,* 2011, **13**, 2392.
10 R. Pluta, P. E. Krach, L. Cavallo, L. Falivene and M. Rueping, *ACS Catal.,* 2018, **8**, 2582.
11 X. Wang, Q. Lan, S. Shirakawa and K. Maruoka, *Chem. Commun.,* 2010, **46**, 321.
12 T. Kitamura, K. Muta and K. Muta, *J. Org. Chem.,* 2014, **79**, 5842.
13 T. Kitamura, K. Muta and J. Oyamada, *J. Org. Chem.,* 2015, **80**, 10431.
14 M. Sawaguchi, S. Hara, T. Fukuhara and N. Yoneda, *J. Fluorine Chem.,* 2000, **104**, 277.
15 https://www.vapourtec.com/products/flow-reactors/ion-electrochemical-reactor-features/, (accessed April 2021).
16 https://www.ika.com/en/Products-Lab-Eq/Electrochemistry-Kit-csp-516/ElectraSyn-20-pro-Package-cpdt-40003261/, (accessed April 2021).
17 M. Zupan, *Synthesis,* 1976, 473.
18 N. O. Ilchenko, M. A. Cortés and K. J. Szabó, *ACS Catal.,* 2016, **6**, 447.
19 R. D. Evans and J. H. Schauble, *Synthesis,* 1987, 551.
20 S. Caddick, L. Gazzard, W. B. Motherwell and J. A. Wilkinson*, Tetrahedron,* 1996, **52**, 149.
21 F. Scheidt, C. Thiehoff, G. Yilmaz, S. Meyer, C. G. Daniliuc, G. Kehr and R. Gilmour, *Beilstein J. Org. Chem.,* 2018, **14***,* 1021.
22 Y. Liu, H. Wu, Y. Guo, J.-C. Xiao, Q.-Y. Chen and C. Liu, *Angew. Chem. Int. Ed.,* 2017, **56**, 15432.
23 https://www.zaiput.com/product/liquid-liquid-gas-separators/, (accessed 03/06/2021).

NMR Spectra for the Starting Materials:

^{1}H NMR of *N*-Allyl-4-methylbenzamide (1a)

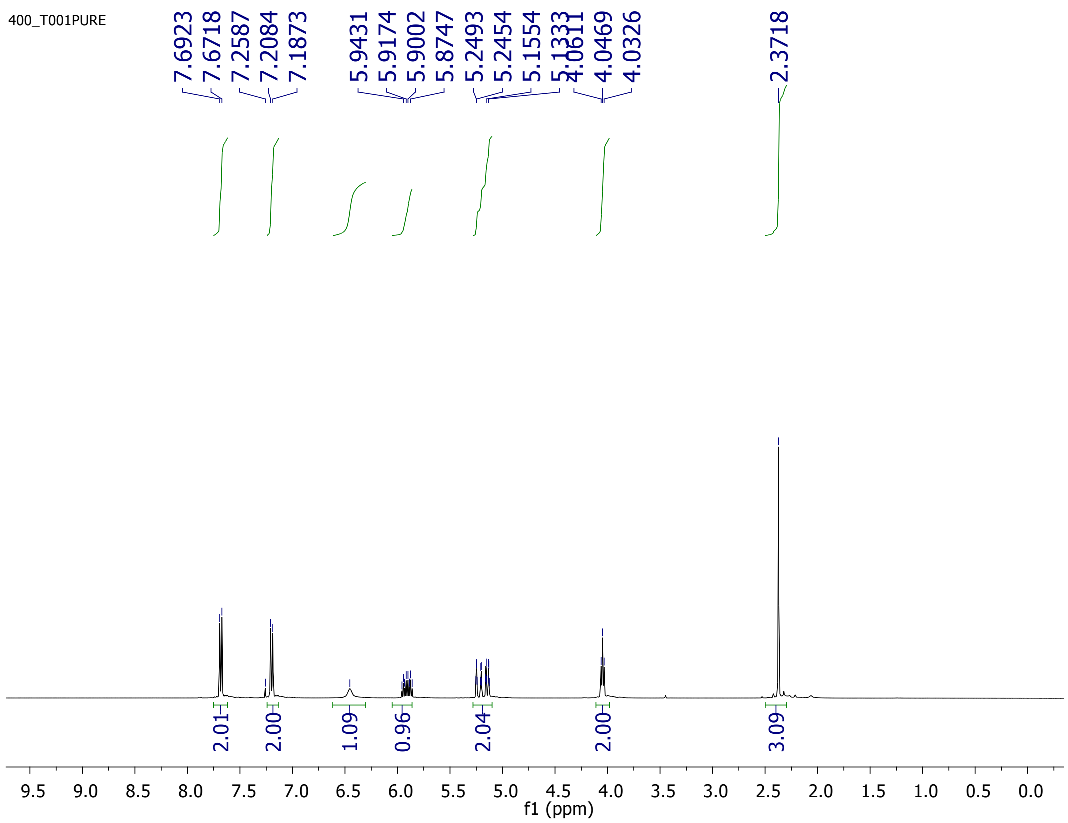

^{13}C NMR of *N*-Allyl-4-methylbenzamide (1a)

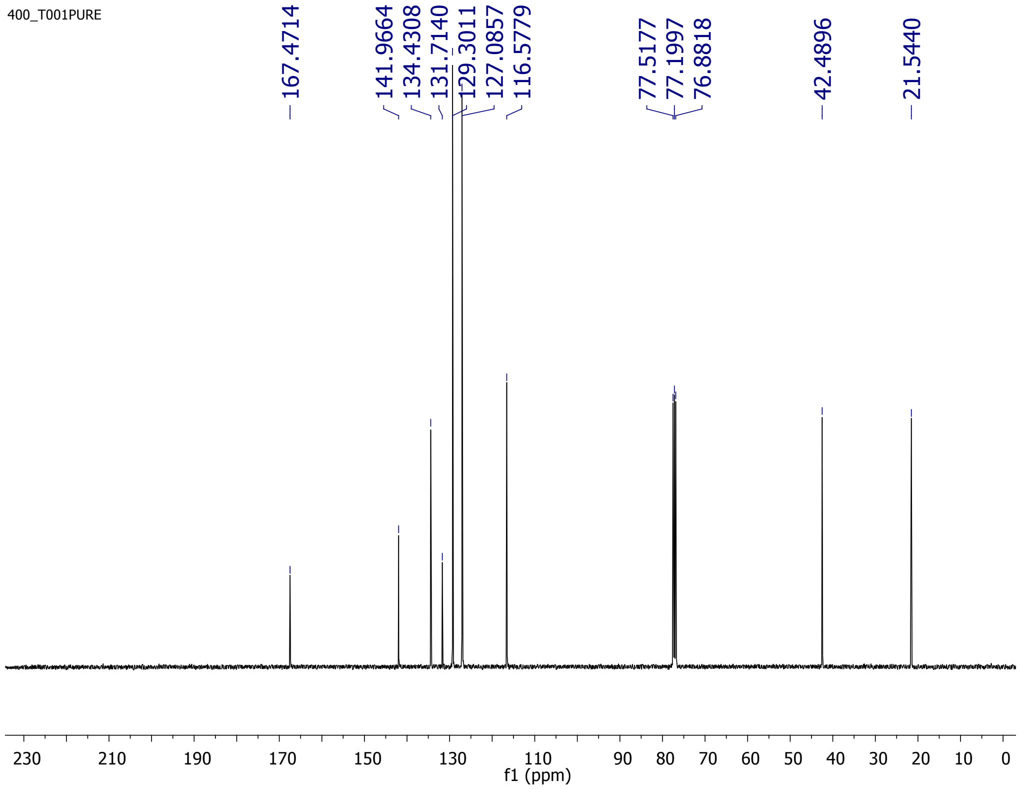

^{1}H NMR of *N*-Allylbenzamide (1b)

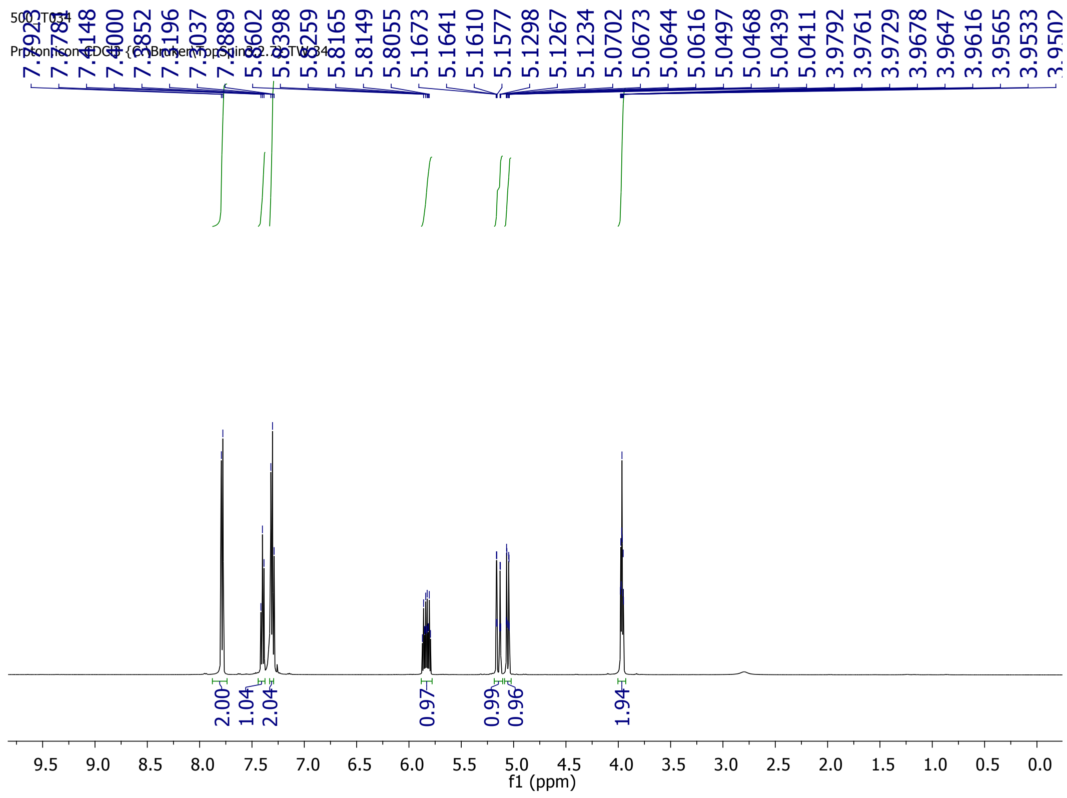

^{13}C NMR of *N*-Allylbenzamide (1b)

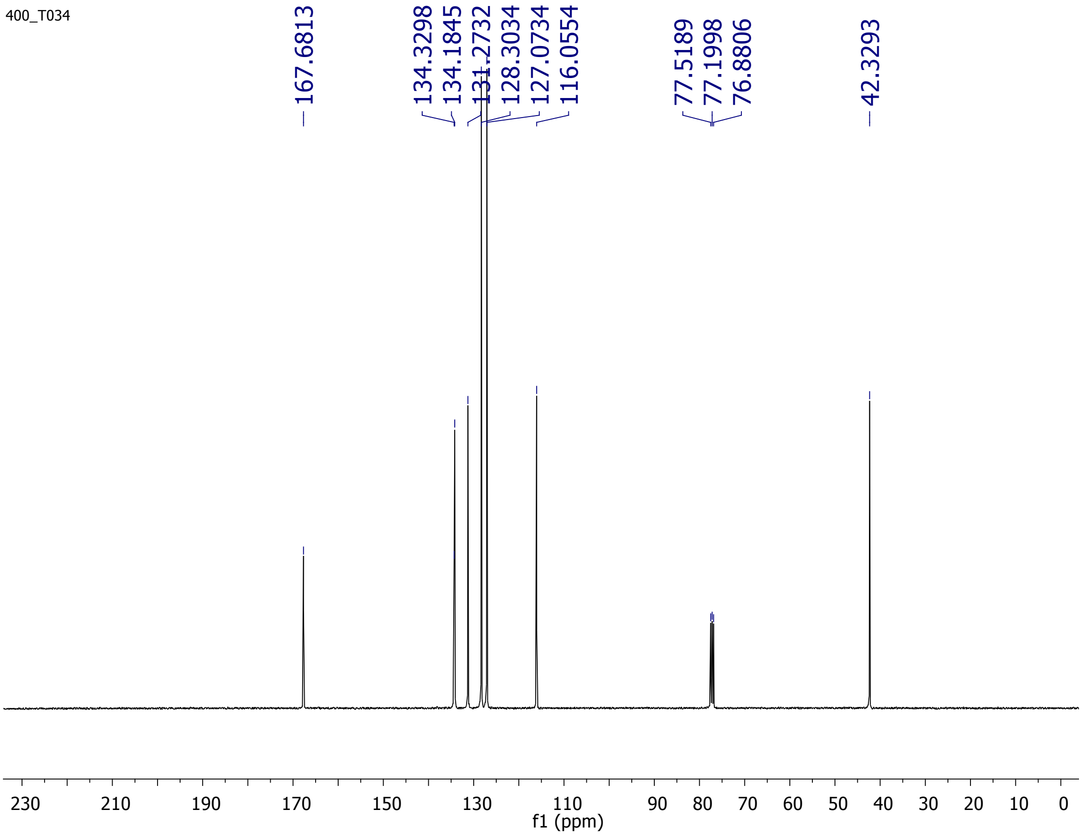

^{1}H NMR of *N*-Allyl-4-nitrobenzamide (1c)

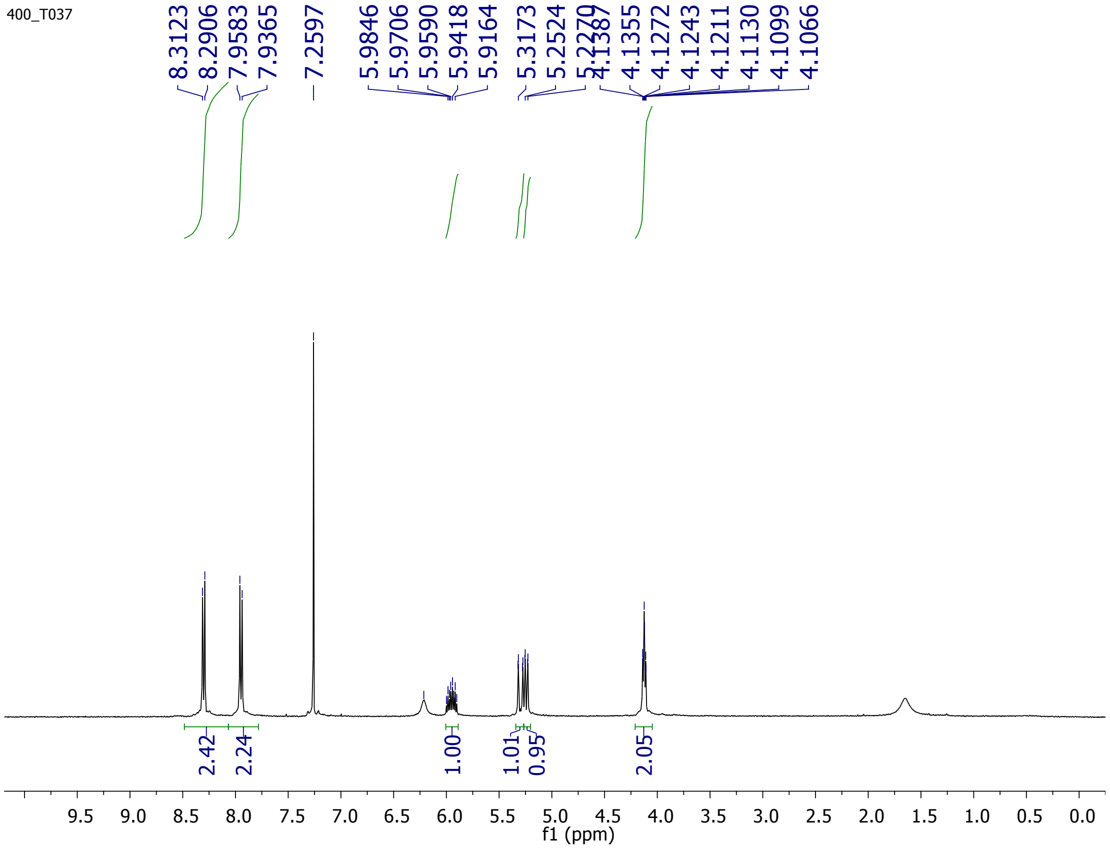

^{13}C NMR of *N*-Allyl-4-nitrobenzamide (1c)

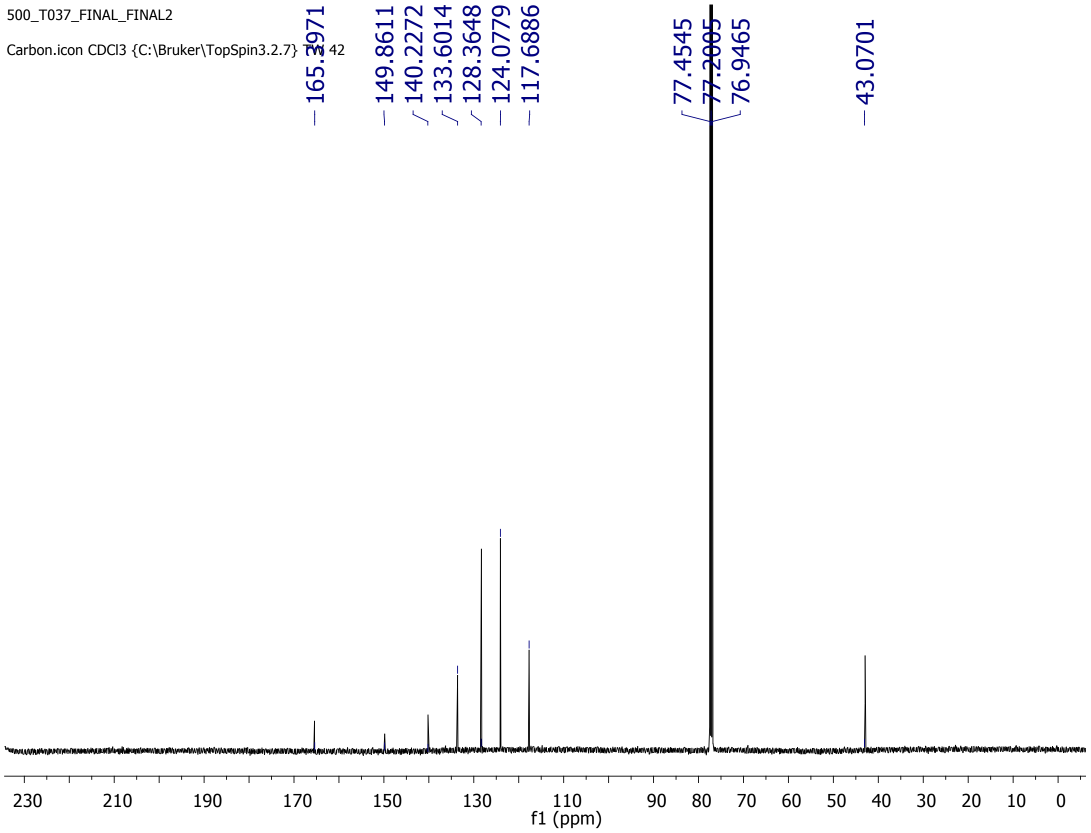

^{1}H NMR of *N*-Allyl-4-(trifluoromethyl)benzamide (1d)

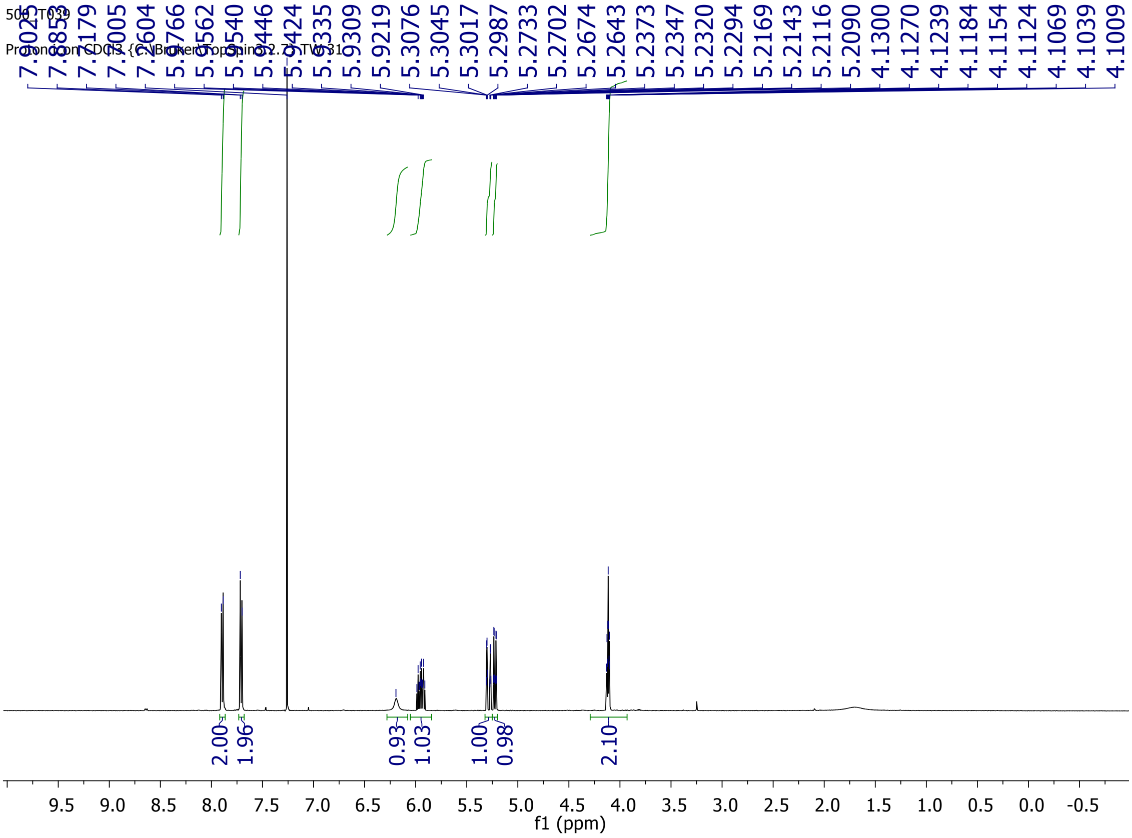

^{19}F NMR of *N*-Allyl-4-(trifluoromethyl)benzamide (1d)

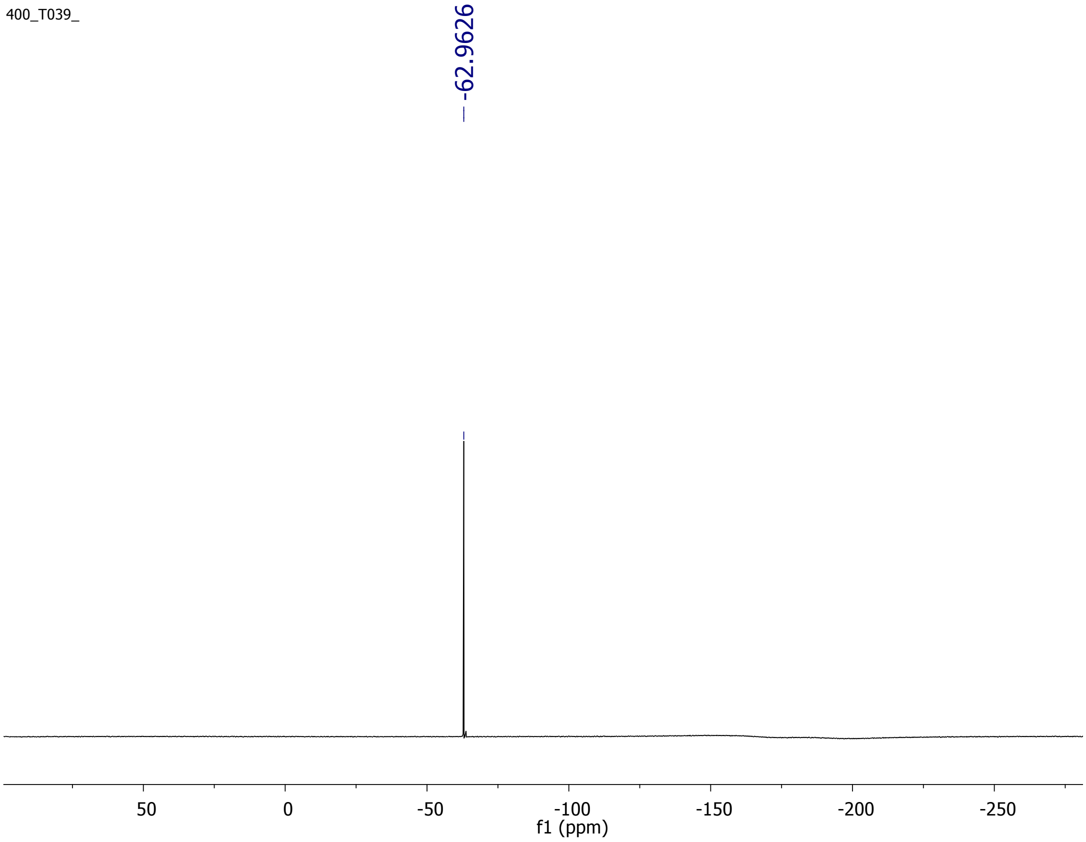

^{13}C NMR of *N*-Allyl-4-(trifluoromethyl)benzamide (1d)

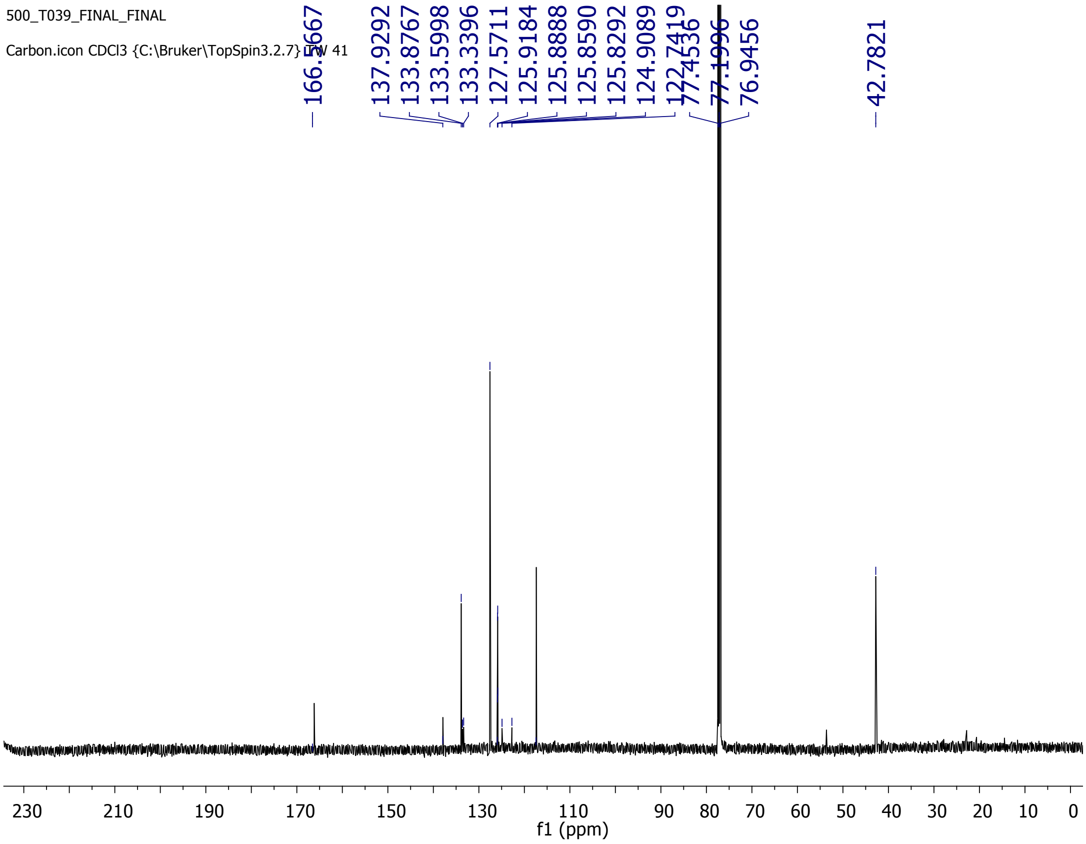

^{1}H NMR of *N*-Allyl-4-methoxybenzamide (1e)

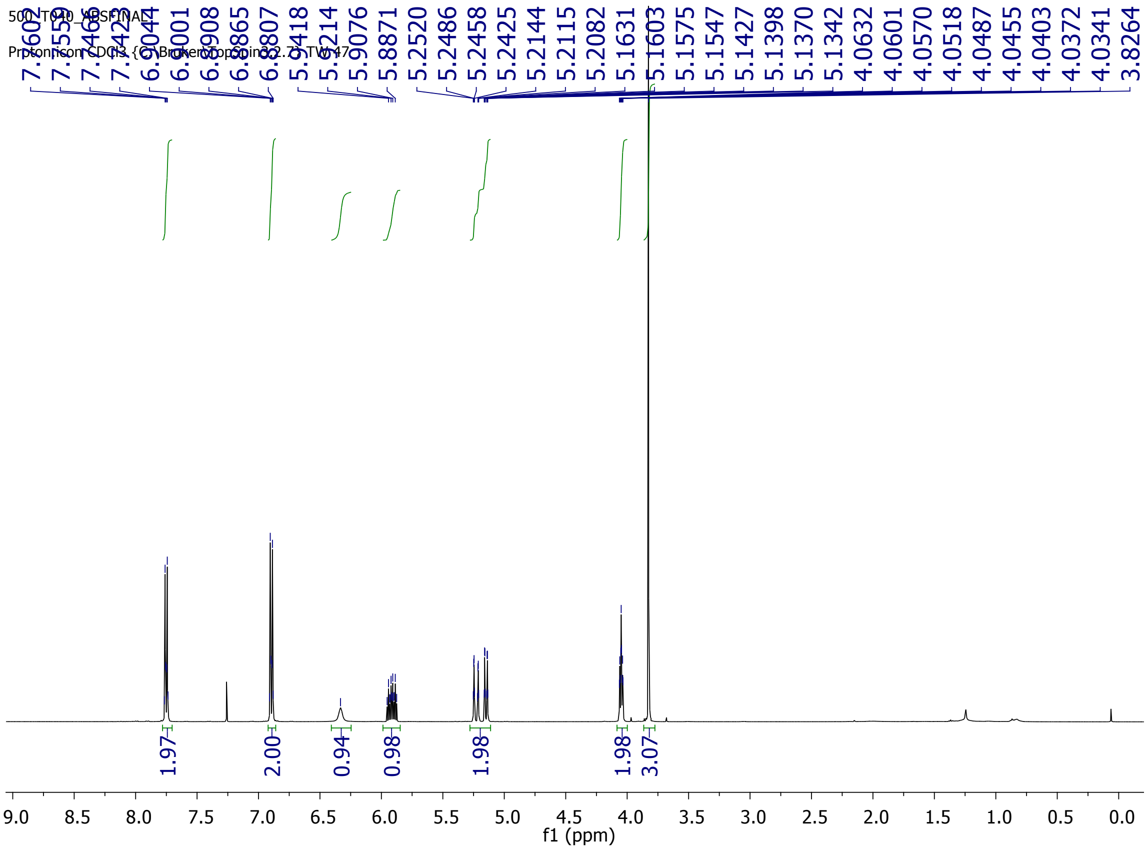

^{13}C NMR of *N*-Allyl-4-methoxybenzamide (1e)

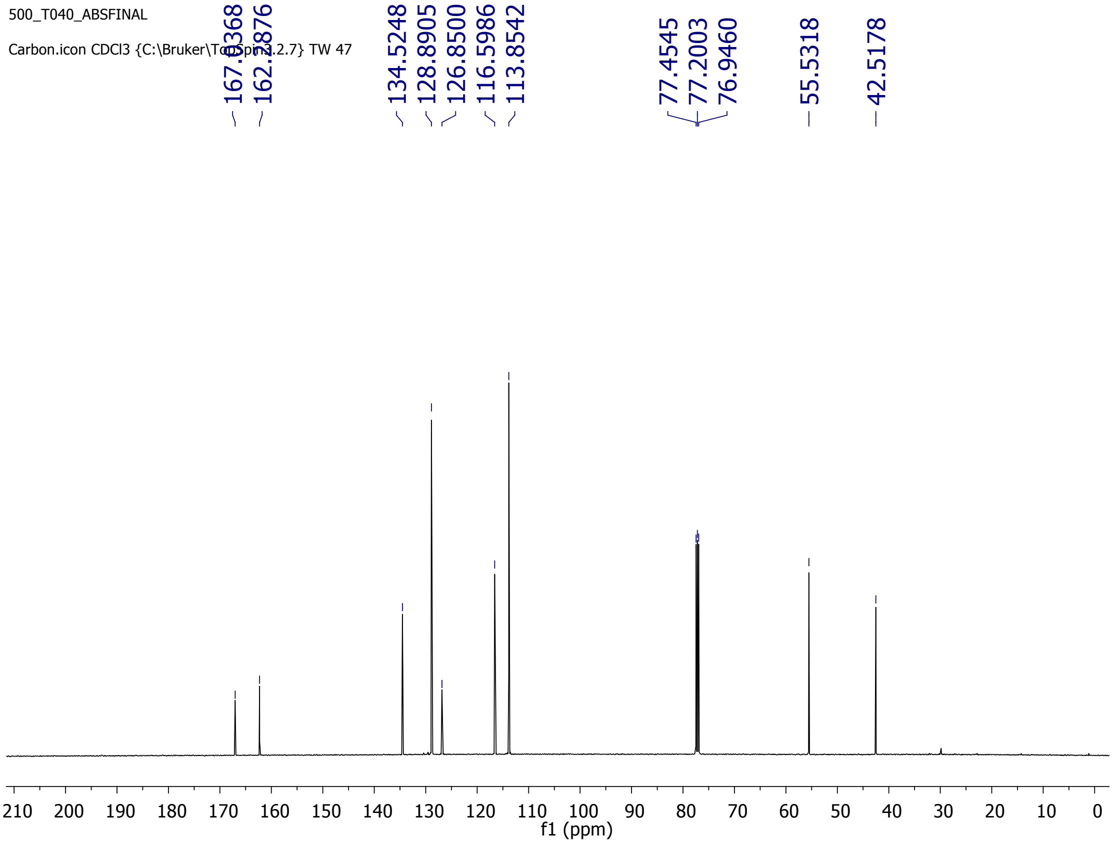

^{1}H NMR of *N*-Allyltetrahydrofuran-2-carboxamide (1i)

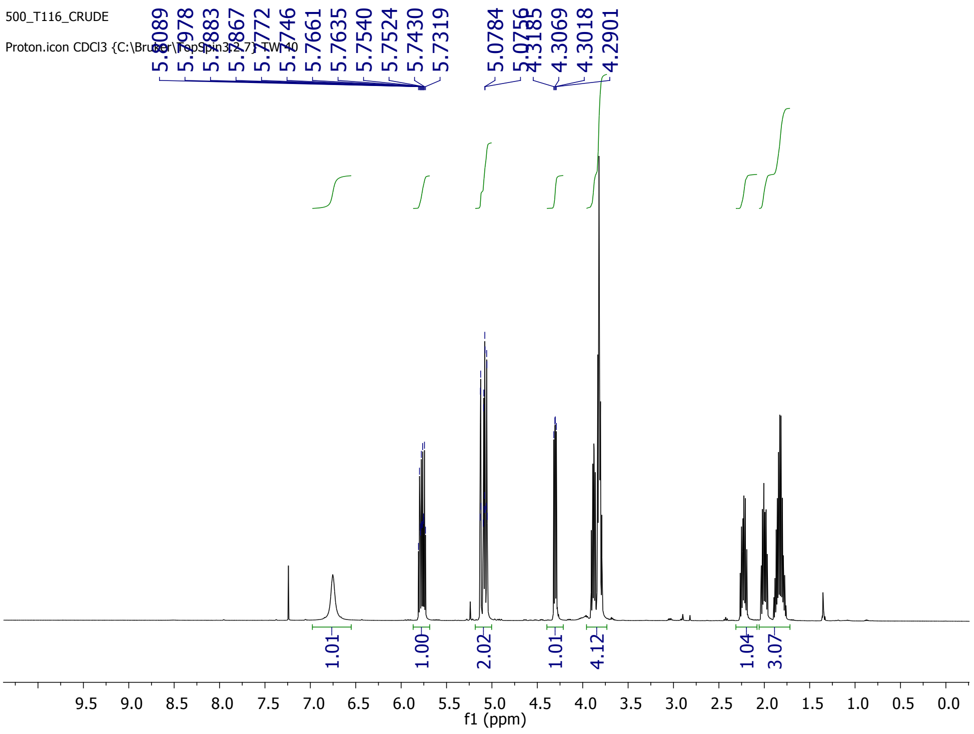

^{13}C NMR of *N*-Allyltetrahydrofuran-2-carboxamide (1i)

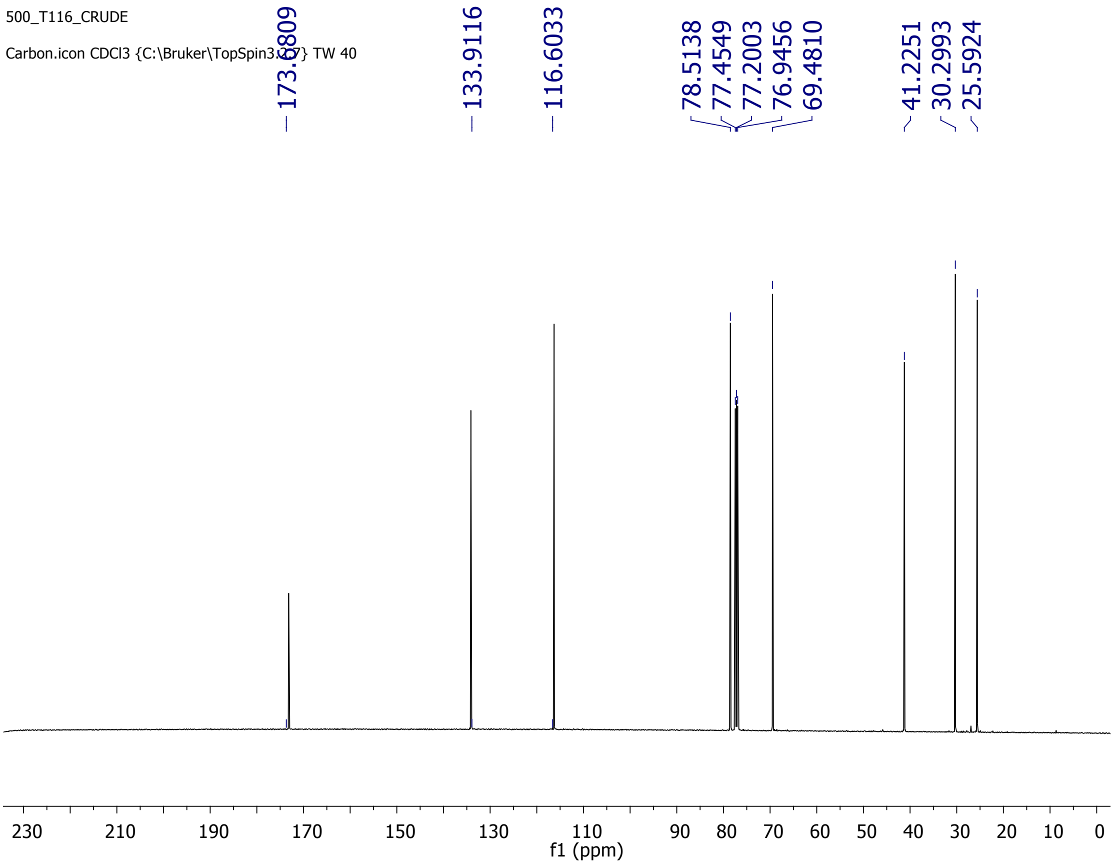

^{1}H NMR of *N*-allylmorpholine-4-carboxamide (1j)

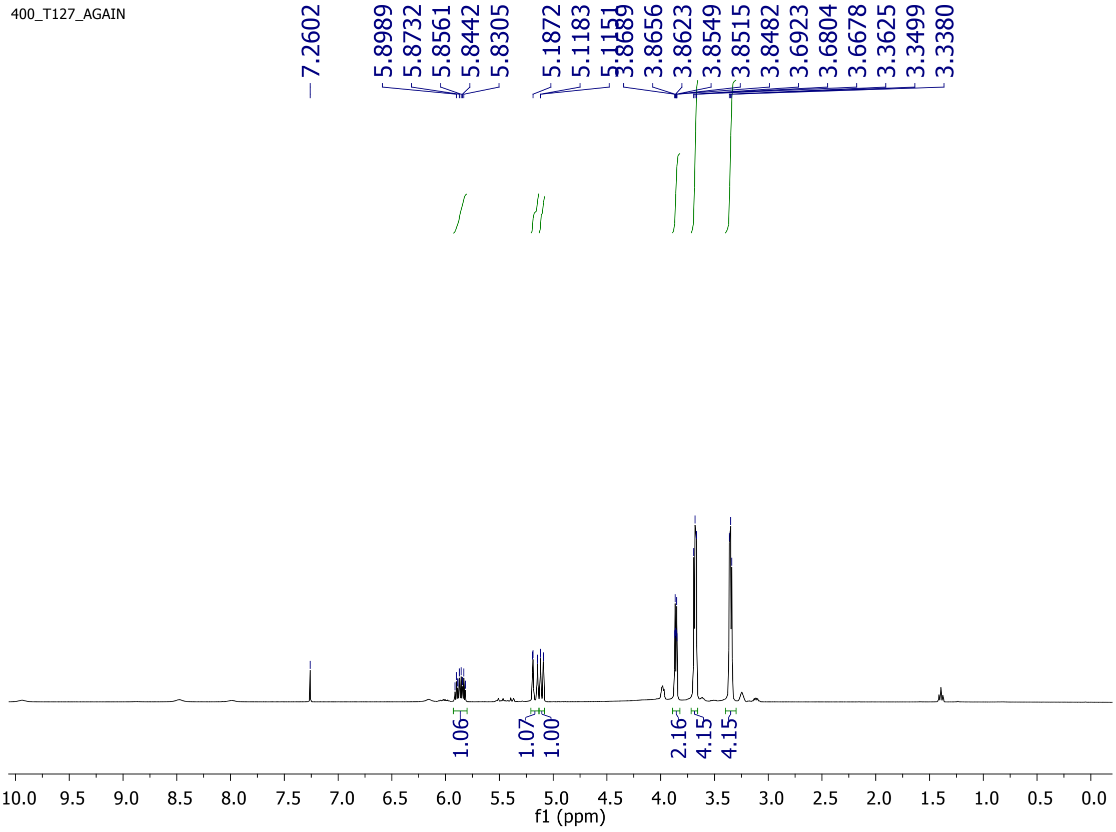

^{13}C NMR of *N*-allylmorpholine-4-carboxamide (1j)

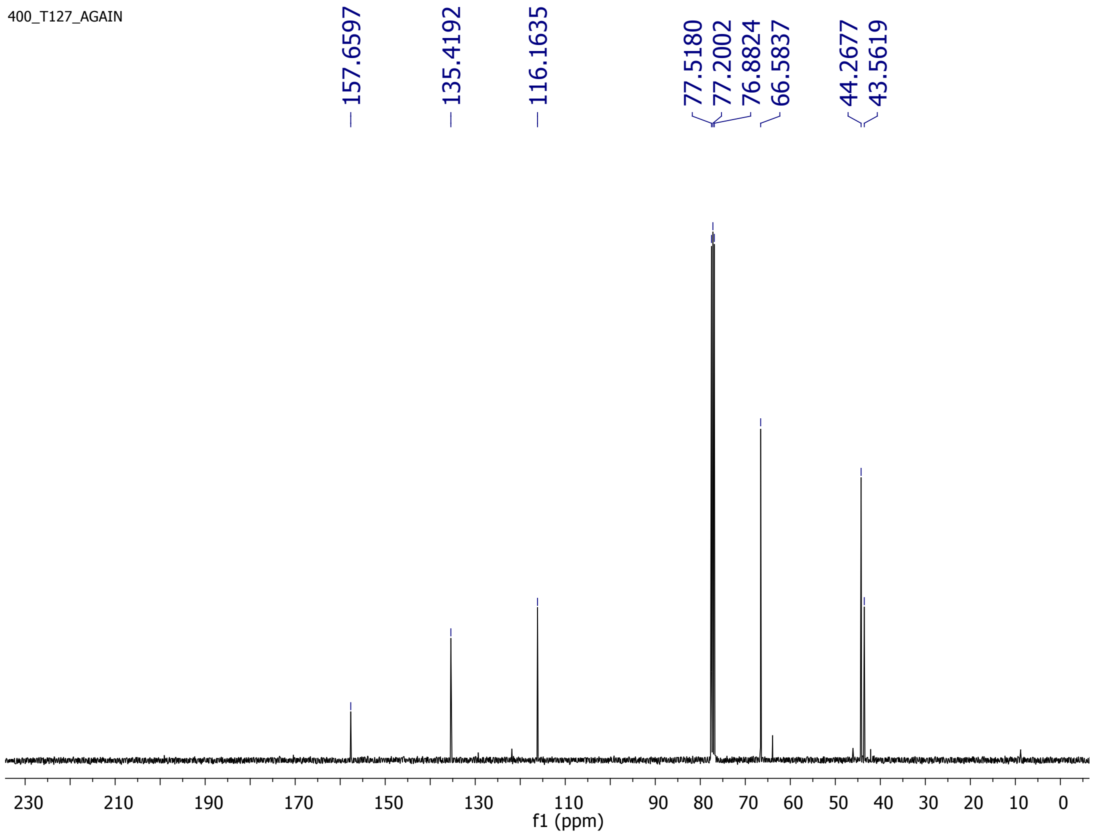

^{1}H NMR of *N*-allylpyrrolidine-1-carboxamide (1k)

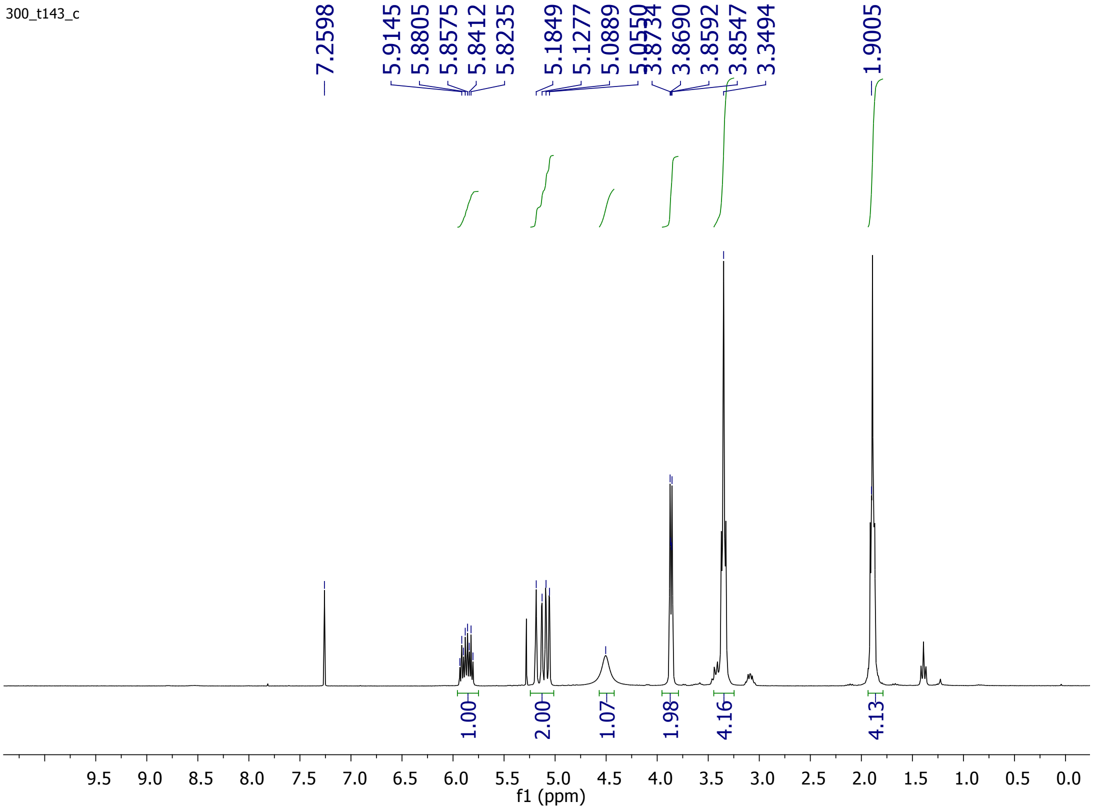

^{13}C NMR of *N*-allylpyrrolidine-1-carboxamide (1k)

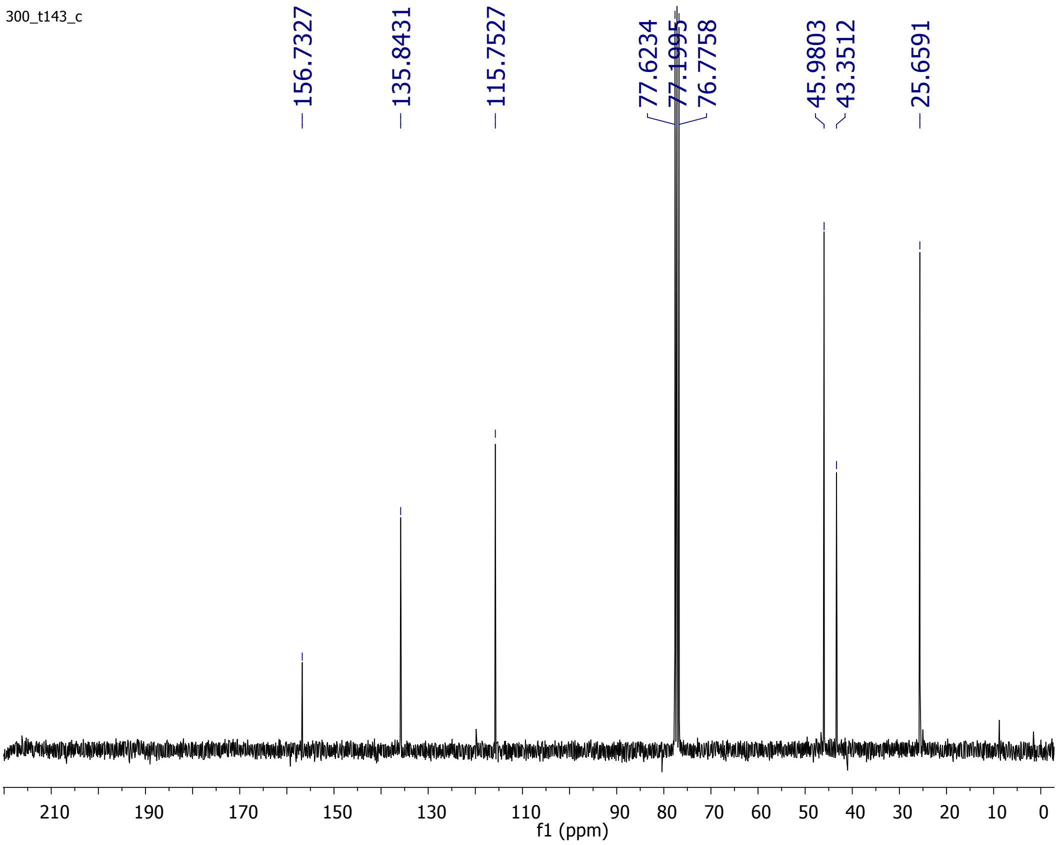

^{1}H NMR of Benzyl 4-(allylamino)-4-oxobutanoate (1l)

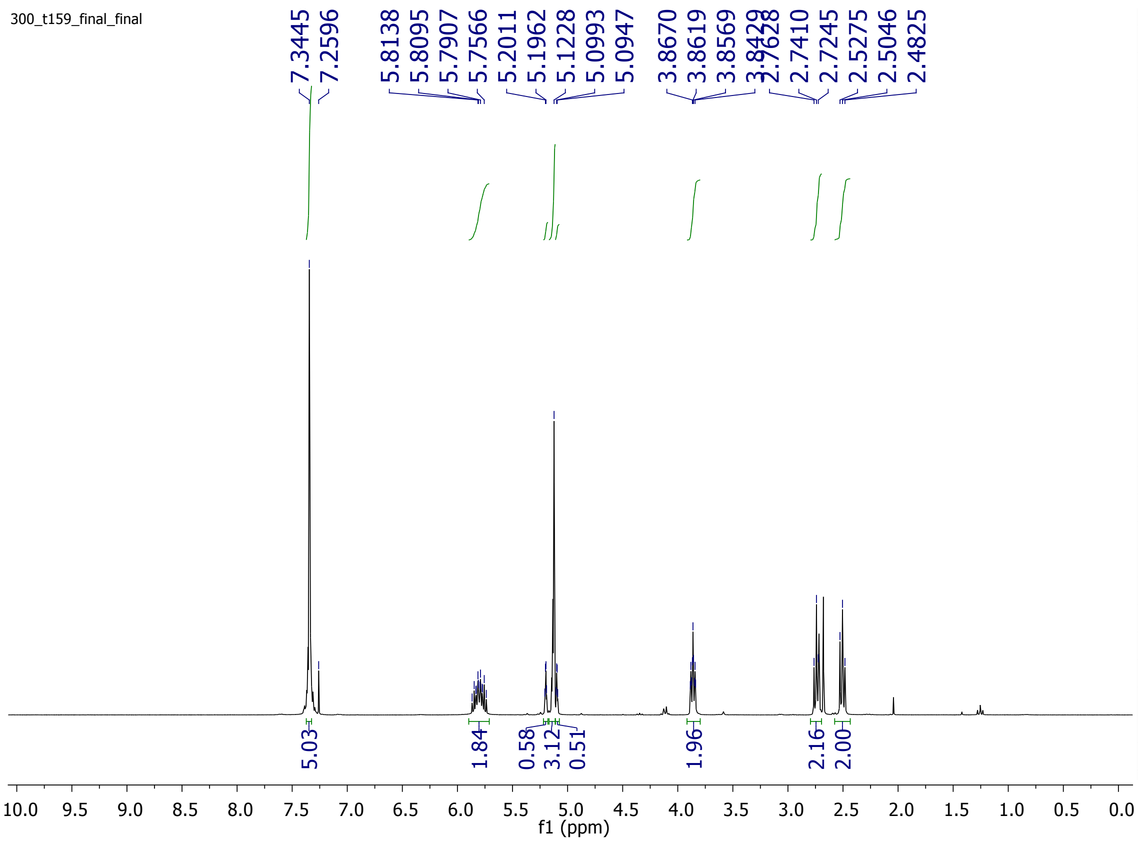

^{13}C NMR of Benzyl 4-(allylamino)-4-oxobutanoate (1l)

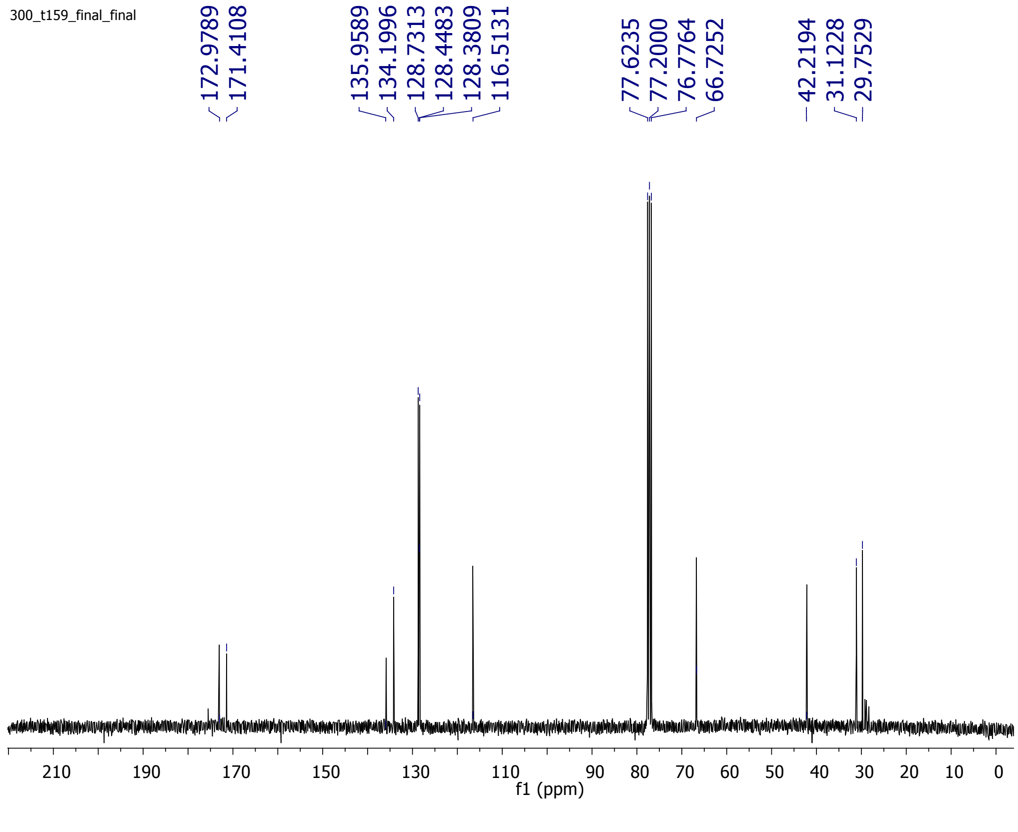

^{1}H NMR of *N*-Allylpiperidine-1-carboxamide (1m)

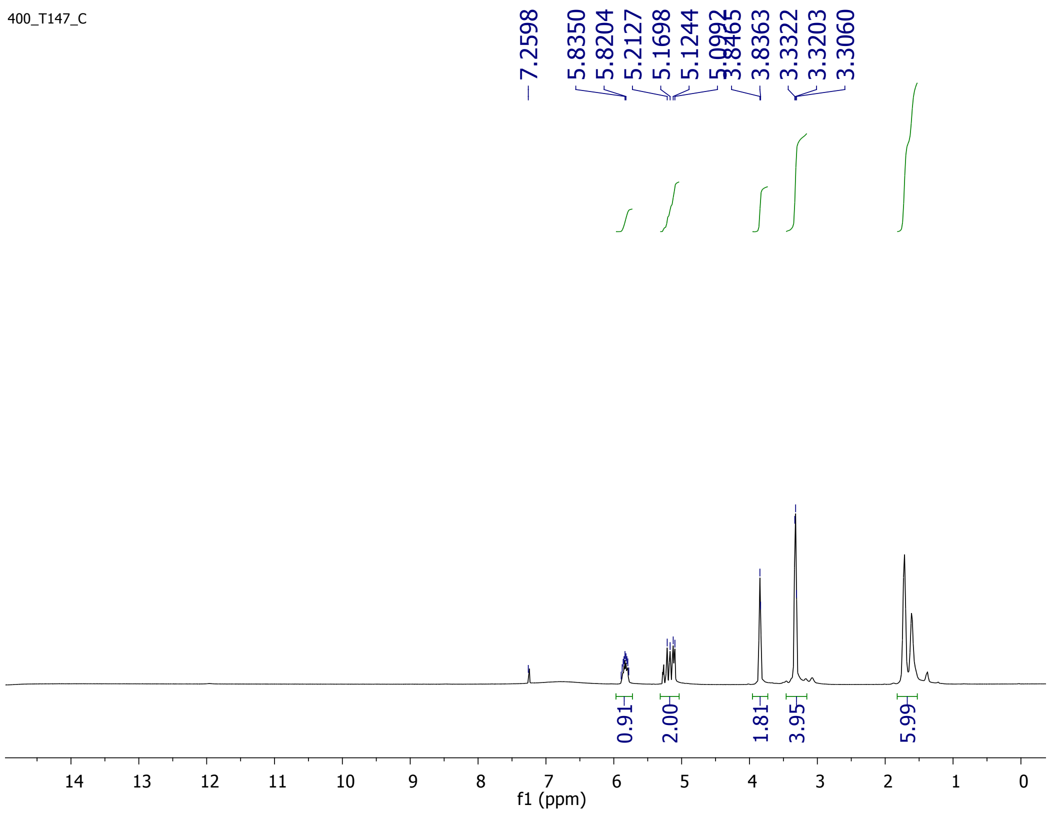

^{13}C NMR of *N*-Allylpiperidine-1-carboxamide (1m)

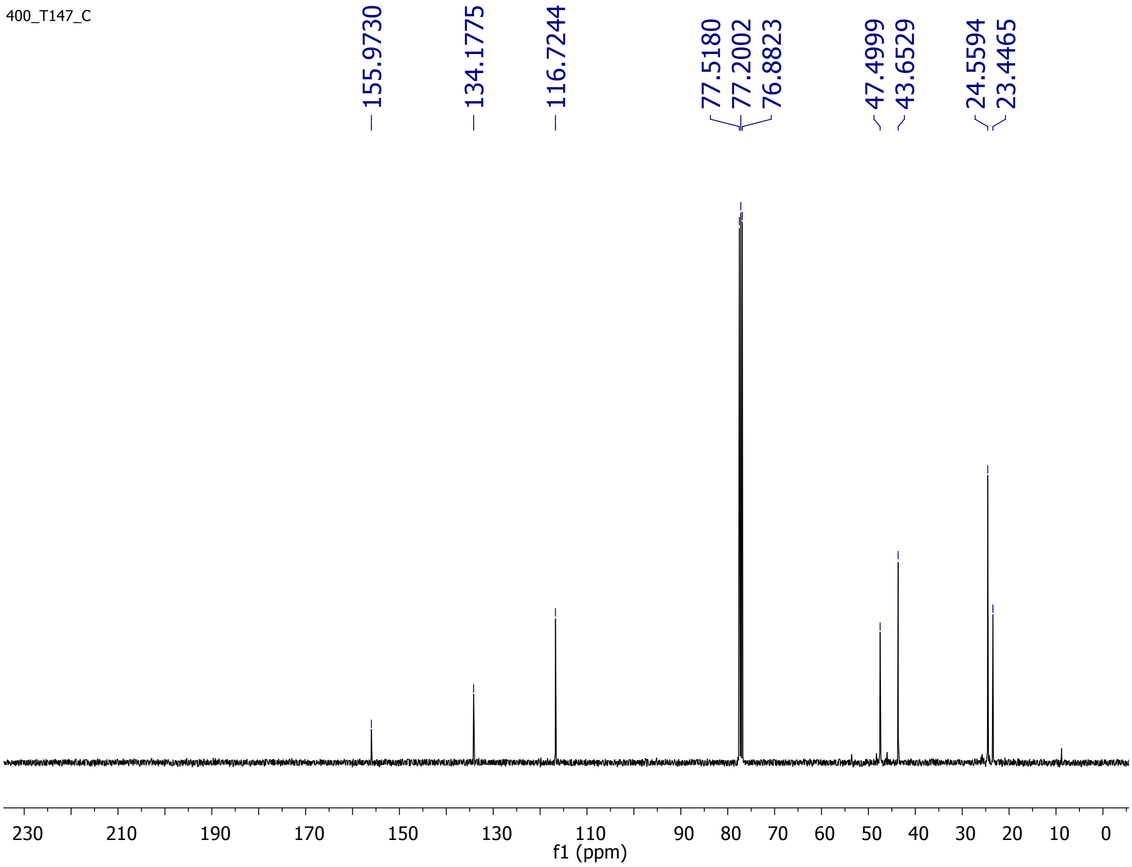

^{1}H NMR of 1-((Allyloxy)methyl)-4-fluorobenzene (6aa)

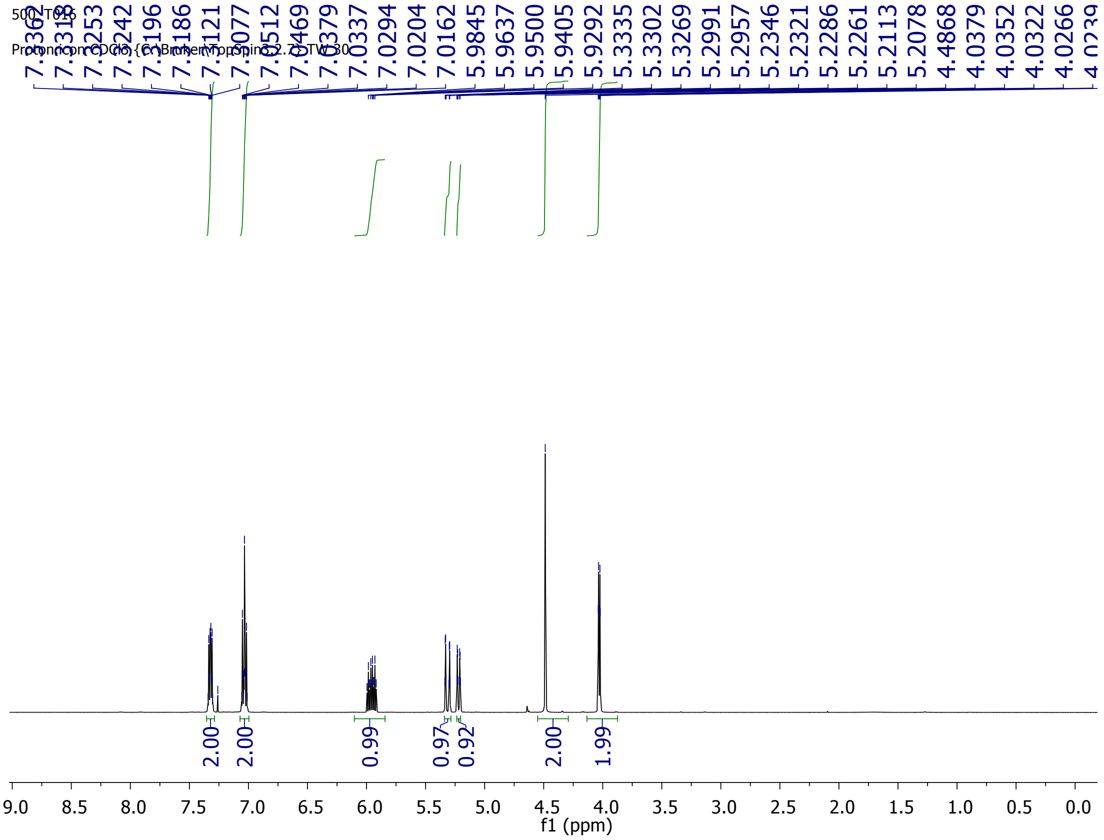

^{13}C NMR of 1-((Allyloxy)methyl)-4-fluorobenzene (6aa)

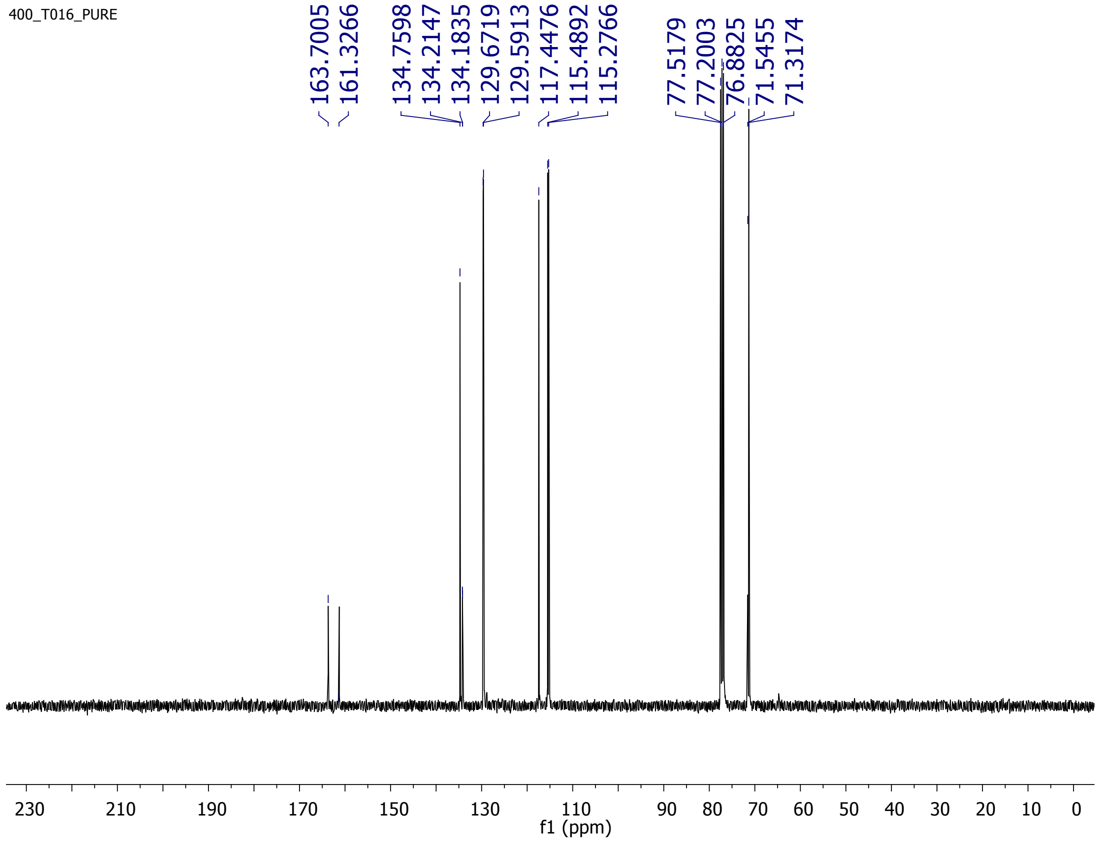

^{1}H NMR of 1-((Allyloxy)methyl)-3,5-dinitrobenzene (6ba)

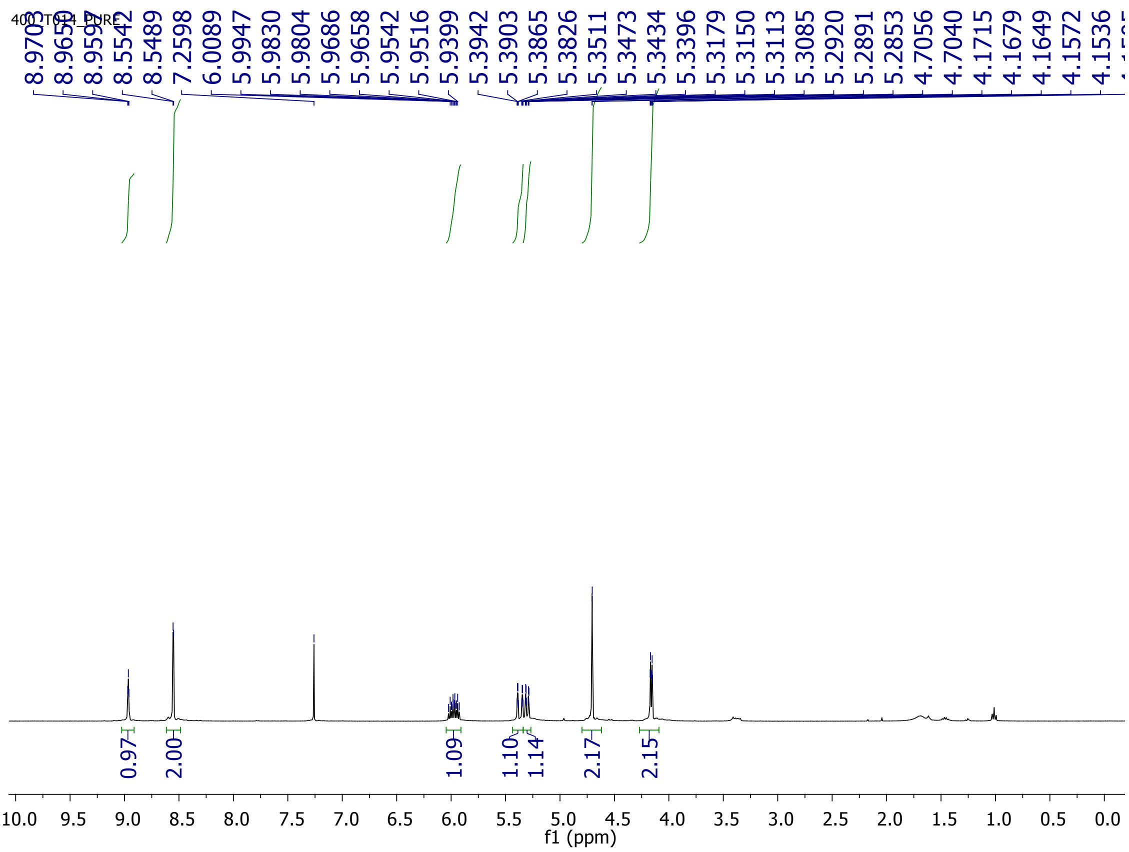

^{13}C NMR of 1-((Allyloxy)methyl)-3,5-dinitrobenzene (6ba)

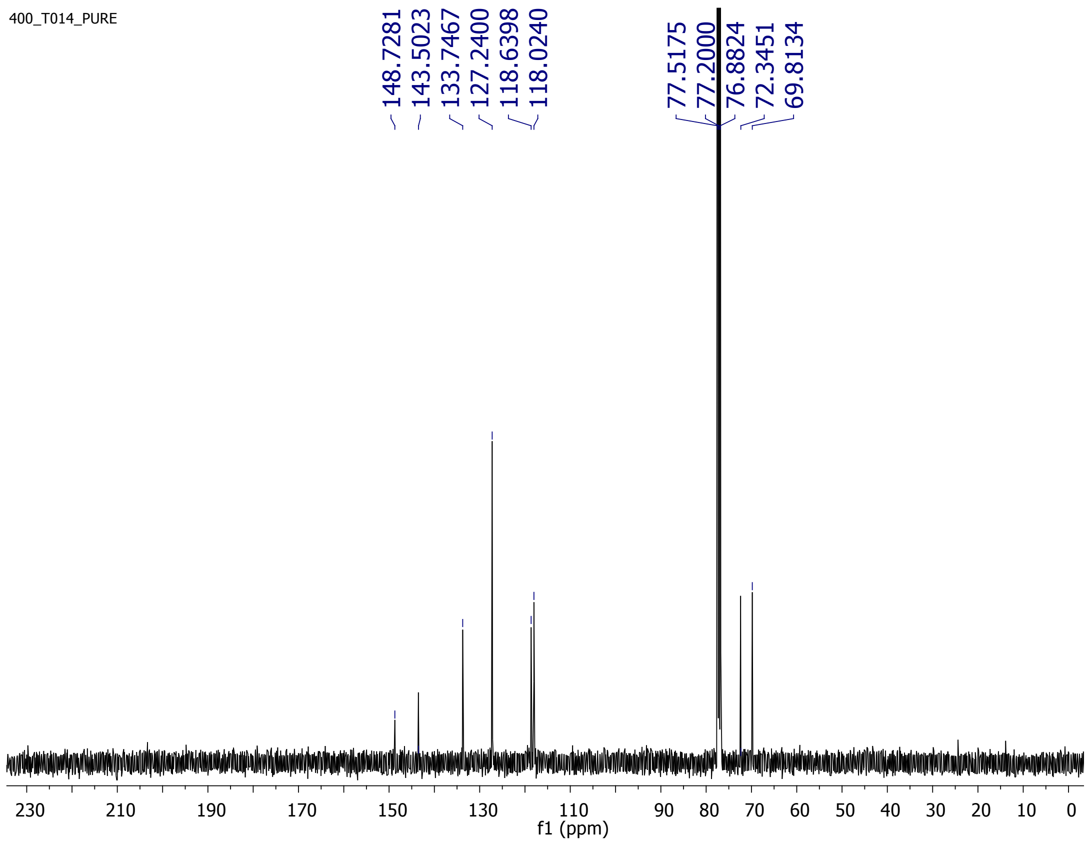

^{1}H NMR of 1-((Allyloxy)methyl)-2-methylbenzene (6ca)

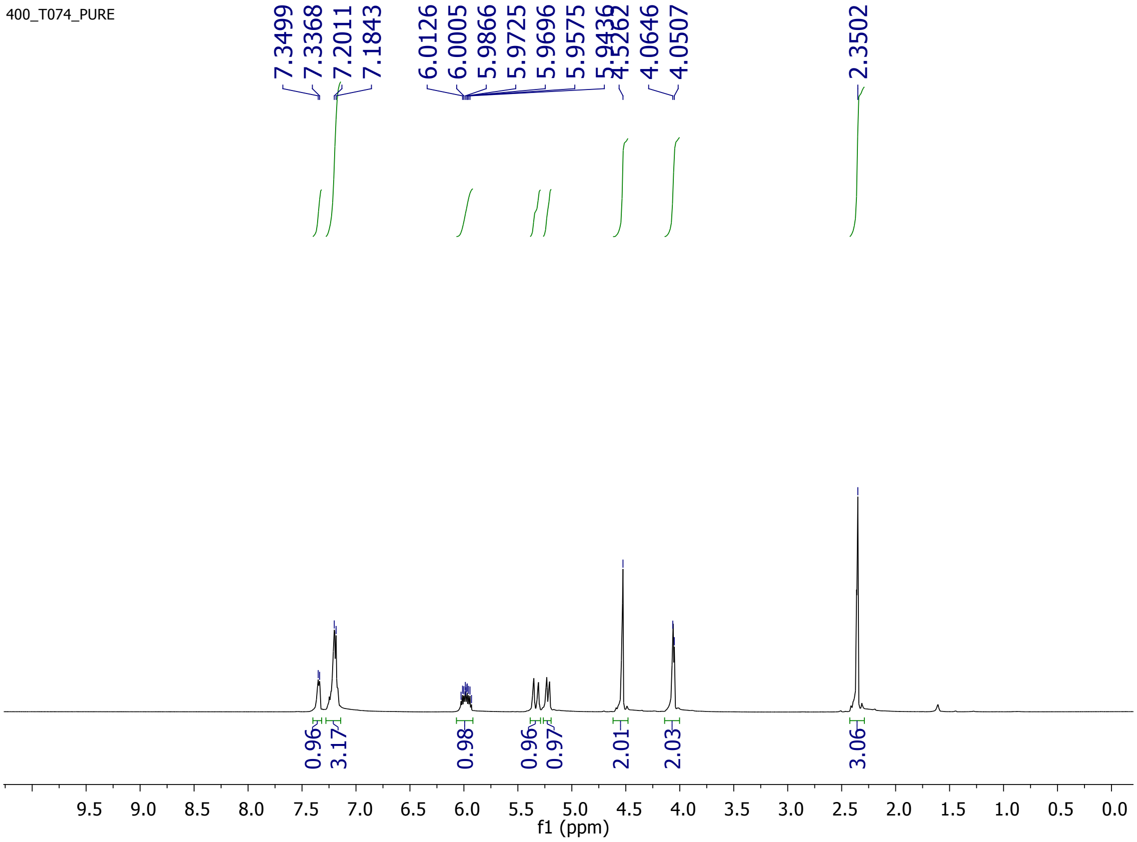

^{13}C NMR of 1-((Allyloxy)methyl)-2-methylbenzene (6ca)

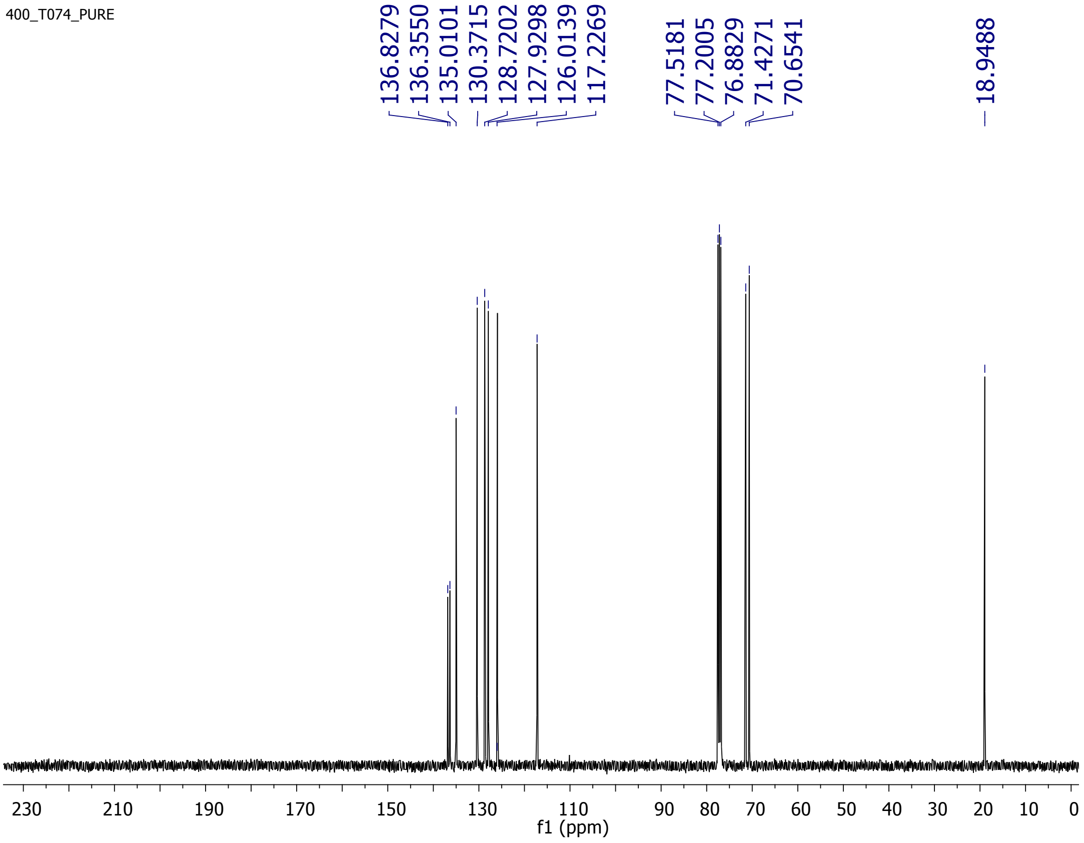

^{1}H NMR of 1-((Allyloxy)methyl)-2-bromobenzene (6da)

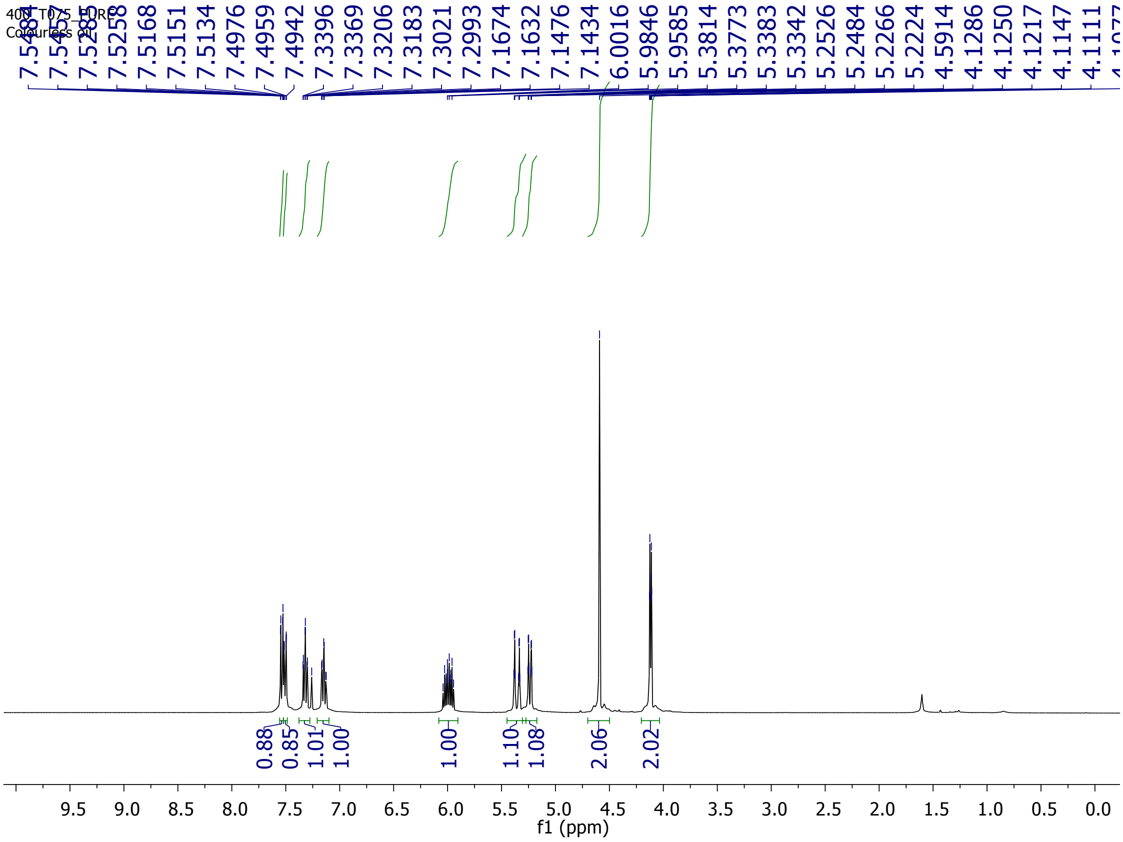

^{13}C NMR of 1-((Allyloxy)methyl)-2-bromobenzene (6da)

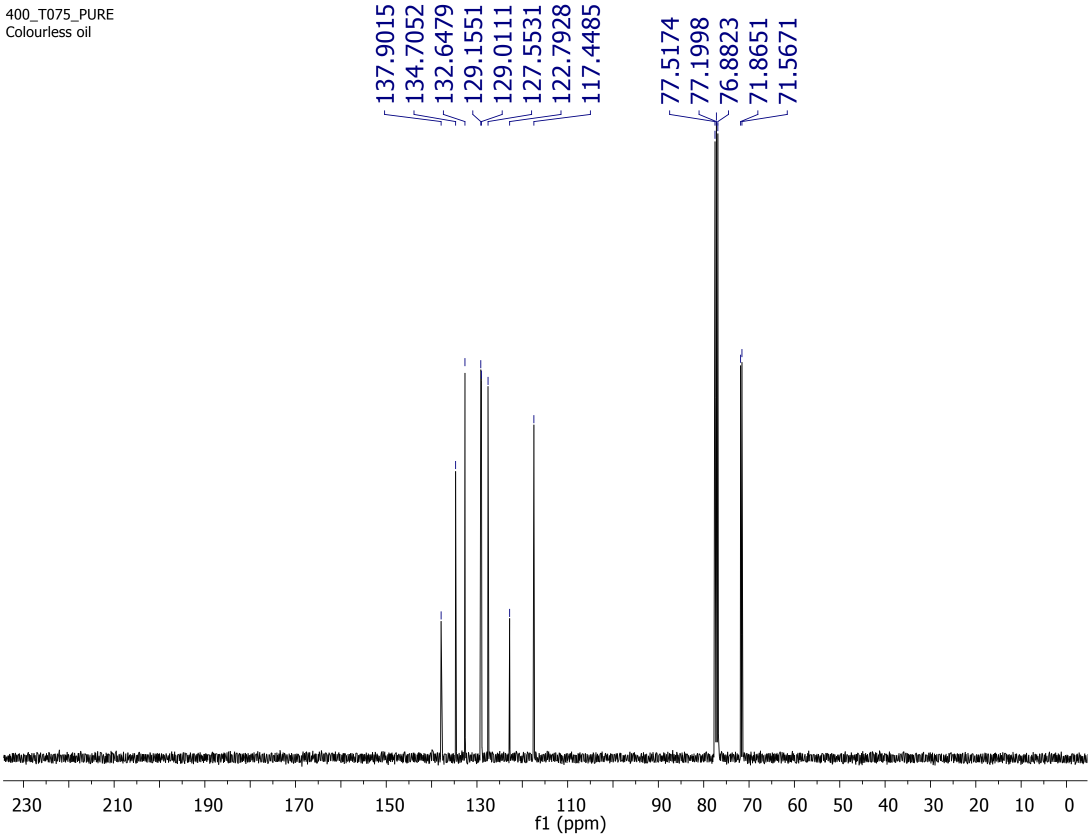

^{1}H NMR of 2-((Allyloxy)methyl)pyridine (6ea)

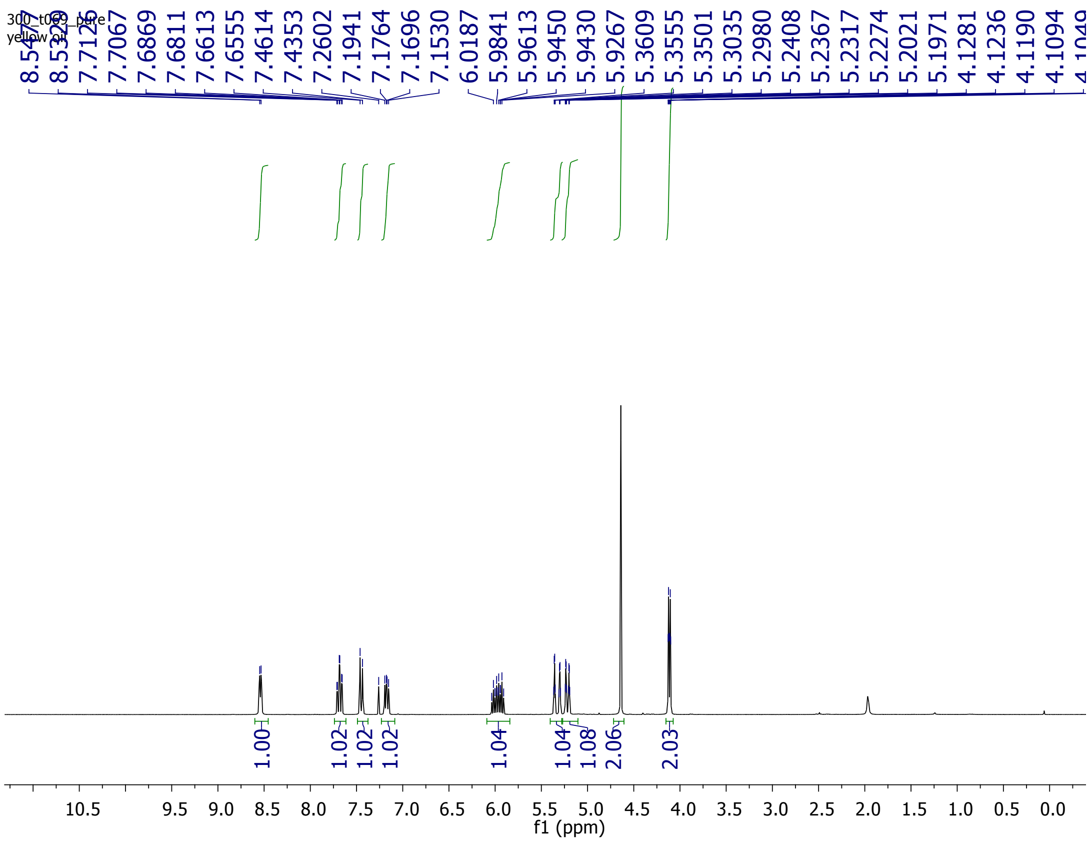

^{13}C NMR of 2-((Allyloxy)methyl)pyridine (6ea)

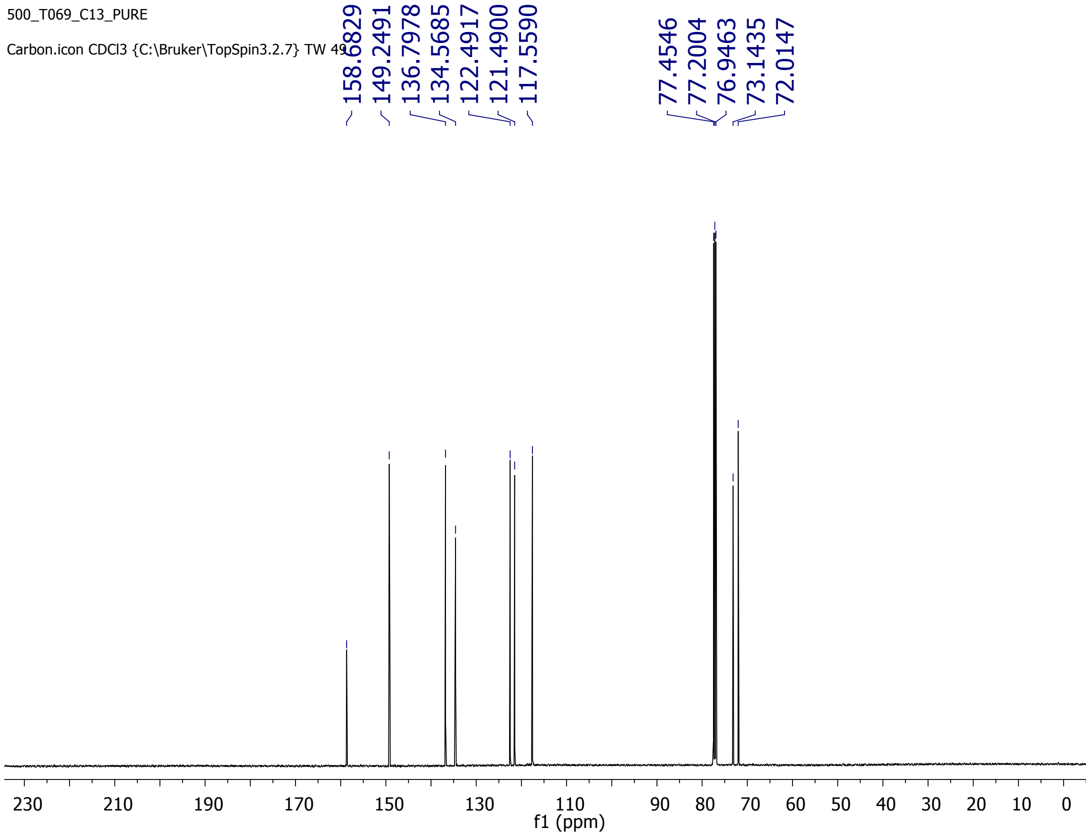

^{1}H NMR of ((2-Methylallyloxy)methyl)benzene (6fa)

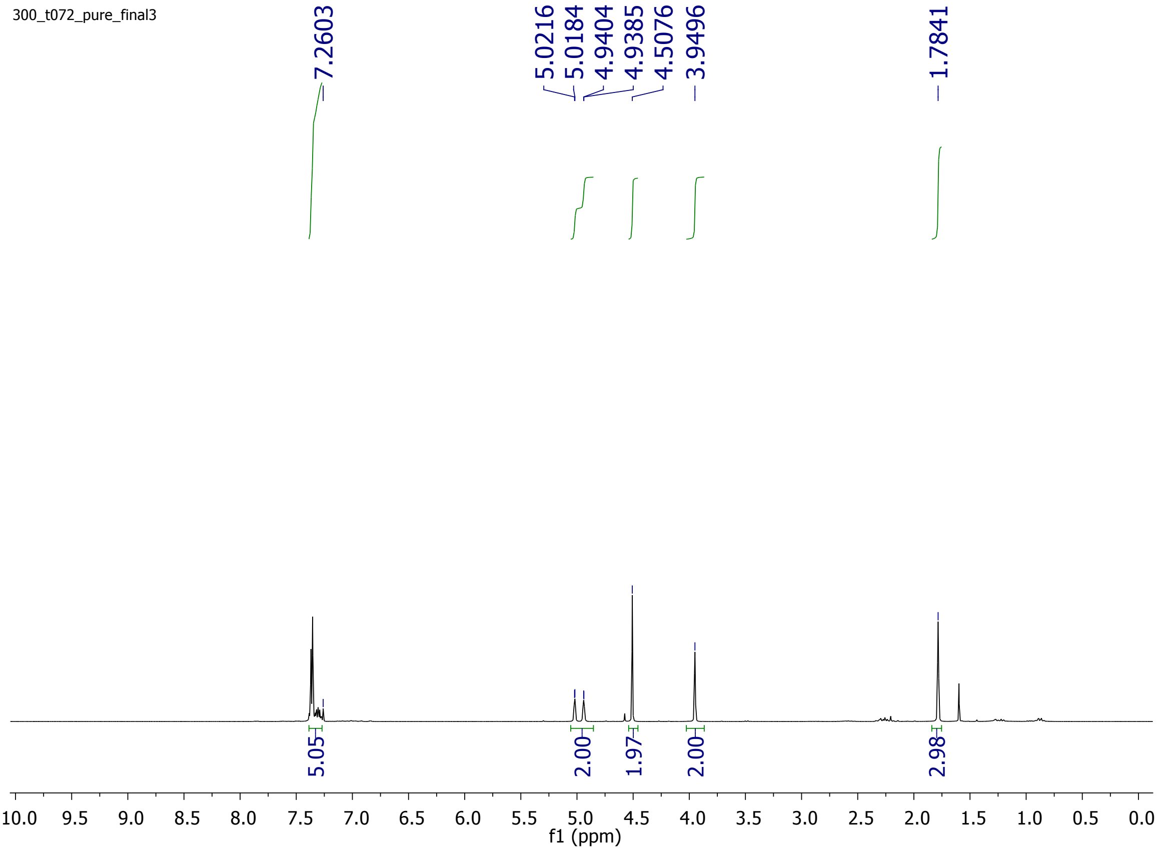

^{13}C NMR of ((2-Methylallyloxy)methyl)benzene (6fa)

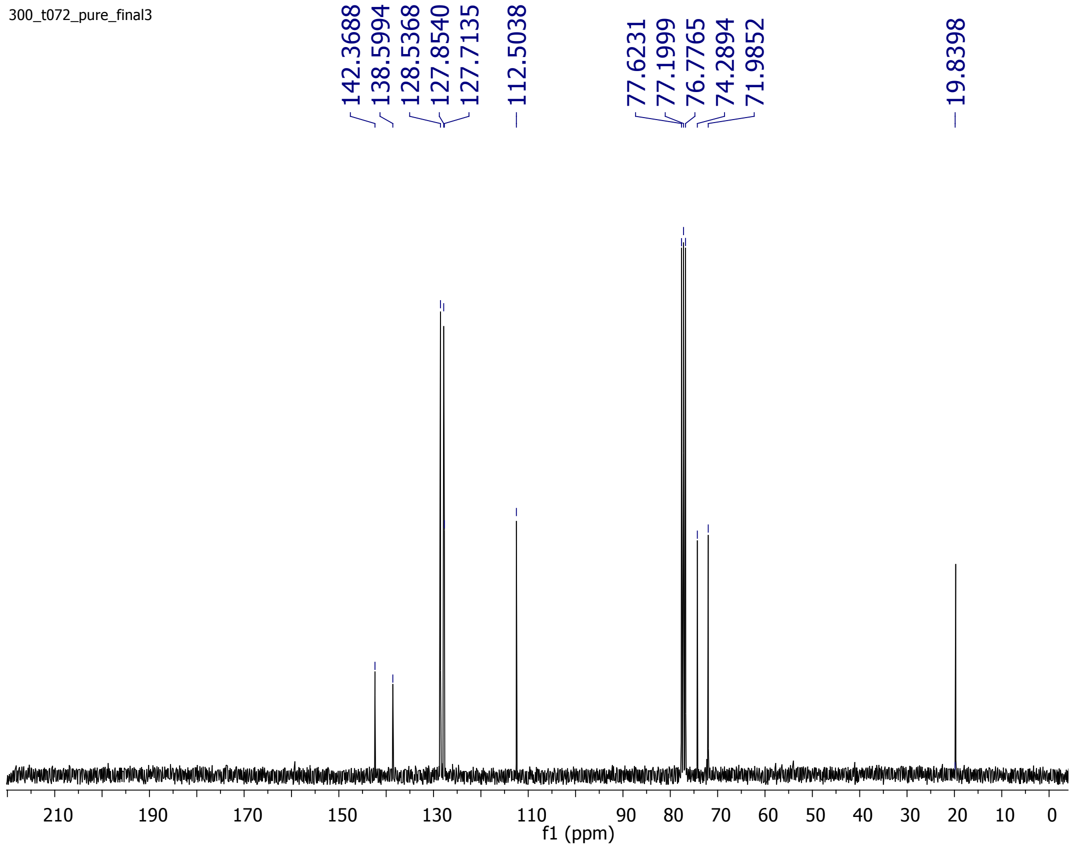

^{1}H NMR of 1-(((2-Methylallyl)oxy)methyl)-4-(trifluoromethyl)benzene (6ga)

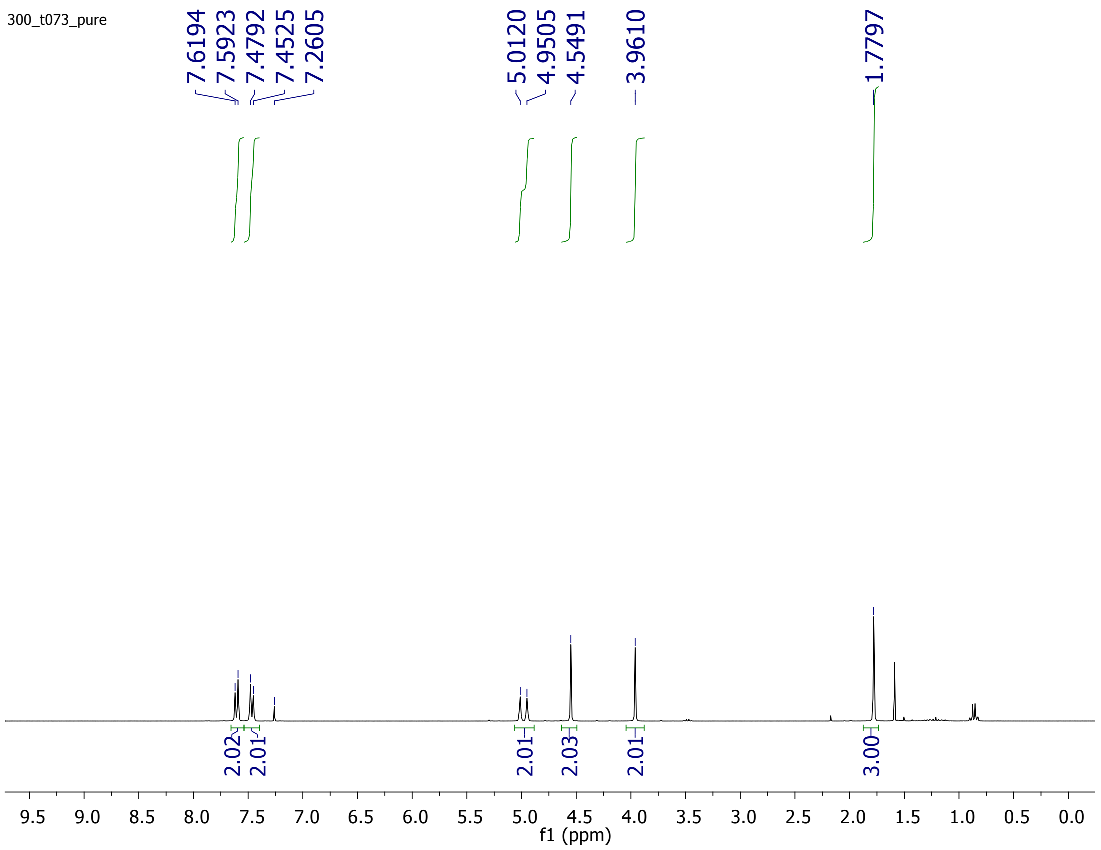

^{13}C NMR of 1-(((2-Methylallyl)oxy)methyl)-4-(trifluoromethyl)benzene (6ga)

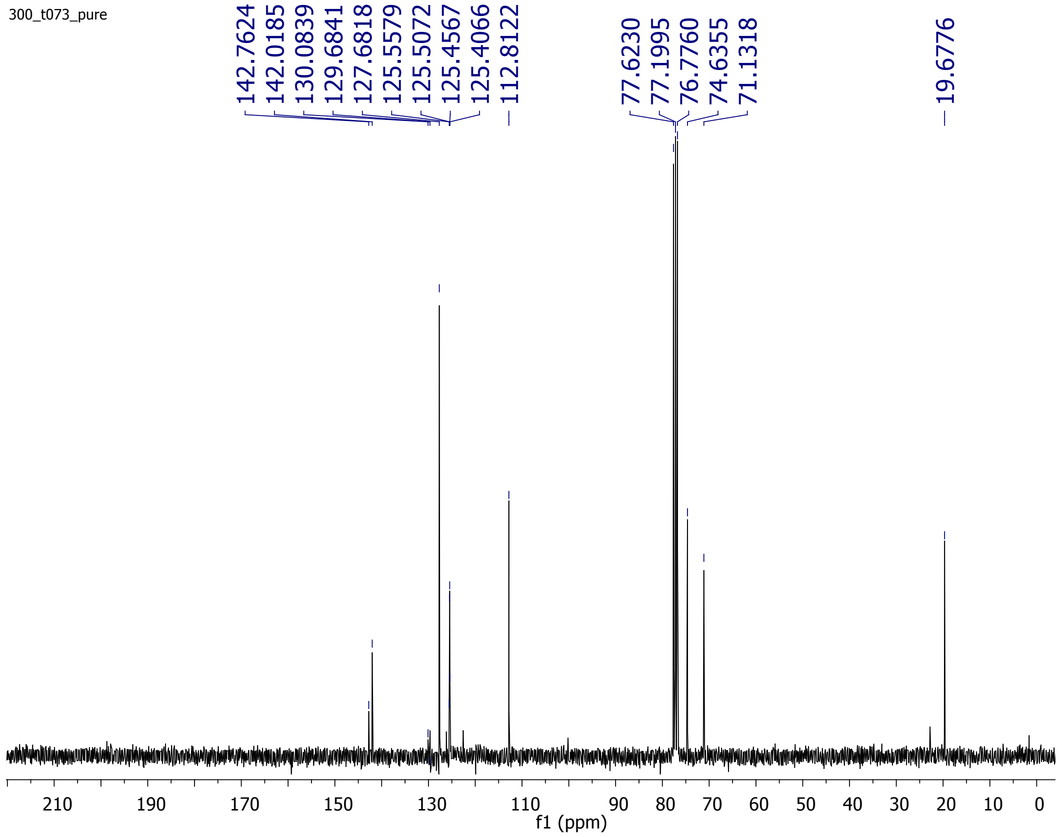

^{1}H NMR of Ethyl 1-oxo-2,3-dihydro-1H-indene-2-carboxylate (7ca)

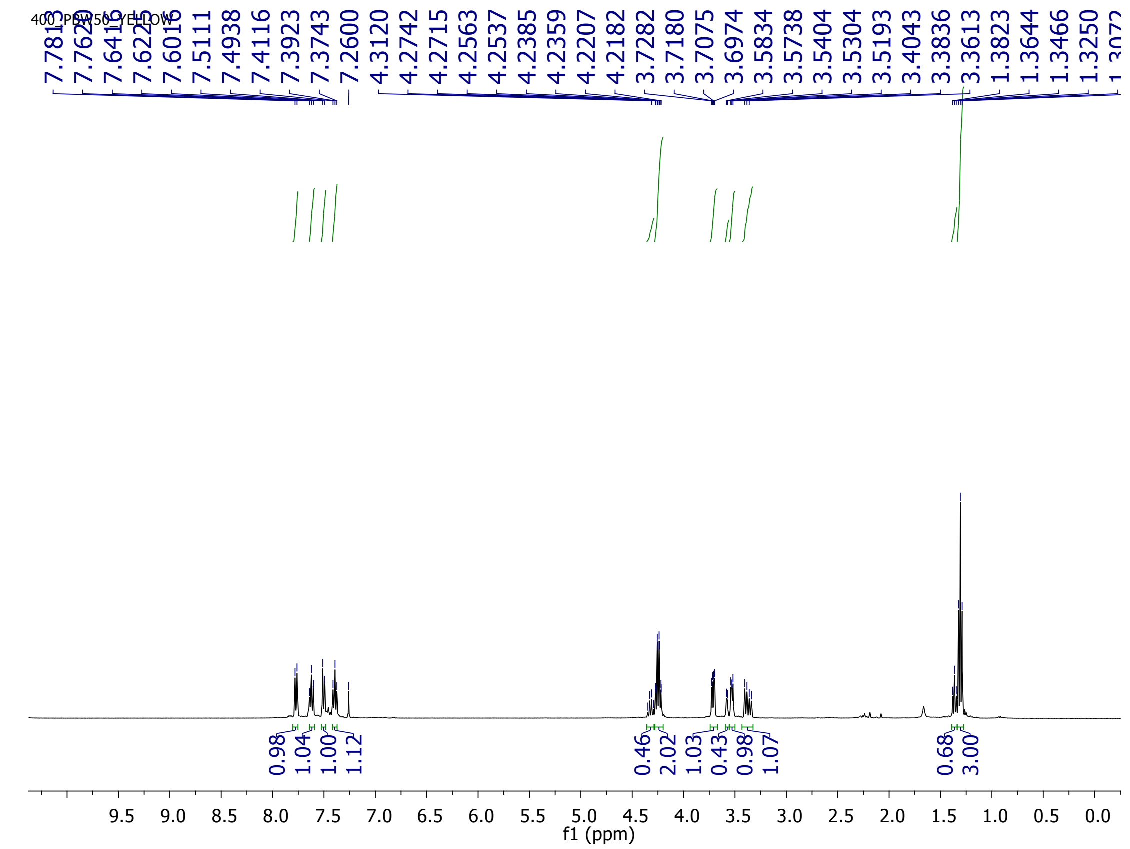

^{13}C NMR of Ethyl 1-oxo-2,3-dihydro-1H-indene-2-carboxylate (7ca)

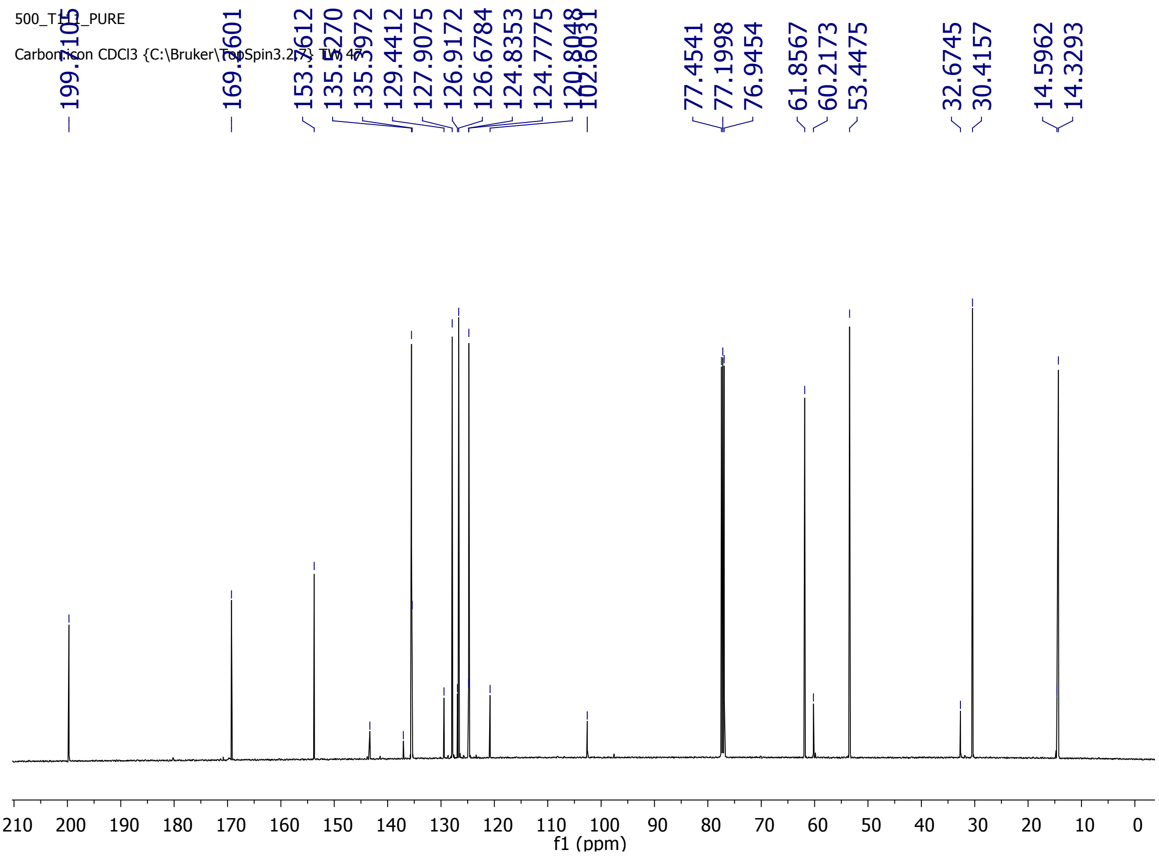

^{1}H NMR of Methyl 1-oxo-2,3-dihydro-1H-indene-2-carboxylate (7da)

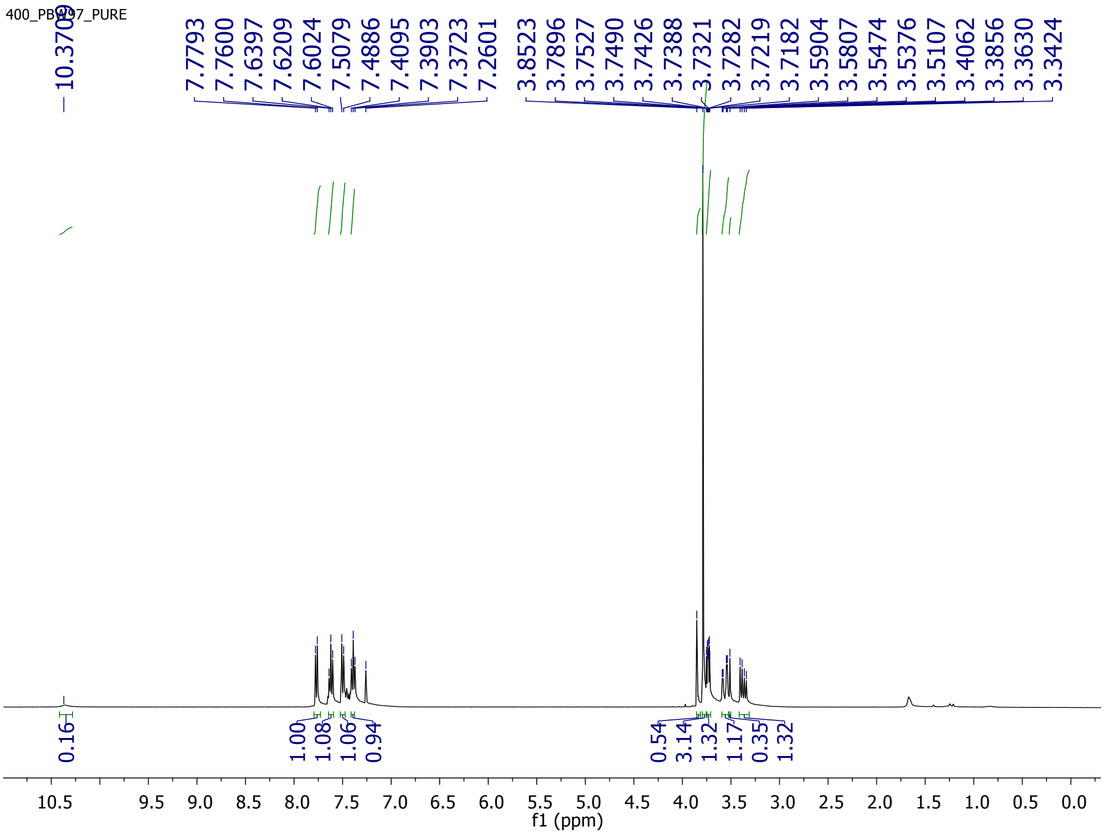

^{13}C NMR of Methyl 1-oxo-2,3-dihydro-1H-indene-2-carboxylate (7da)

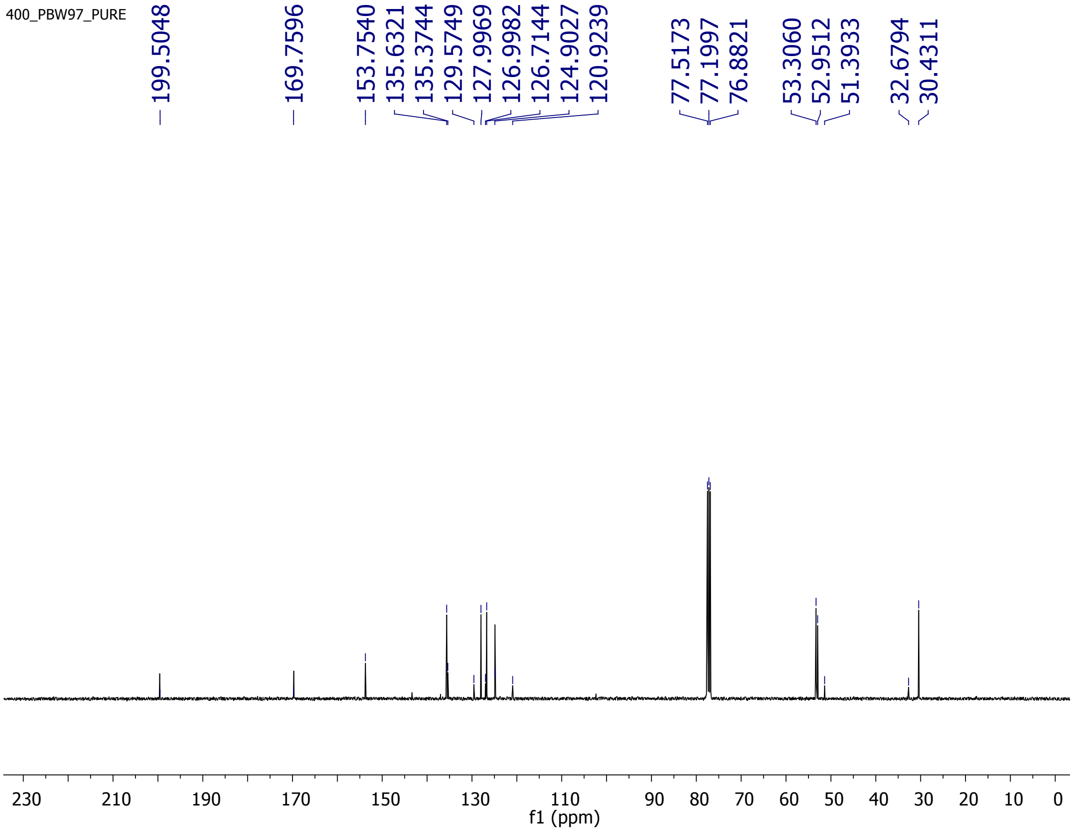

^{1}H NMR of 3-(4-Nitrophenyl)but-3-en-1-ol (9aa)

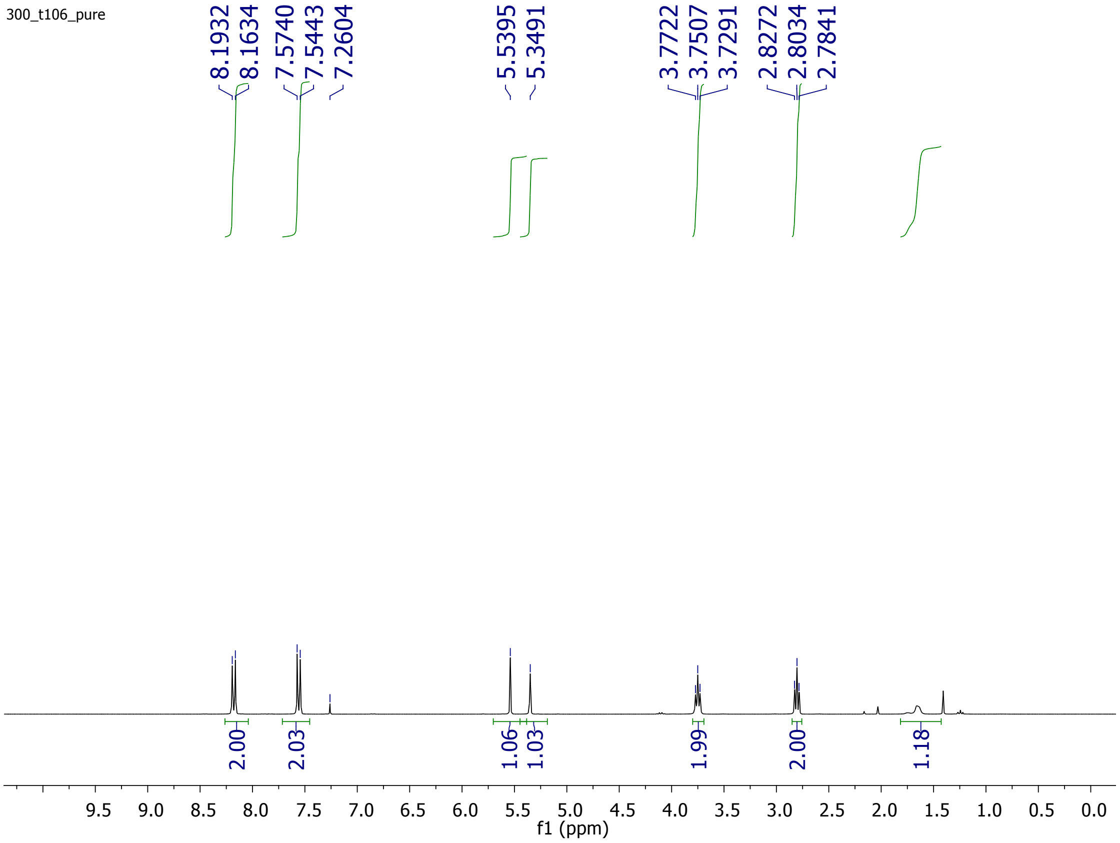

^{13}C NMR of 3-(4-Nitrophenyl)but-3-en-1-ol (9aa)

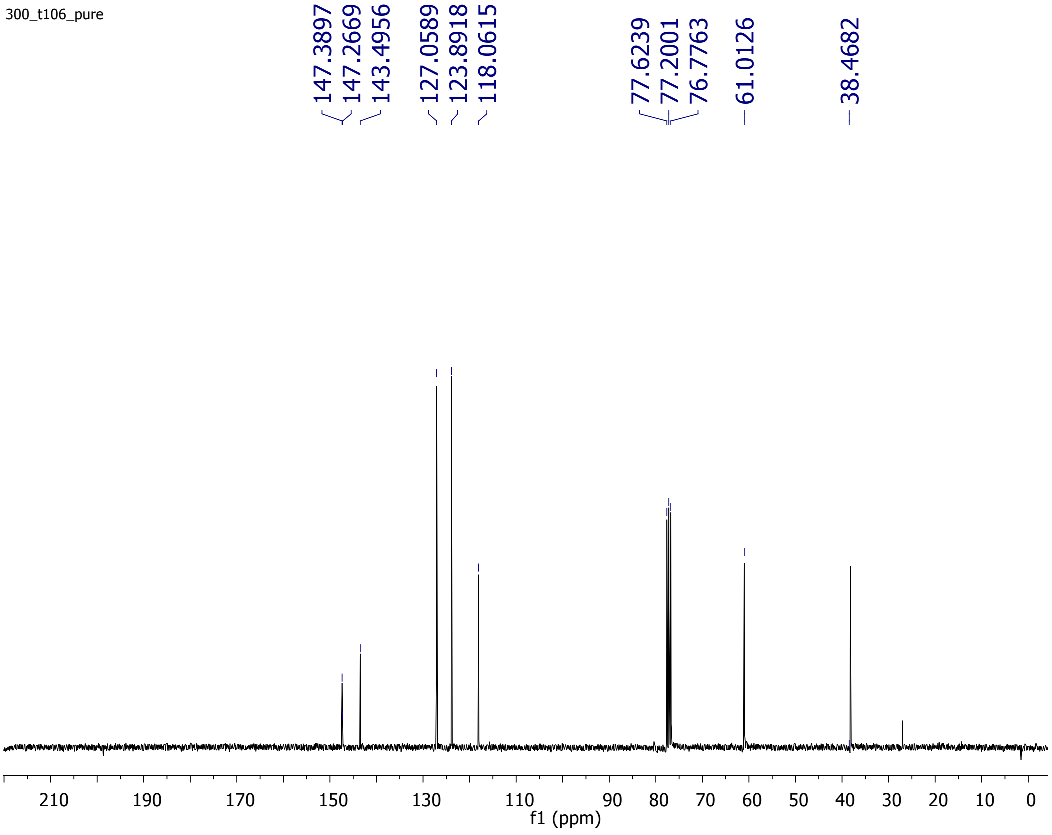

HRMS of 3-(4-Nitrophenyl)but-3-en-1-ol (9aa)

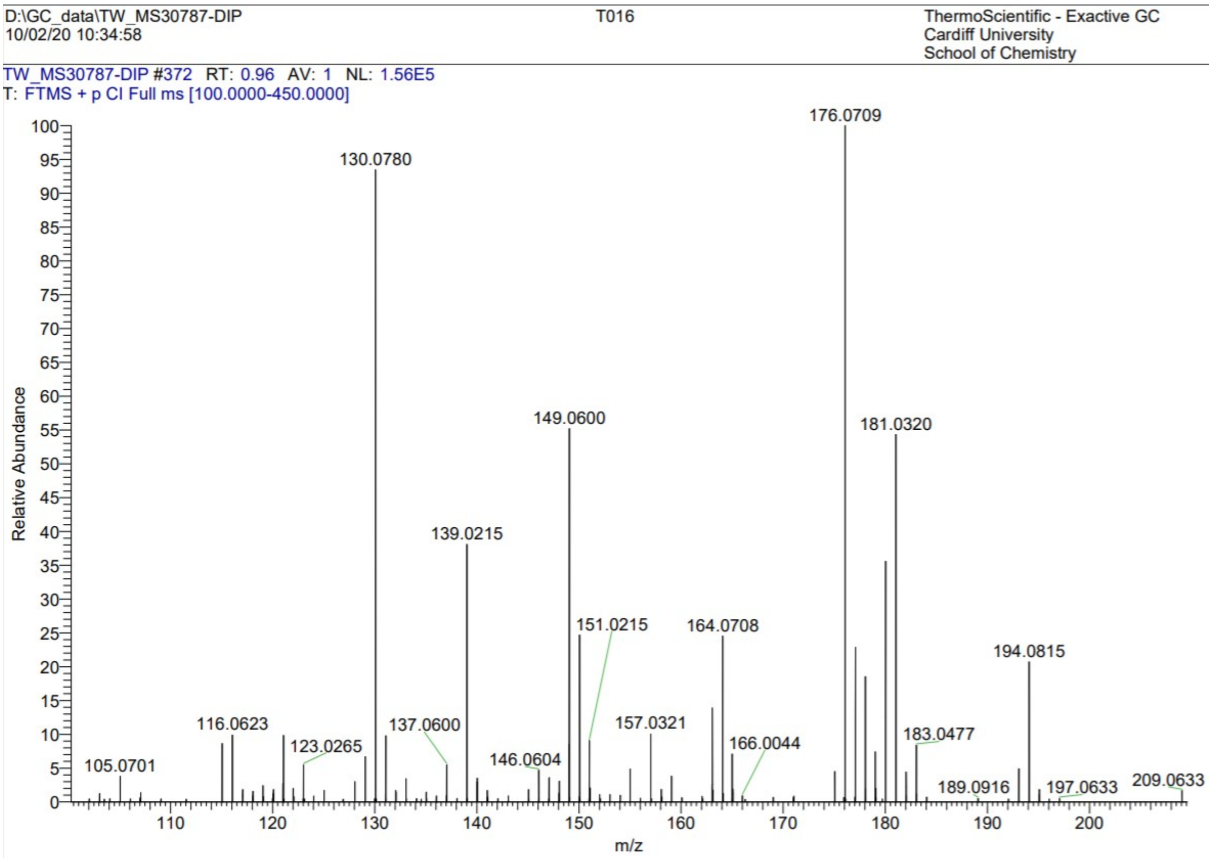

^{1}H NMR of 2-Phenylpent-4-enoic acid (10aa)

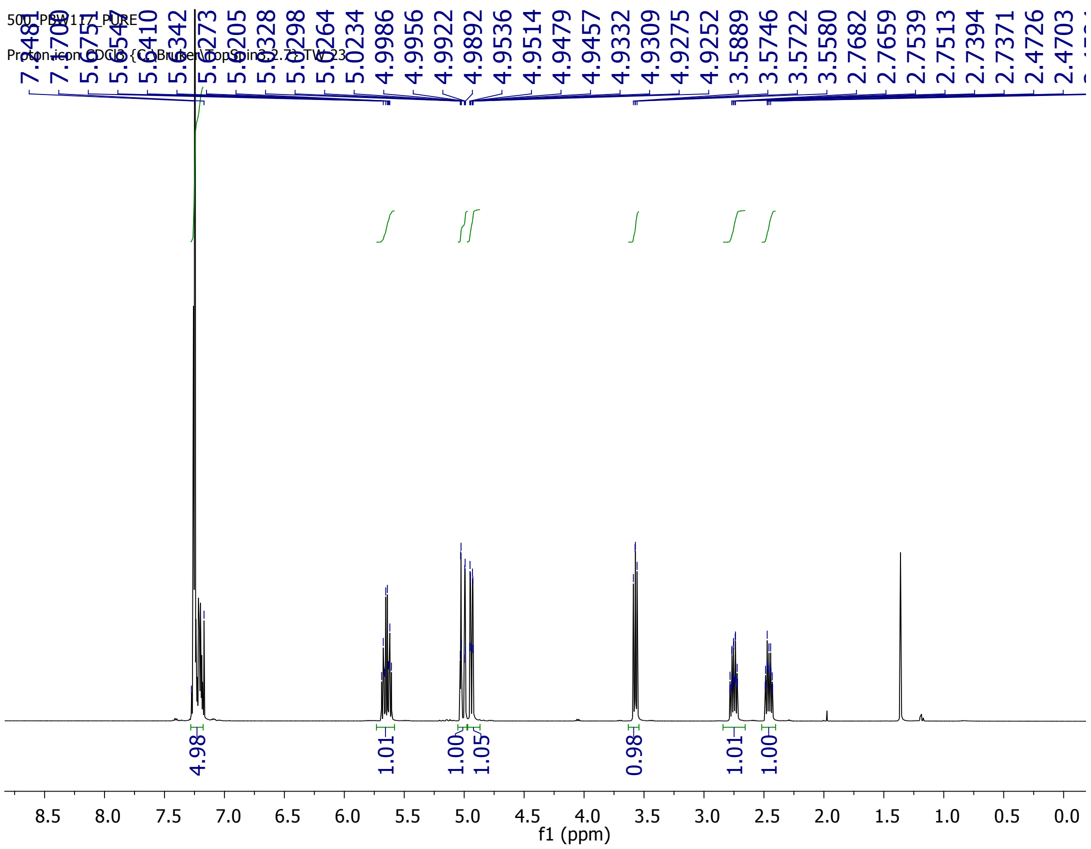

^{13}C NMR of 2-Phenylpent-4-enoic acid (10aa)

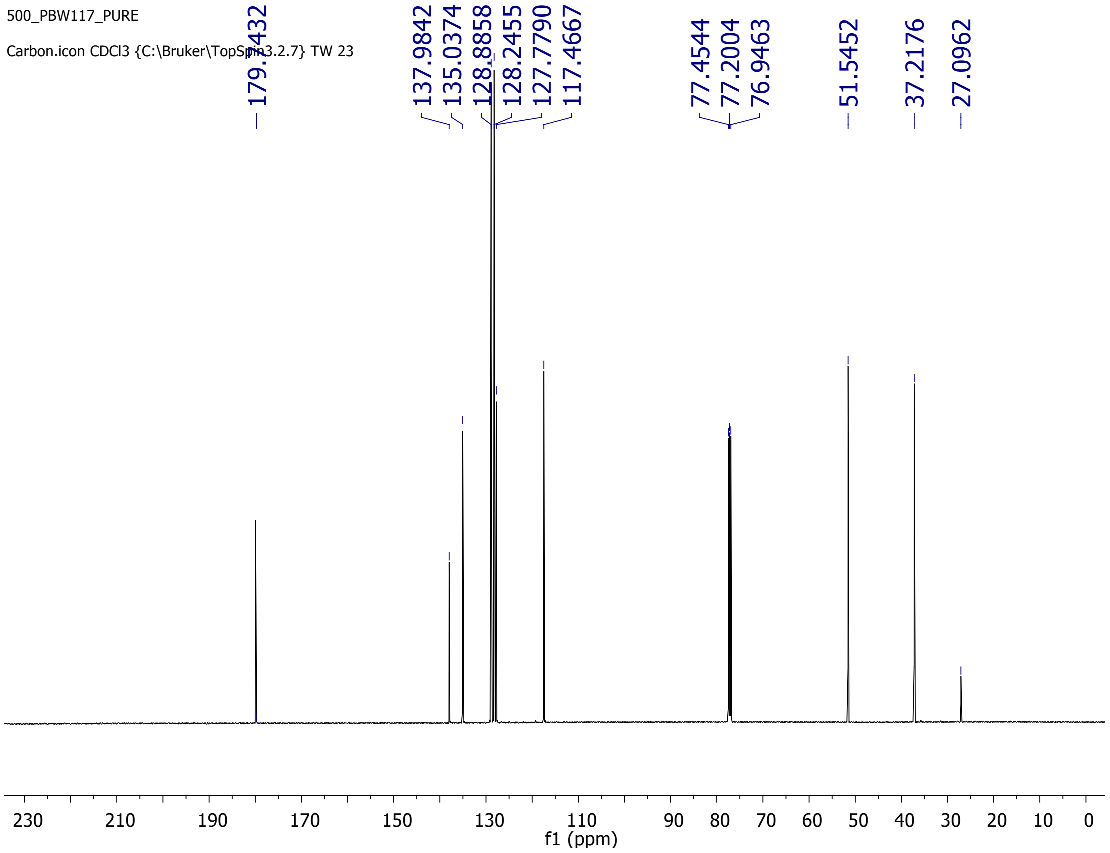

^{1}H NMR of 2-(Pent-4-en-1-yl)isoindoline-1,3-dione (14aa)

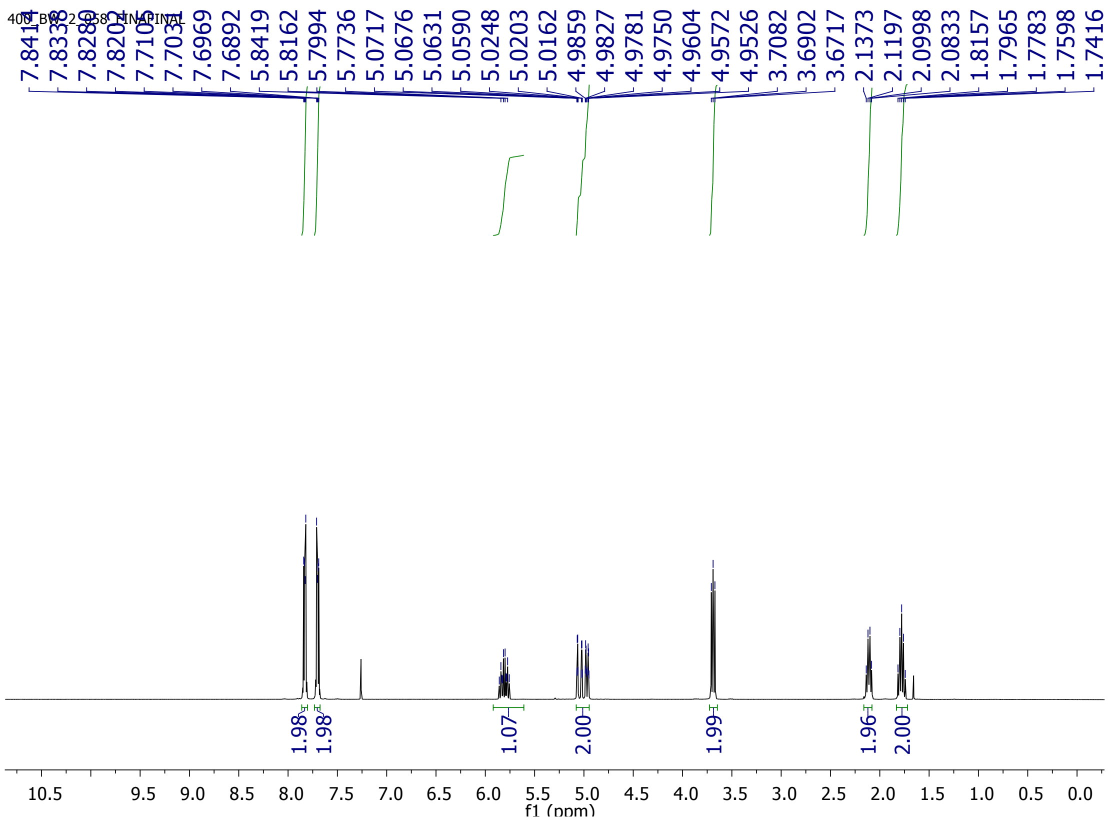

^{13}C NMR of 2-(Pent-4-en-1-yl)isoindoline-1,3-dione (14aa)

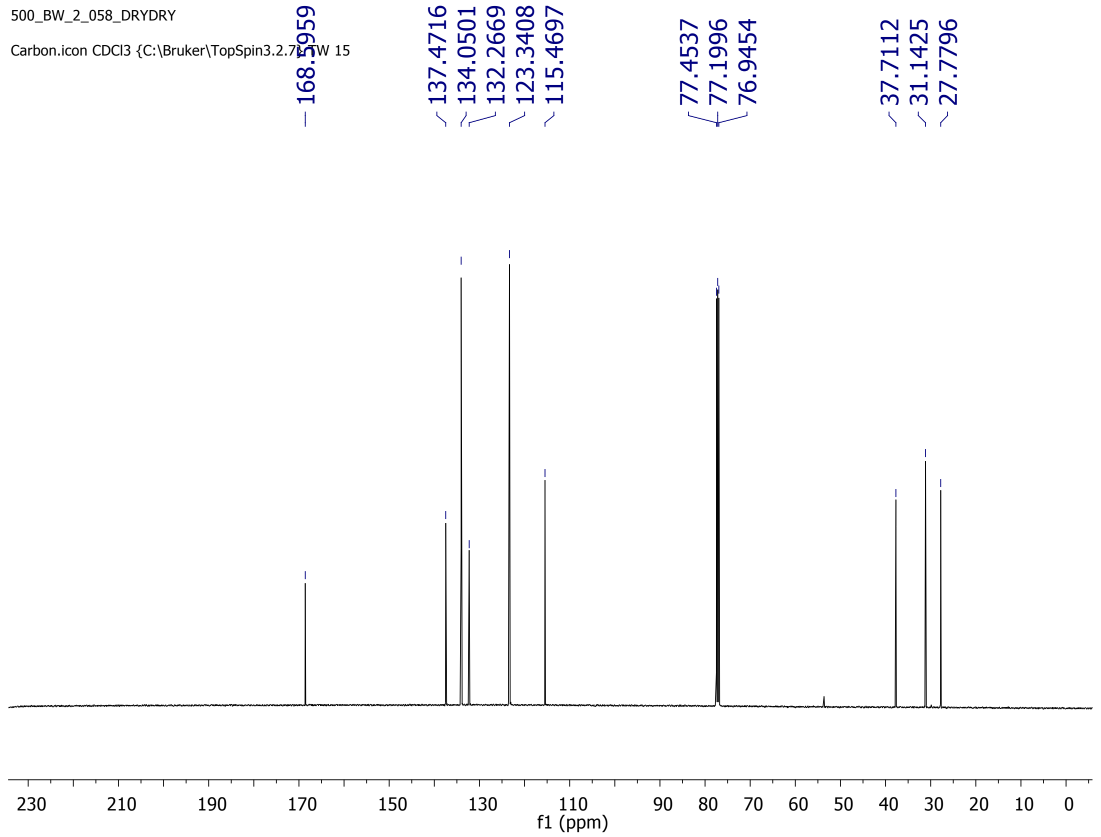

NMR Spectra for the Fluorinated Products:

^{1}H NMR of (Difluoroiodo)toluene (4b)

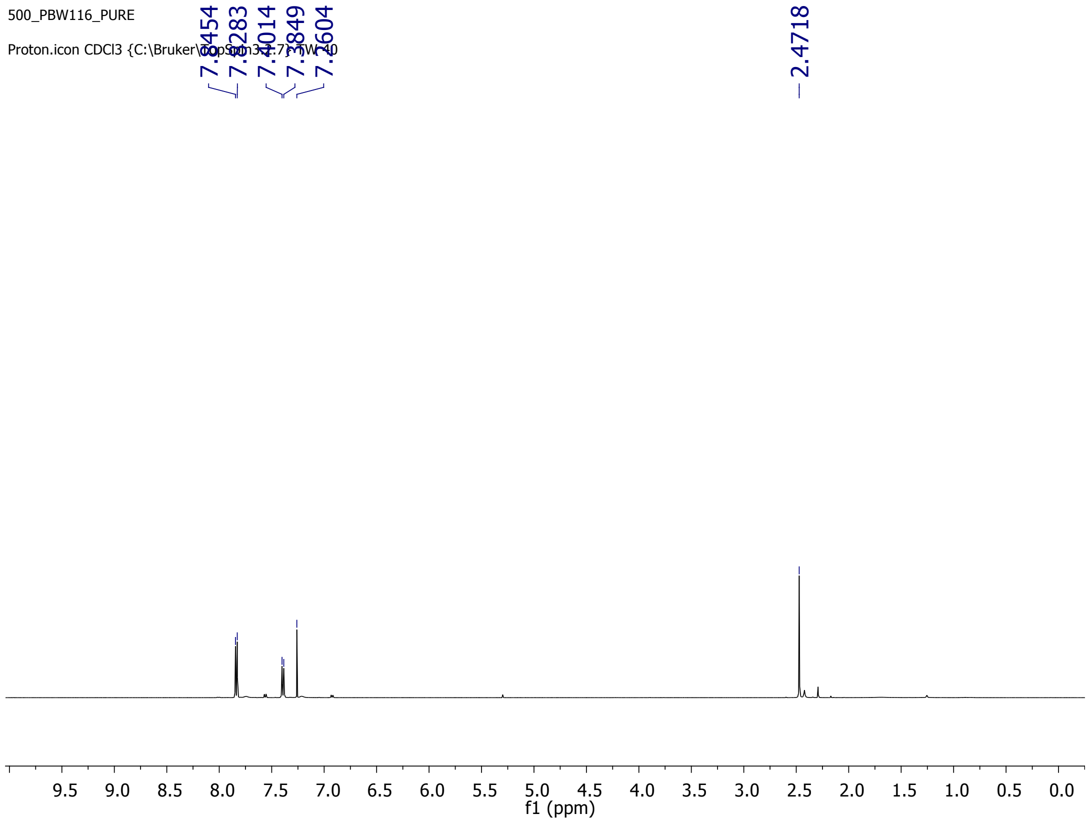

^{19}F NMR of (Difluoroiodo)toluene (4b)

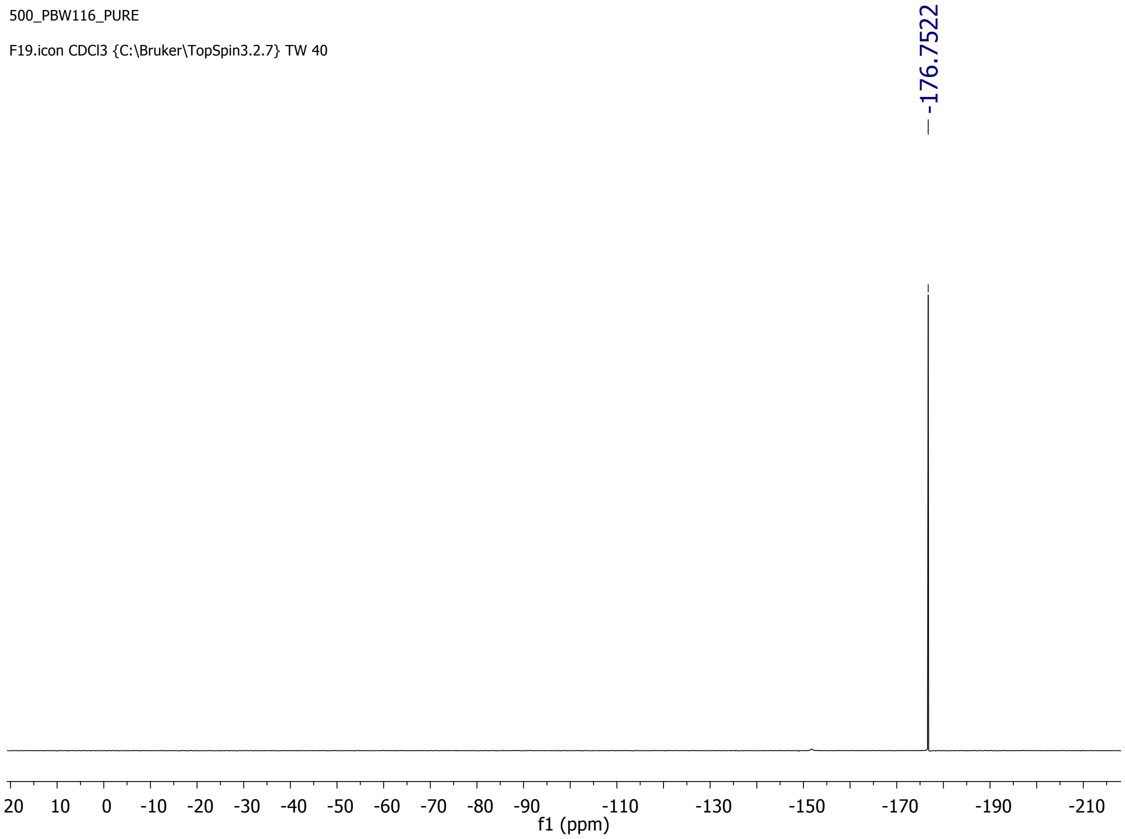

^{13}C NMR of (Difluoroiodo)toluene (4b)

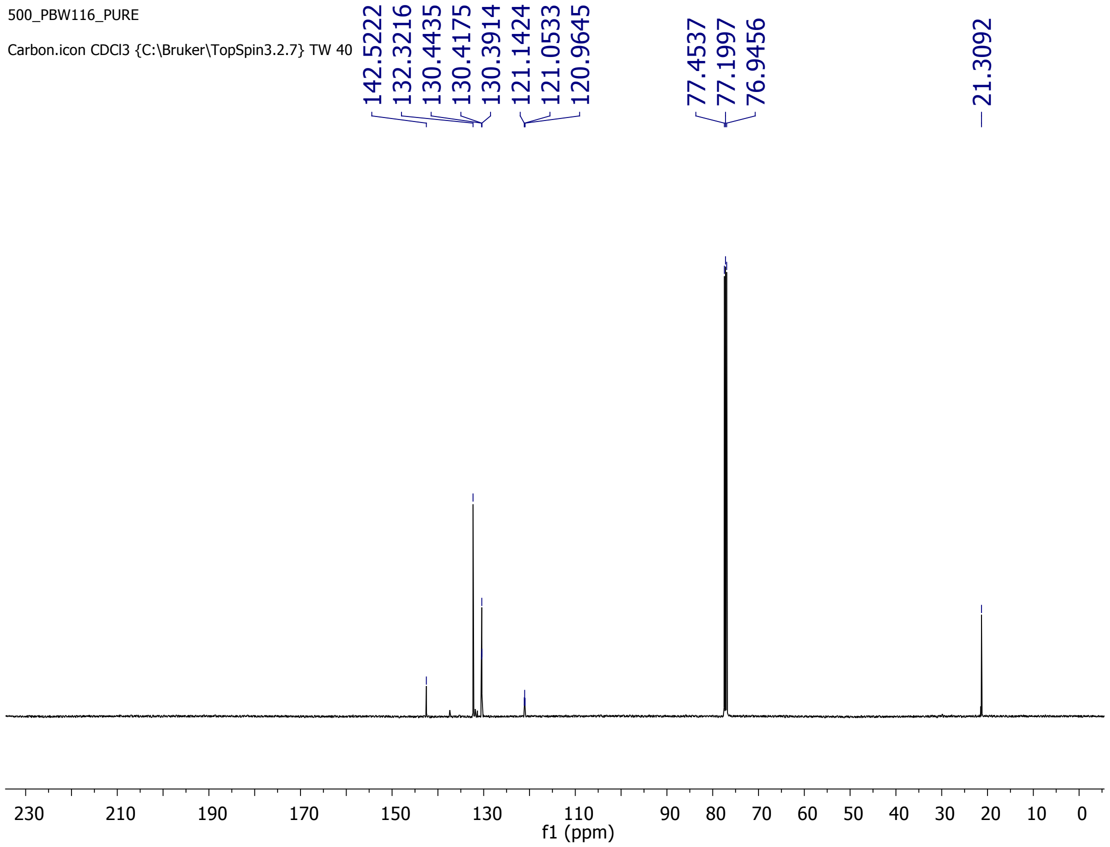

^{1}H NMR of Difluoro(*m*-tolyl)-λ^3-iodane (4f)

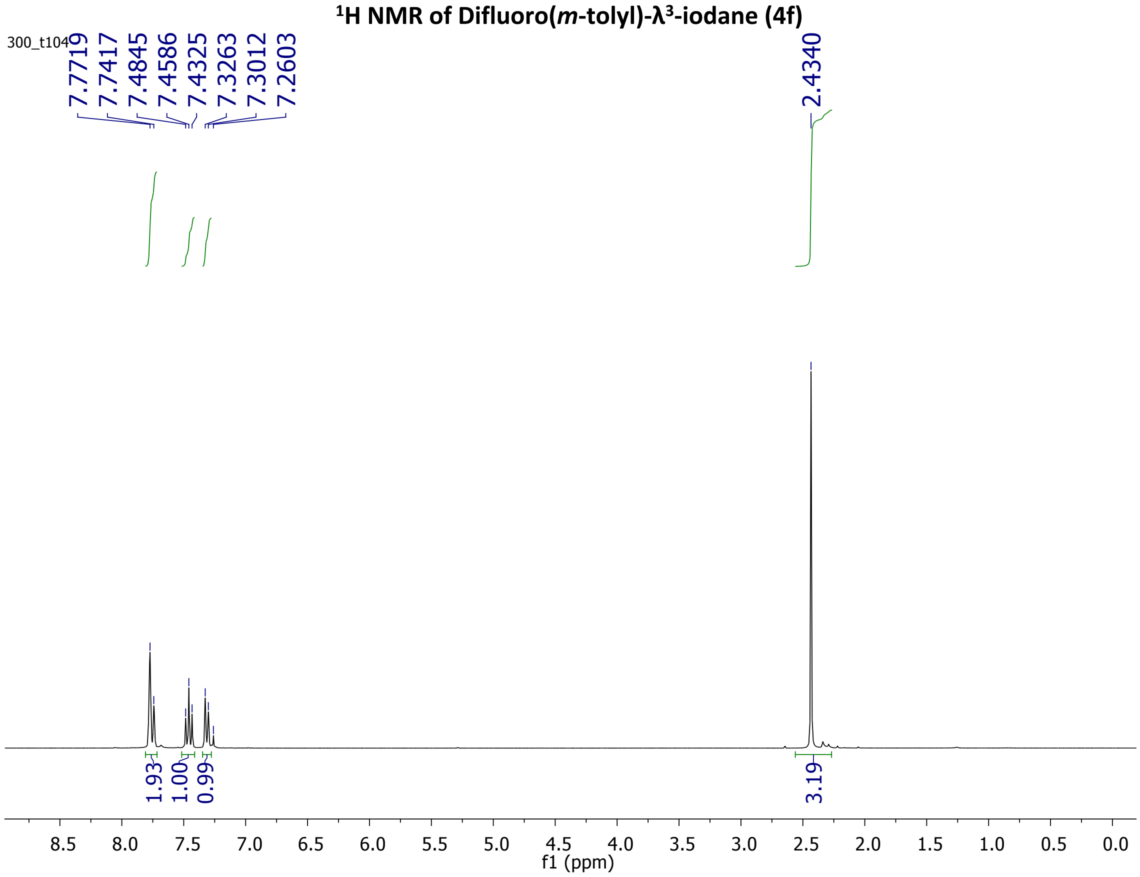

^{19}F NMR of Difluoro(*m*-tolyl)-λ^3-iodane (4f)

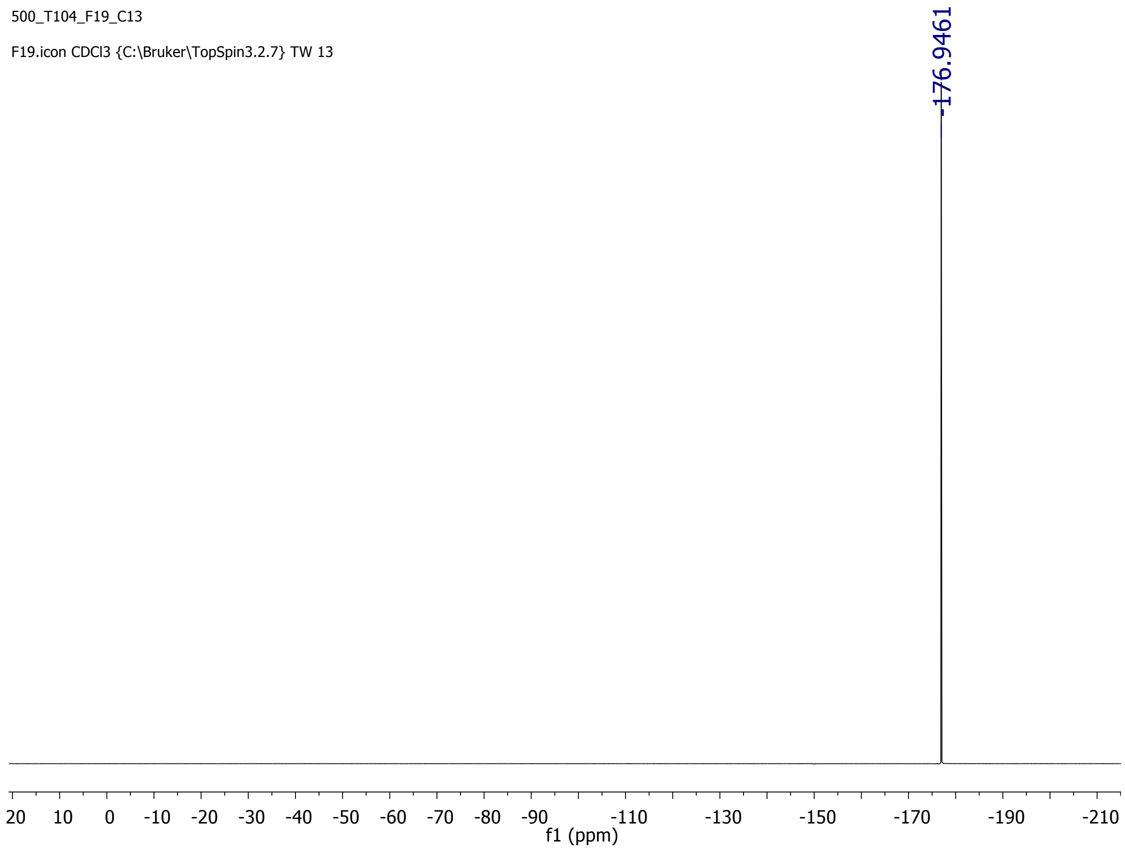

^{13}C NMR of Difluoro(*m*-tolyl)-λ^3-iodane (4f)

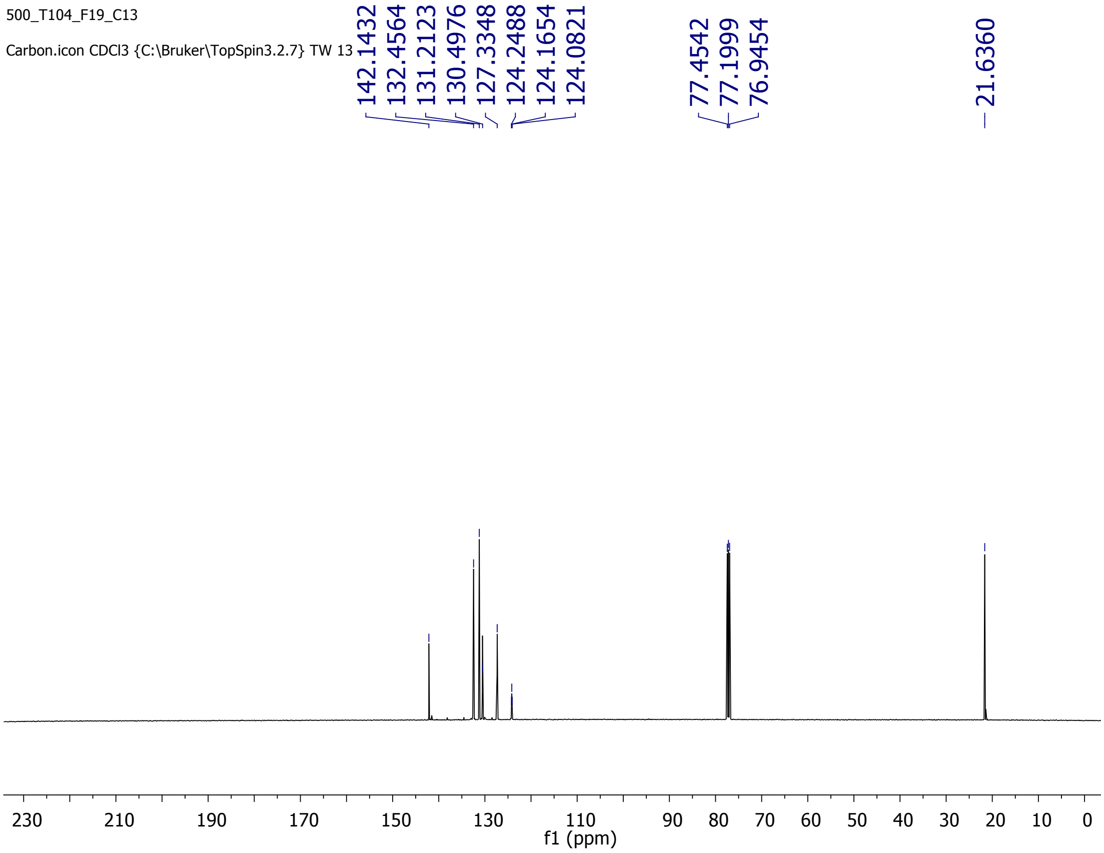

^{1}H NMR of Difluoro(3-(trifluoromethyl)phenyl)-λ^3-iodane (4g)

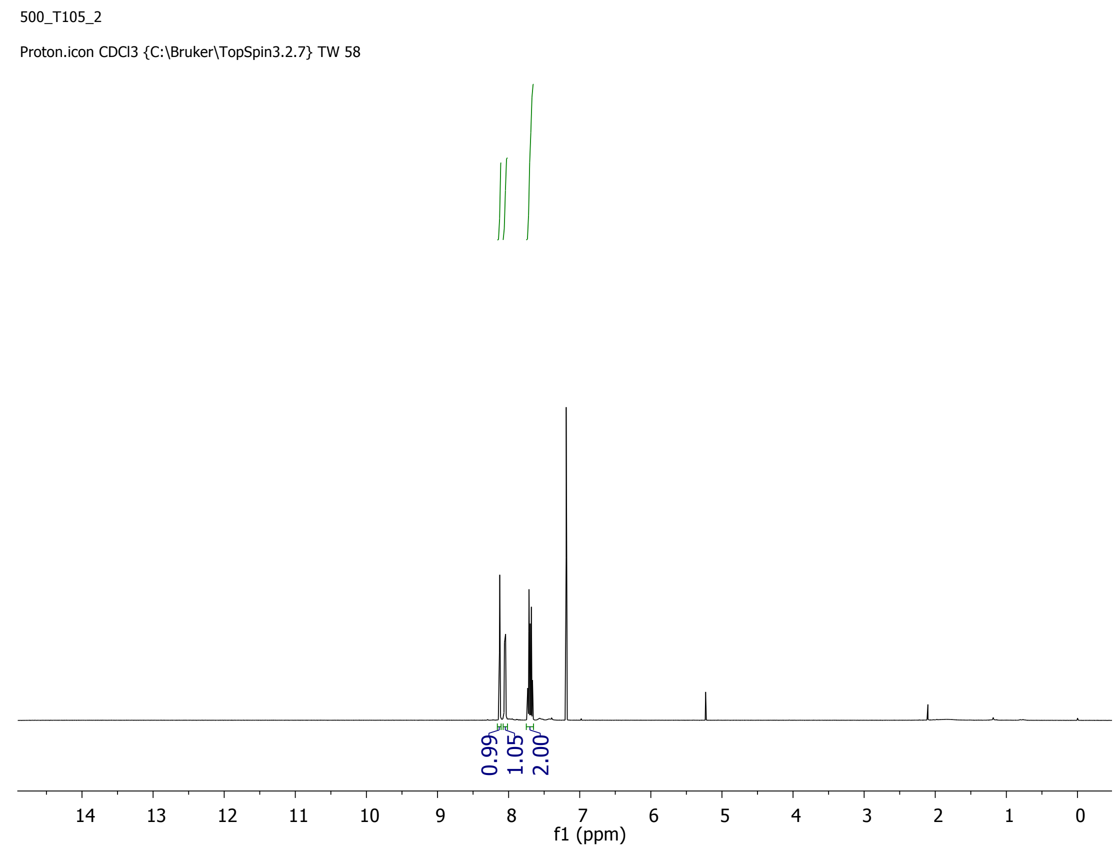

^{19}F NMR of Difluoro(3-(trifluoromethyl)phenyl)-λ^3-iodane (4g)

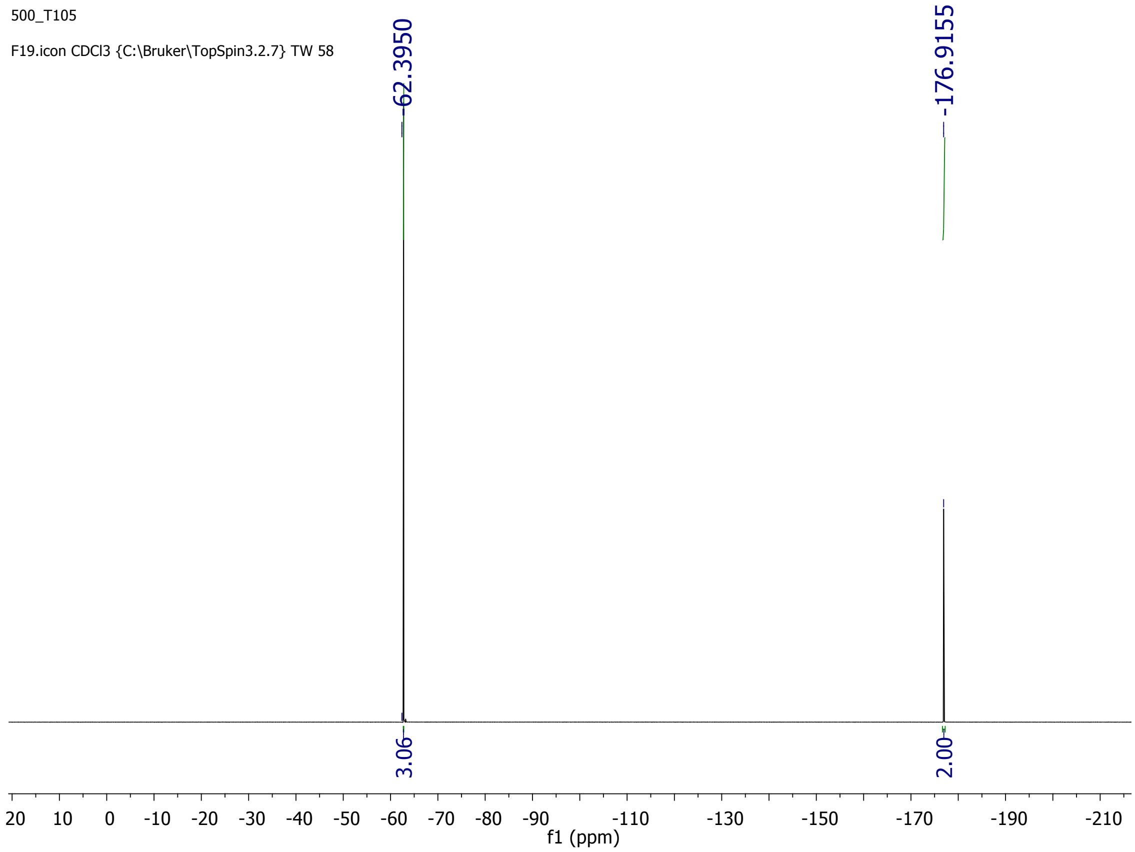

^{13}C NMR of Difluoro(3-(trifluoromethyl)phenyl)-λ^3-iodane (4g)

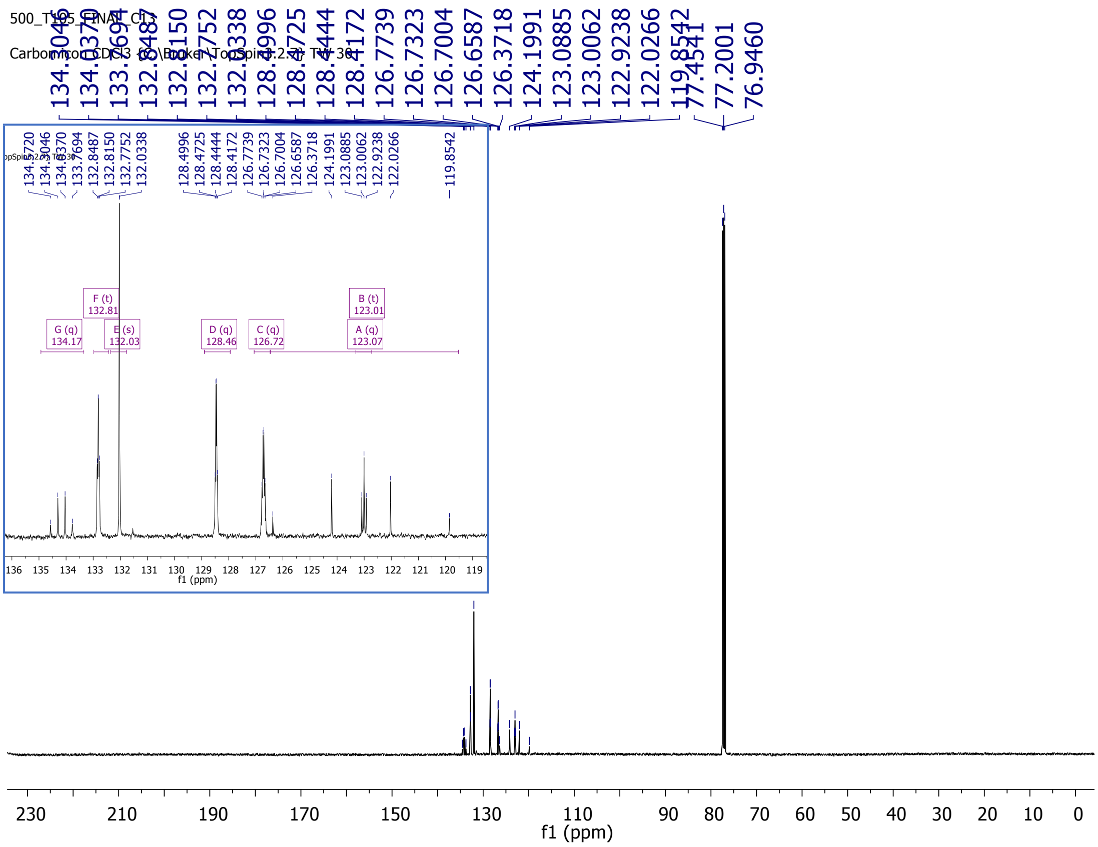

^{1}H NMR - 5-(Fluoromethyl)-2-phenyl-oxazoline (2b)

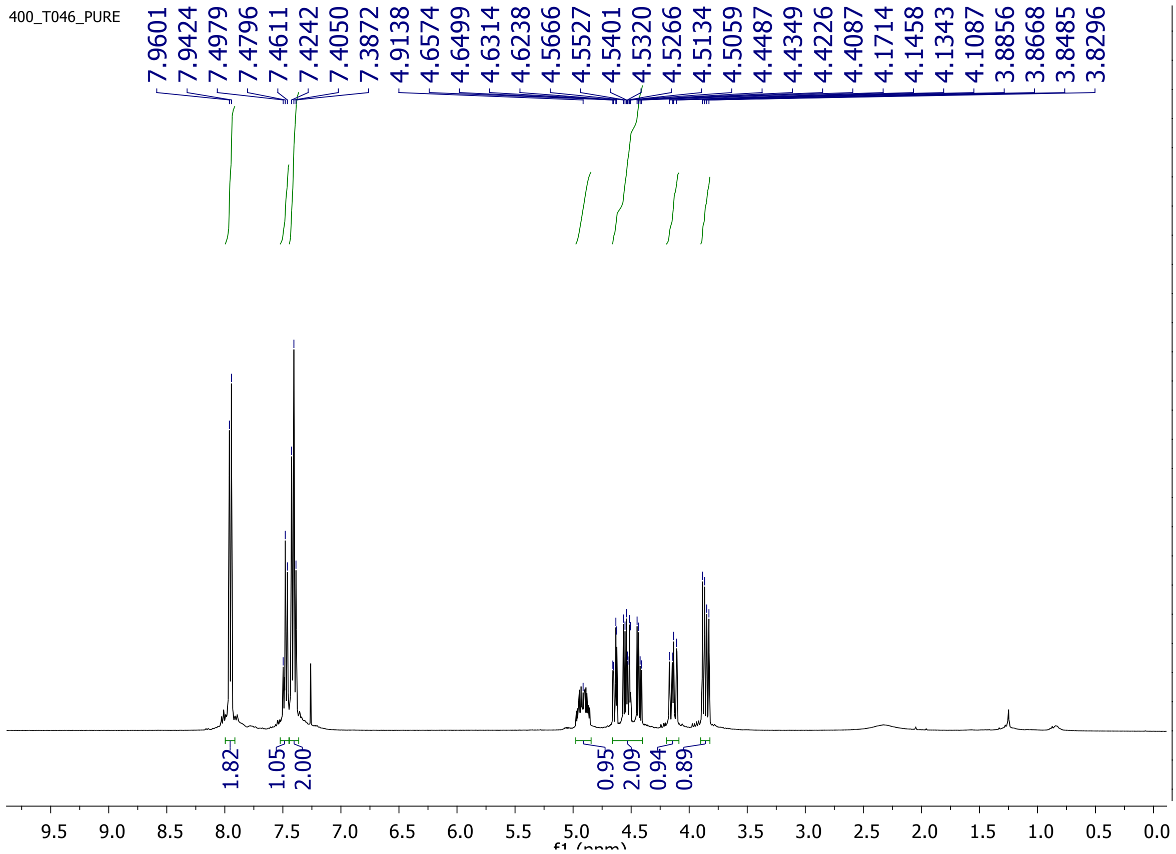

^{19}F NMR - 5-(Fluoromethyl)-2-phenyl-oxazoline (2b)

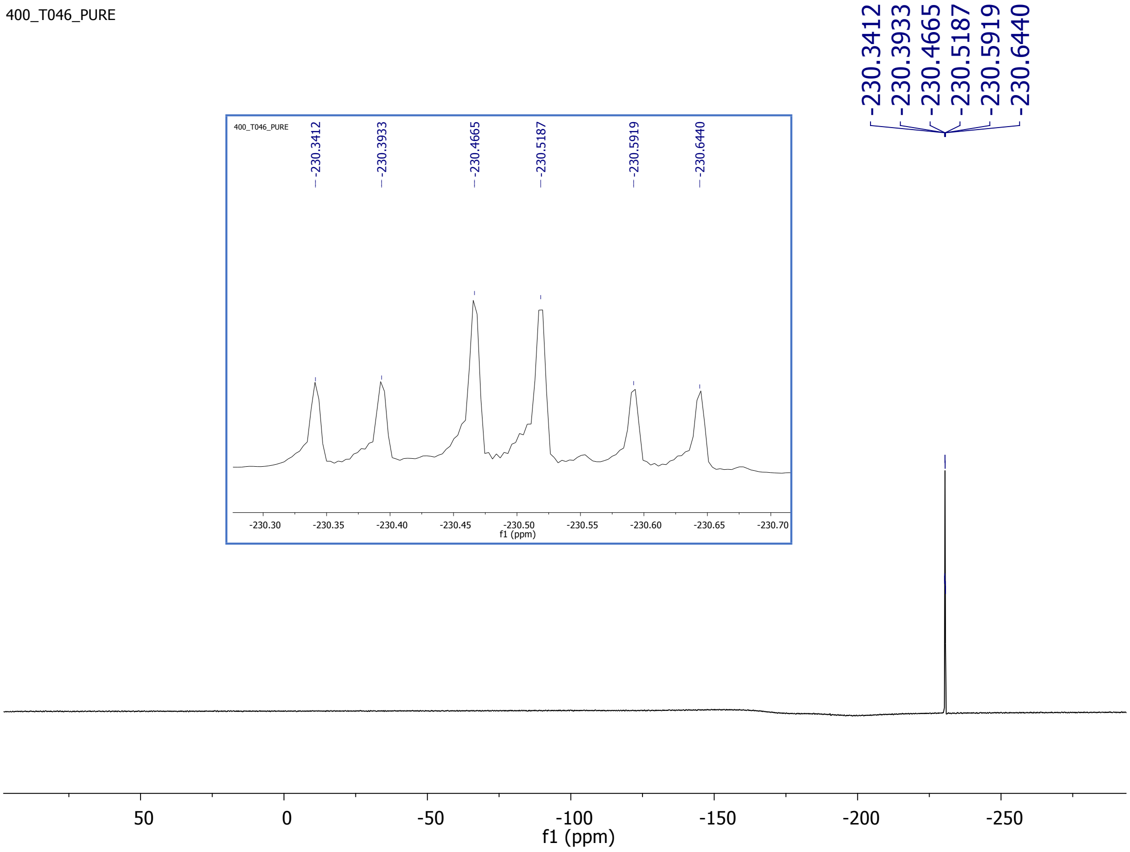

^{13}C NMR - 5-(Fluoromethyl)-2-phenyl-oxazoline (2b)

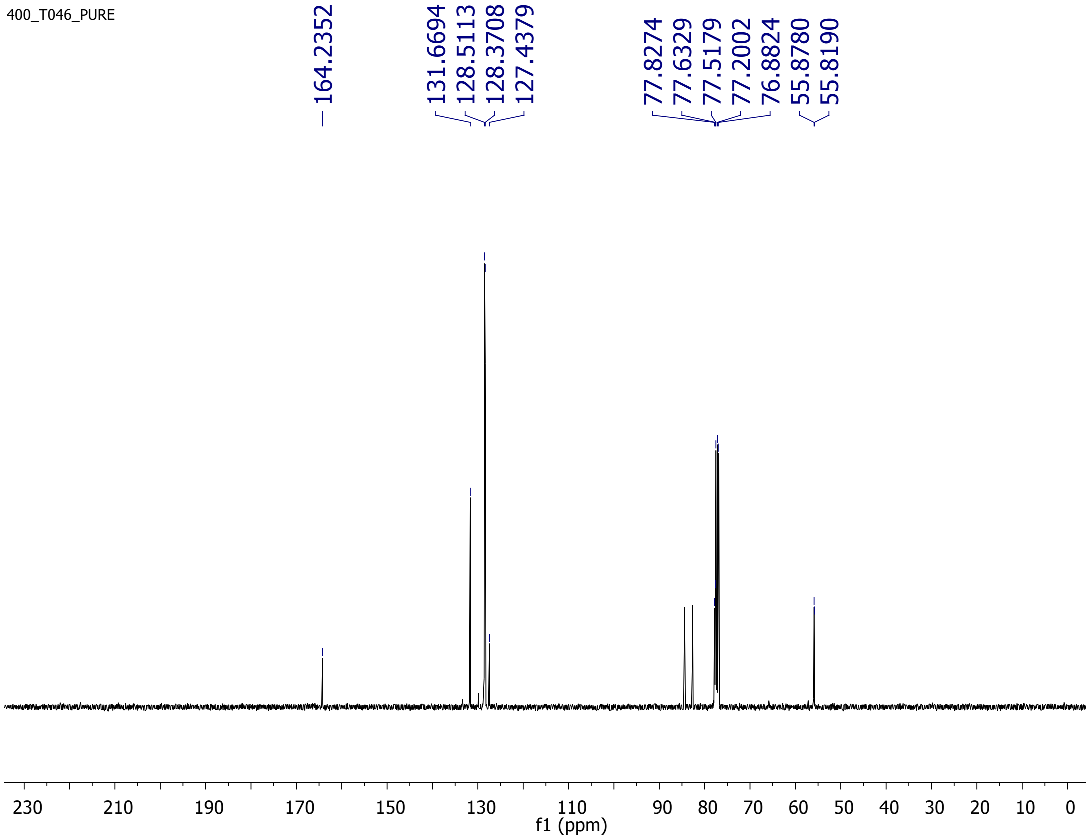

^{1}H NMR of 5-(Fluoromethyl)-2-(4-methylphenyl)-oxazoline (2a)

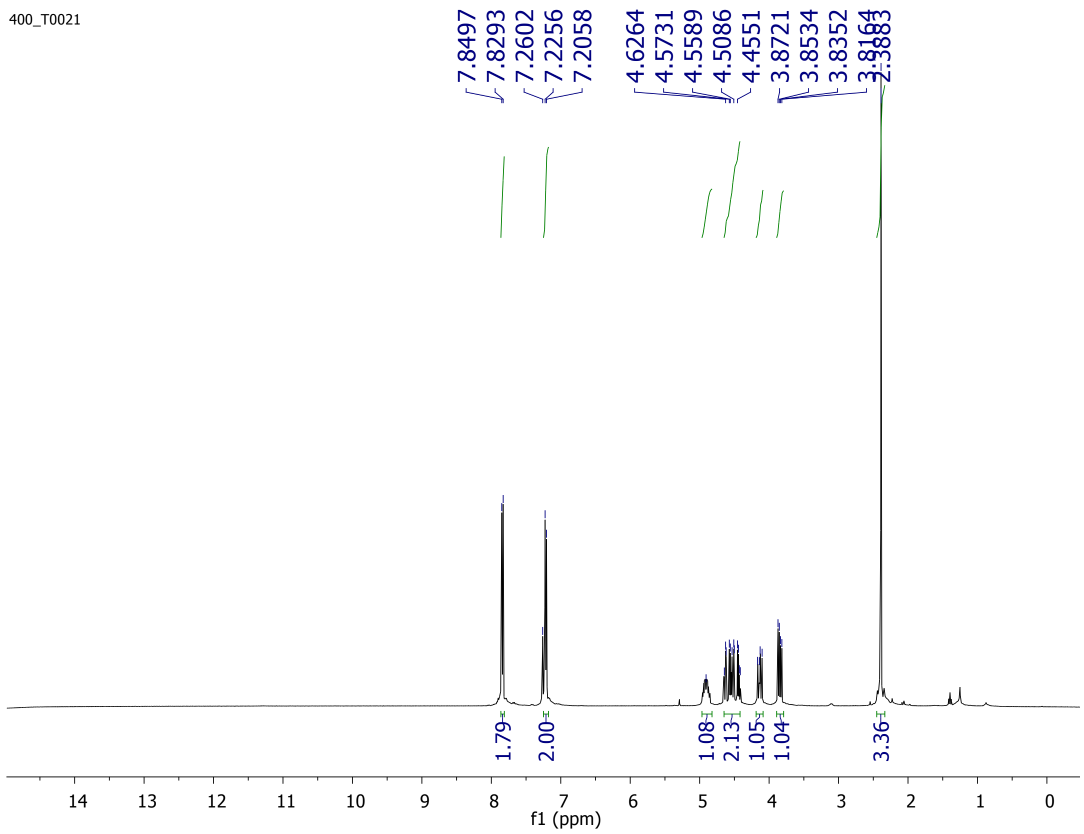

^{19}F NMR of 5-(Fluoromethyl)-2-(4-methylphenyl)-oxazoline (2a)

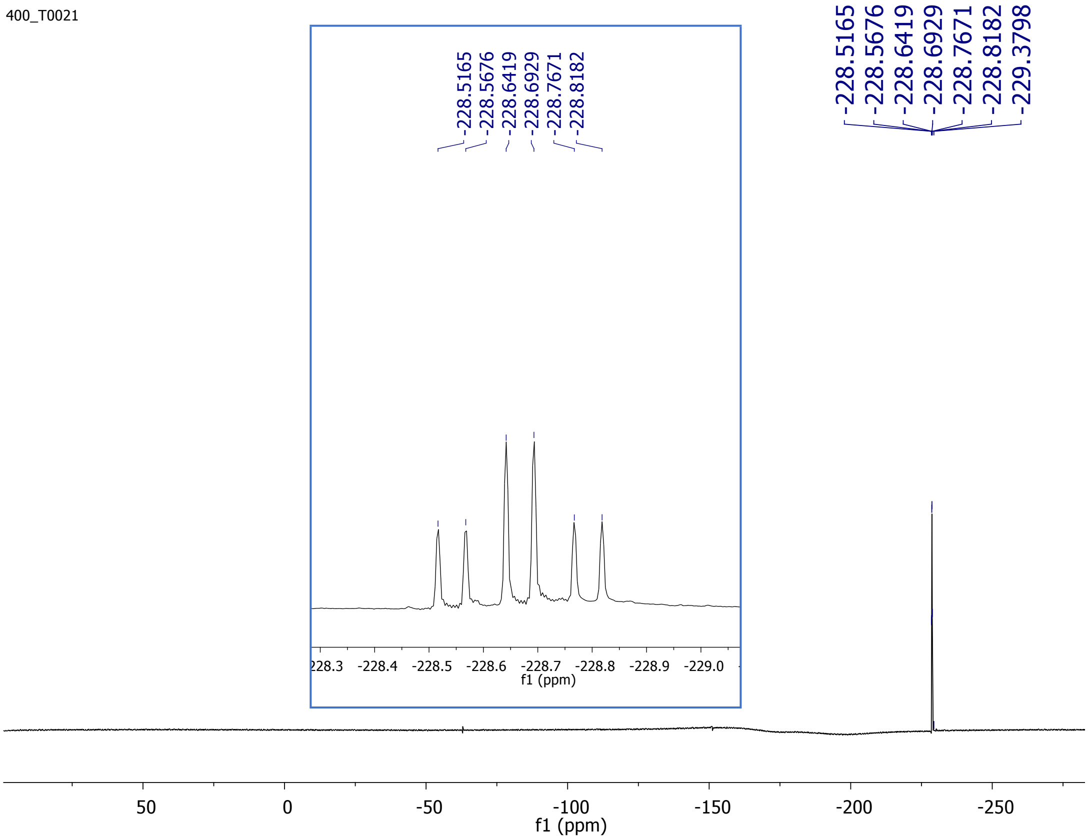

^{13}C NMR of 5-(Fluoromethyl)-2-(4-methylphenyl)-oxazoline (2a)

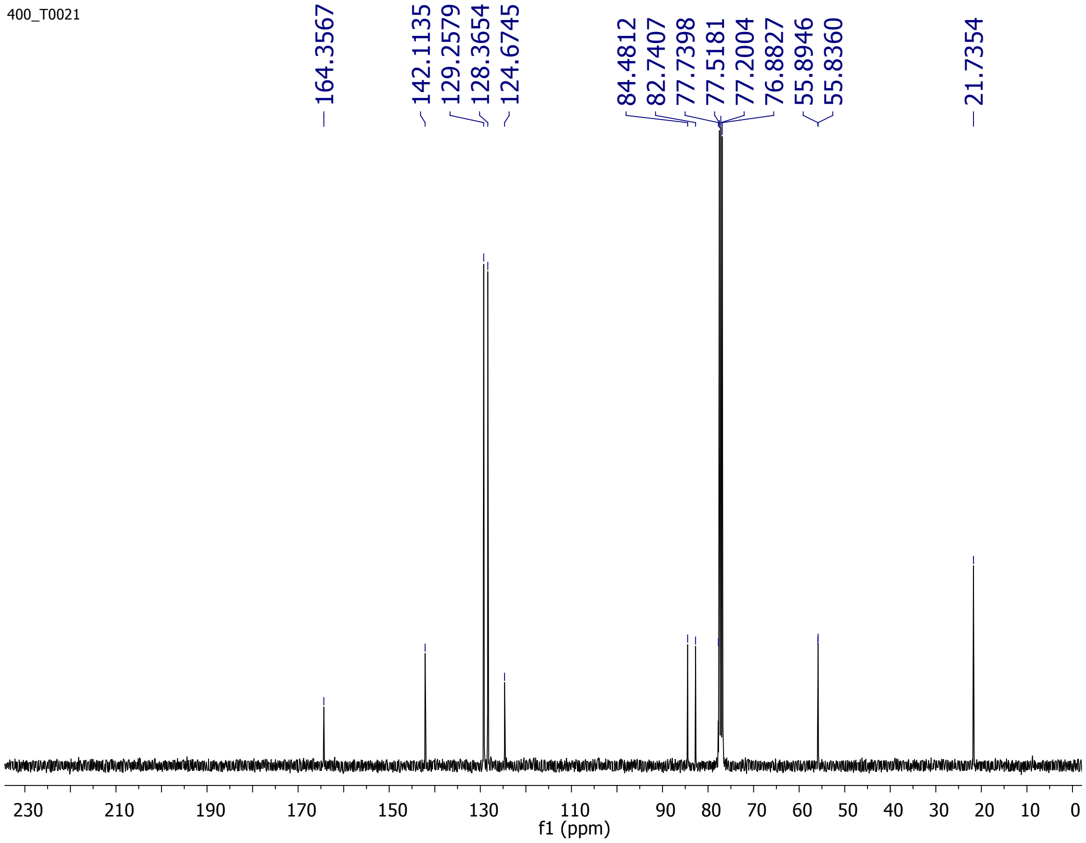

^{1}H NMR of 5-(Fluoromethyl)-2-(4-nitrophenyl)-oxazoline (2c)

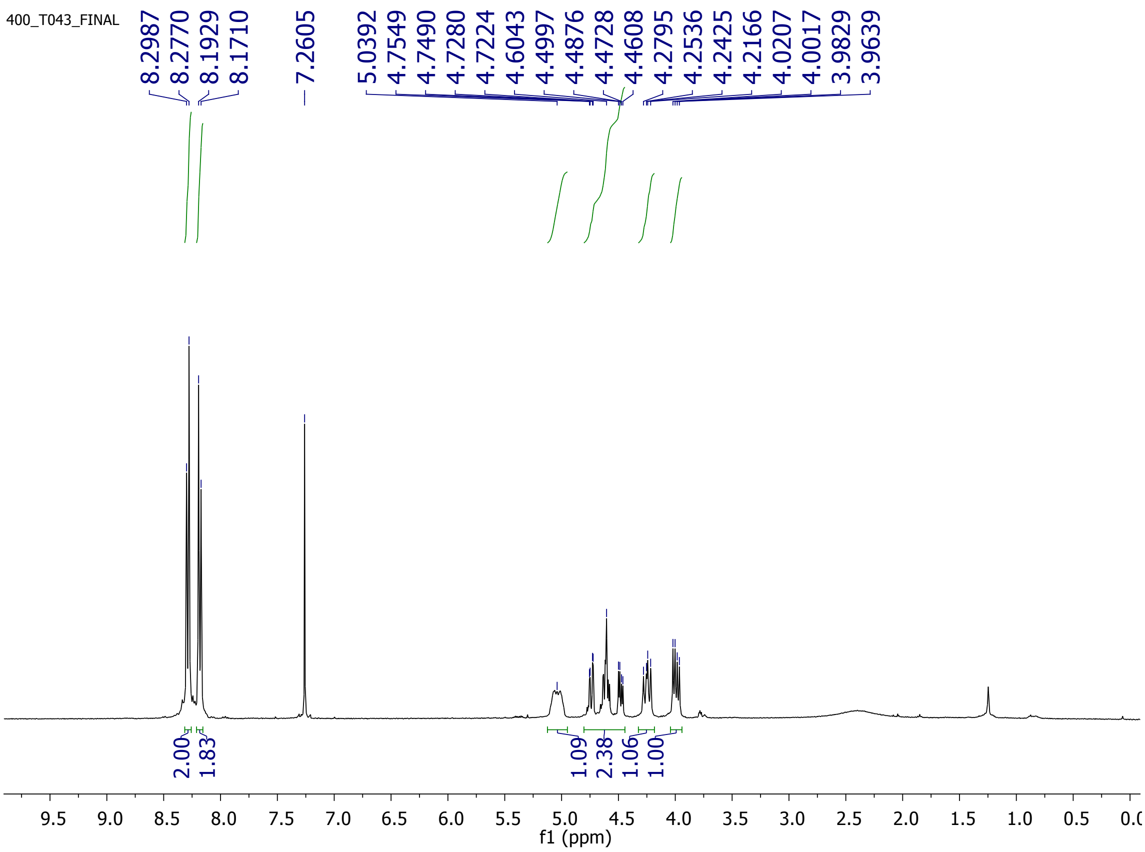

^{19}F NMR of 5-(Fluoromethyl)-2-(4-nitrophenyl)-oxazoline (2c)

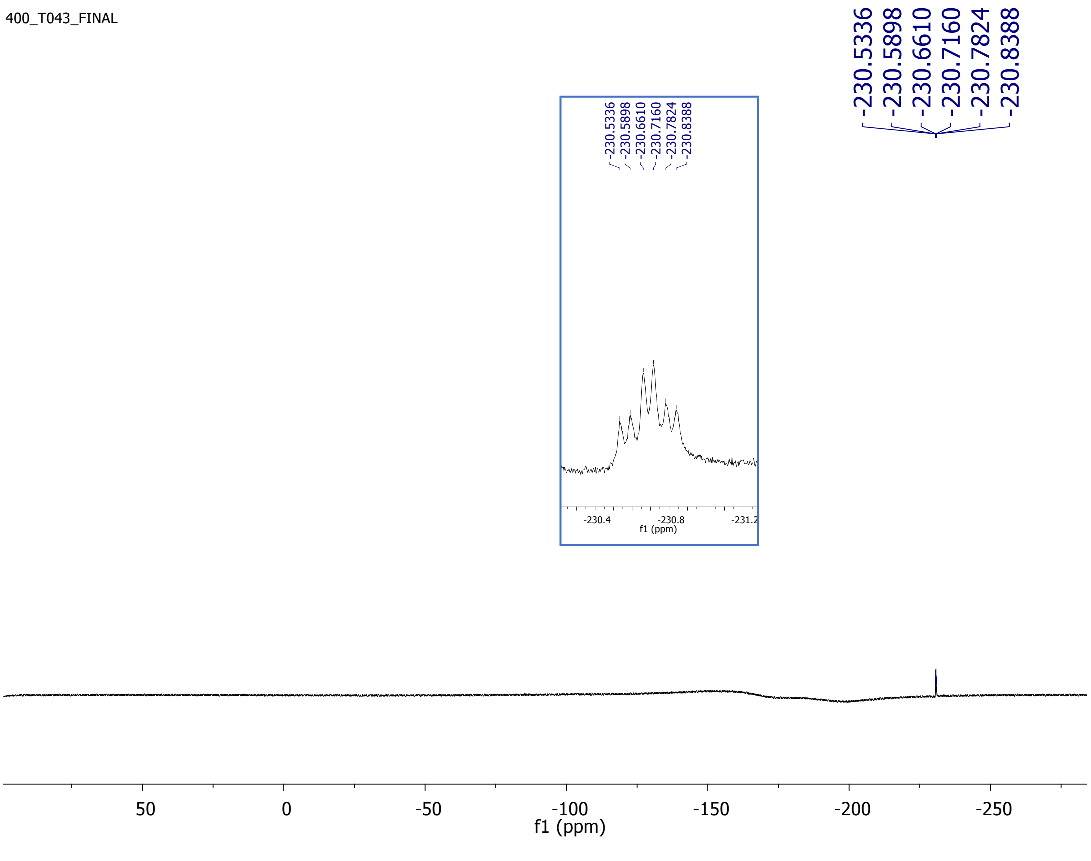

^{19}C NMR of 5-(Fluoromethyl)-2-(4-nitrophenyl)-oxazoline (2c)

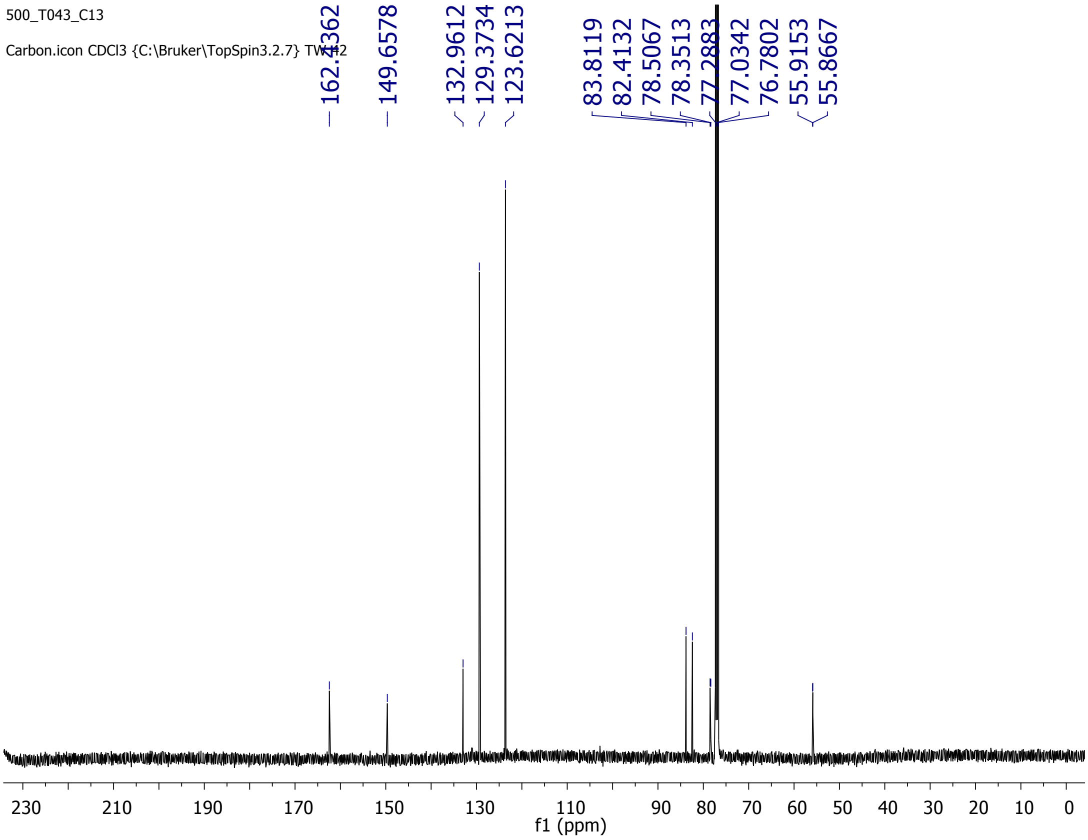

^{1}H NMR of 5-(Fluoromethyl)-2-(4-(trifluoromethyl)phenyl)-oxazoline (2d)

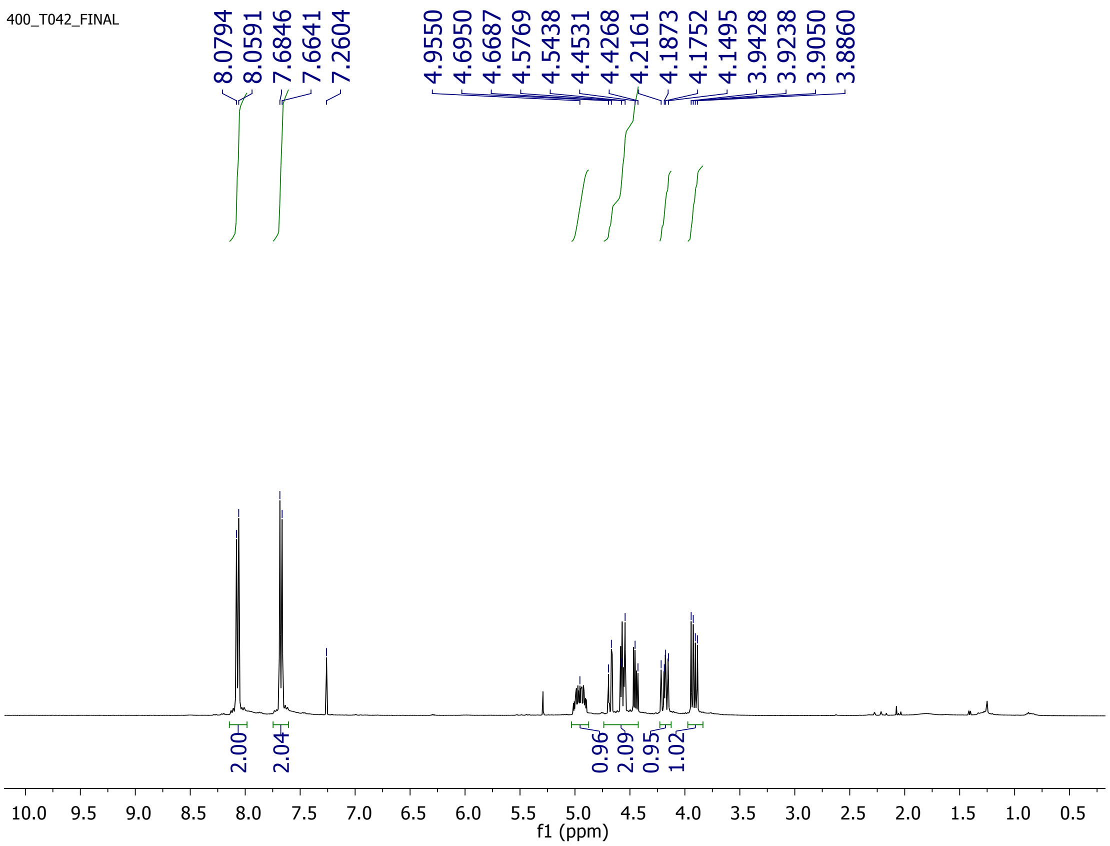

^{19}F NMR of 5-(Fluoromethyl)-2-(4-(trifluoromethyl)phenyl)-oxazoline (2d)

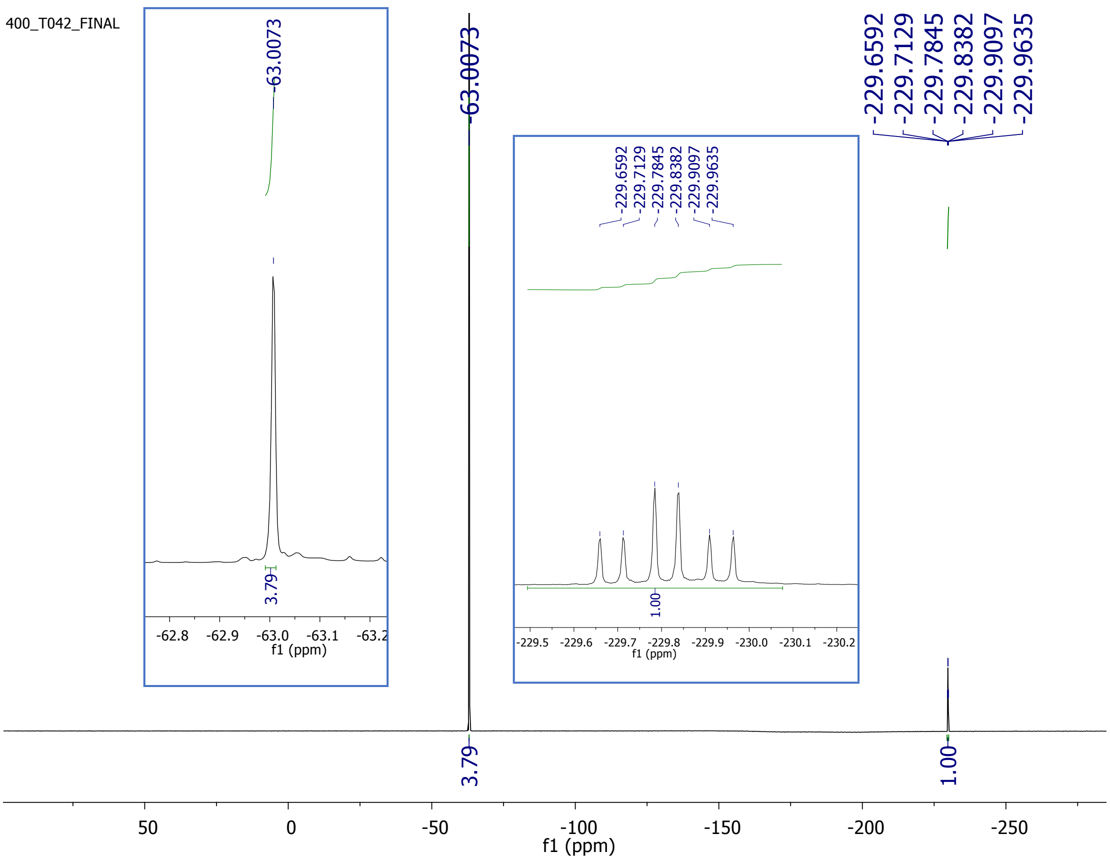

^{13}C NMR of 5-(Fluoromethyl)-2-(4-(trifluoromethyl)phenyl)-oxazoline (2d)

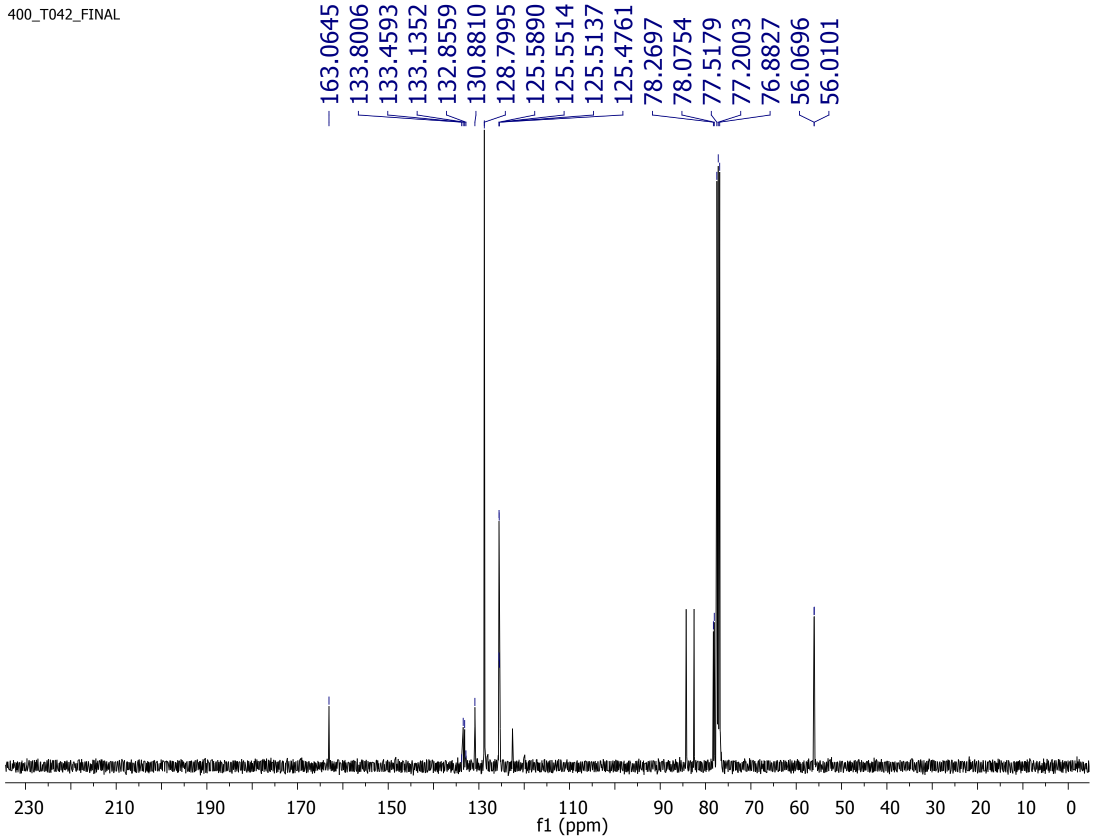

^{1}H NMR of 5-(Fluoromethyl)-2-(4-methoxyphenyl)-oxazoline (2e)

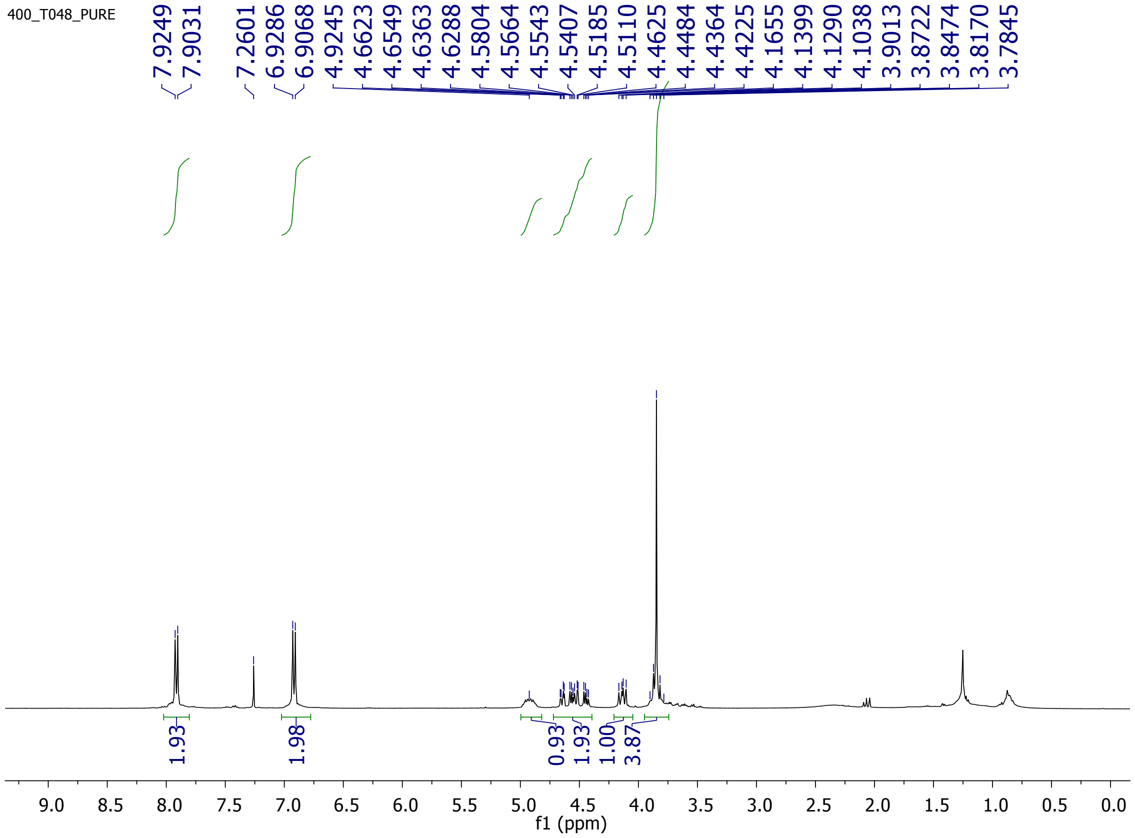

^{19}F NMR of 5-(Fluoromethyl)-2-(4-methoxyphenyl)-oxazoline (2e)

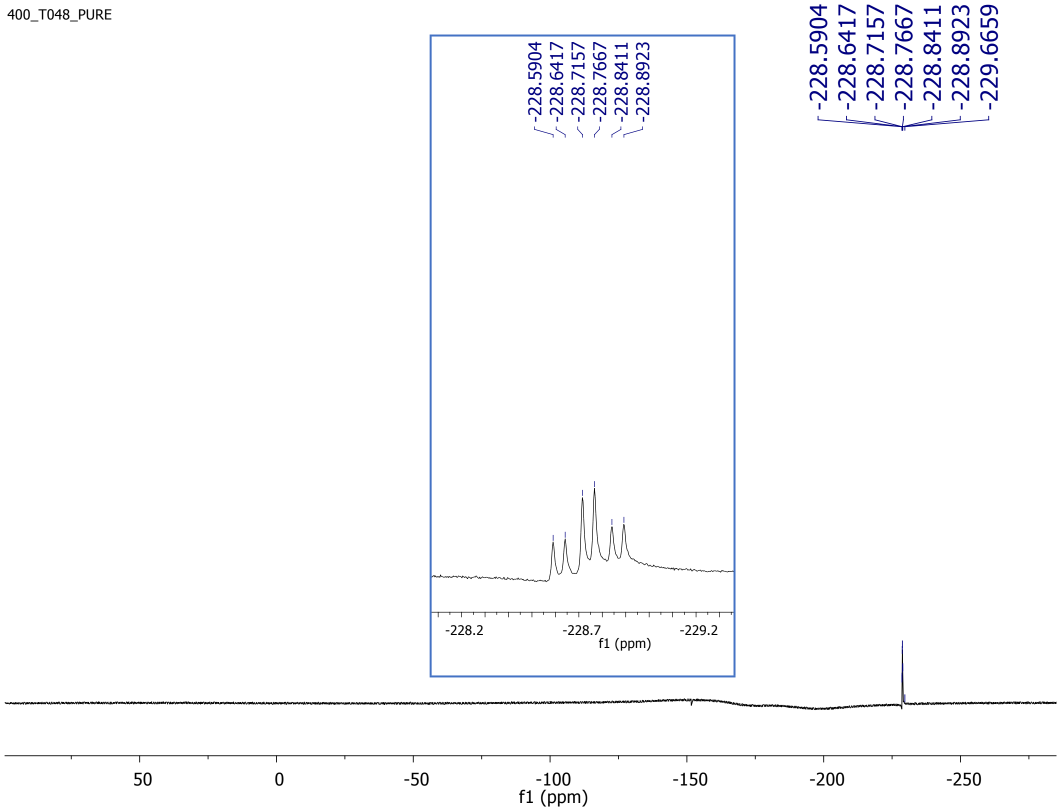

^{13}C NMR of 5-(Fluoromethyl)-2-(4-methoxyphenyl)-oxazoline (2e)

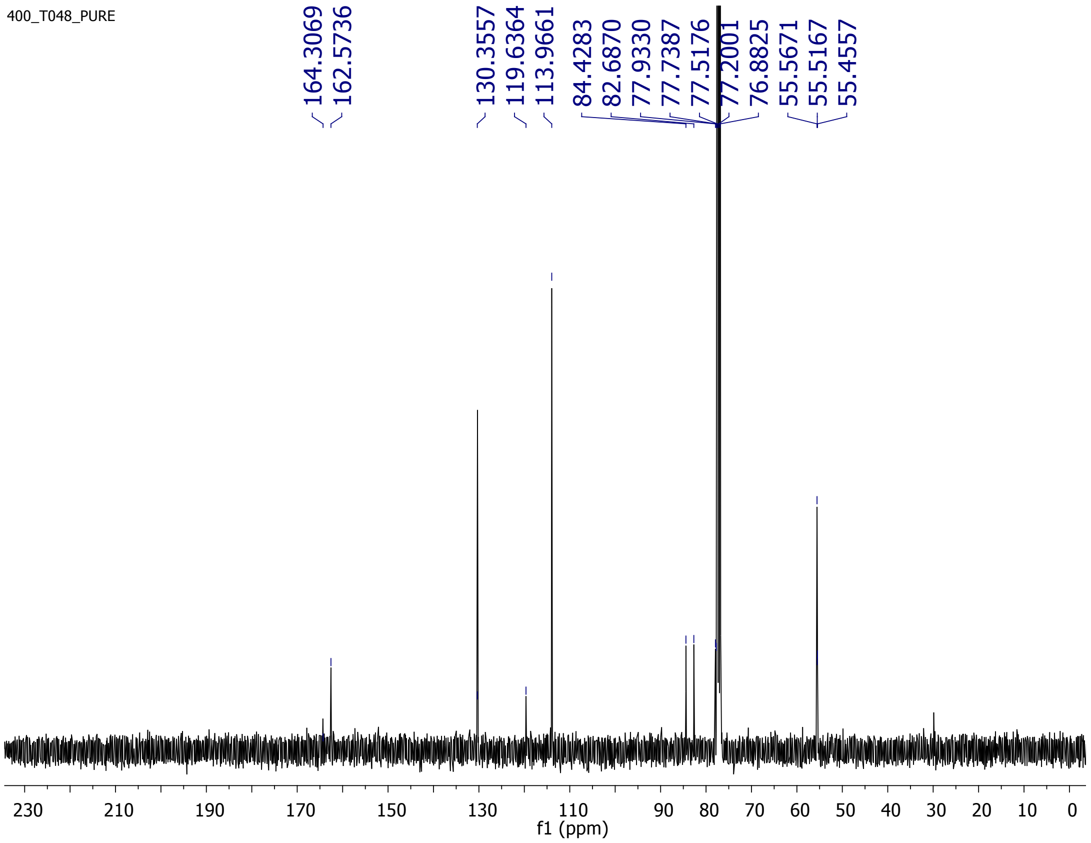

^{1}H NMR of 5-(Fluoromethyl)-2-(thiophen-2-yl)-oxazoline (2f)

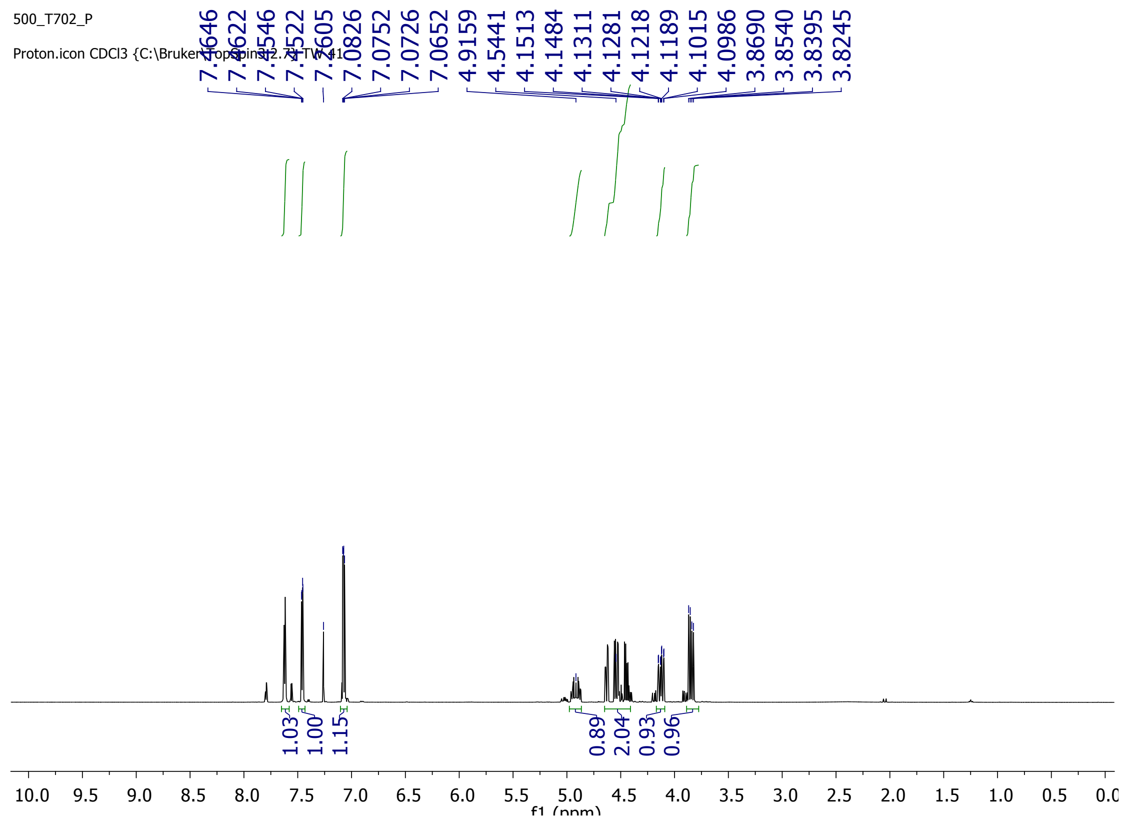

^{13}C NMR of 5-(Fluoromethyl)-2-(thiophen-2-yl)-oxazoline (2f)

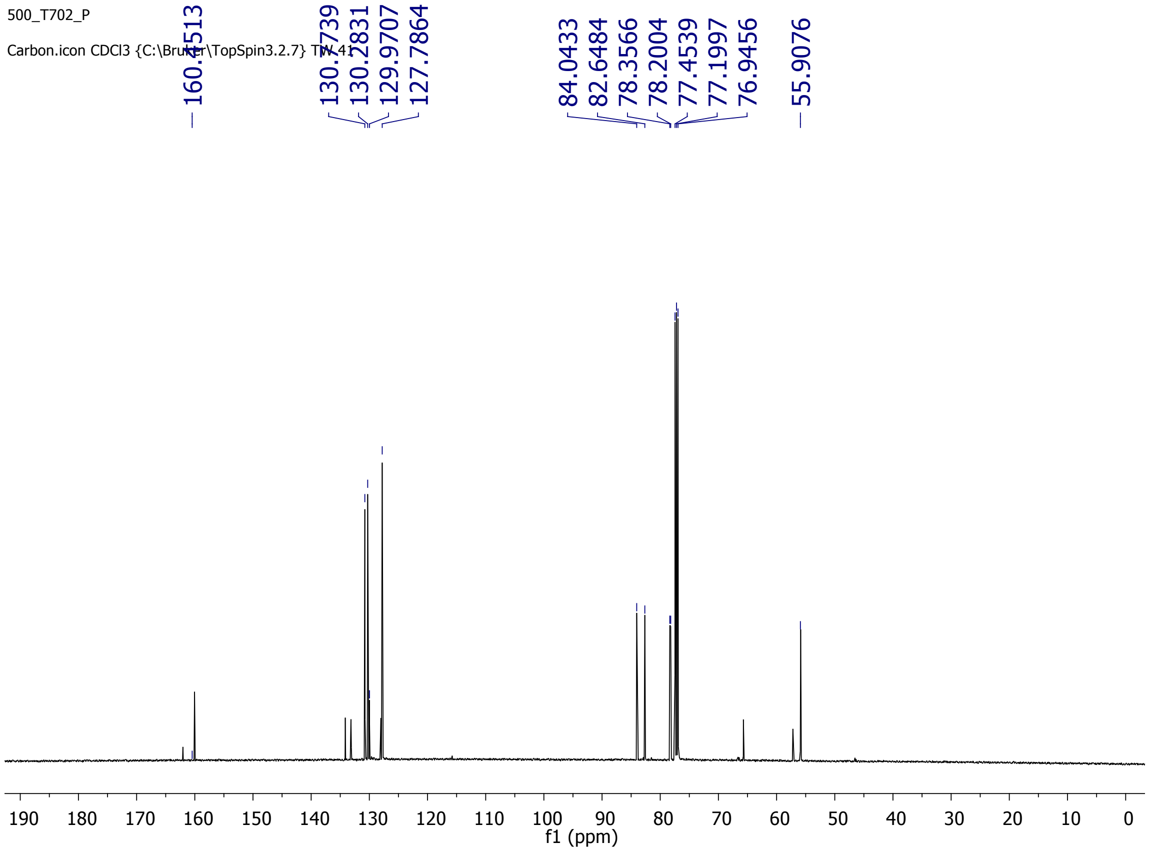

^{1}H NMR of 5-(Fluoromethyl)-2-(furan-2-yl)-4,5-dihydrooxazole (2g)

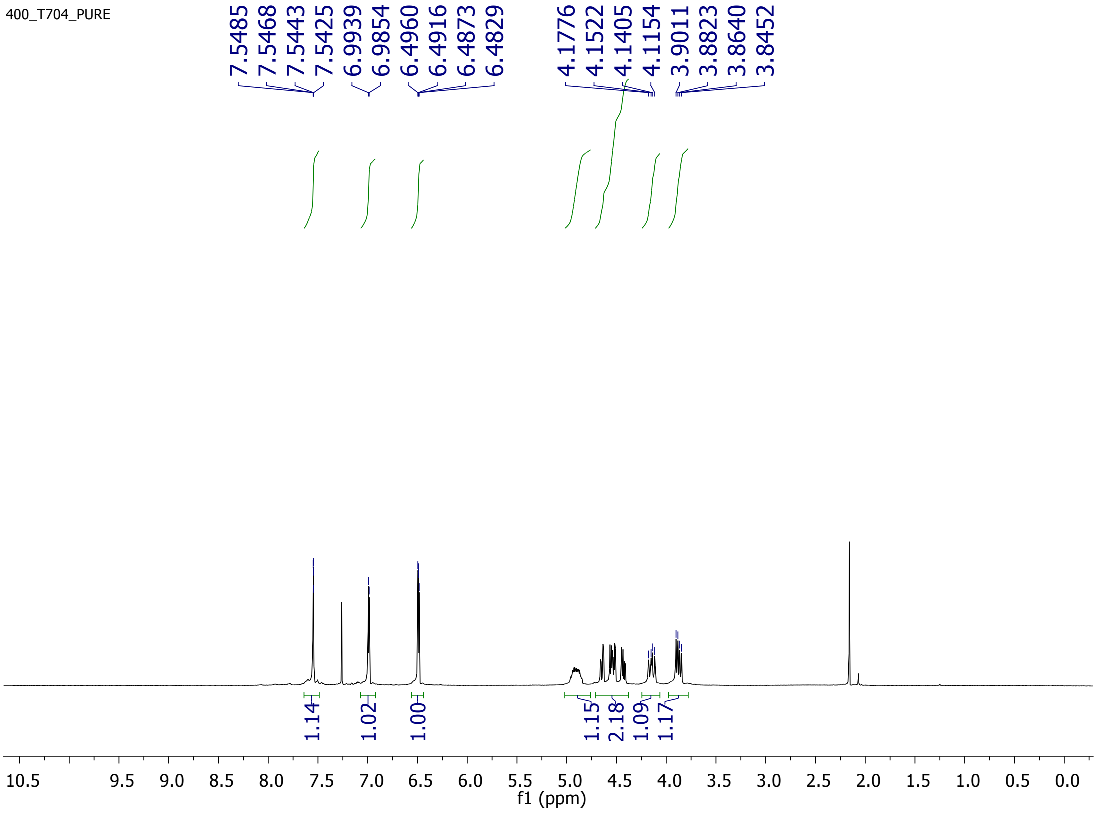

^{19}F NMR of 5-(Fluoromethyl)-2-(furan-2-yl)-4,5-dihydrooxazole (2g)

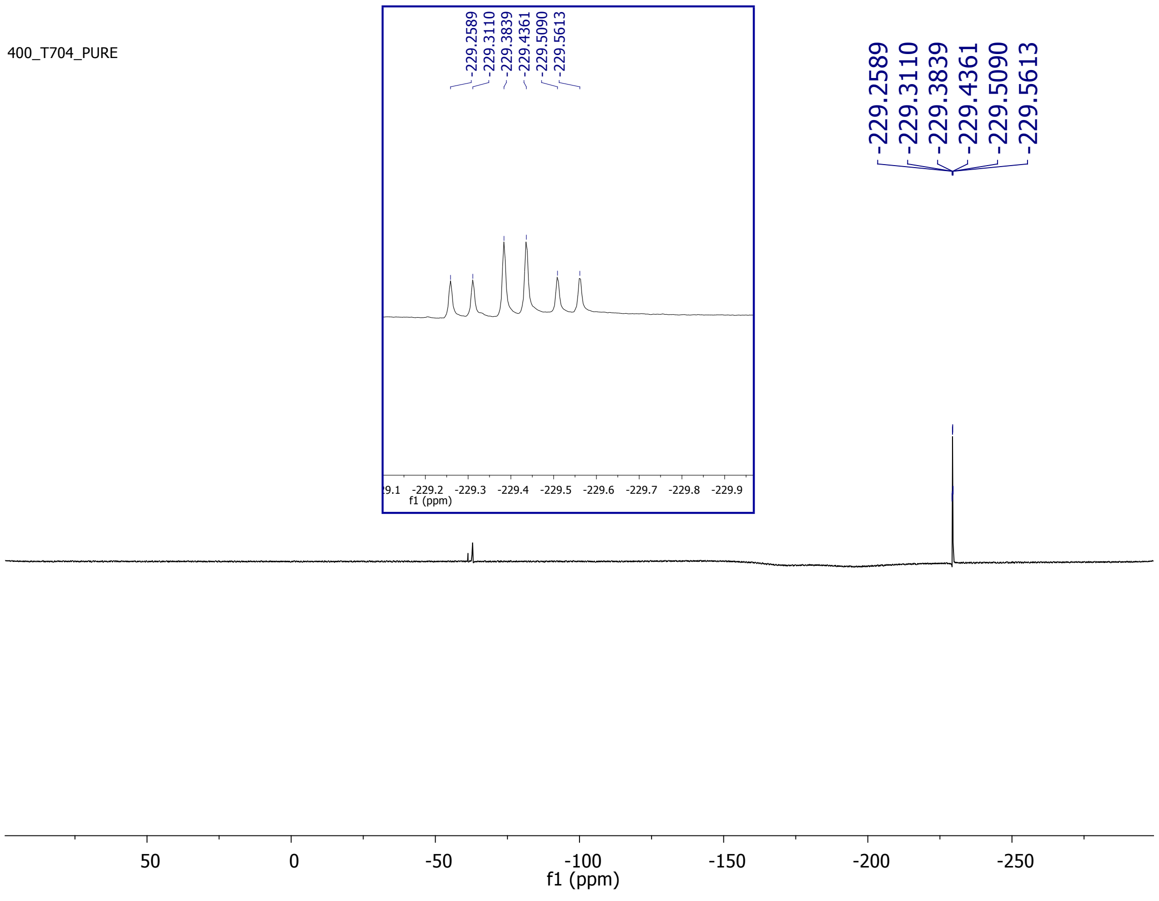

^{13}C NMR of 5-(Fluoromethyl)-2-(furan-2-yl)-4,5-dihydrooxazole (2g)

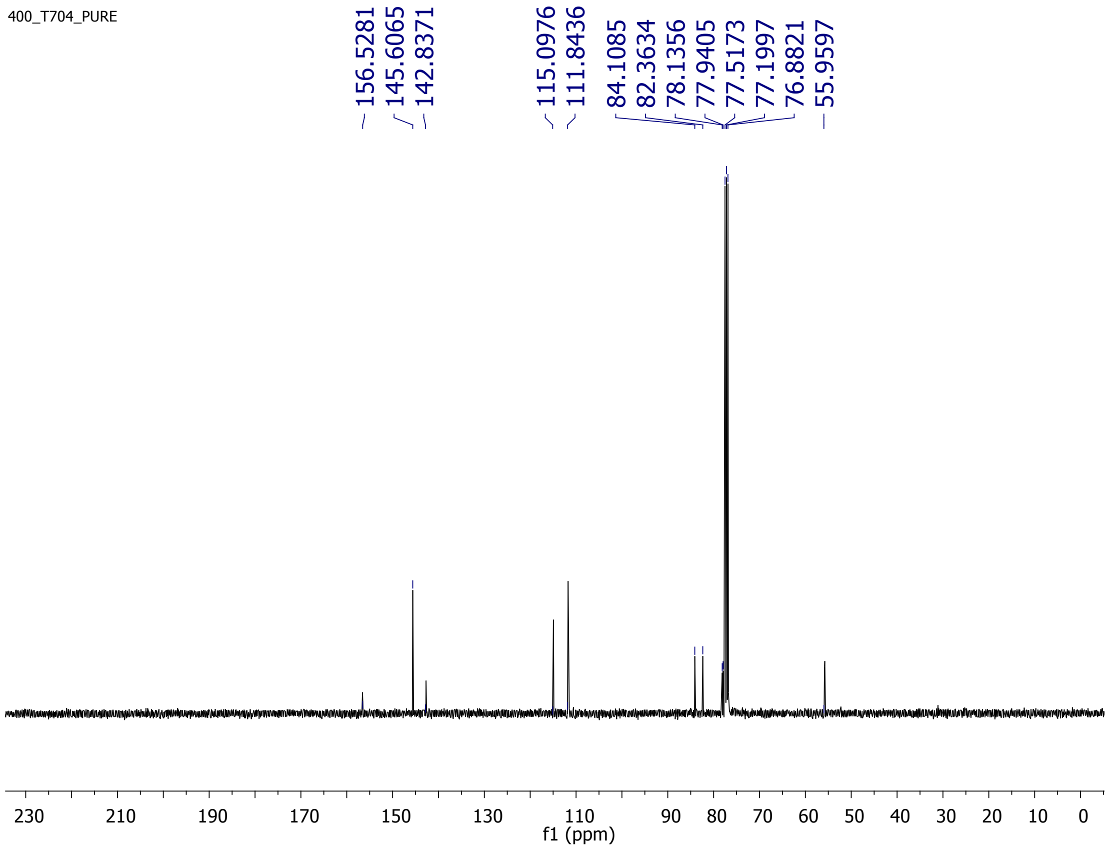

^{1}H NMR of 5-(Fluoromethyl)-2-(tetrahydrofuran-2-yl)-4,5-dihydrooxazole (2i)

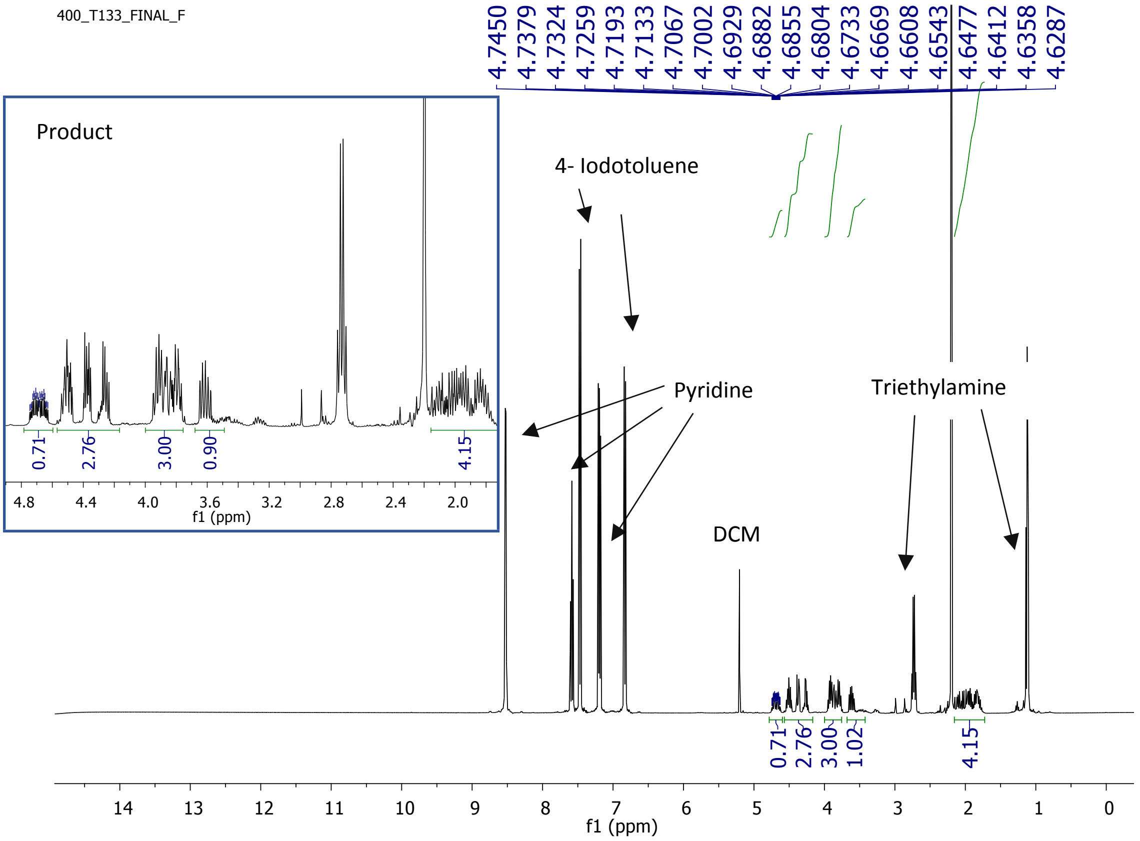

^{19}F NMR of 5-(Fluoromethyl)-2-(tetrahydrofuran-2-yl)-4,5-dihydrooxazole (2i)

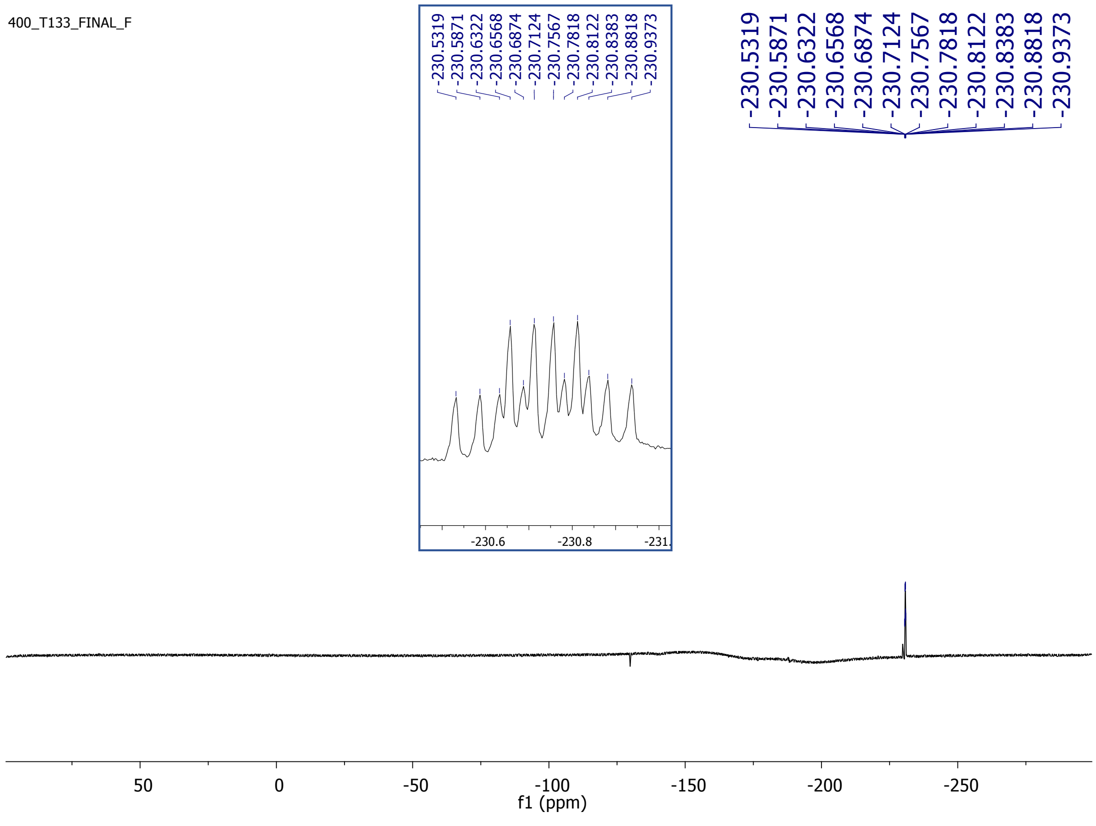

^{13}C NMR of 5-(Fluoromethyl)-2-(tetrahydrofuran-2-yl)-4,5-dihydrooxazole (2i)

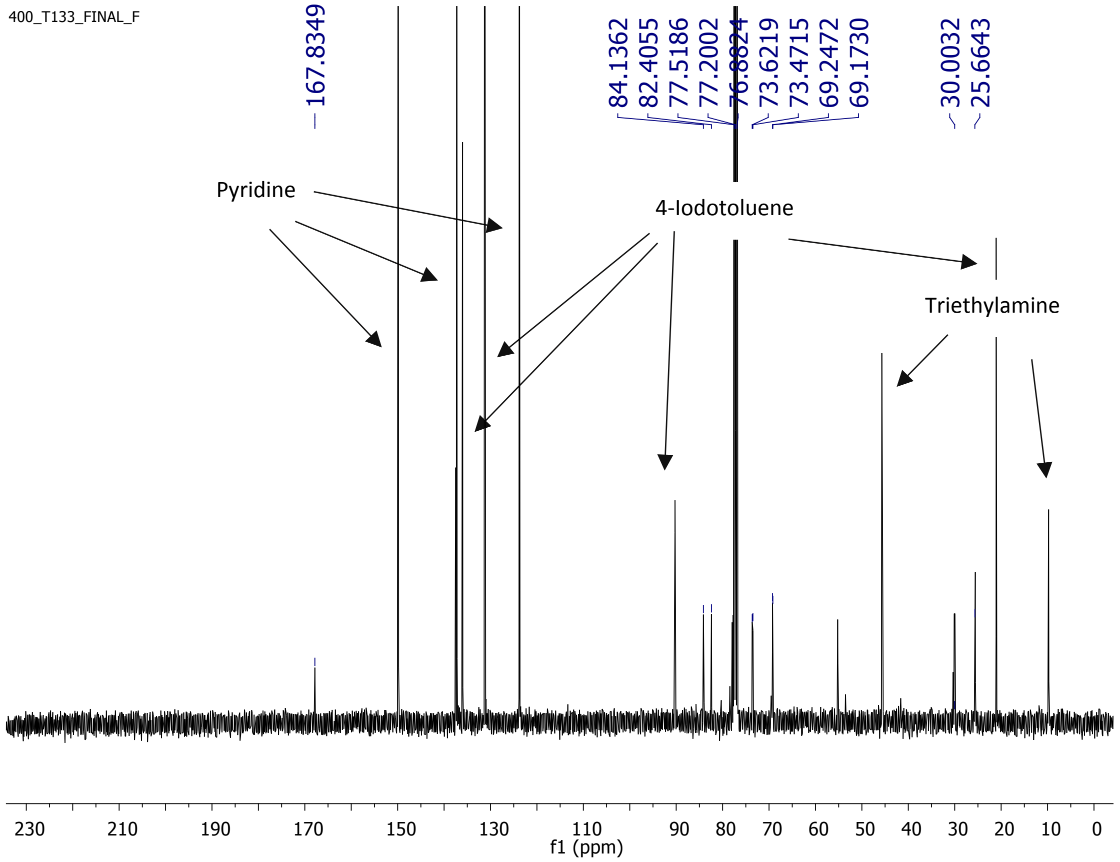

HRMS of 5-(Fluoromethyl)-2-(tetrahydrofuran-2-yl)-4,5-dihydrooxazole (2i)

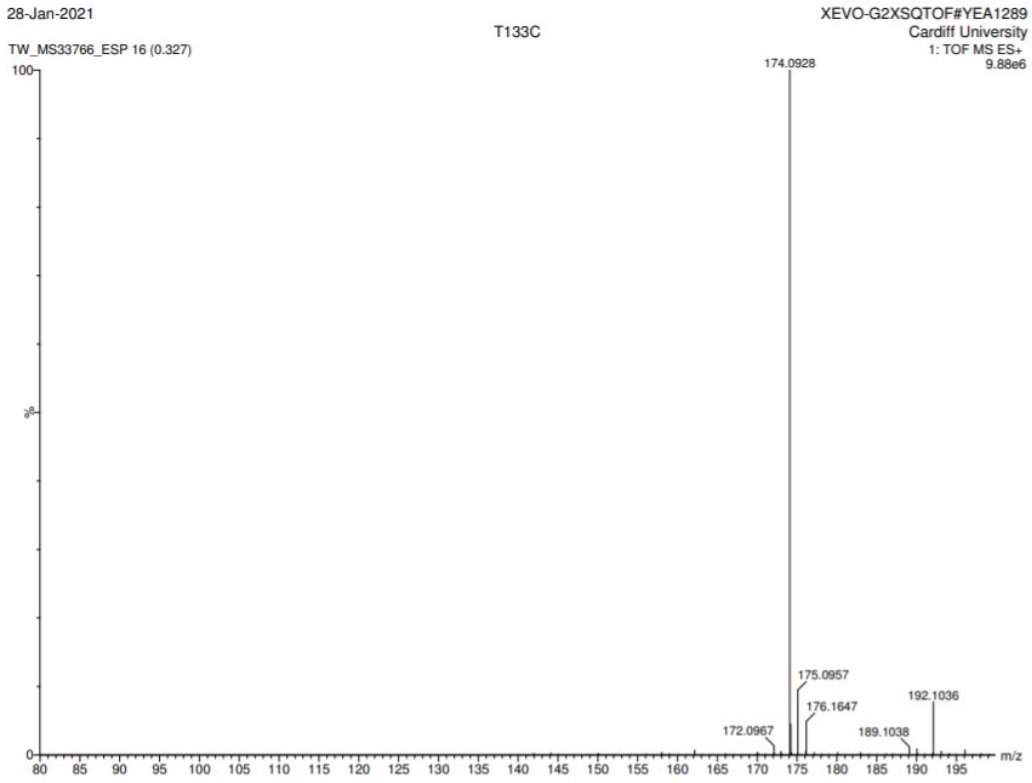

^{1}H NMR of 4-(5-(Fluoromethyl)-4,5-dihydrooxazol-2-yl)morpholine (2j)

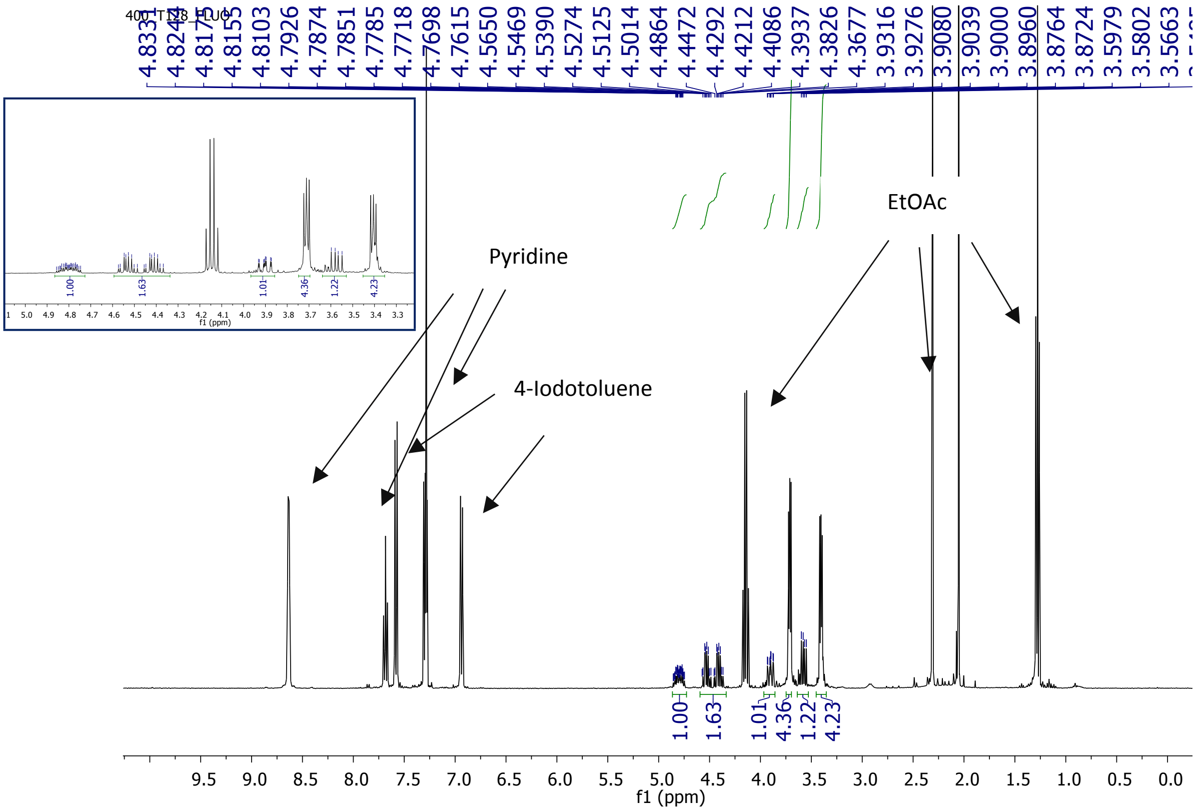

^{19}F NMR of 4-(5-(Fluoromethyl)-4,5-dihydrooxazol-2-yl)morpholine (2j)

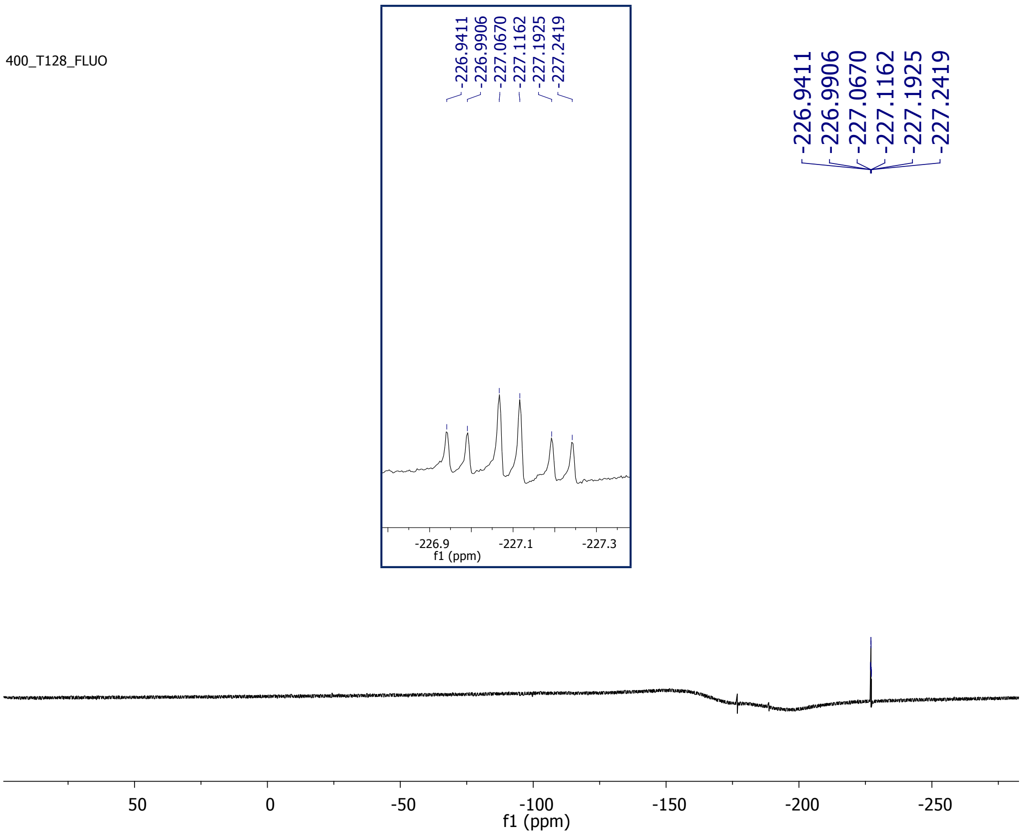

^{13}C NMR of 4-(5-(Fluoromethyl)-4,5-dihydrooxazol-2-yl)morpholine (2j)

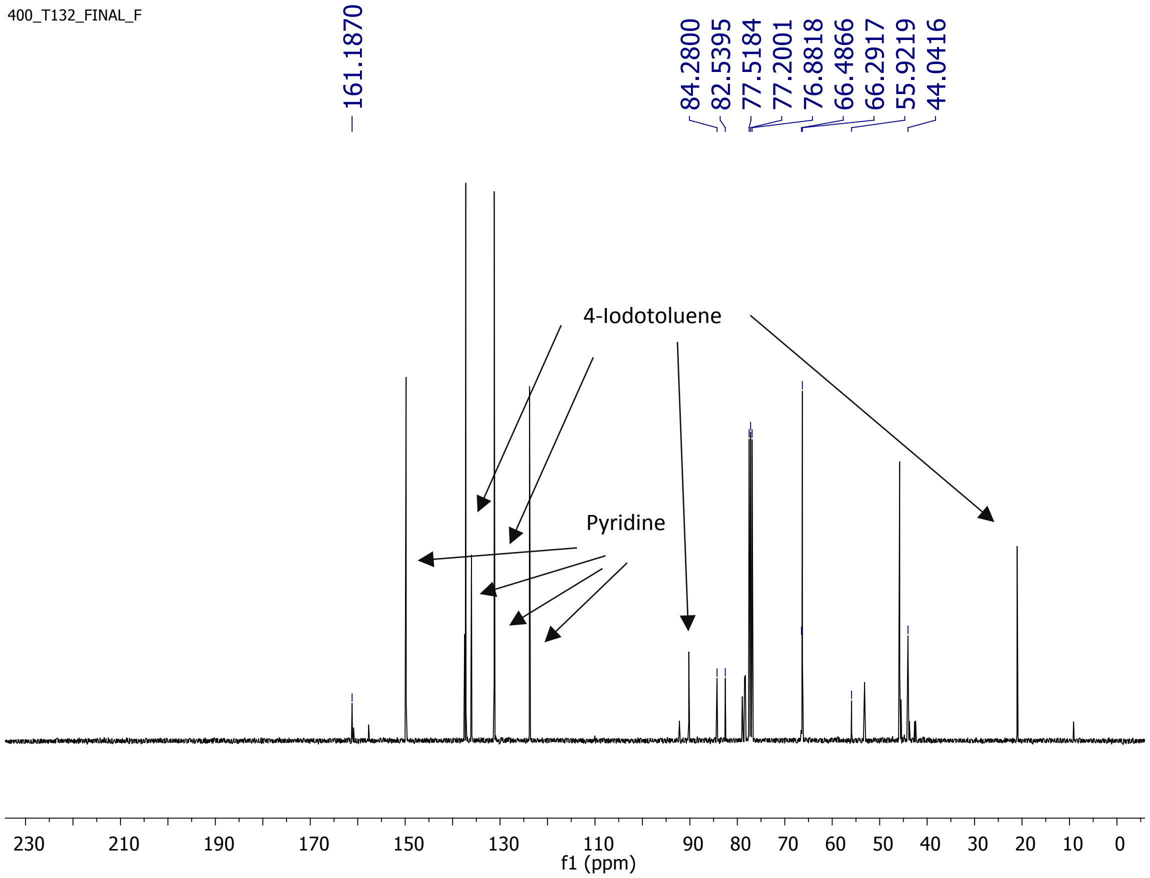

HRMS of 4-(5-(Fluoromethyl)-4,5-dihydrooxazol-2-yl)morpholine (2j)

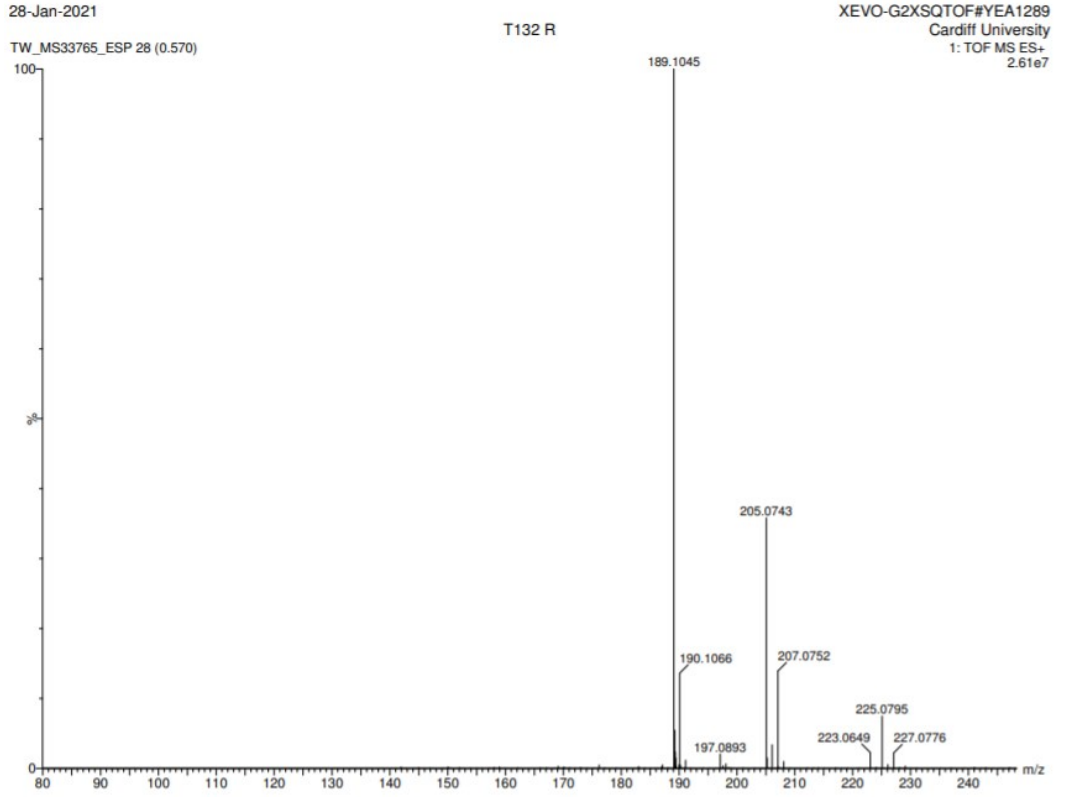

^{1}H NMR of 5-(Fluoromethyl)-2-(pyrrolidin-1-yl)-4,5-dihydrooxazole (2k)

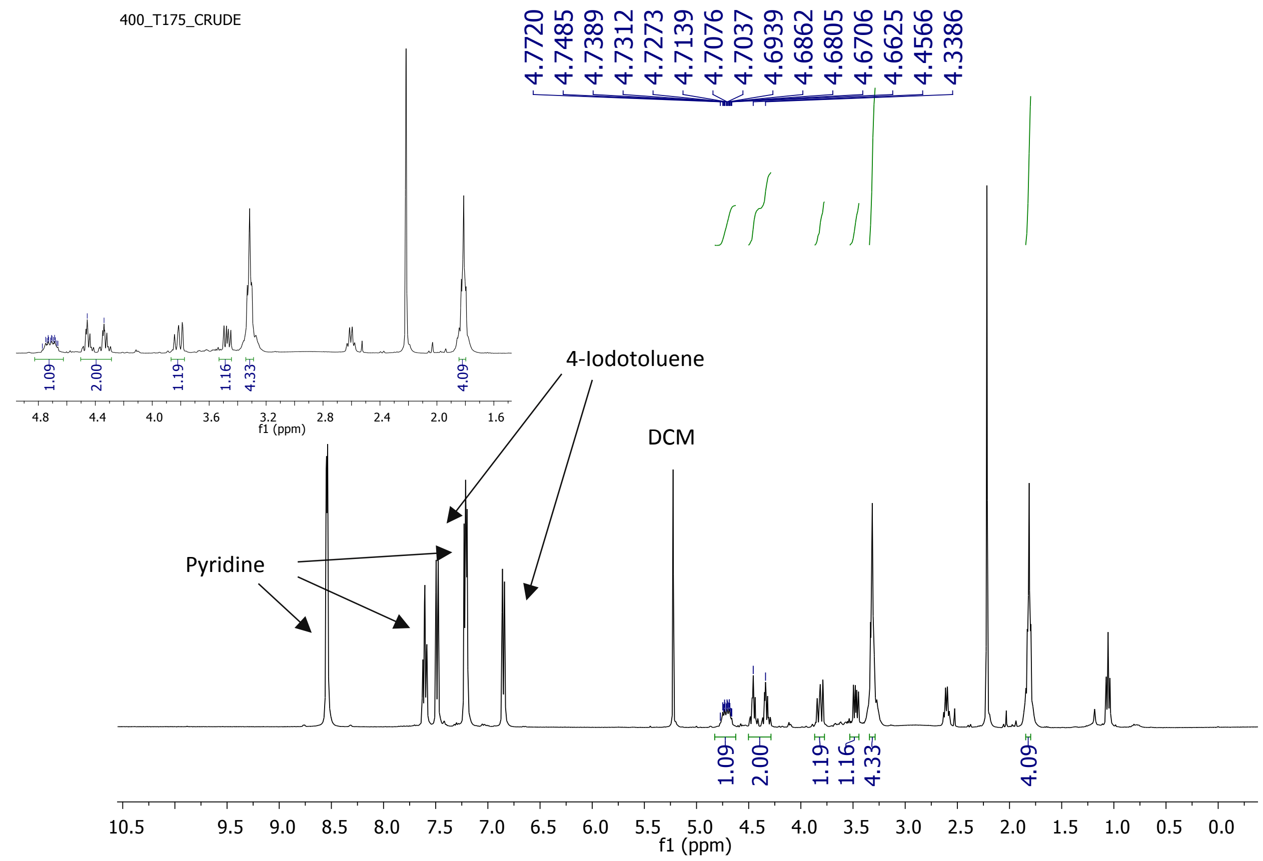

^{19}F NMR of 5-(Fluoromethyl)-2-(pyrrolidin-1-yl)-4,5-dihydrooxazole (2k)

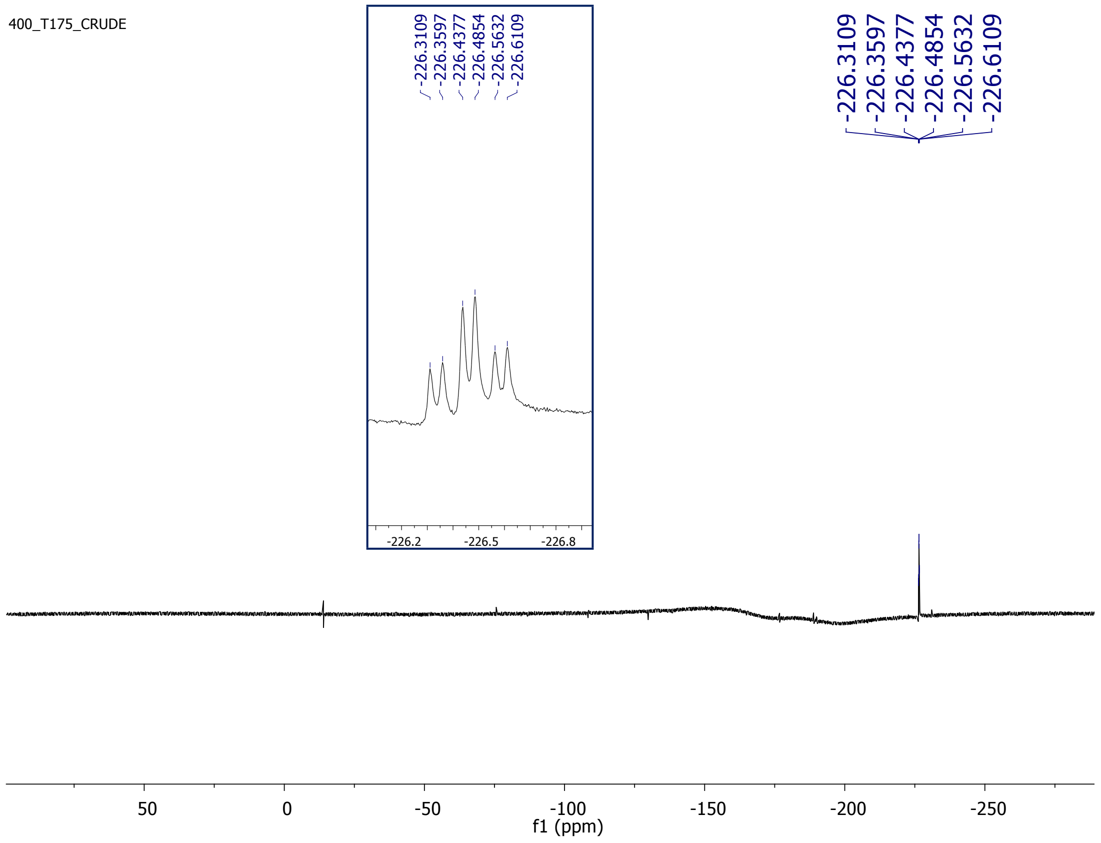

^{13}C NMR of 5-(Fluoromethyl)-2-(pyrrolidin-1-yl)-4,5-dihydrooxazole (2k)

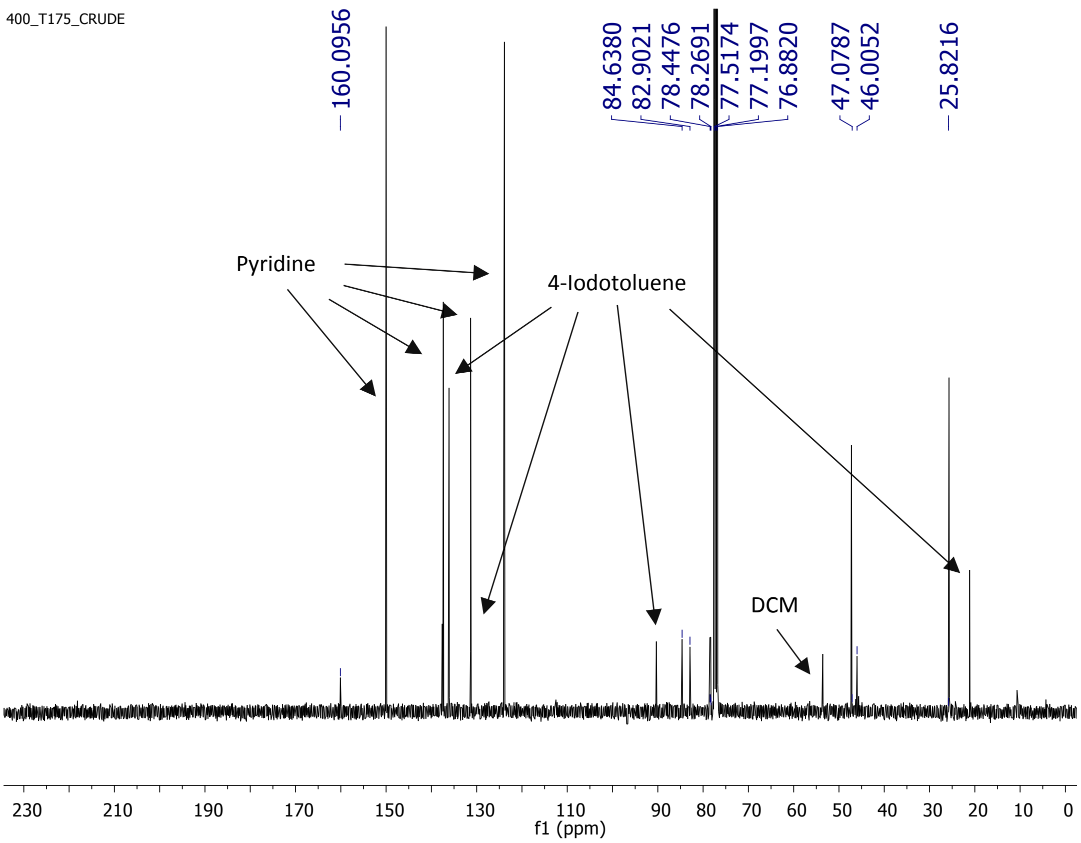

HRMS of 5-(Fluoromethyl)-2-(pyrrolidin-1-yl)-4,5-dihydrooxazole (2k)

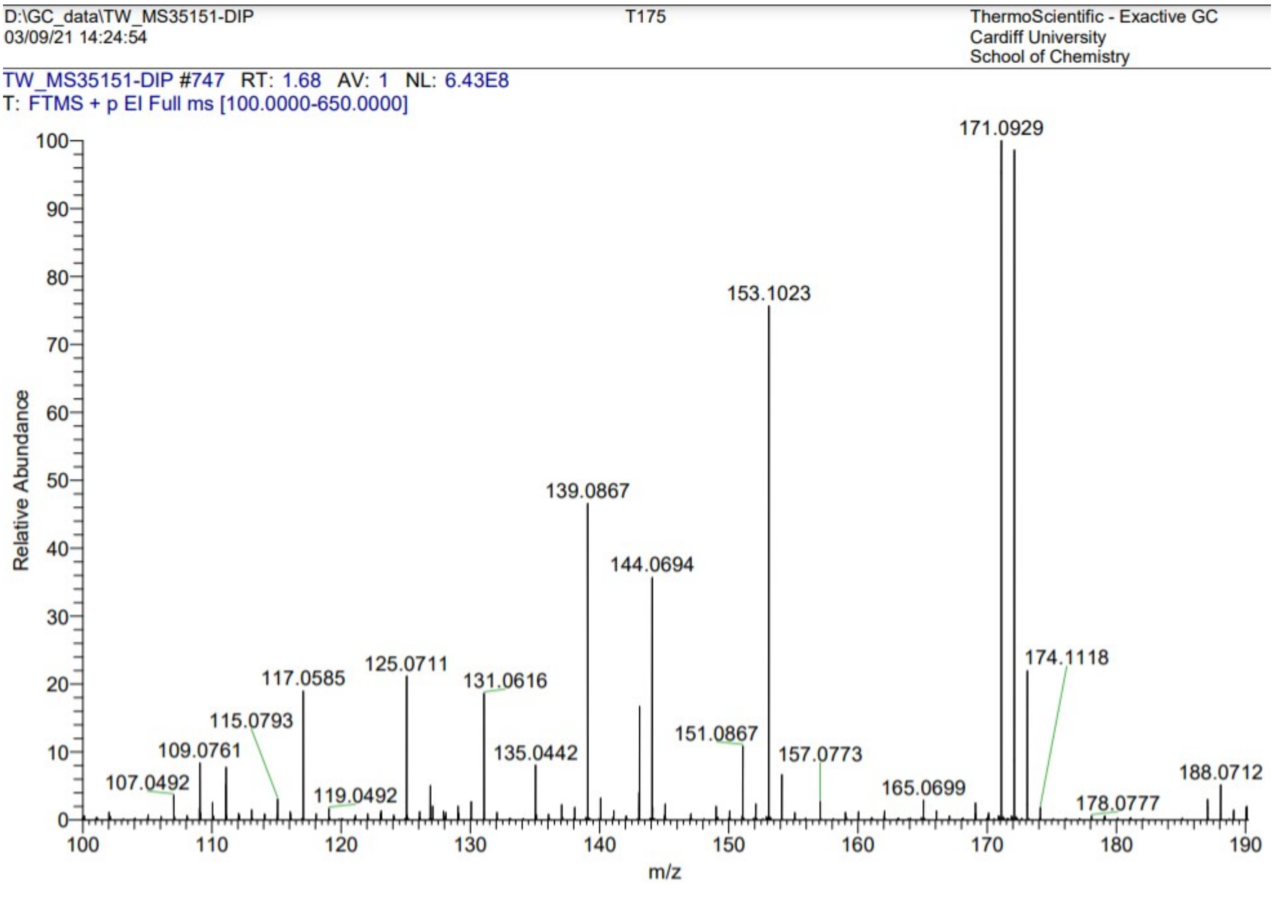

^{1}H NMR of 5-(fluoromethyl)-2-(piperidin-1-yl)-4,5-dihydrooxazole (2ma)

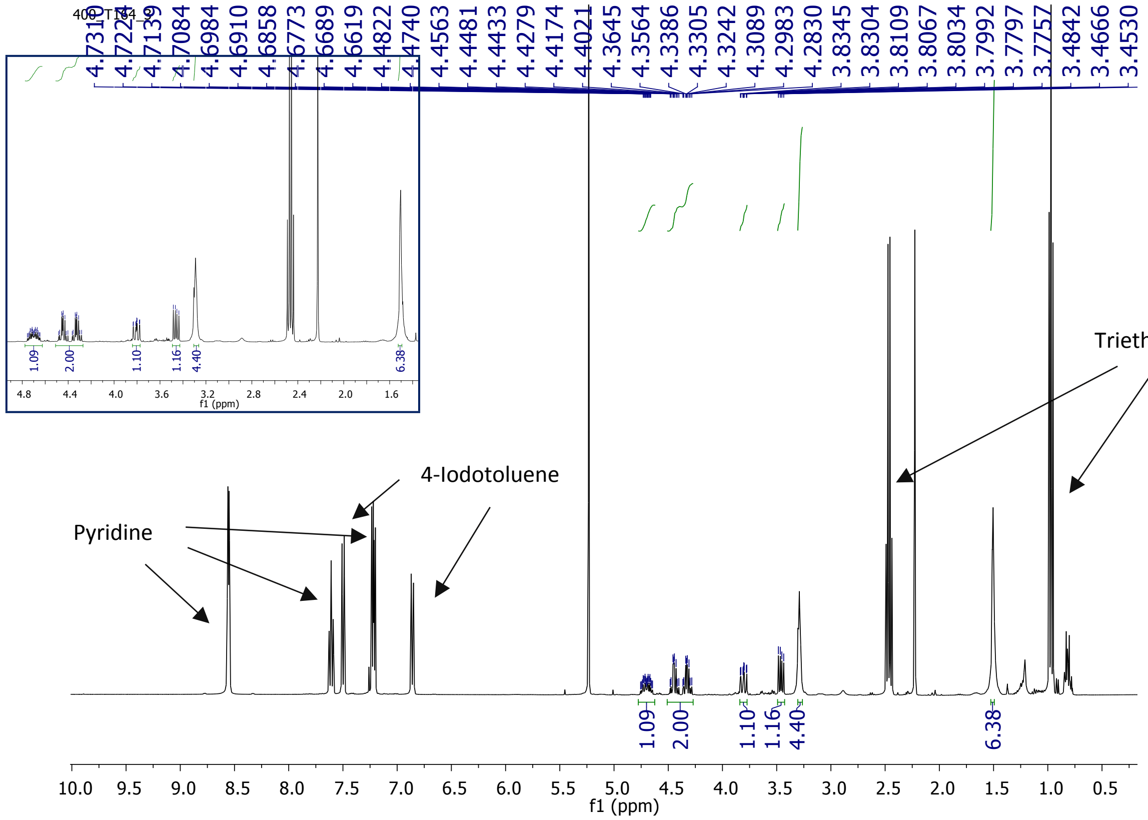

^{19}F NMR of 5-(fluoromethyl)-2-(piperidin-1-yl)-4,5-dihydrooxazole (2ma)

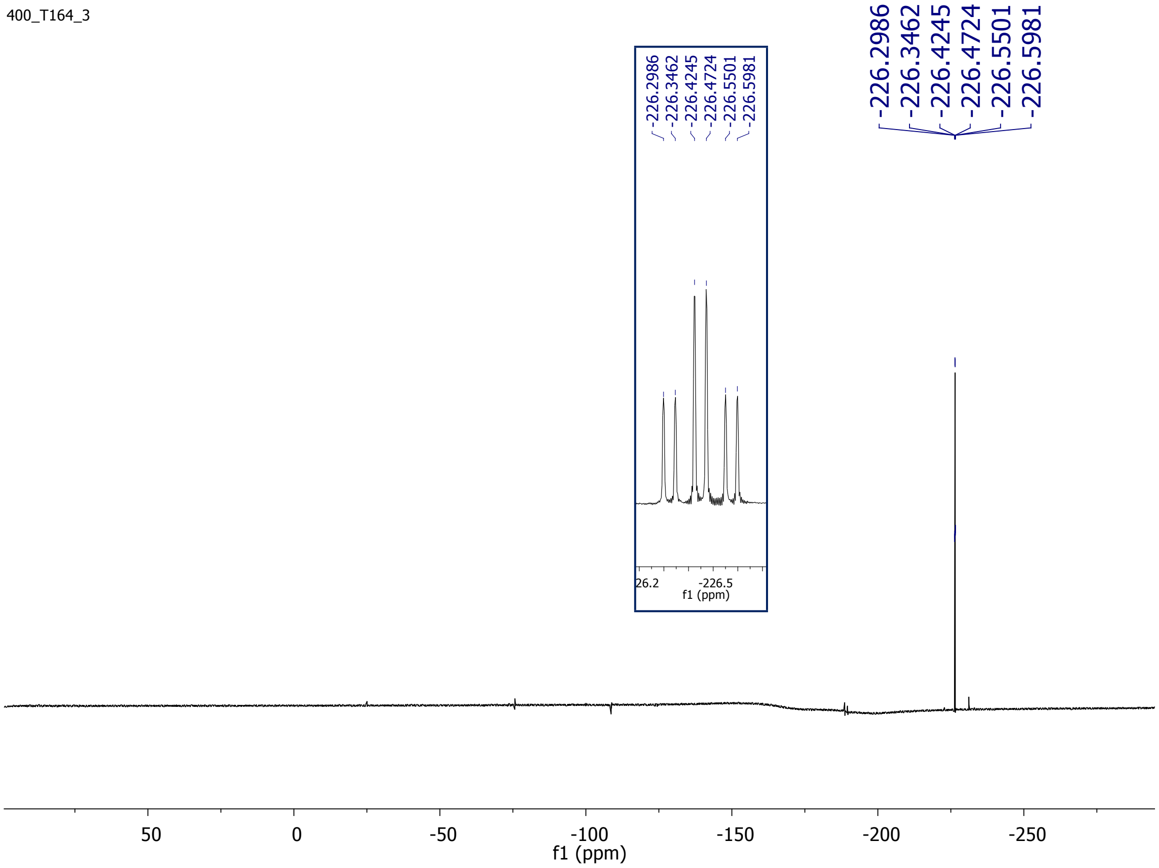

^{13}C NMR of 5-(fluoromethyl)-2-(piperidin-1-yl)-4,5-dihydrooxazole (2ma)

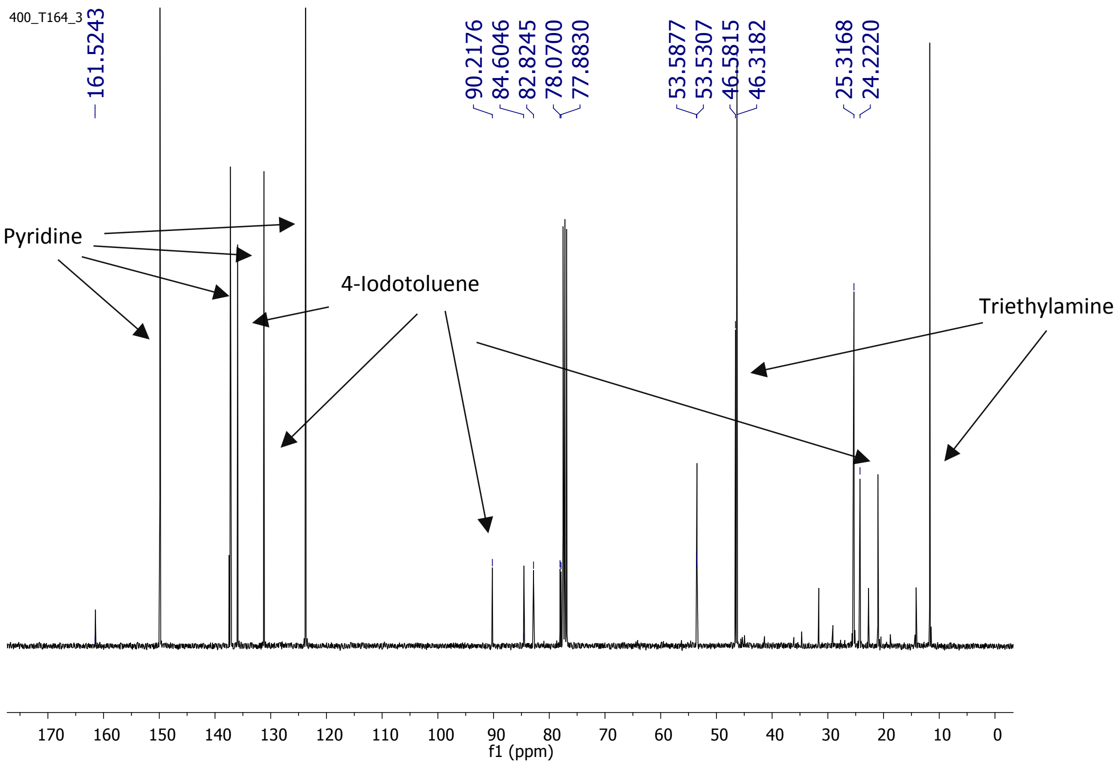

HRMS of 5-(fluoromethyl)-2-(piperidin-1-yl)-4,5-dihydrooxazole (2ma)

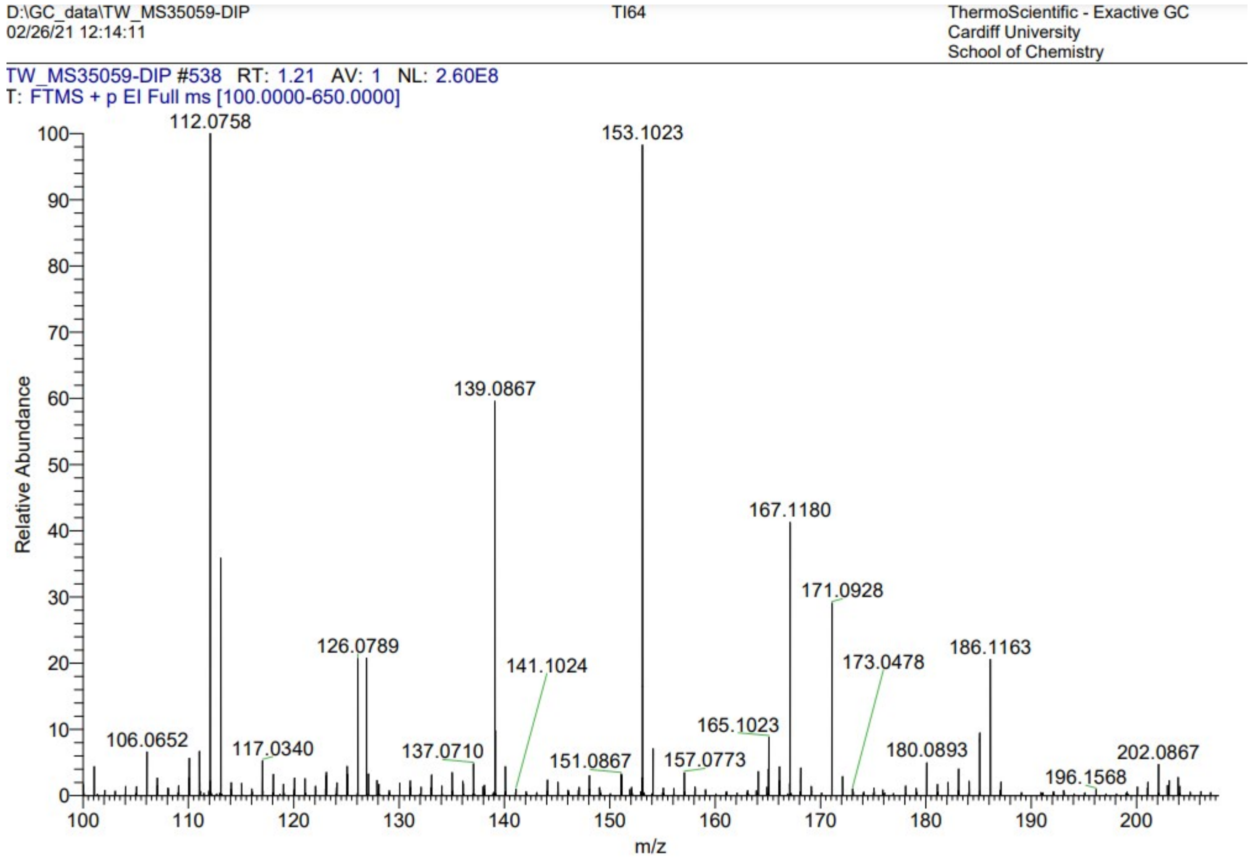

^{1}H NMR of Benzyl 3-(5-(fluoromethyl)-4,5-dihydrooxazol-2-yl)propanoate (2l)

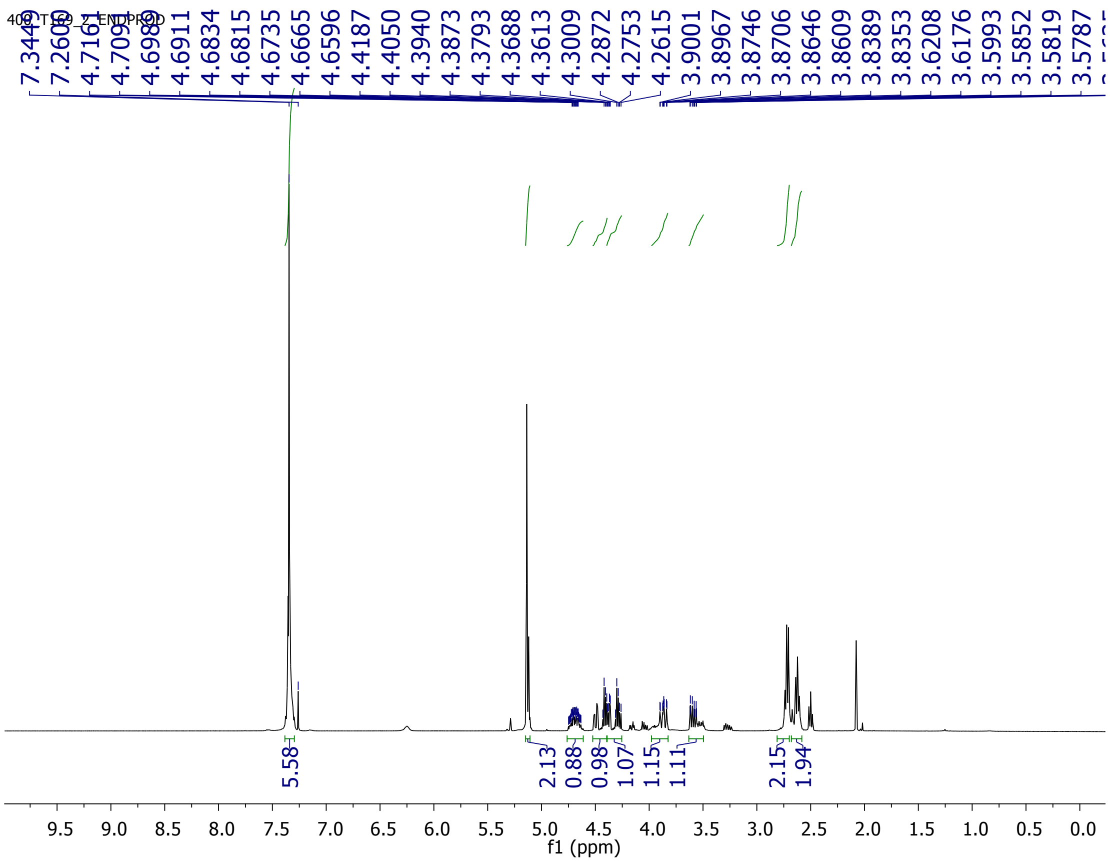

^{19}F NMR of Benzyl 3-(5-(fluoromethyl)-4,5-dihydrooxazol-2-yl)propanoate (2l)

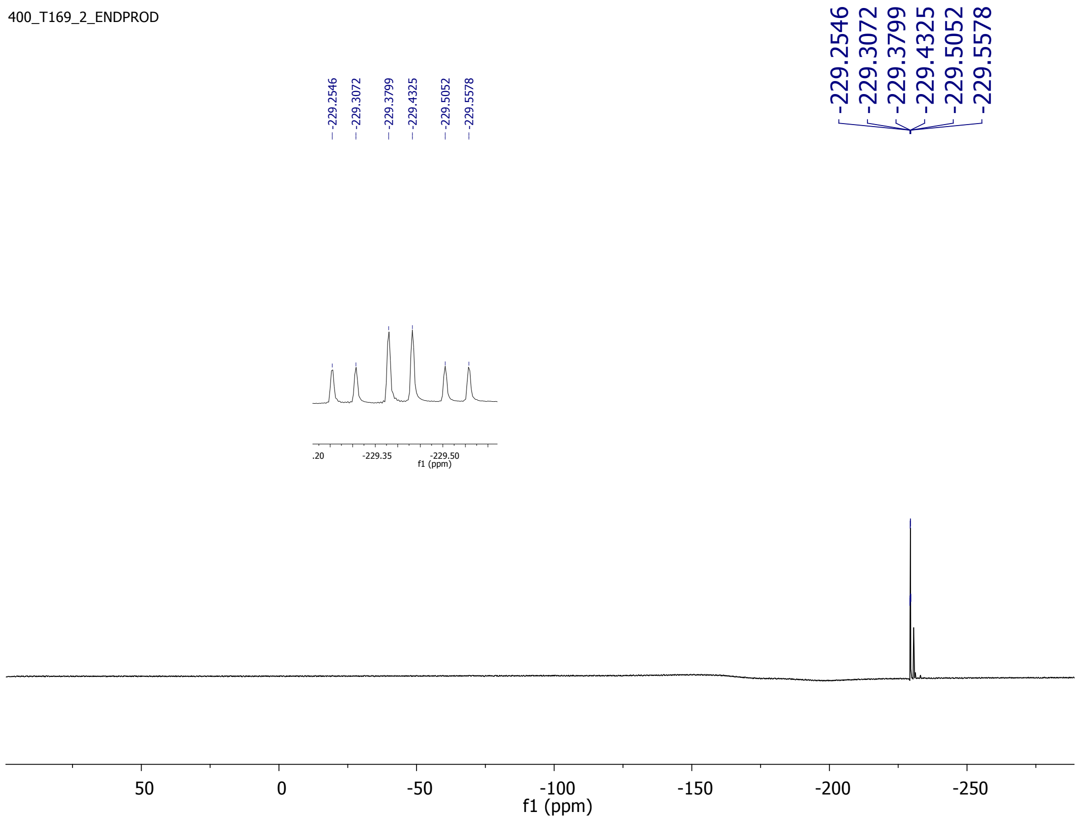

^{13}C NMR of Benzyl 3-(5-(fluoromethyl)-4,5-dihydrooxazol-2-yl)propanoate (2l)

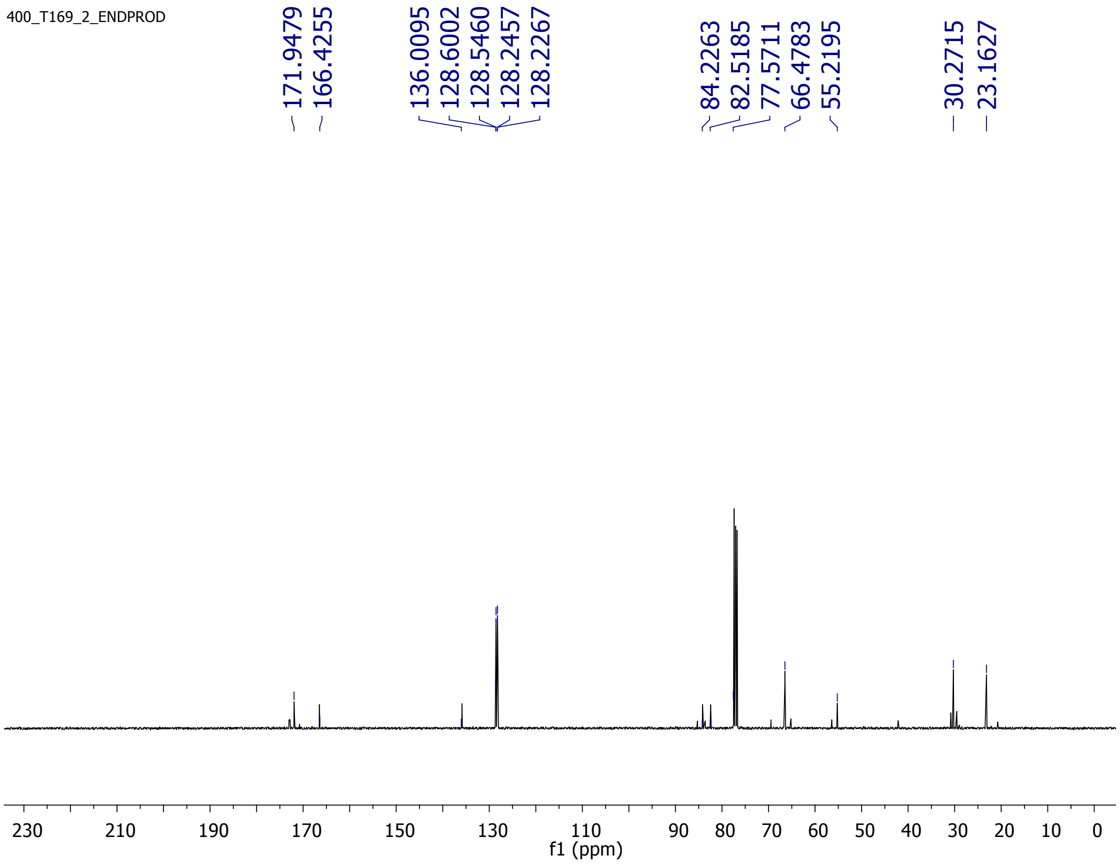

^{19}F NMR of *N*-allyl-2-fluoropiperidine-1-carboxamide (2mb)

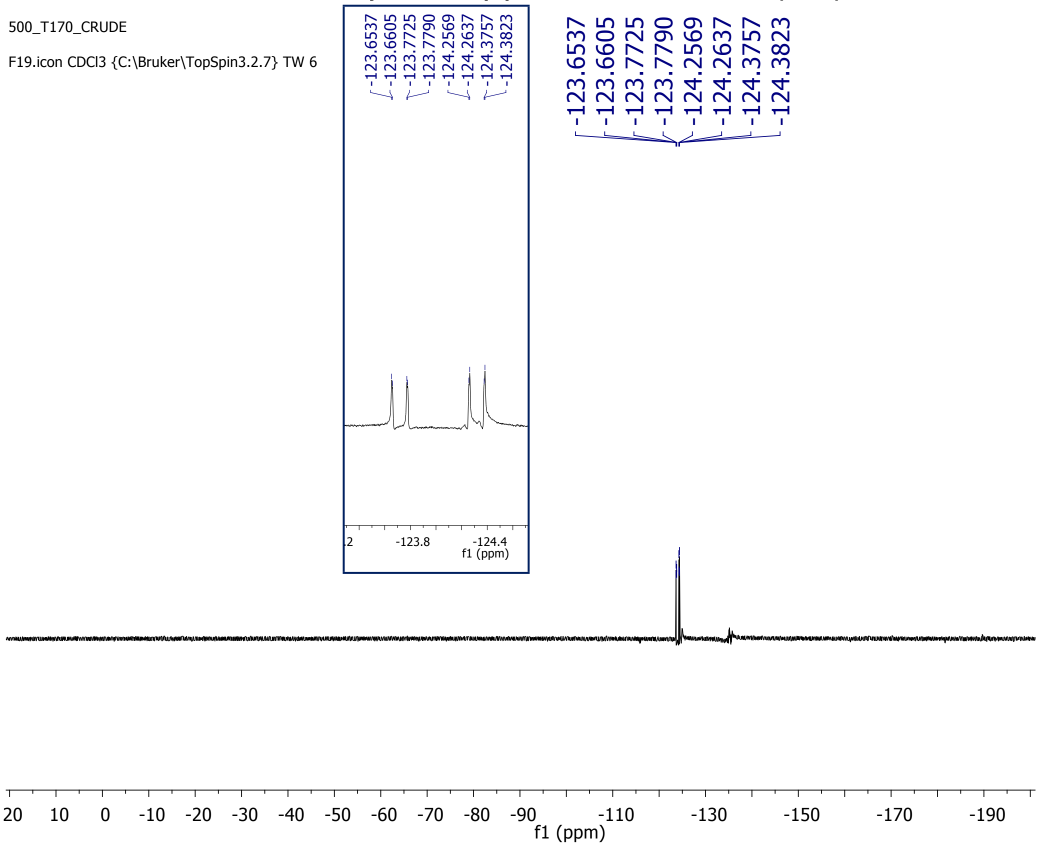

HRMS of *N*-allyl-2-fluoropiperidine-1-carboxamide (2mb)

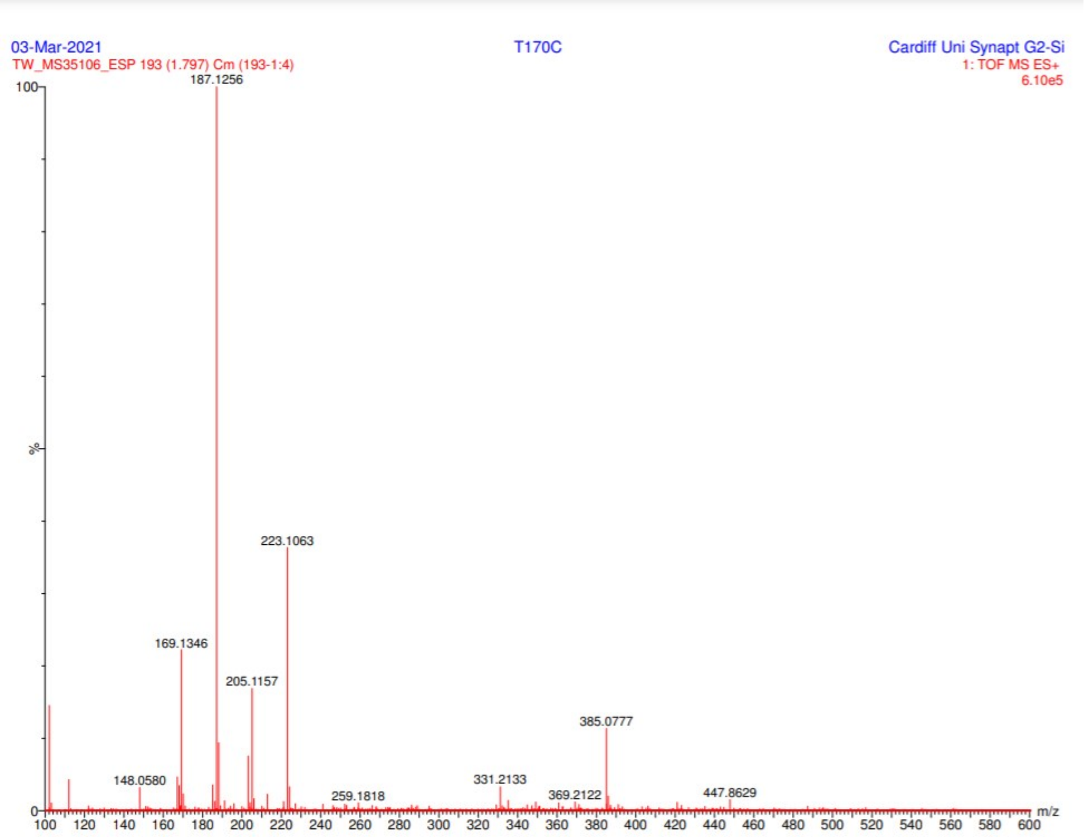

^{1}H NMR of 2-Fluoro-1,3-diphenylpropane-1,3-dione (7a)

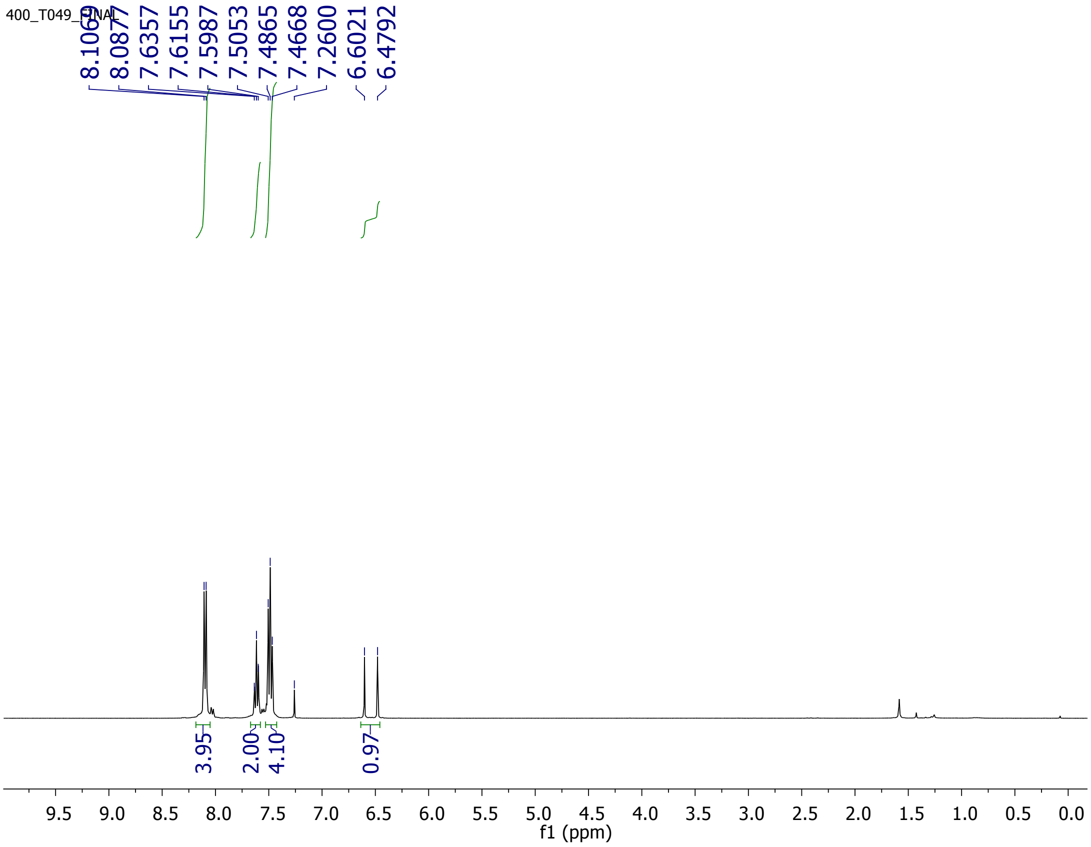

^{19}F NMR of 2-fluoro-1,3-diphenylpropane-1,3-dione (7a)

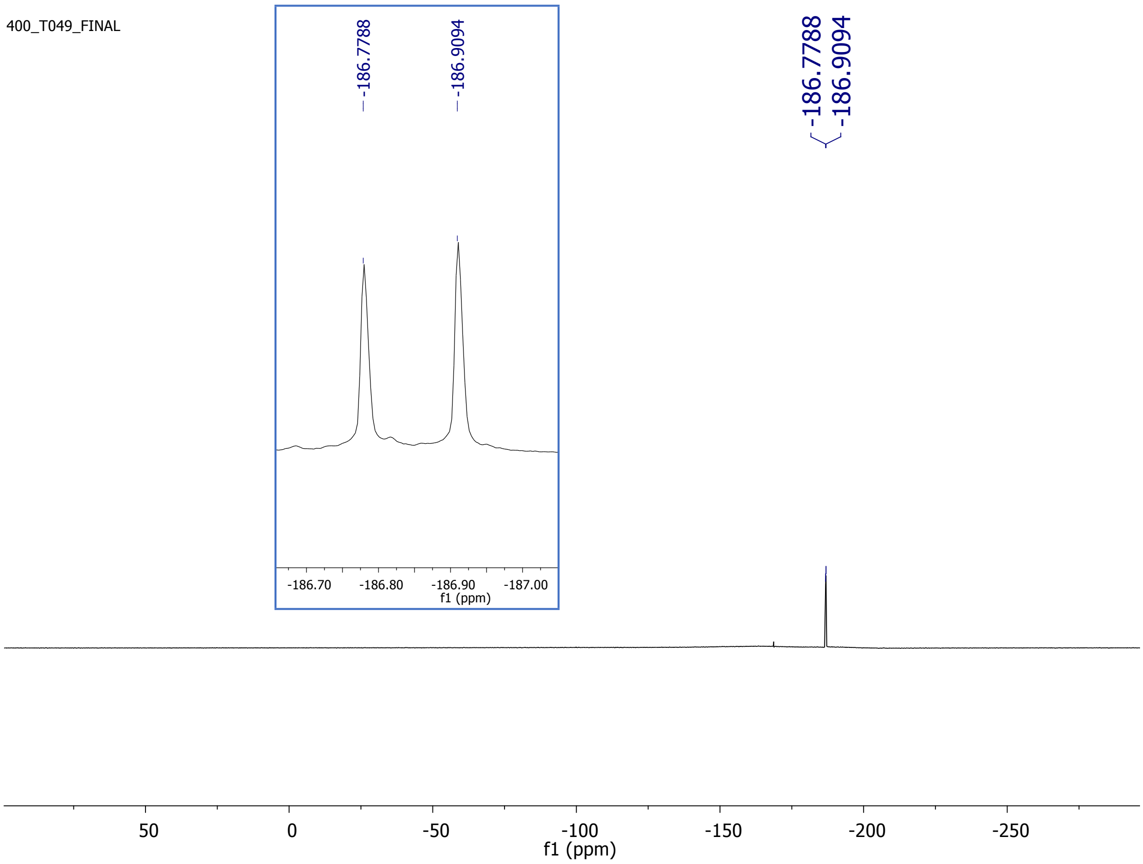

^{13}C NMR of 2-Fluoro-1,3-diphenylpropane-1,3-dione (7a)

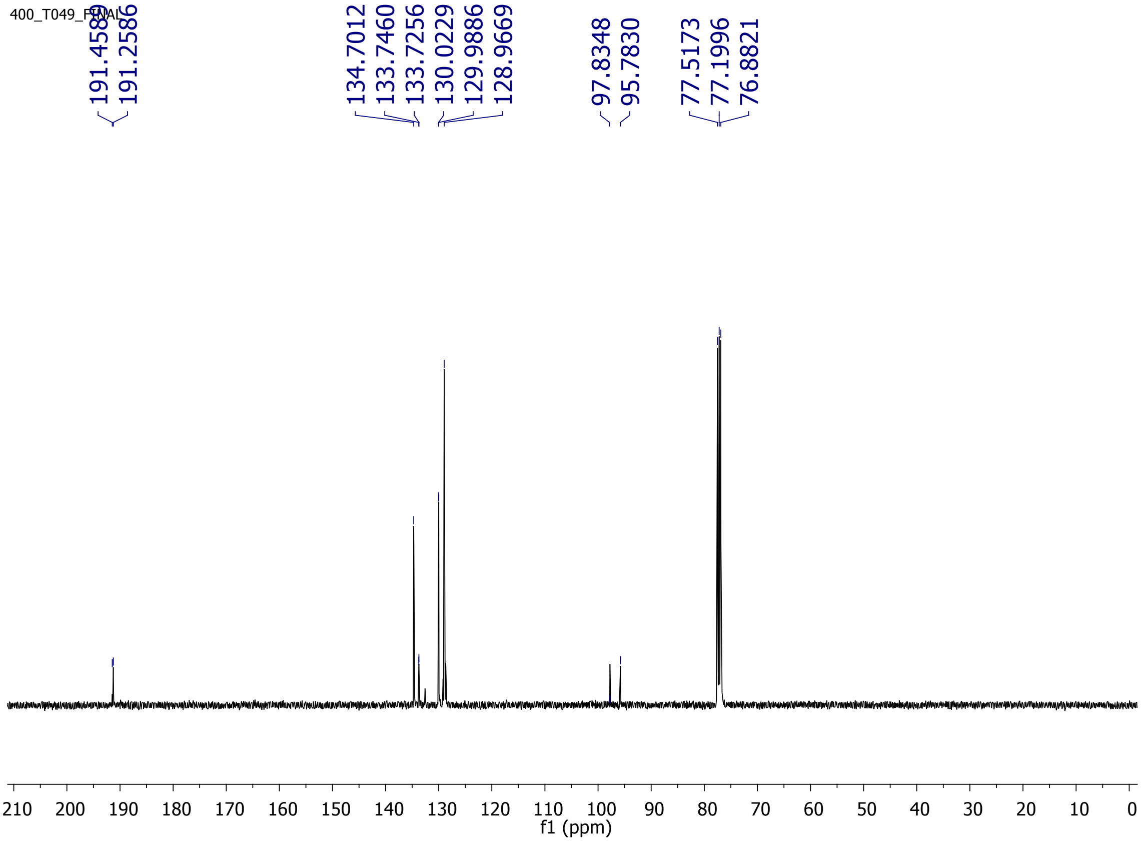

^{1}H NMR of Ethyl 2-fluoro-1-oxo-2,3-dihydro-1H-indene-2-carboxylate (7c)

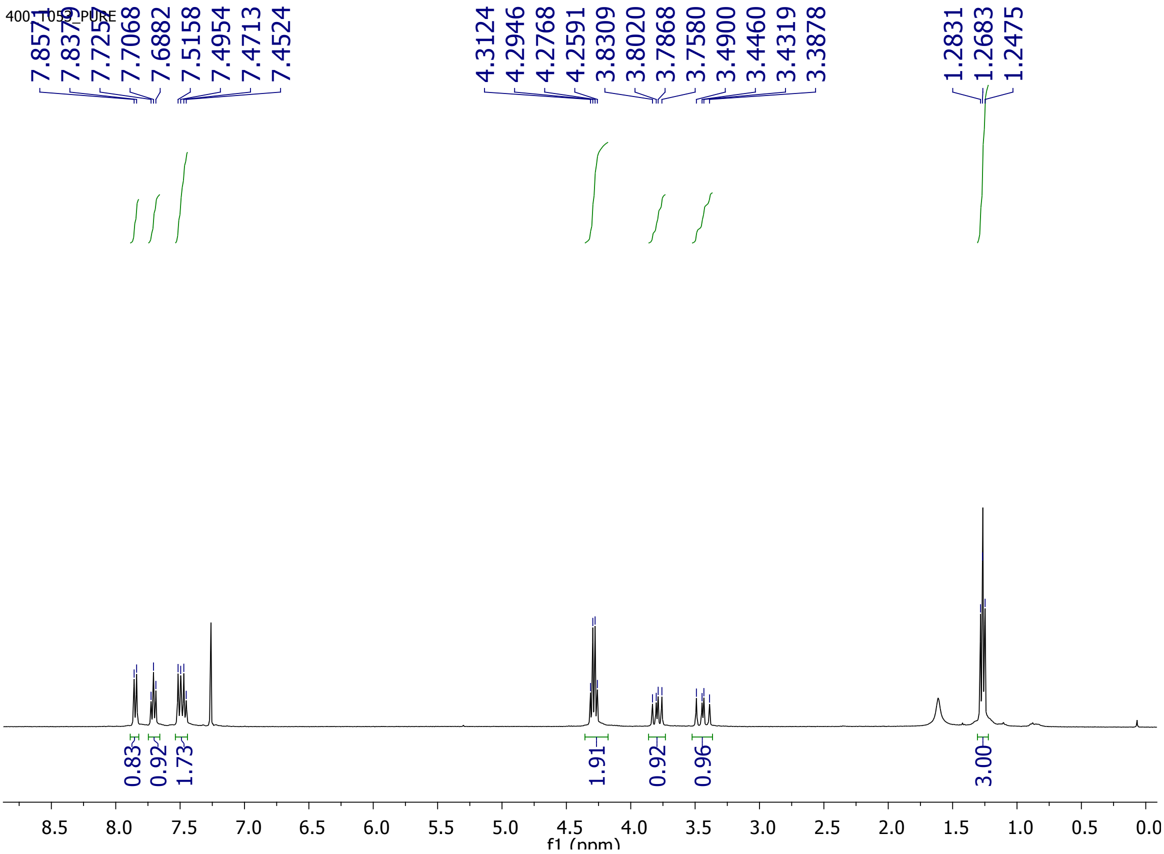

^{19}F NMR of Ethyl 2-fluoro-1-oxo-2,3-dihydro-1H-indene-2-carboxylate (7c)

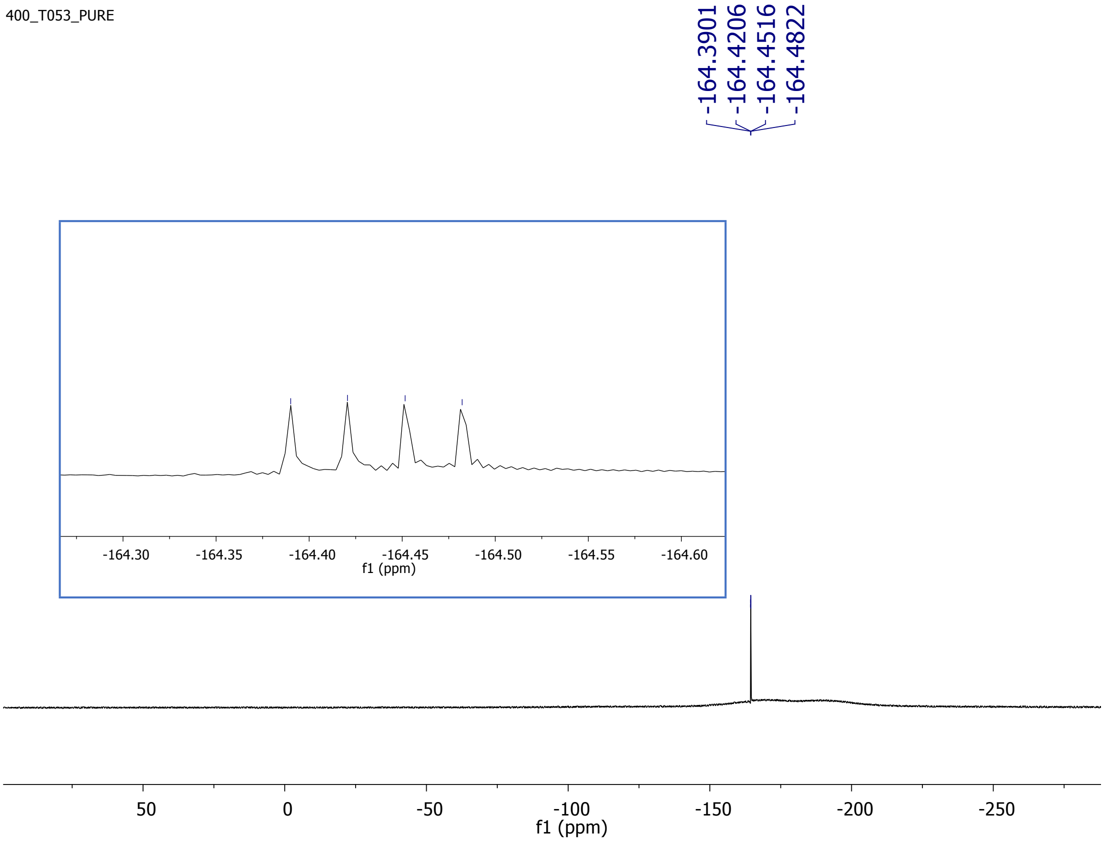

^{13}C NMR of Ethyl 2-fluoro-1-oxo-2,3-dihydro-1H-indene-2-carboxylate (7c)

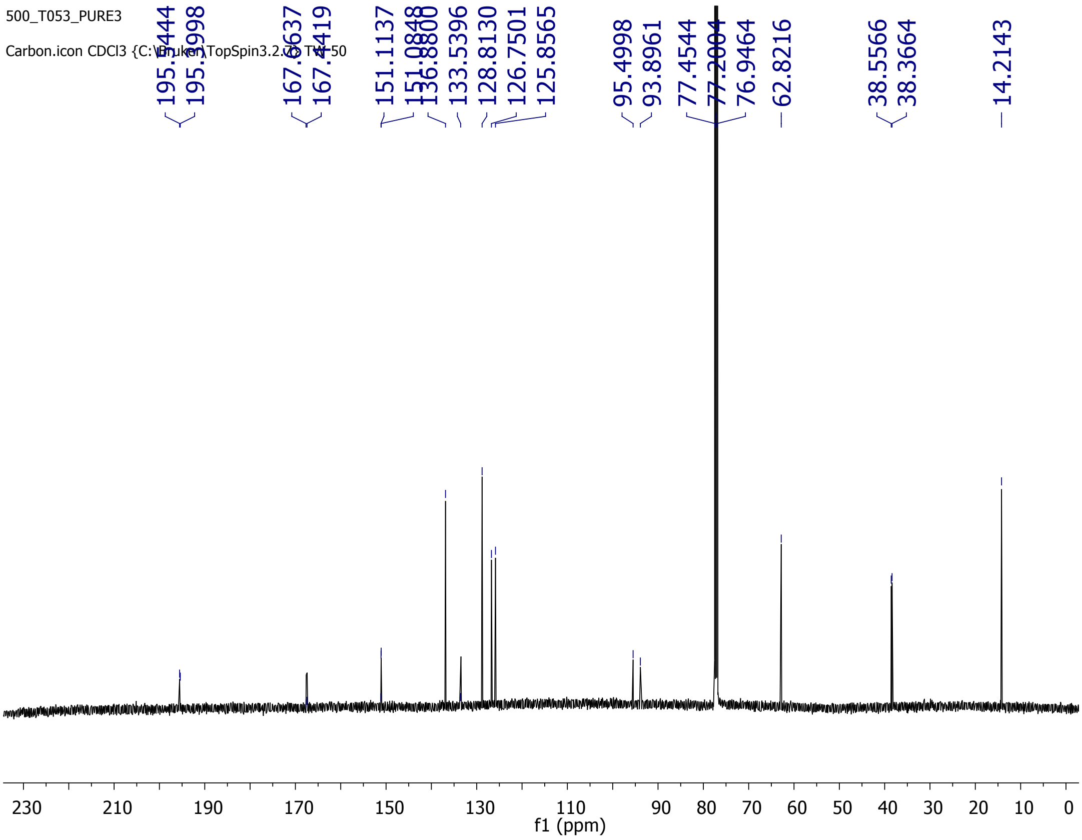

^{1}H NMR of Ethyl 2-fluoro-3-oxo-3-phenylpropionate (7b)

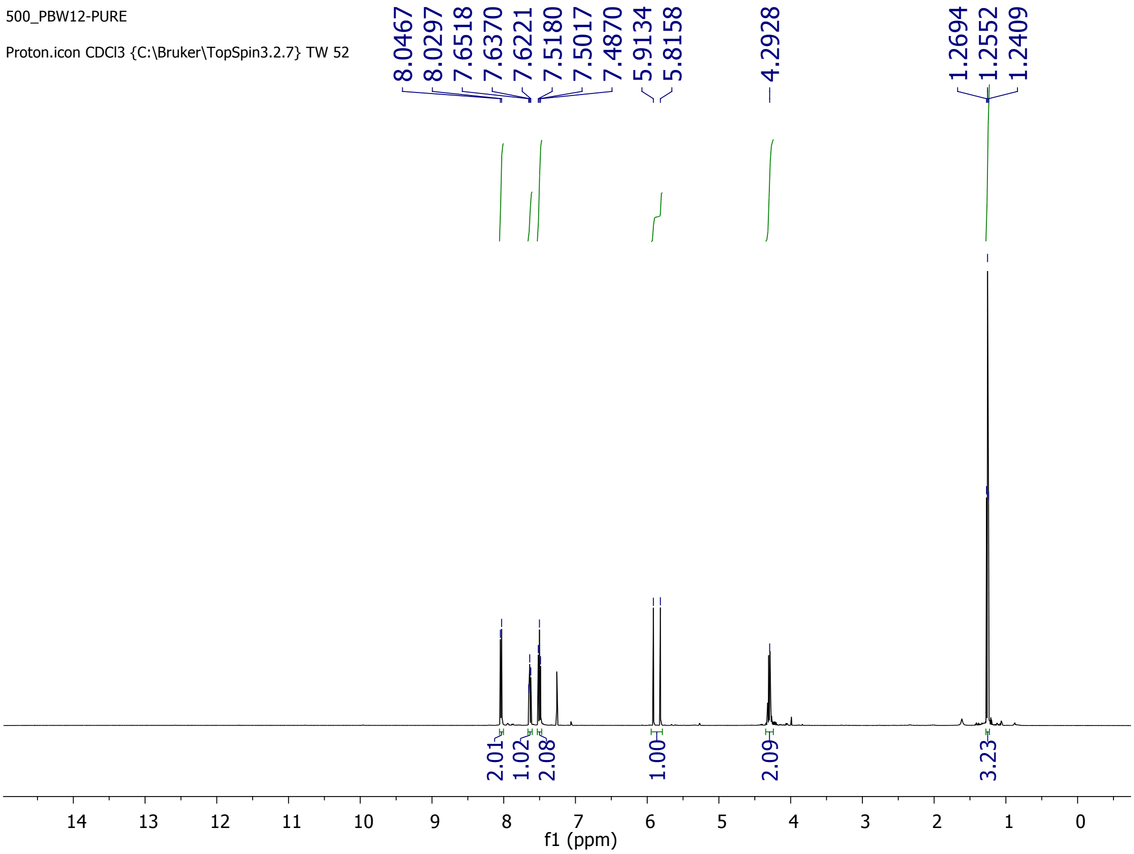

^{13}C NMR of Ethyl 2-fluoro-3-oxo-3-phenylpropionate (7b)

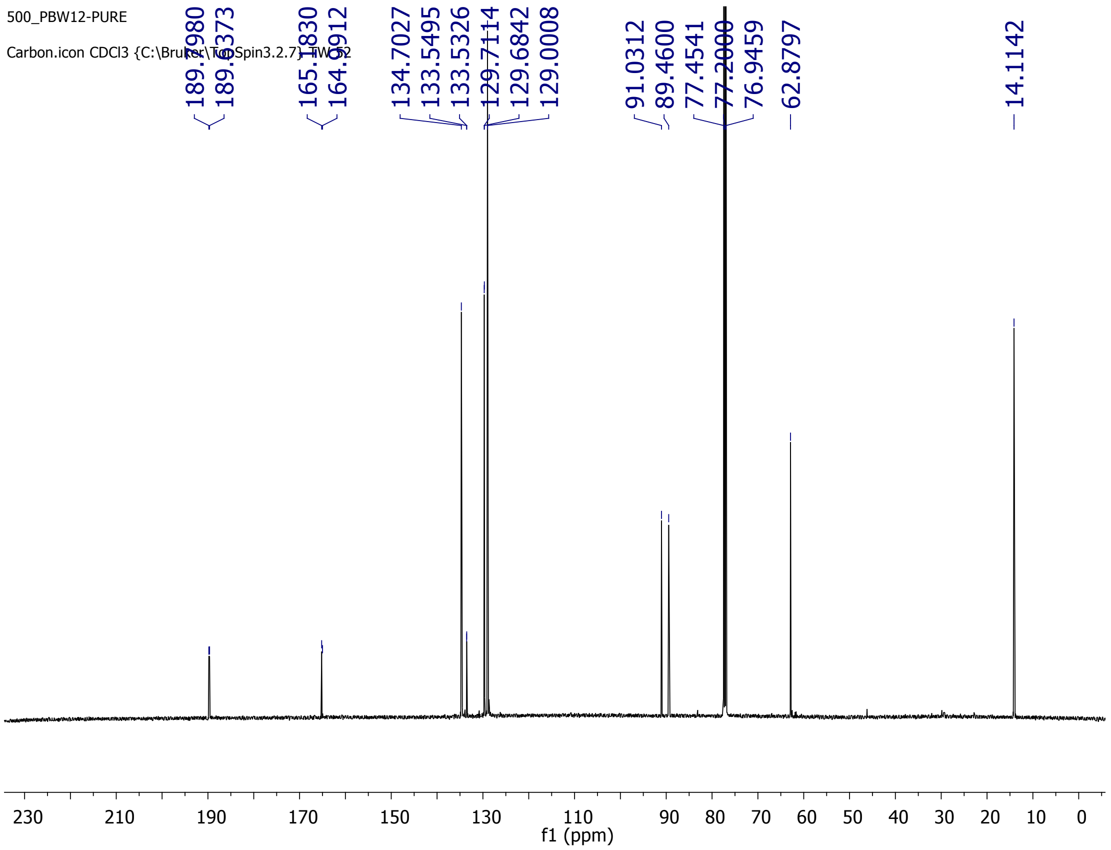

^{19}F NMR of Ethyl 2-fluoro-3-oxo-3-phenylpropionate (7b)

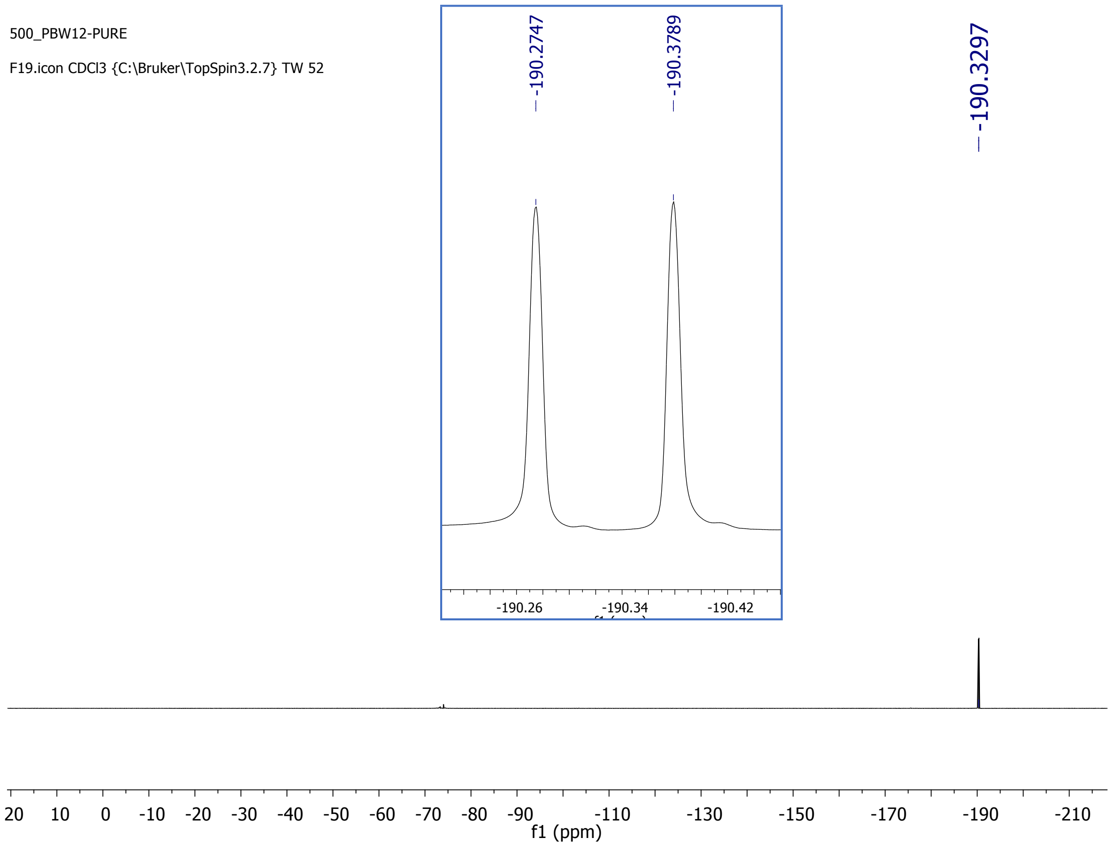

^{1}H NMR of methyl 2-fluoro-1-oxo-2,3-dihydro-1H-indene-2-carboxylate (7d)

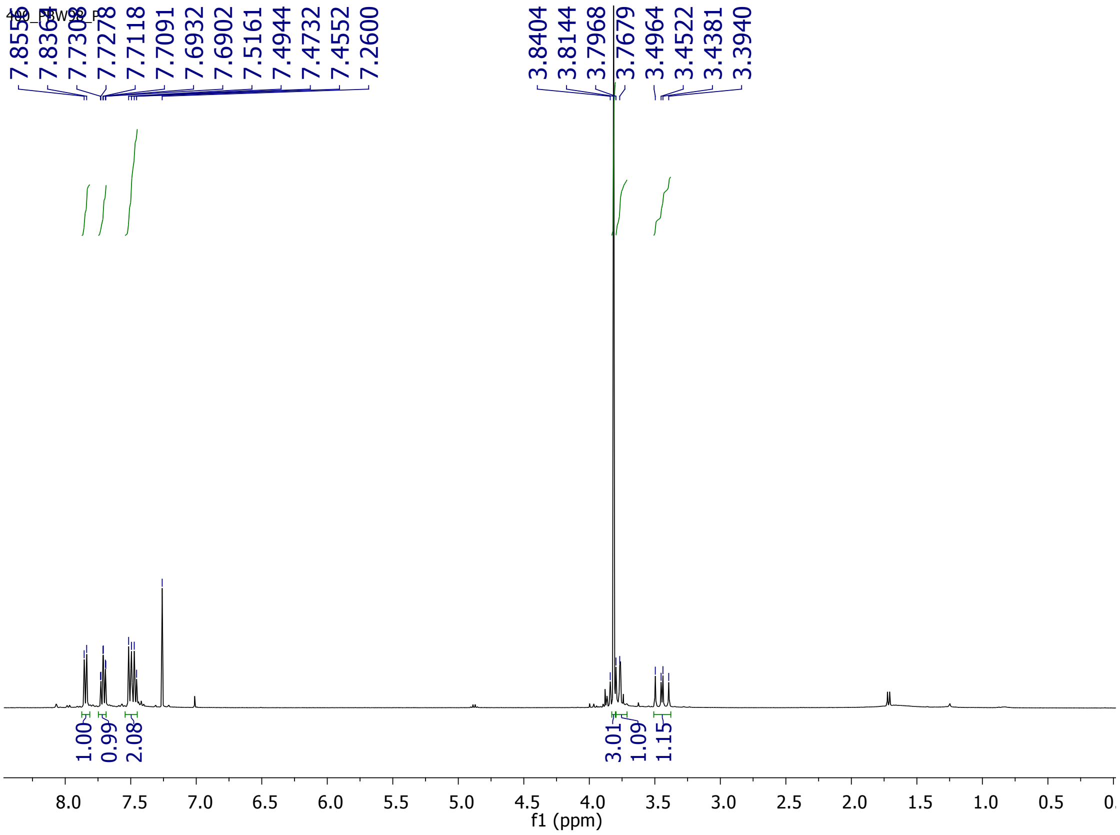

^{19}F NMR of methyl 2-fluoro-1-oxo-2,3-dihydro-1H-indene-2-carboxylate (7d)

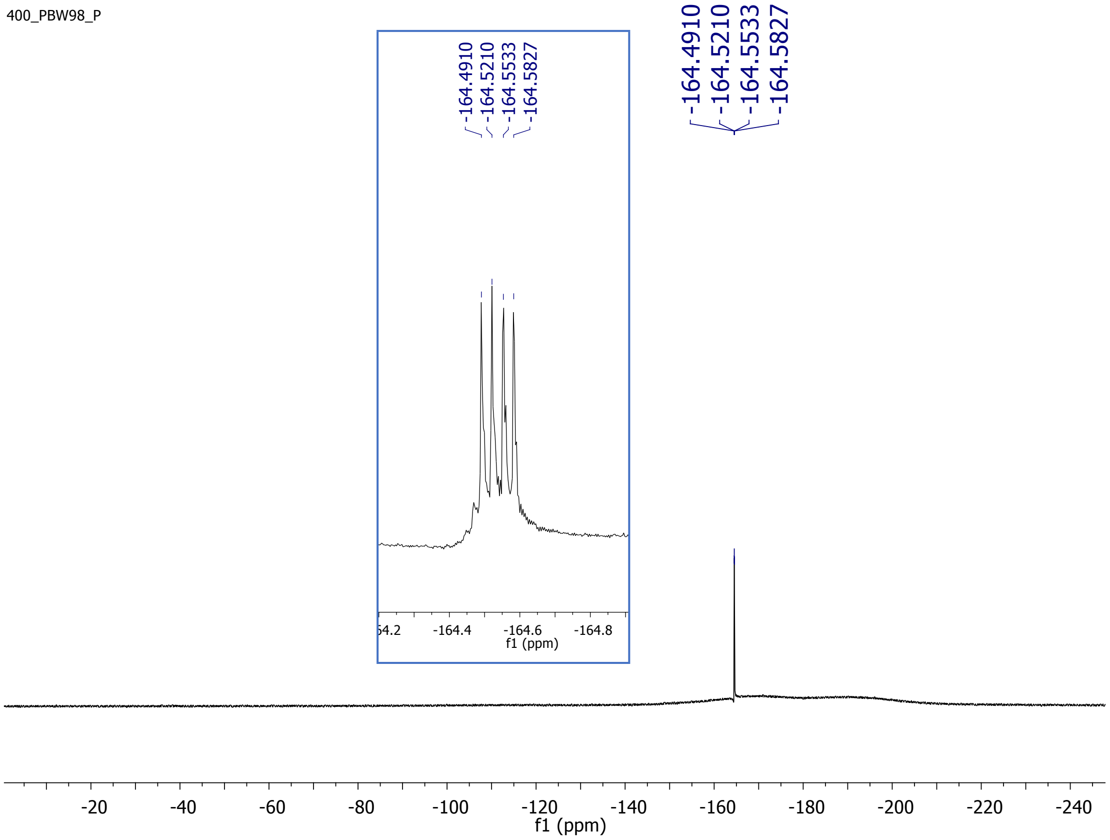

^{13}C NMR of methyl 2-fluoro-1-oxo-2,3-dihydro-1H-indene-2-carboxylate (7d)

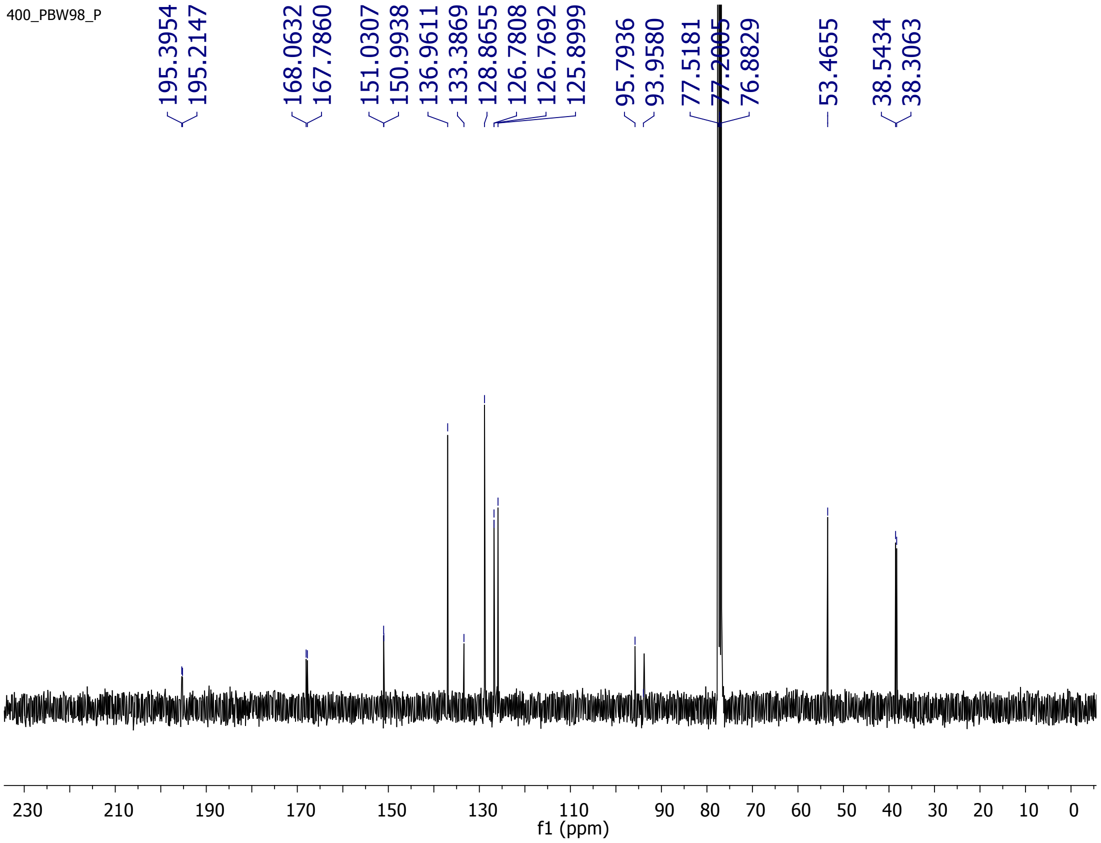

^{1}H NMR of 2-Fluoro-1-phenyl-1-propanone (8a)

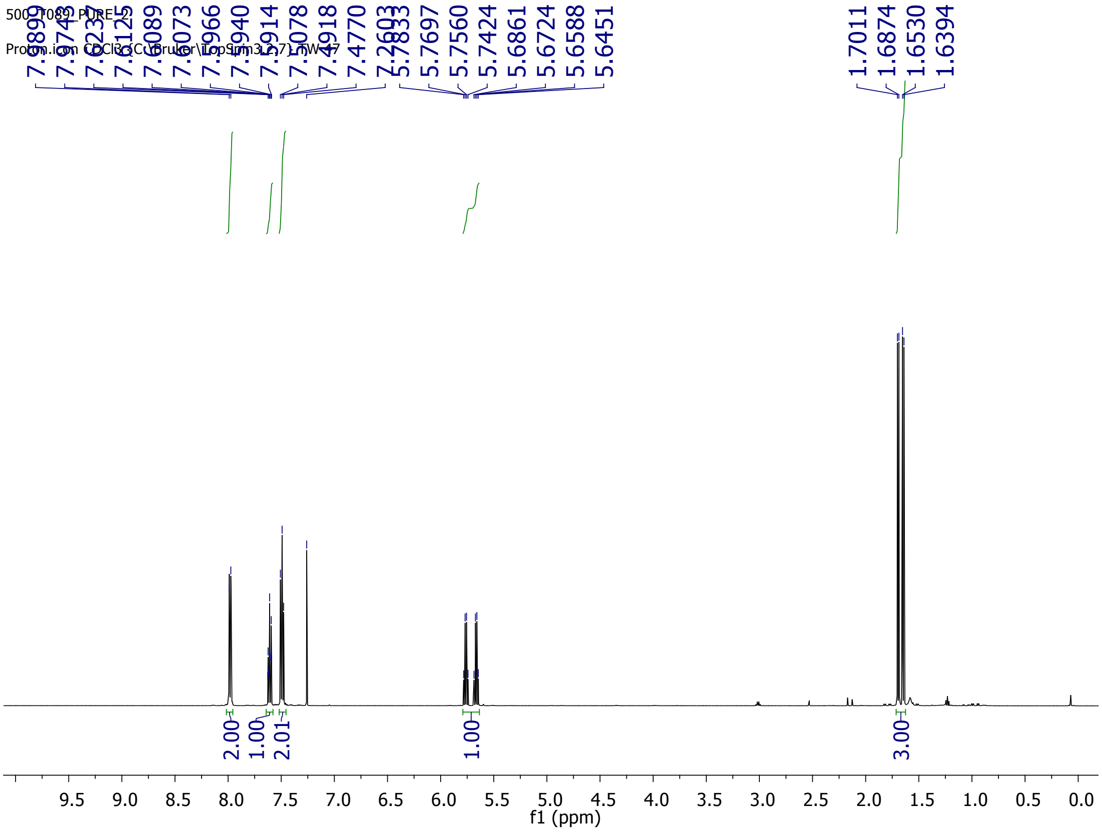

^{19}F NMR of 2-Fluoro-1-phenyl-1-propanone (8a)

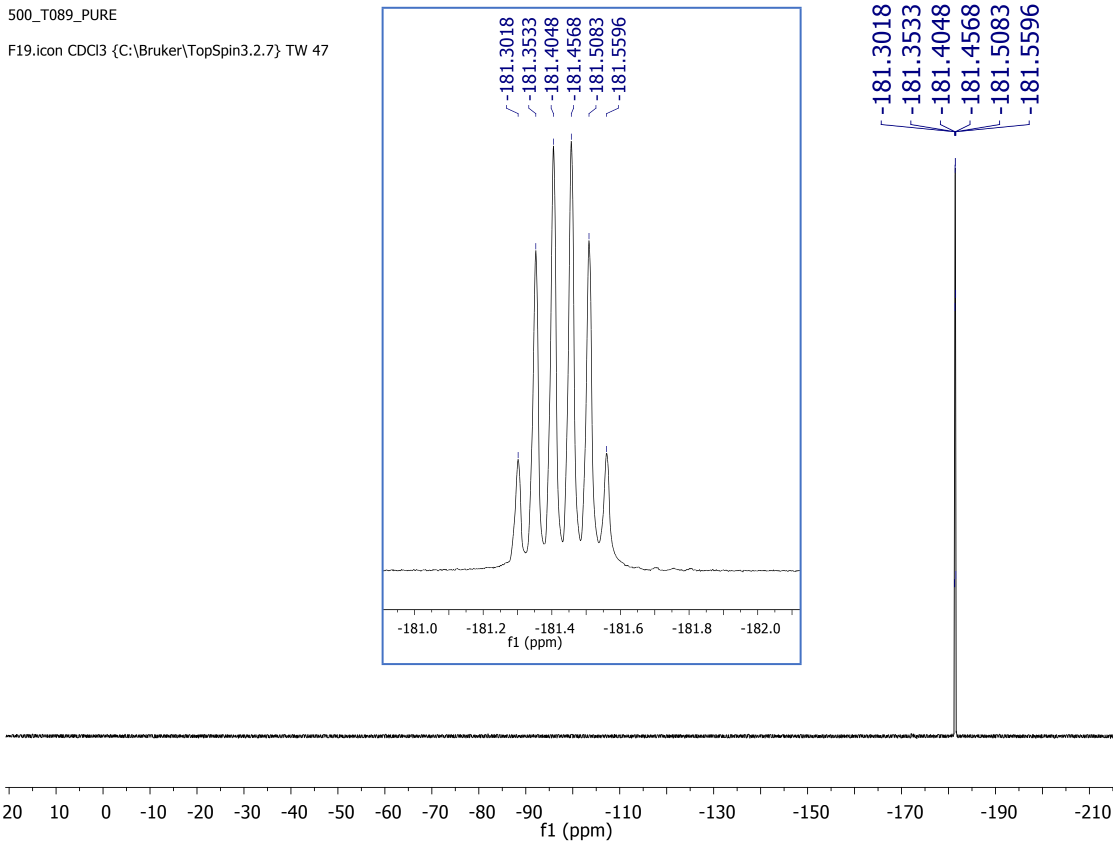

^{13}C NMR of 2-Fluoro-1-phenyl-1-propanone (8a)

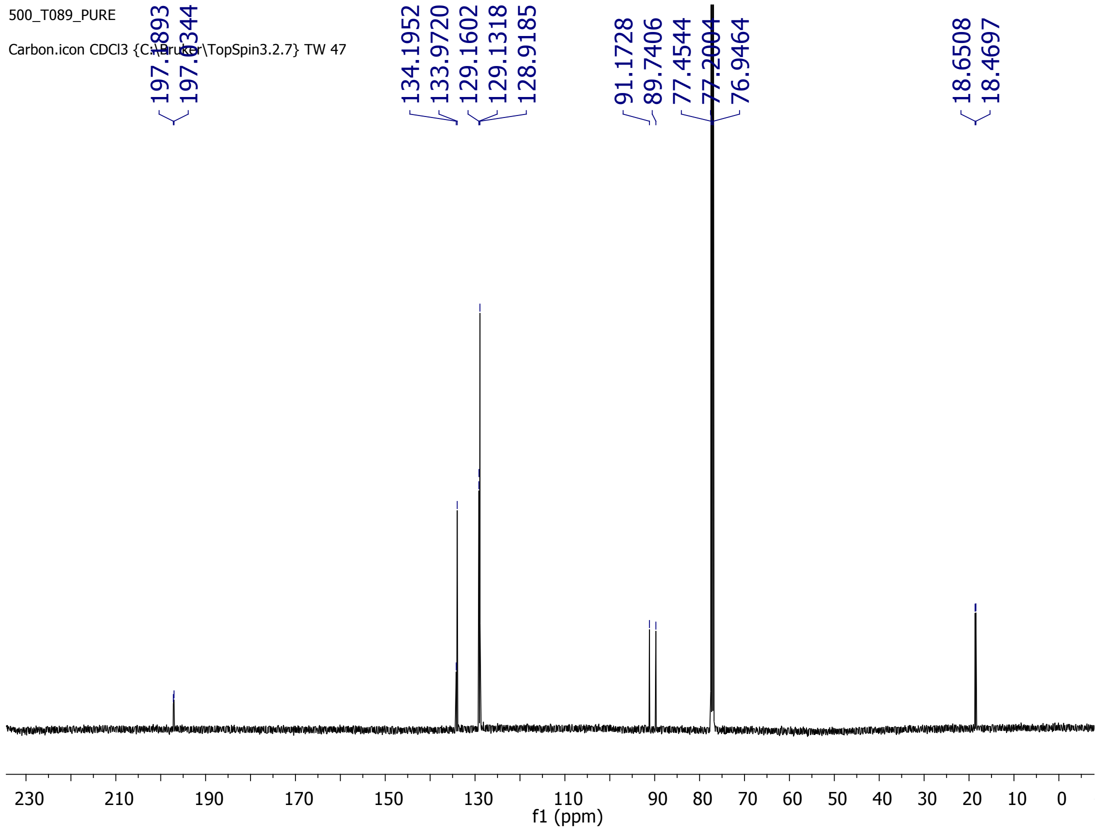

^{1}H NMR of 2-Fluoro-1-(1-naphthyl)ethenone (8b)

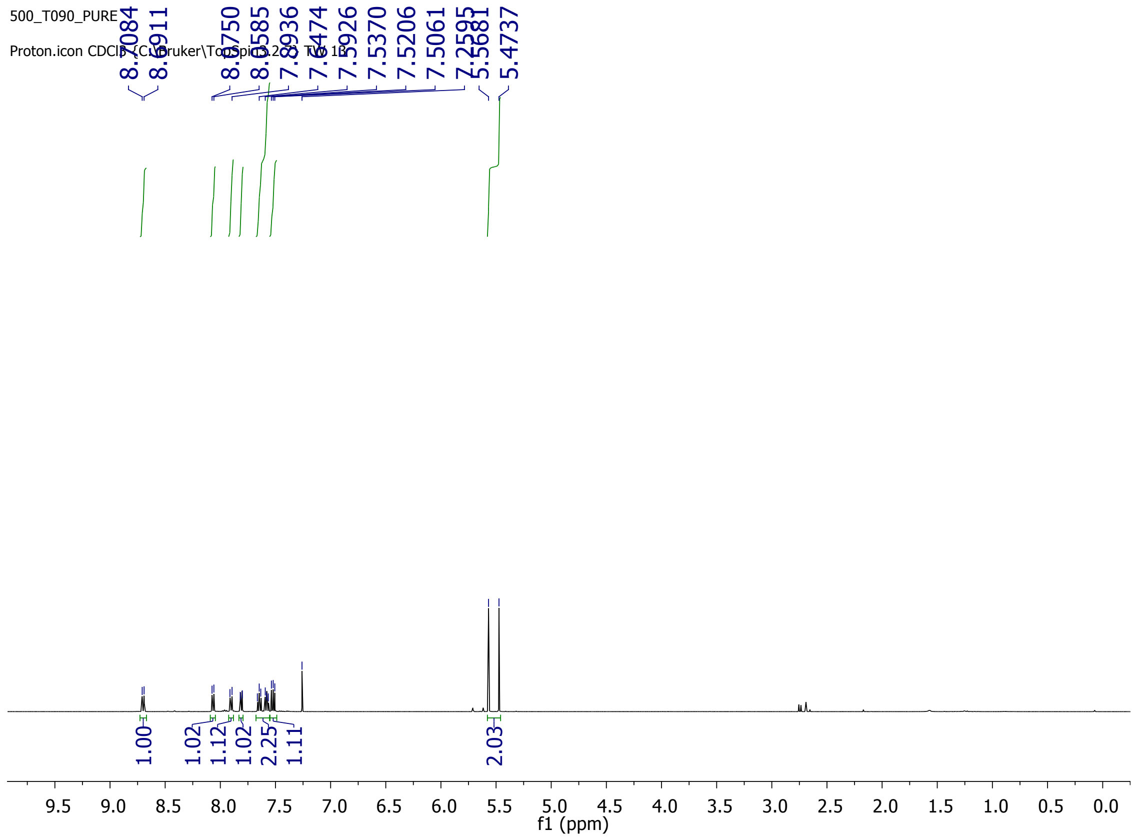

^{19}F NMR of 2-Fluoro-1-(1-naphthyl)ethenone (8b)

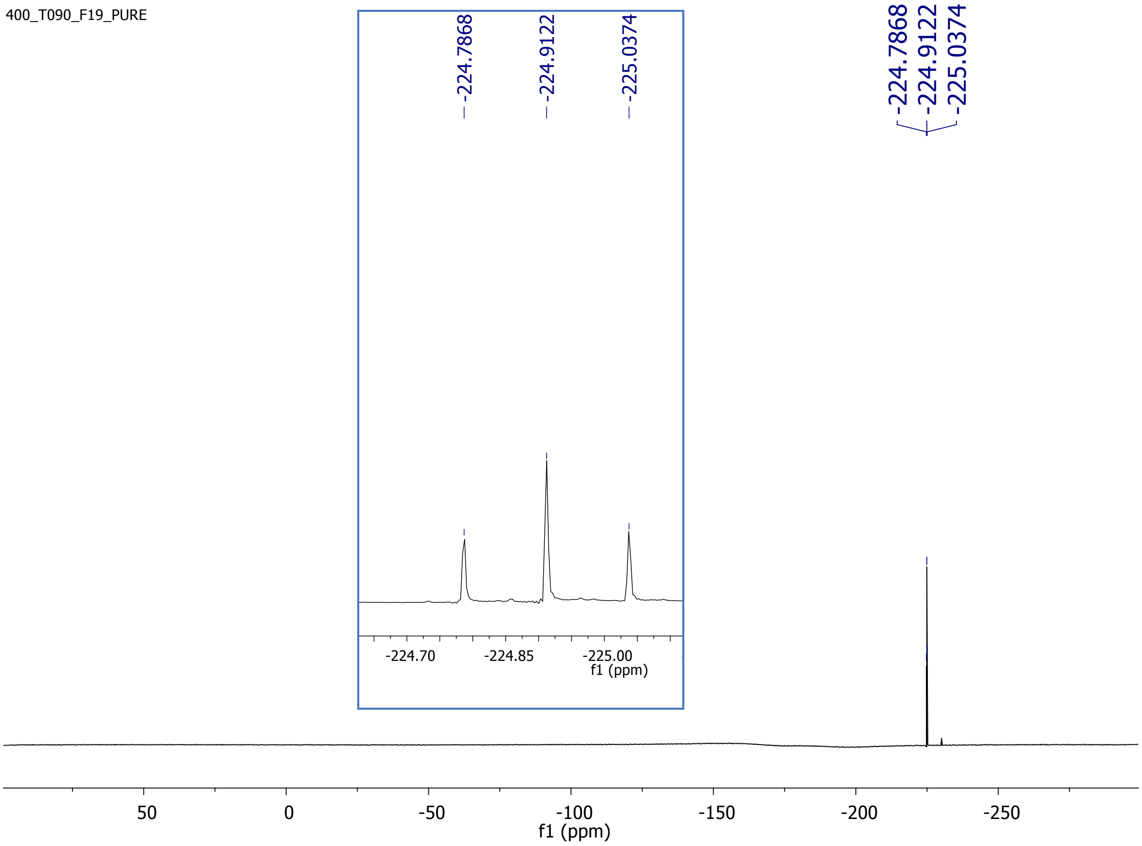

^{13}C NMR of 2-Fluoro-1-(1-naphthyl)ethenone (8b)

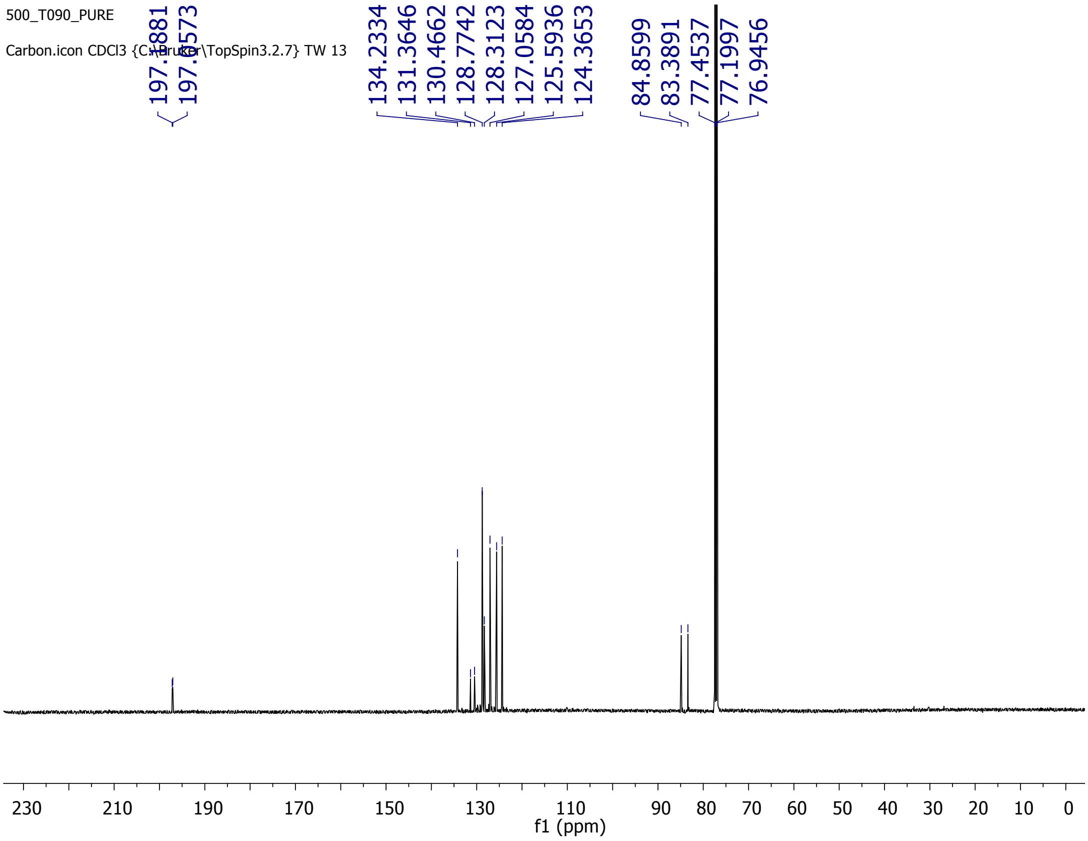

^{1}H NMR of 2-Fluoro-1-indanone (8c)

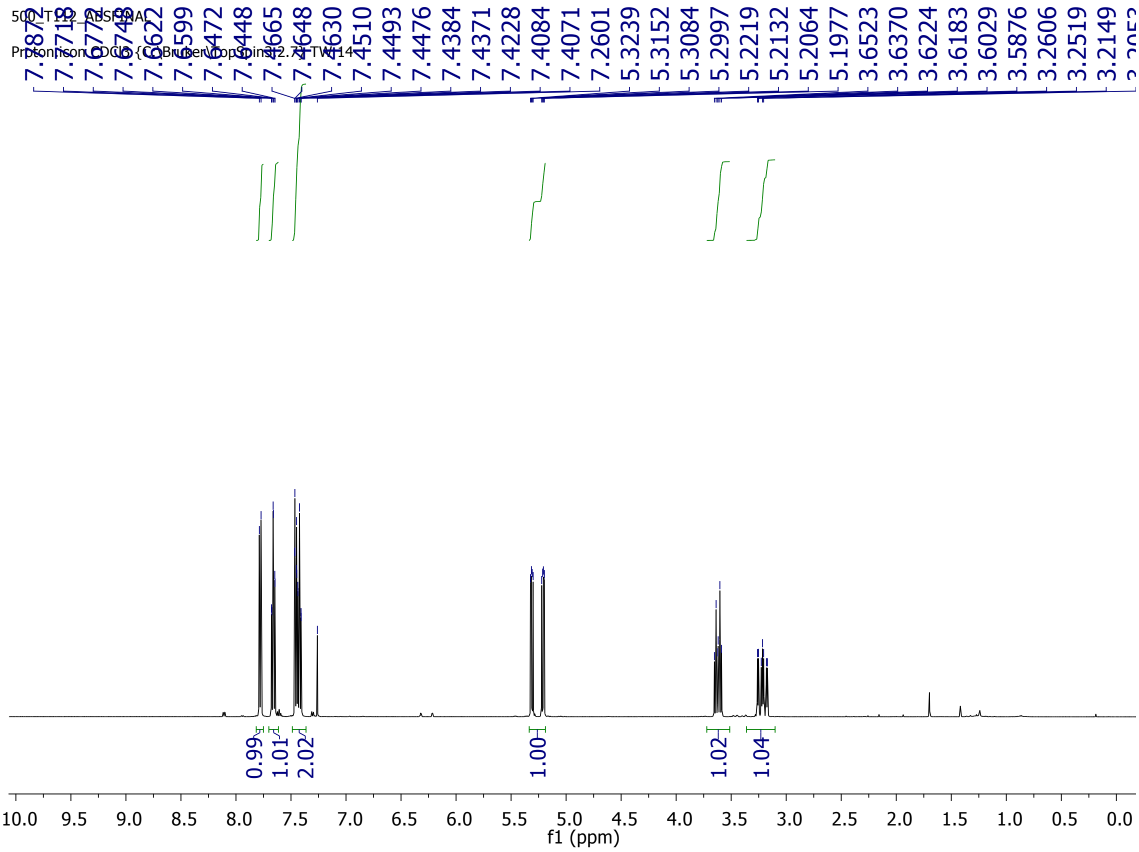

^{19}F NMR of 2-Fluoro-1-indanone (8c)

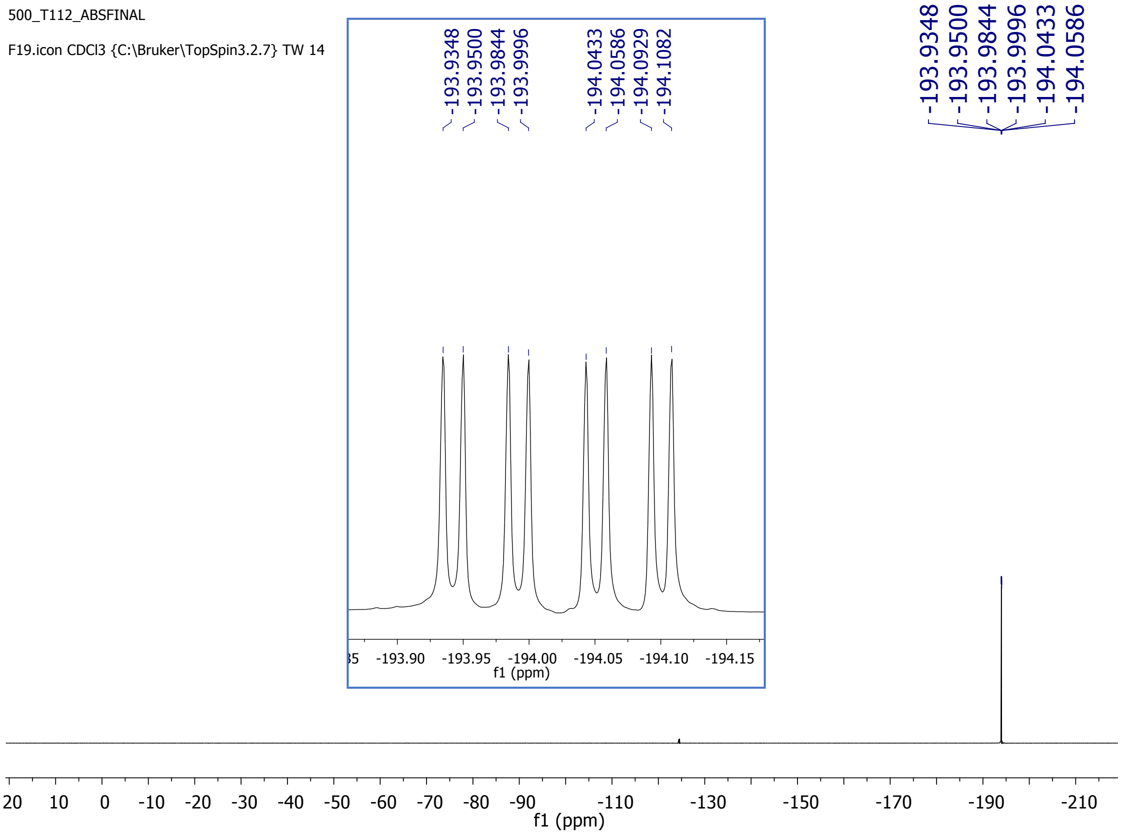

^{13}C NMR of 2-Fluoro-1-indanone (8c)

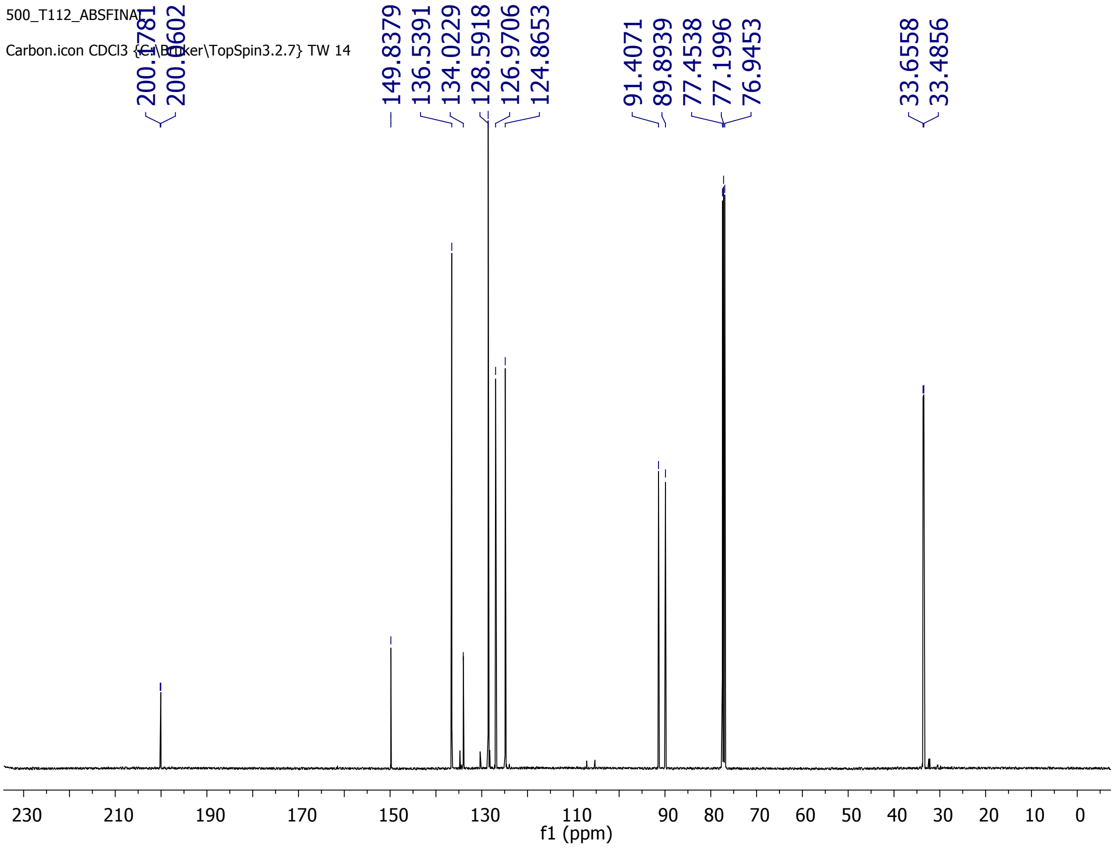

^{1}H NMR of 1-((2,3-Difluoropropoxy)methyl)-4-fluorobenzene (6a)

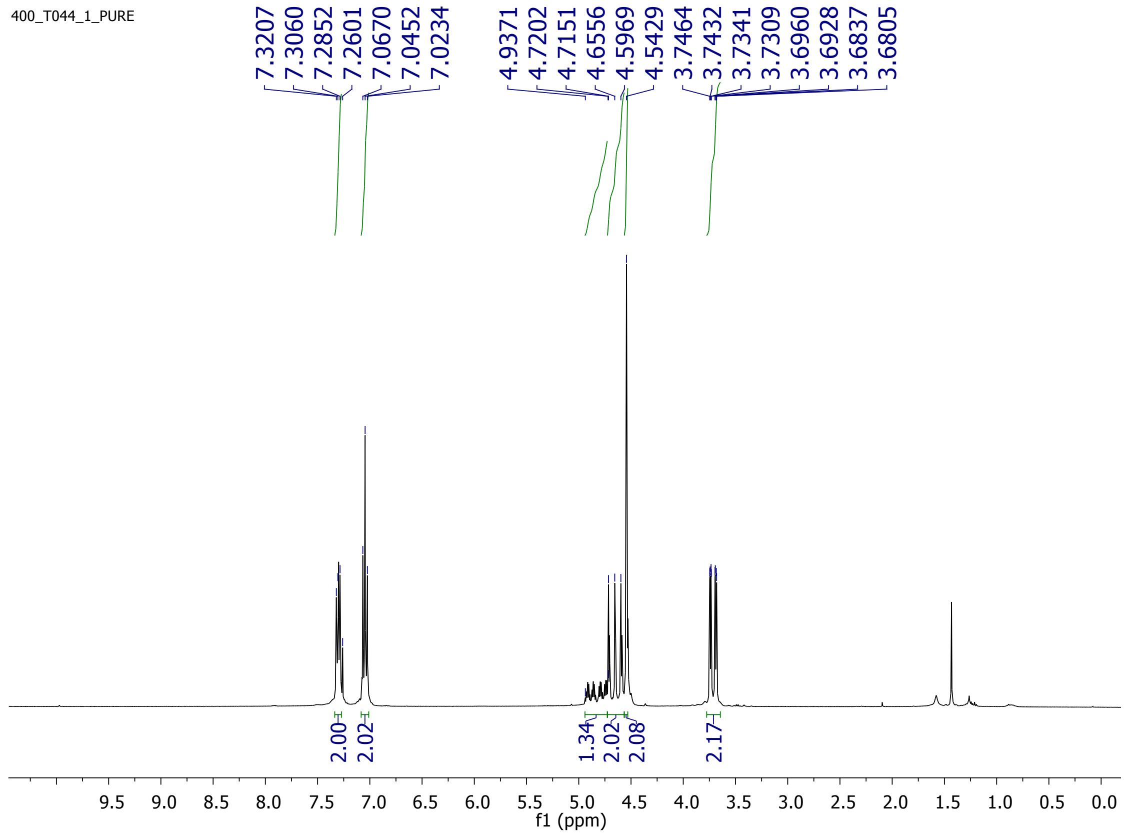

^{13}C NMR of 1-((2,3-Difluoropropoxy)methyl)-4-fluorobenzene (6a)

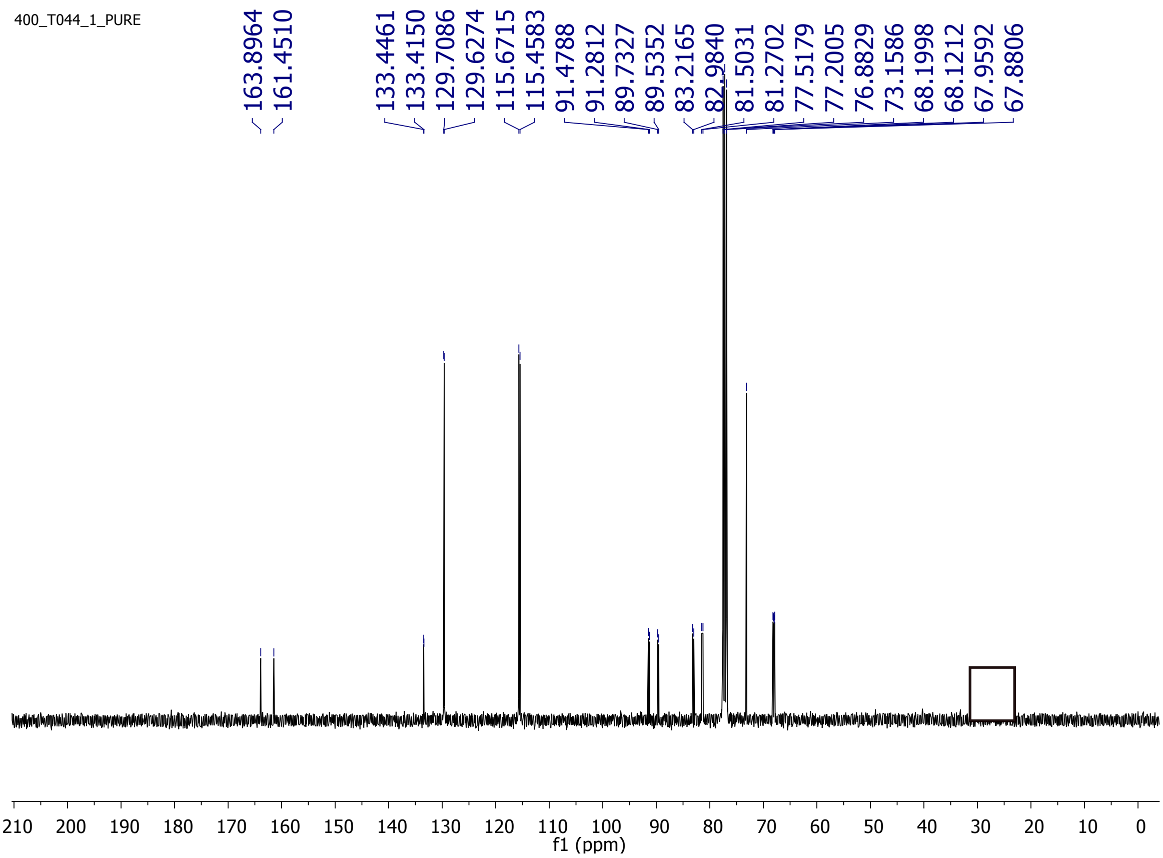

^{19}F NMR of 1-((2,3-Difluoropropoxy)methyl)-4-fluorobenzene (6a)

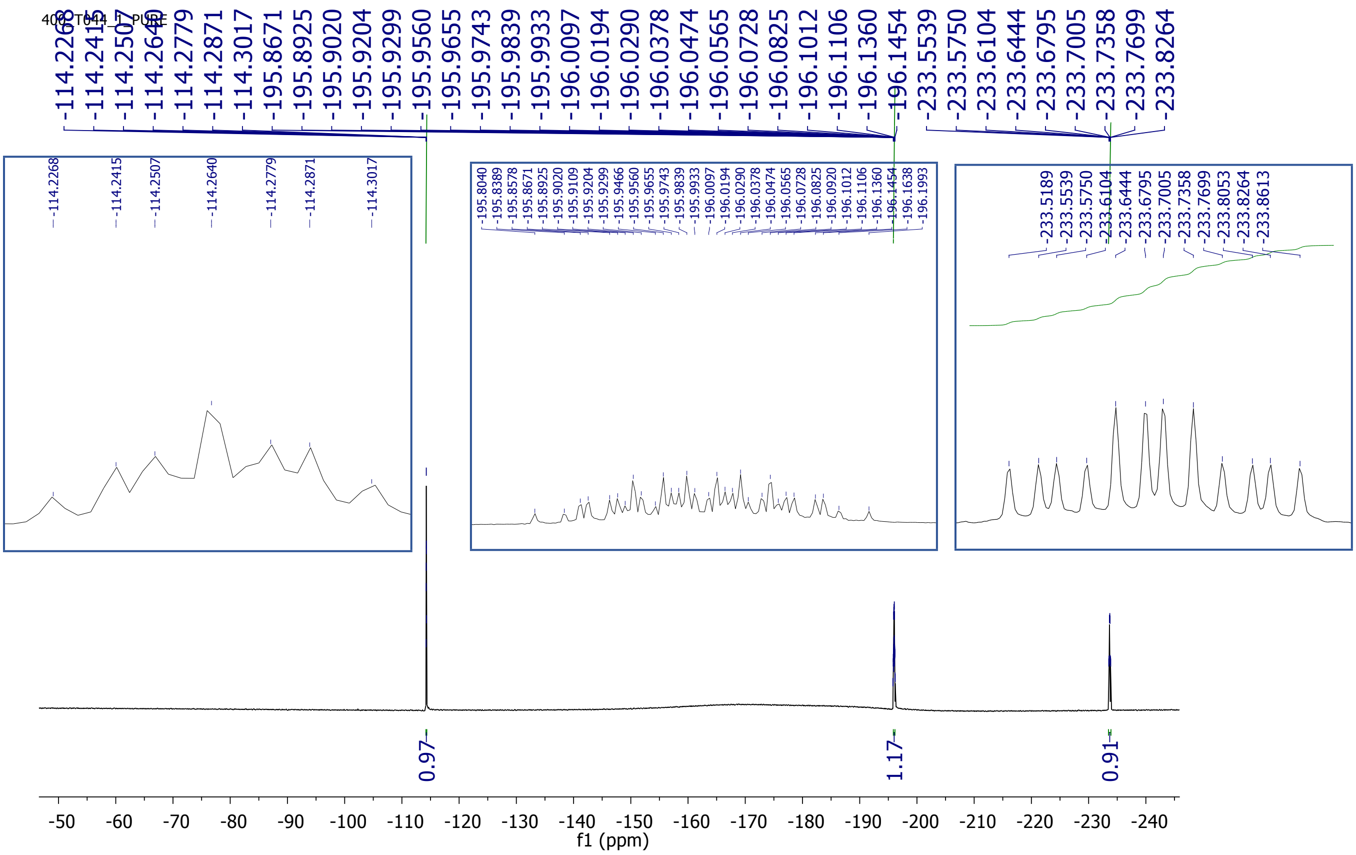

^{1}H NMR of 1-((2,3-Difluoropropoxy)methyl)-3,5-dinitrobenzene (6b)

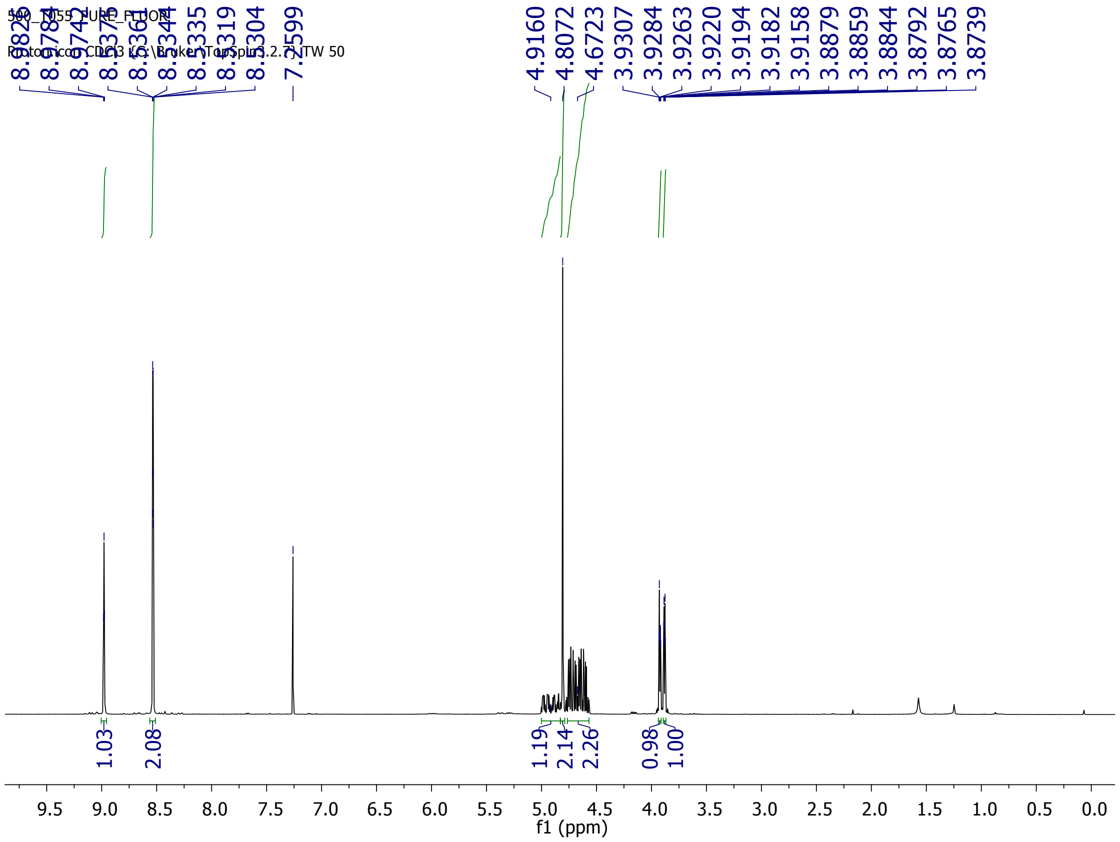

^{19}F NMR of 1-((2,3-Difluoropropoxy)methyl)-3,5-dinitrobenzene (6b)

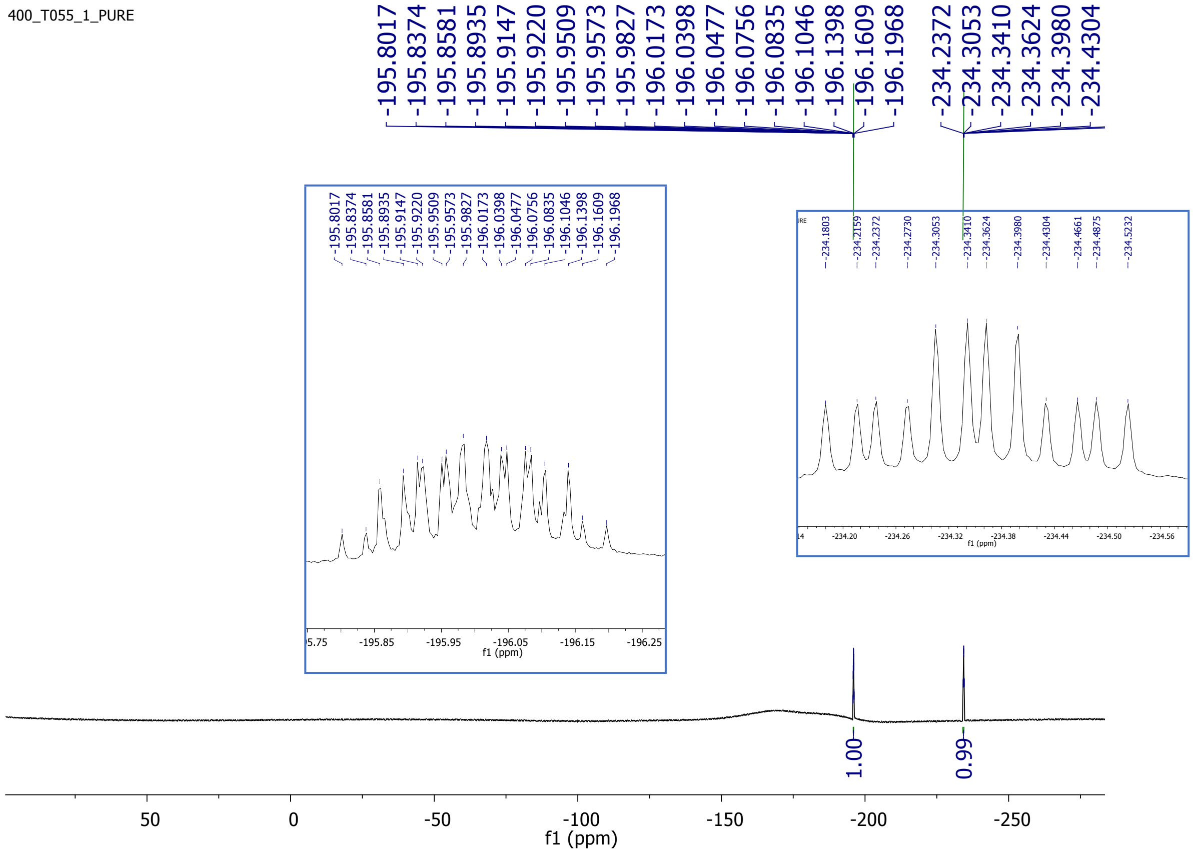

^{13}C NMR of 1-((2,3-Difluoropropoxy)methyl)-3,5-dinitrobenzene (6b)

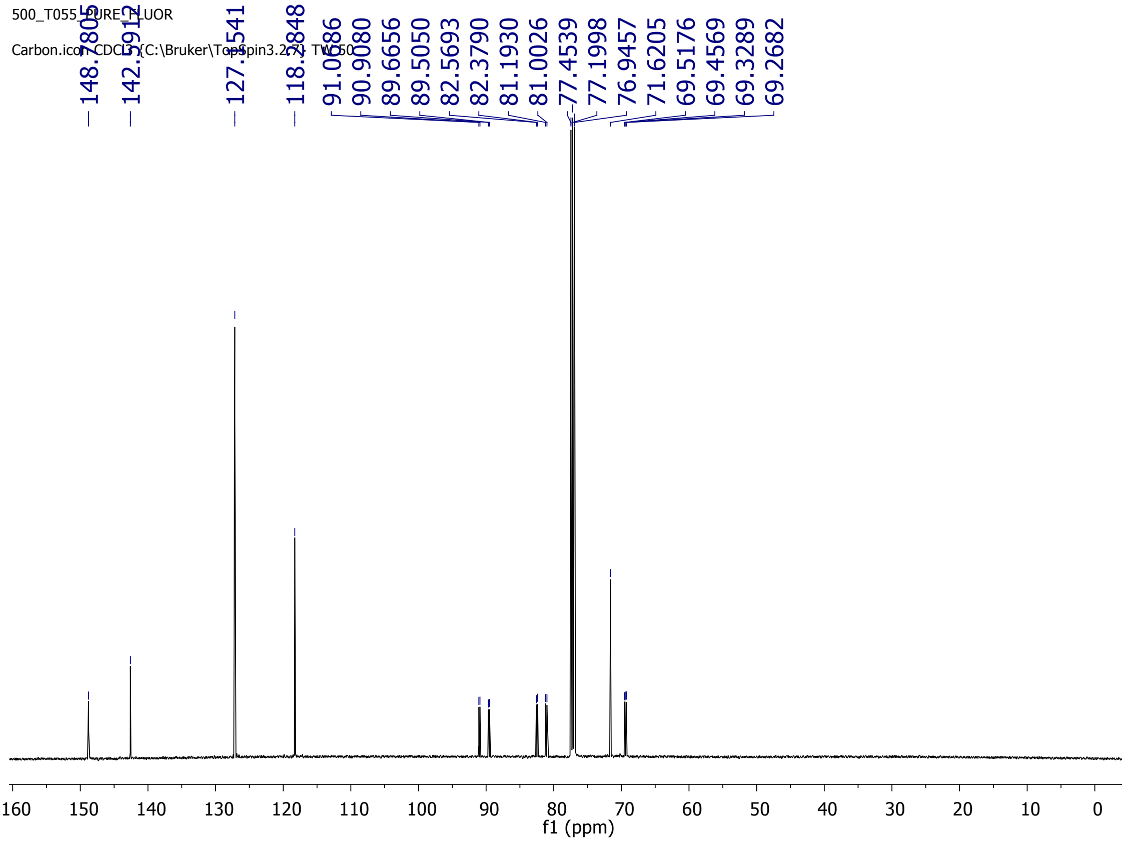

^{1}H NMR of 1-Bromo-2-((2,3-difluoropropoxy)methyl)benzene (6d)

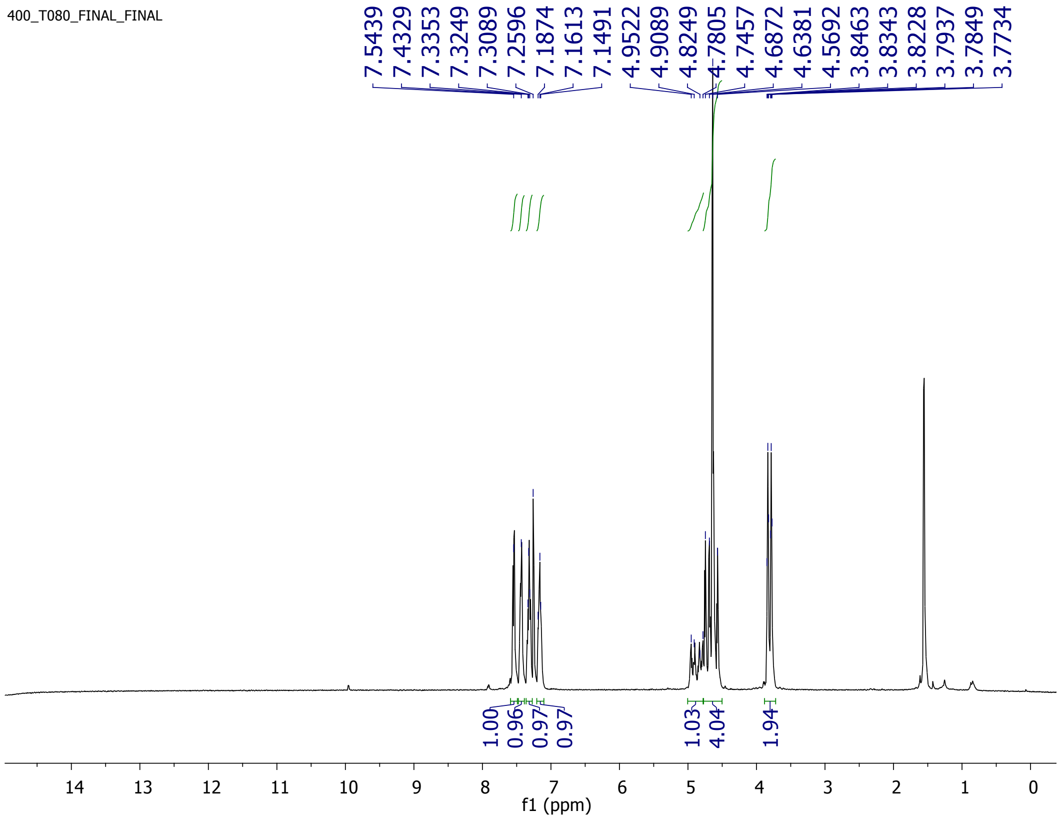

^{19}F NMR of 1-Bromo-2-((2,3-difluoropropoxy)methyl)benzene (6d)

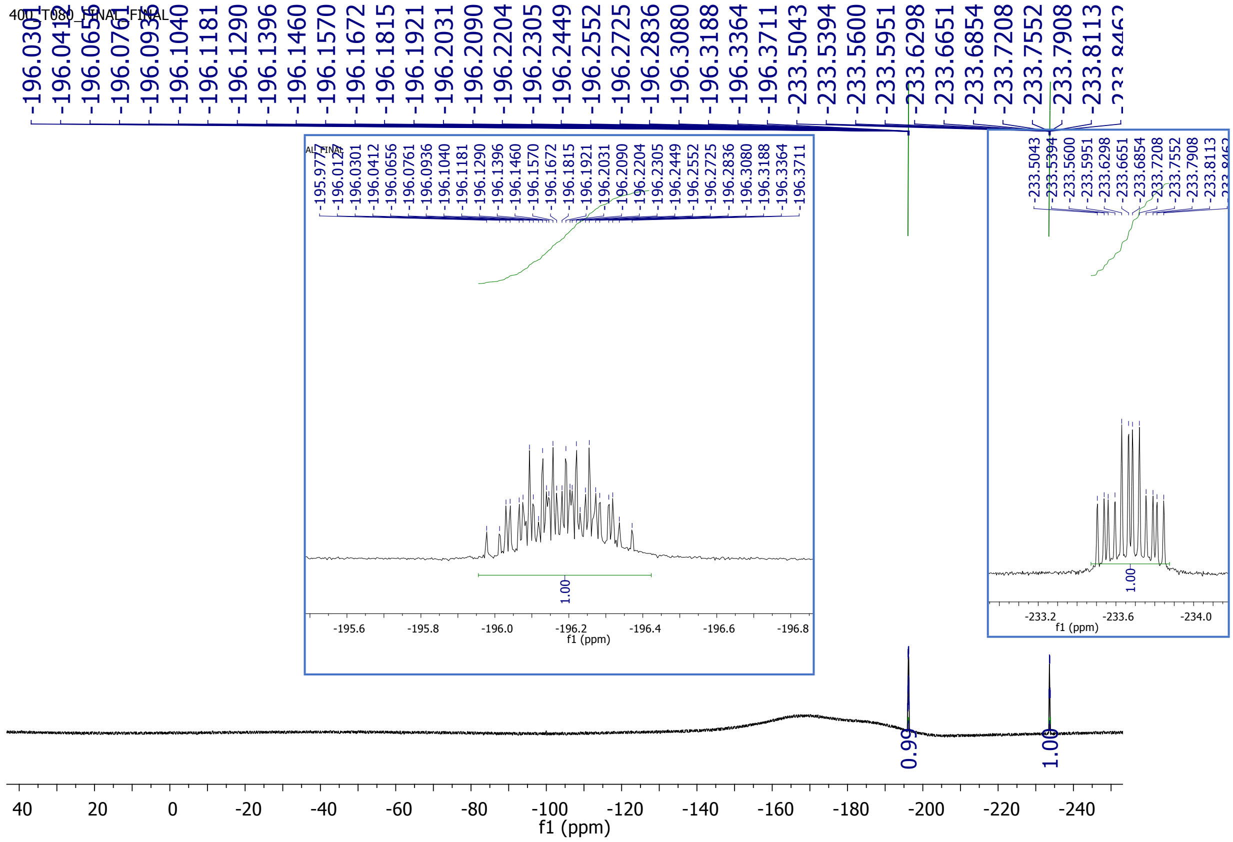

^{13}C NMR of 1-Bromo-2-((2,3-difluoropropoxy)methyl)benzene (6d)

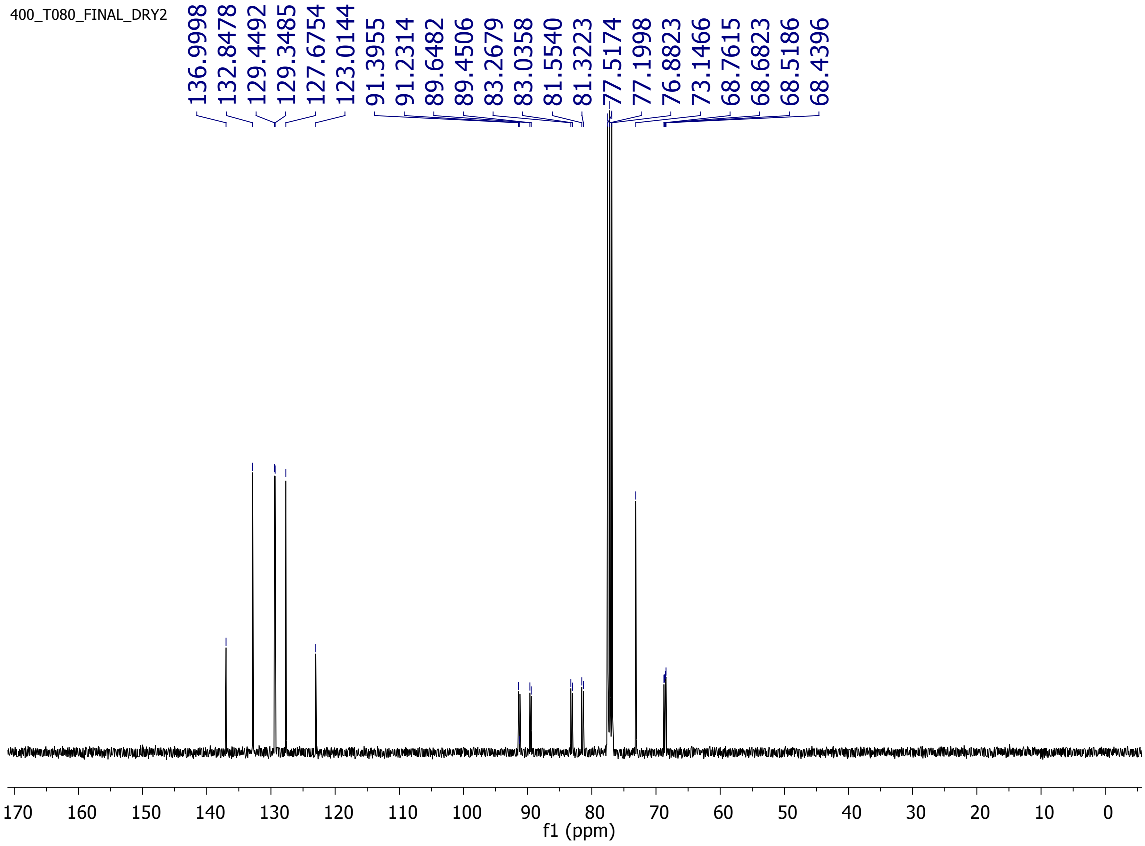

^{1}H NMR of 1-((2,3-Difluoropropoxy)methyl)-2-methylbenzene (6c)

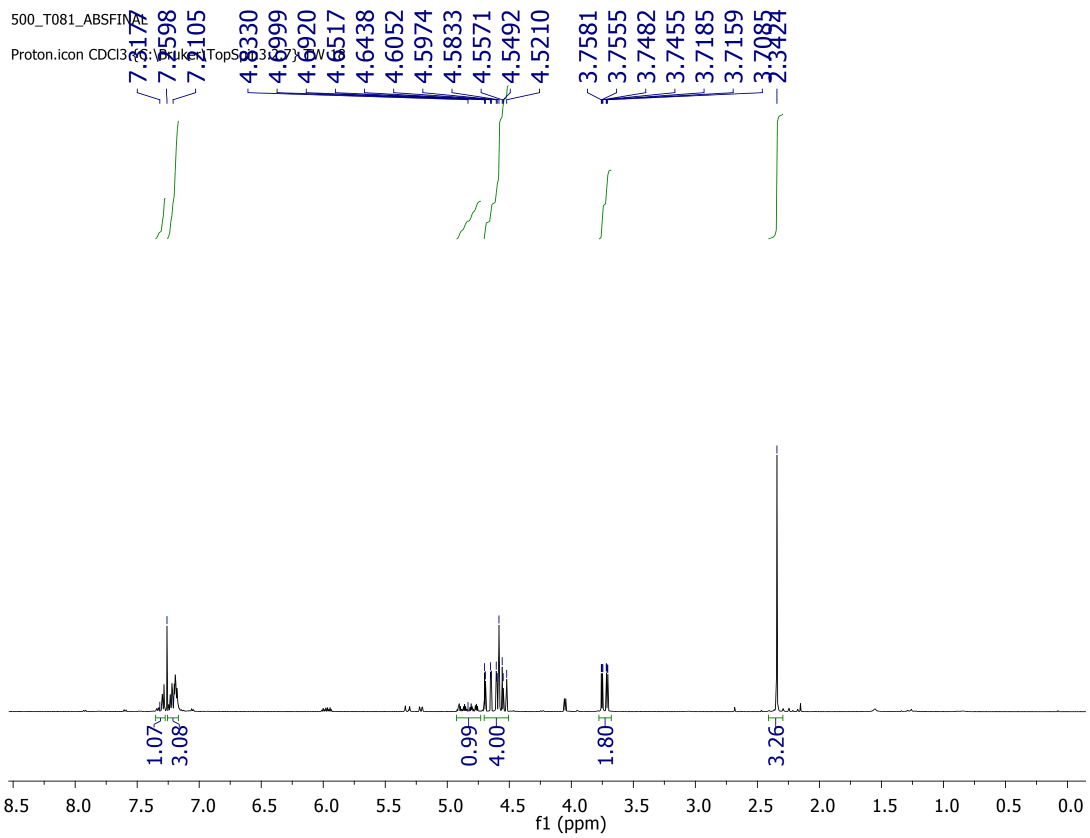

^{19}F NMR of 1-((2,3-Difluoropropoxy)methyl)-2-methylbenzene (6c)

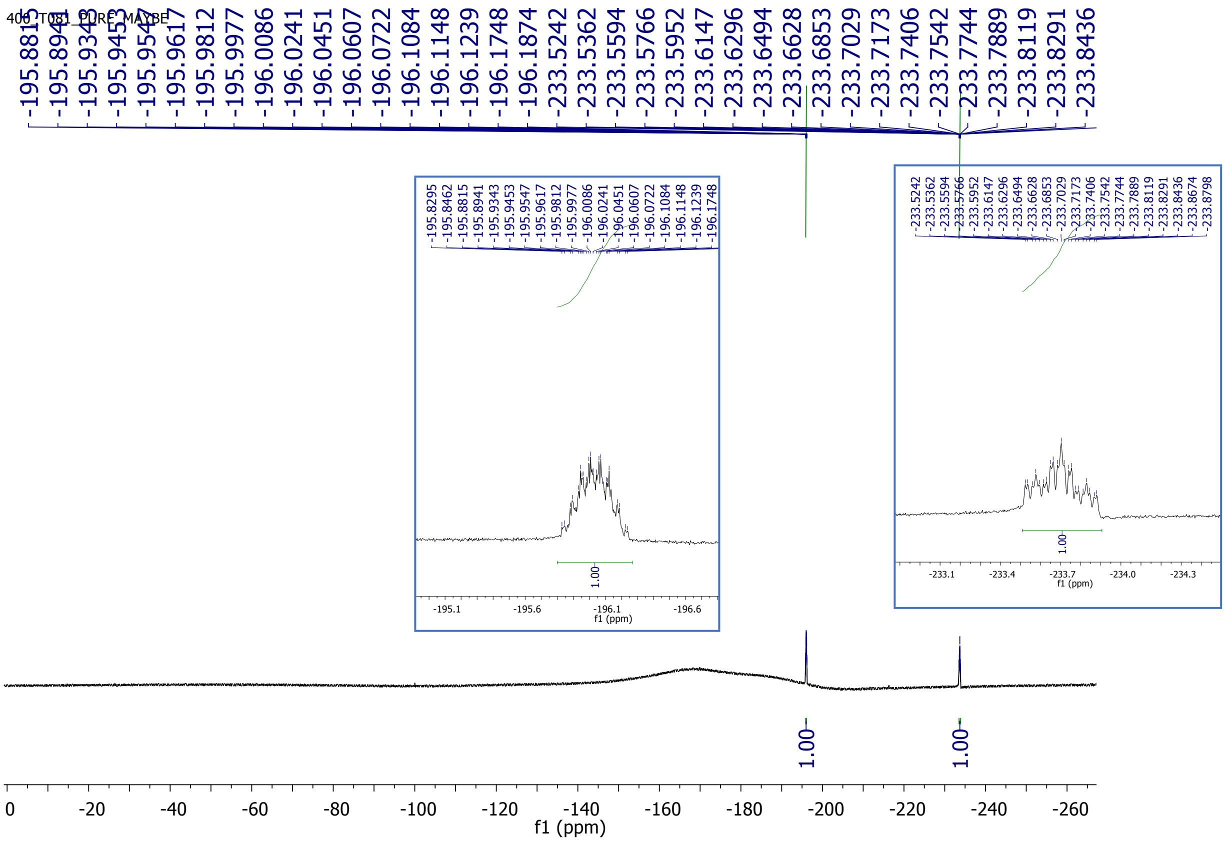

^{13}C NMR of 1-((2,3-Difluoropropoxy)methyl)-2-methylbenzene (6c)

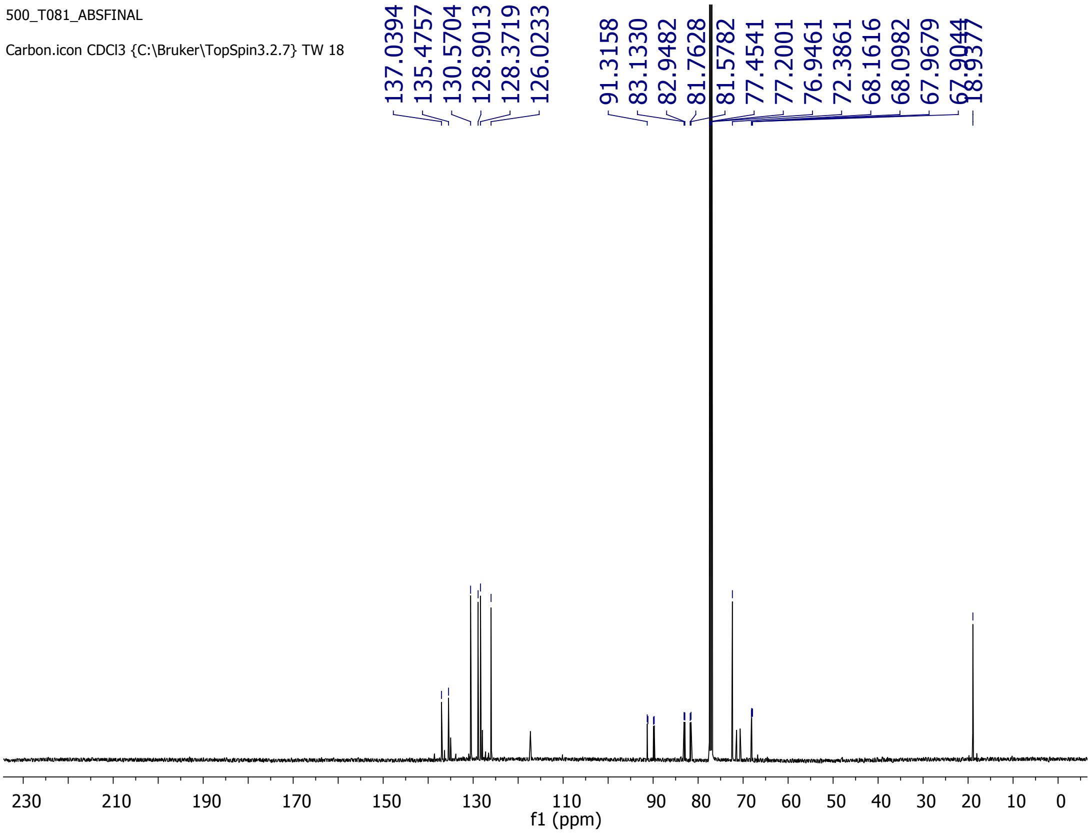

^{1}H NMR of 2-((2,3-difluoropropoxy)methyl)pyridine (6e)

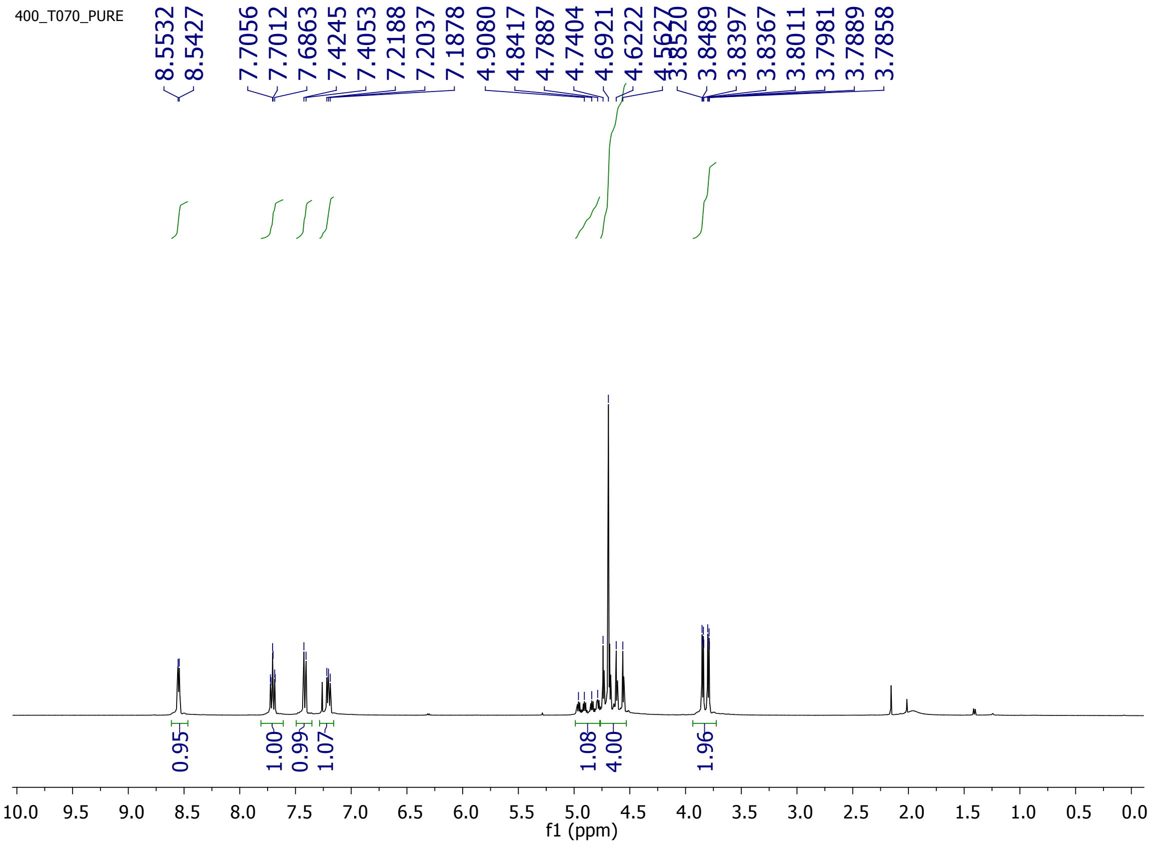

^{13}C NMR of 2-((2,3-difluoropropoxy)methyl)pyridine (6e)

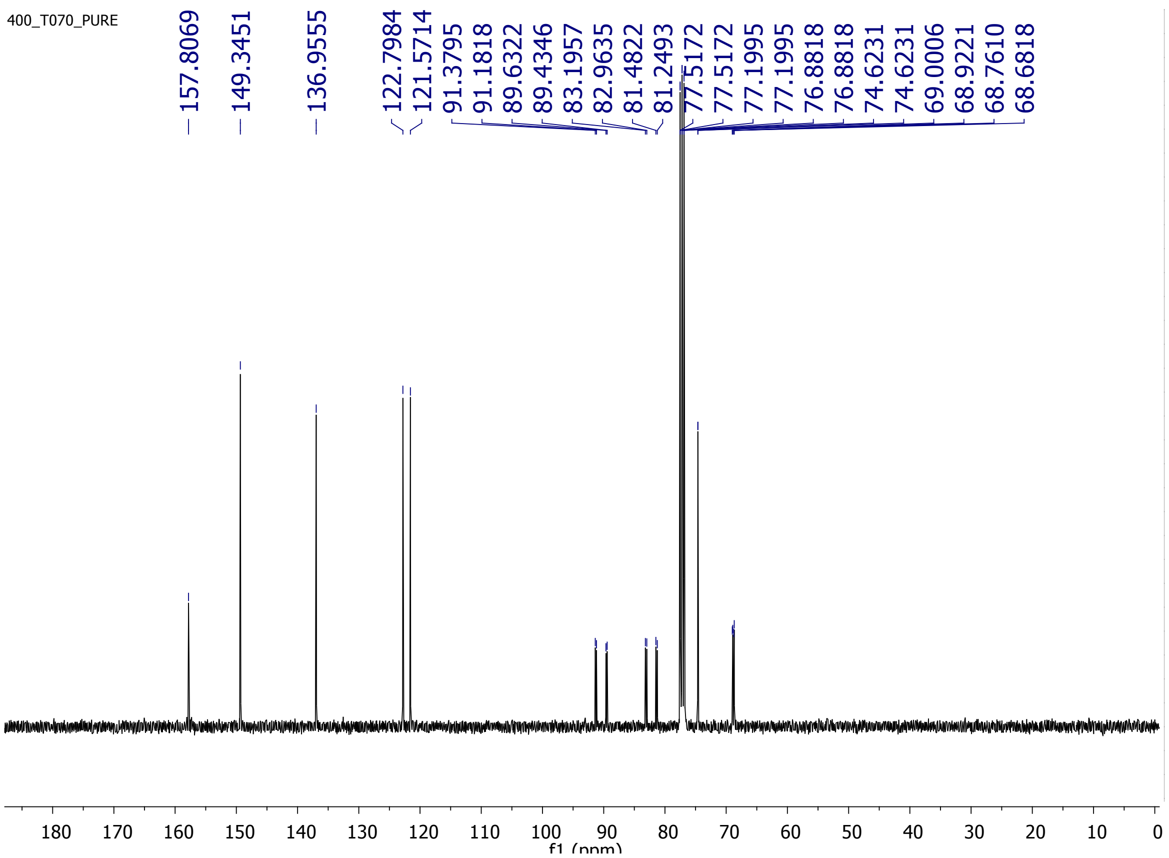

^{19}F NMR of 2-((2,3-difluoropropoxy)methyl)pyridine (6e)

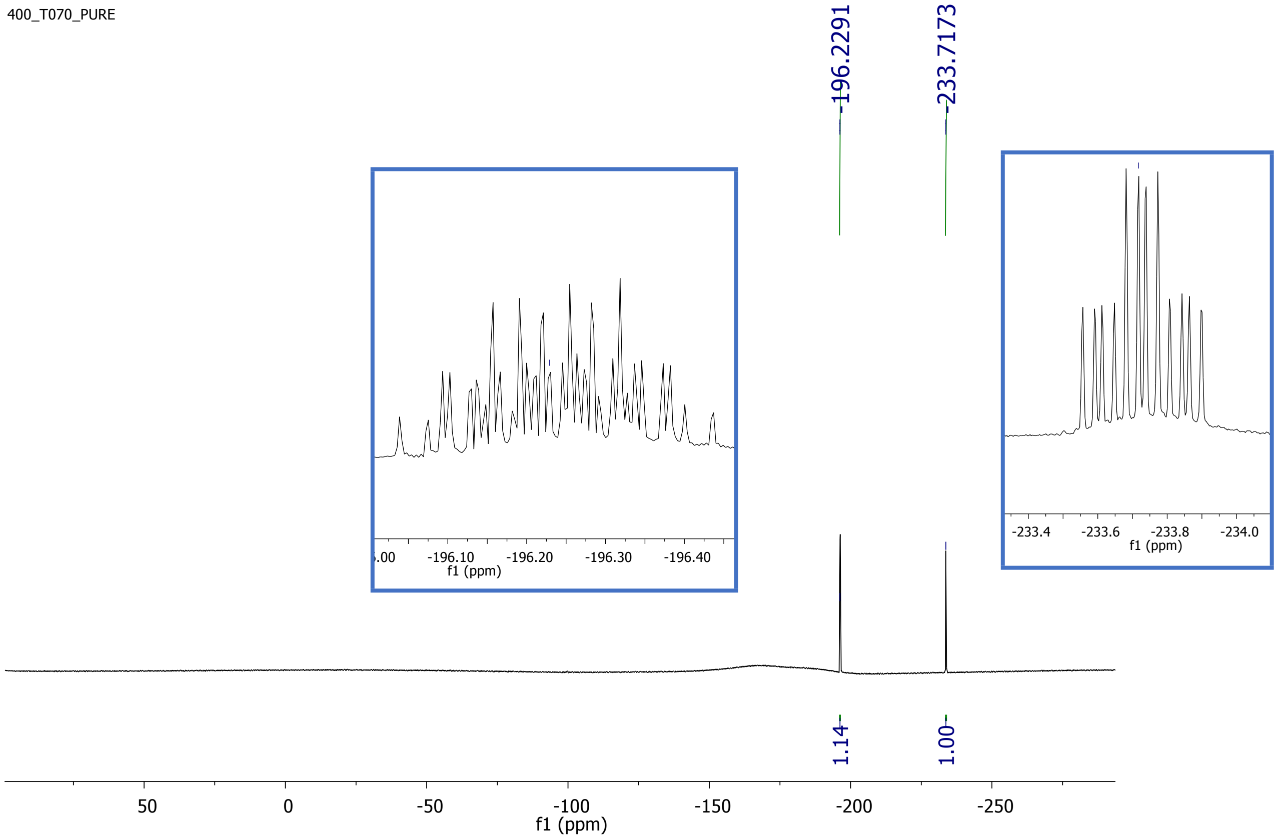

^{1}H NMR of ((2,3-Difluoro-2-methylpropoxy)methyl)benzene (6f)

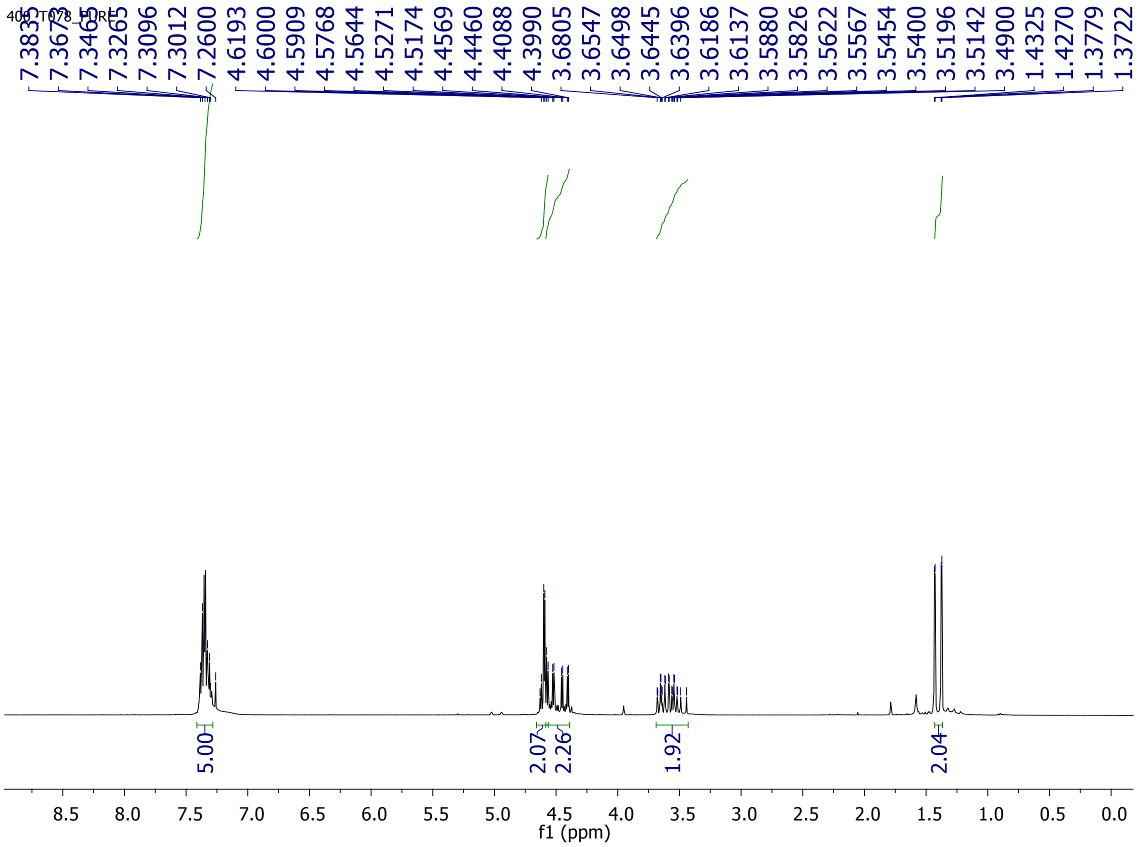

^{19}F NMR of ((2,3-Difluoro-2-methylpropoxy)methyl)benzene (6f)

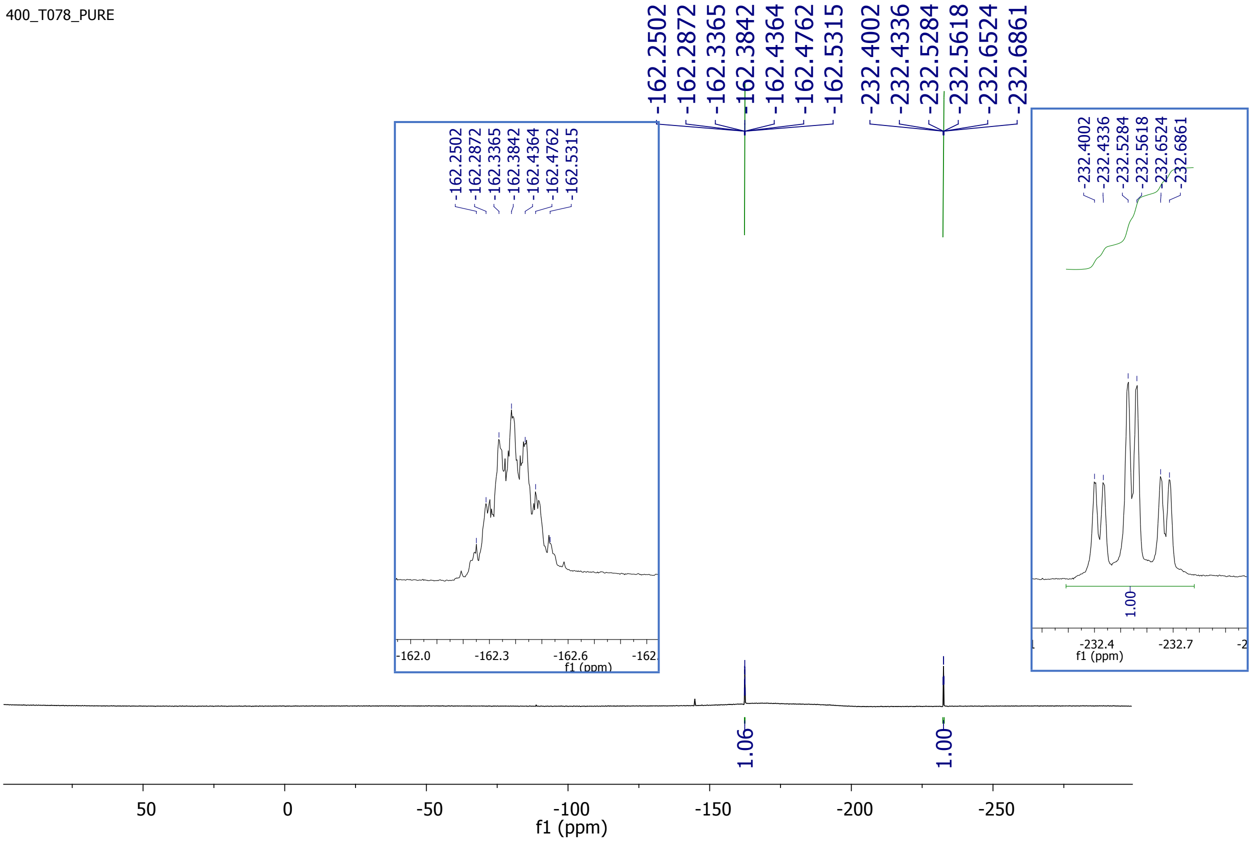

^{13}C NMR of ((2,3-Difluoro-2-methylpropoxy)methyl)benzene (6f)

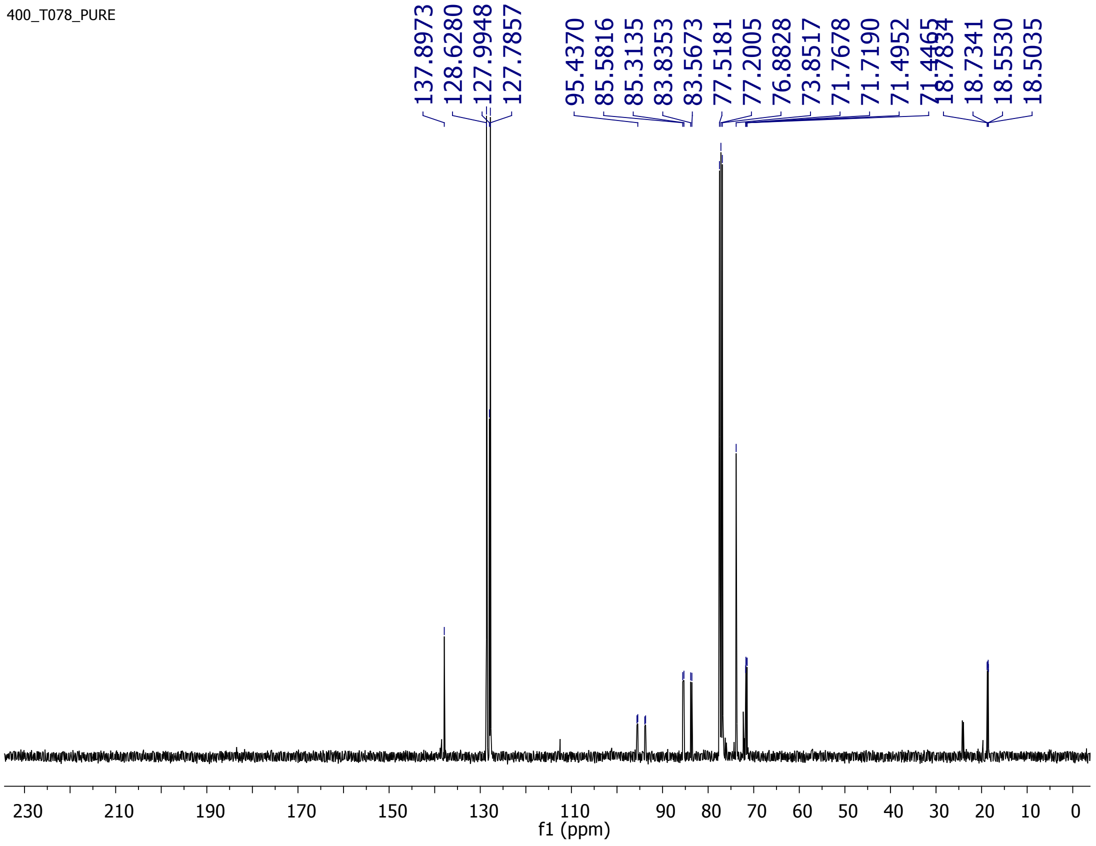

^{1}H NMR of 1-((2,3-Difluoro-2-methylpropoxy)methyl)-4-(trifluoromethyl)benzene (6g)

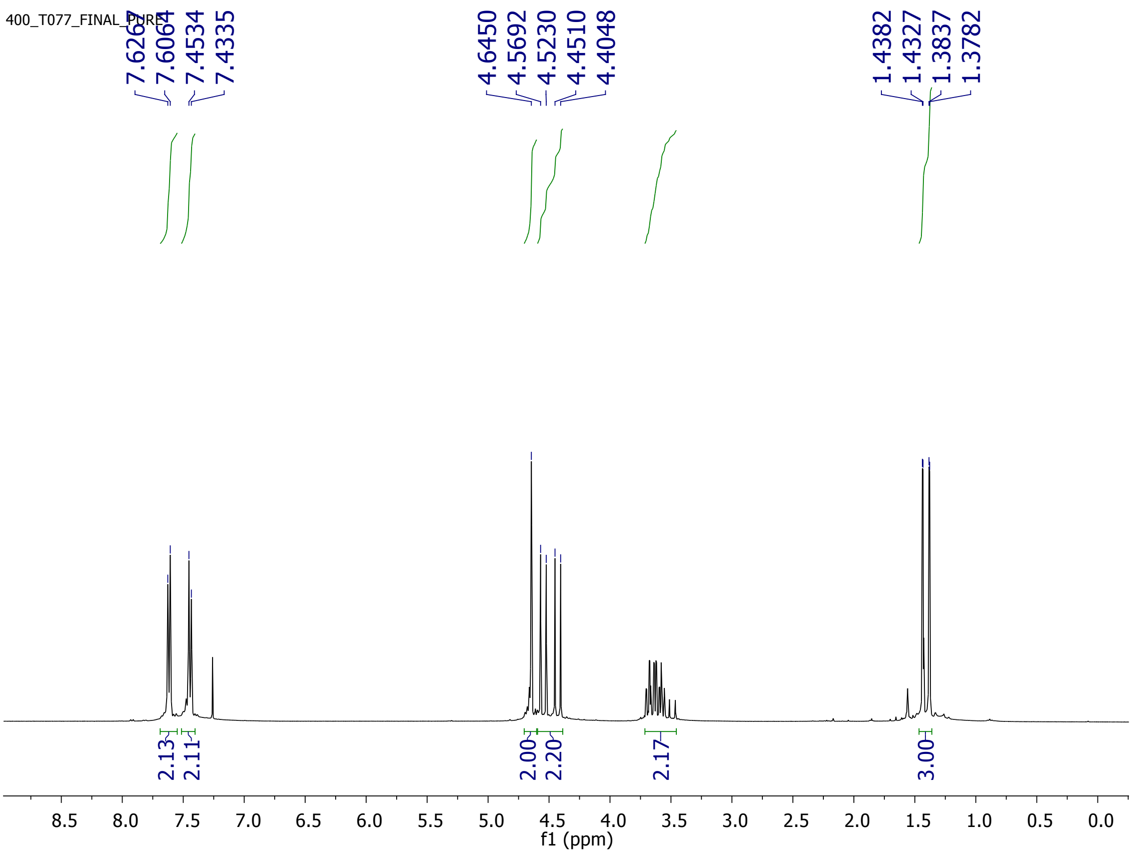

^{19}F NMR of 1-((2,3-Difluoro-2-methylpropoxy)methyl)-4-(trifluoromethyl)benzene (6g)

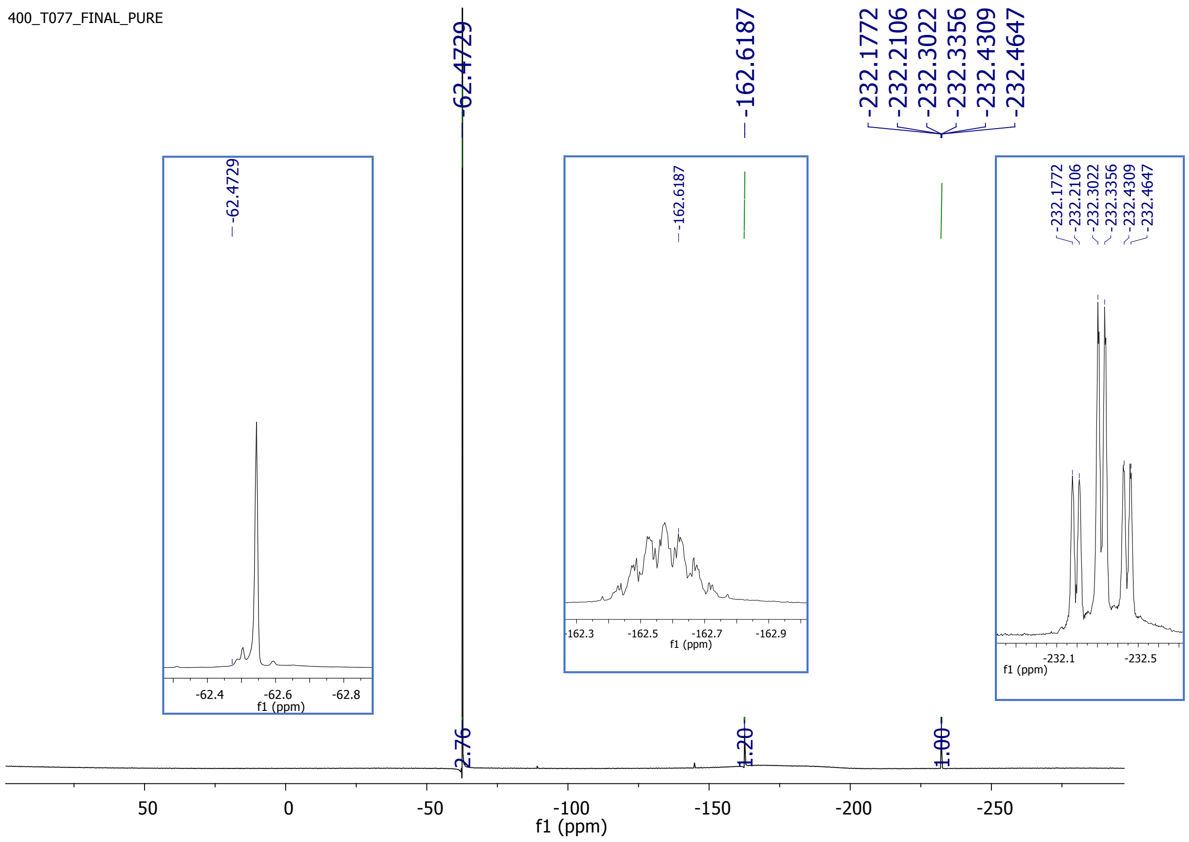

^{13}C NMR of 1-((2,3-Difluoro-2-methylpropoxy)methyl)-4-(trifluoromethyl)benzene (6g)

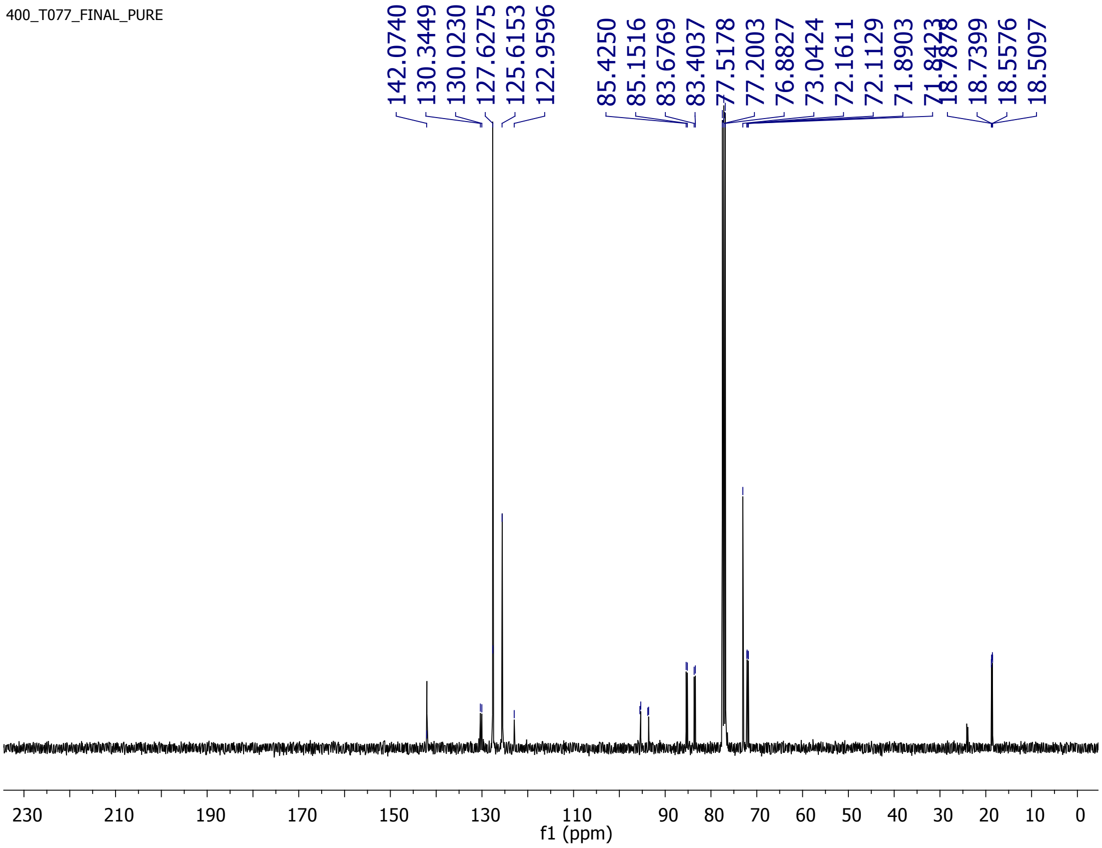

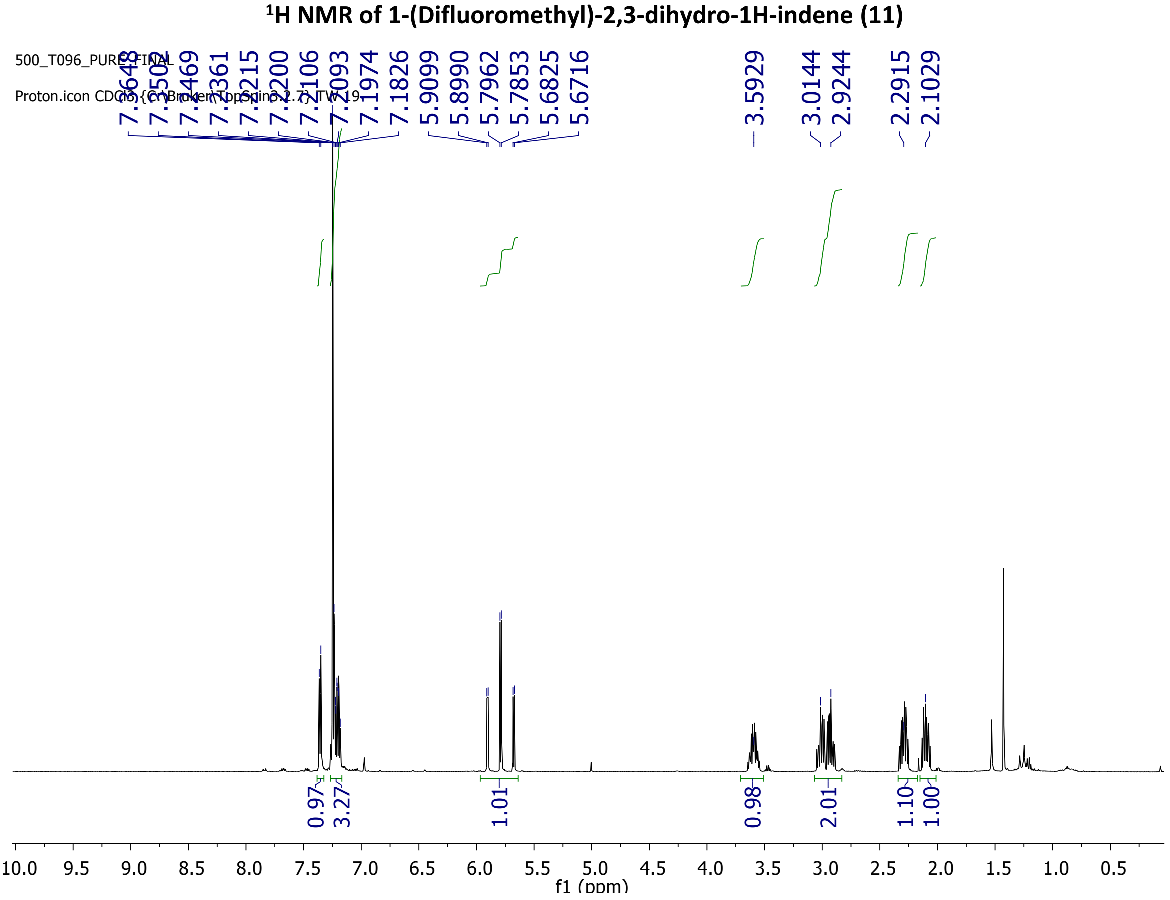

¹H NMR of 1-(Difluoromethyl)-2,3-dihydro-1H-indene (11)
500_T096_PURE_FINAL
Proton.icon CDCl3 {C:\Bruker\TopSpin3.2.7} TW 19
7.3502
7.3469
7.3361
7.2215
7.2200
7.2106
7.2093
7.1974
7.1826
5.9099
5.8990
5.7962
5.7853
5.6825
5.6716
3.5929
3.0144
2.9244
2.2915
2.1029
0.97
3.27
1.01
0.98
2.01
1.10
1.00
f1 (ppm)

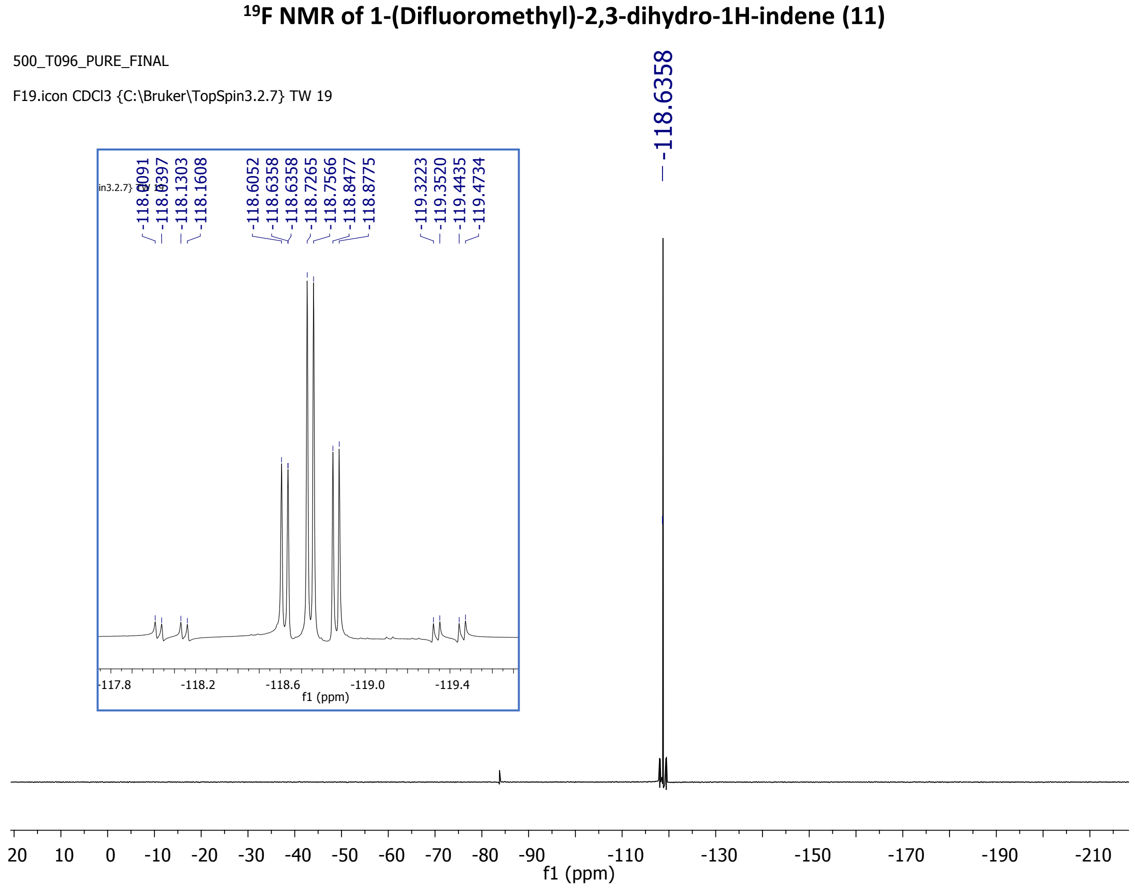

¹⁹F NMR of 1-(Difluoromethyl)-2,3-dihydro-1H-indene (11)
500_T096_PURE_FINAL
F19.icon CDCl3 {C:\Bruker\TopSpin3.2.7} TW 19
-118.6358
-118.0397
-118.1303
-118.1608
-118.6052
-118.6358
-118.6358
-118.7265
-118.7566
-118.8477
-118.8775
-119.3223
-119.3520
-119.4435
-119.4734
f1 (ppm)

^{13}C NMR of 1-(Difluoromethyl)-2,3-dihydro-1H-indene (11)

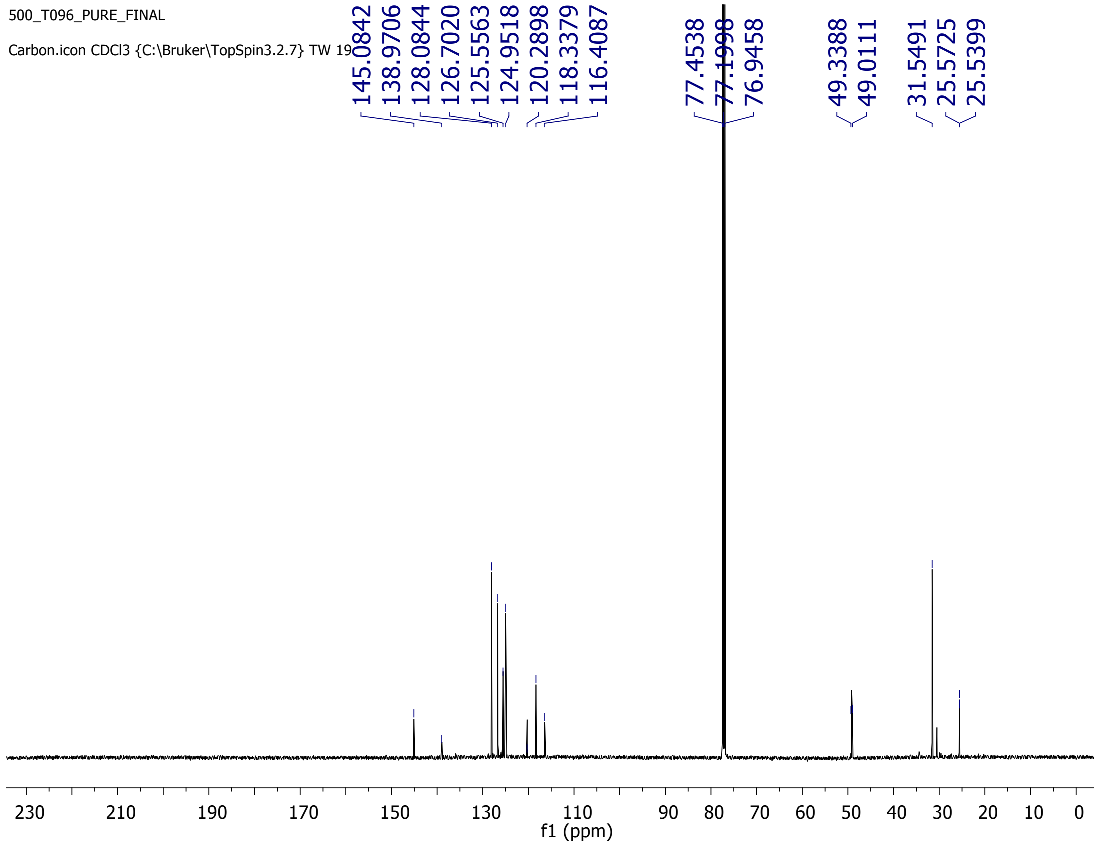

^{1}H NMR of 1,1-Difluoro-1,2-diphenylethane (5a)

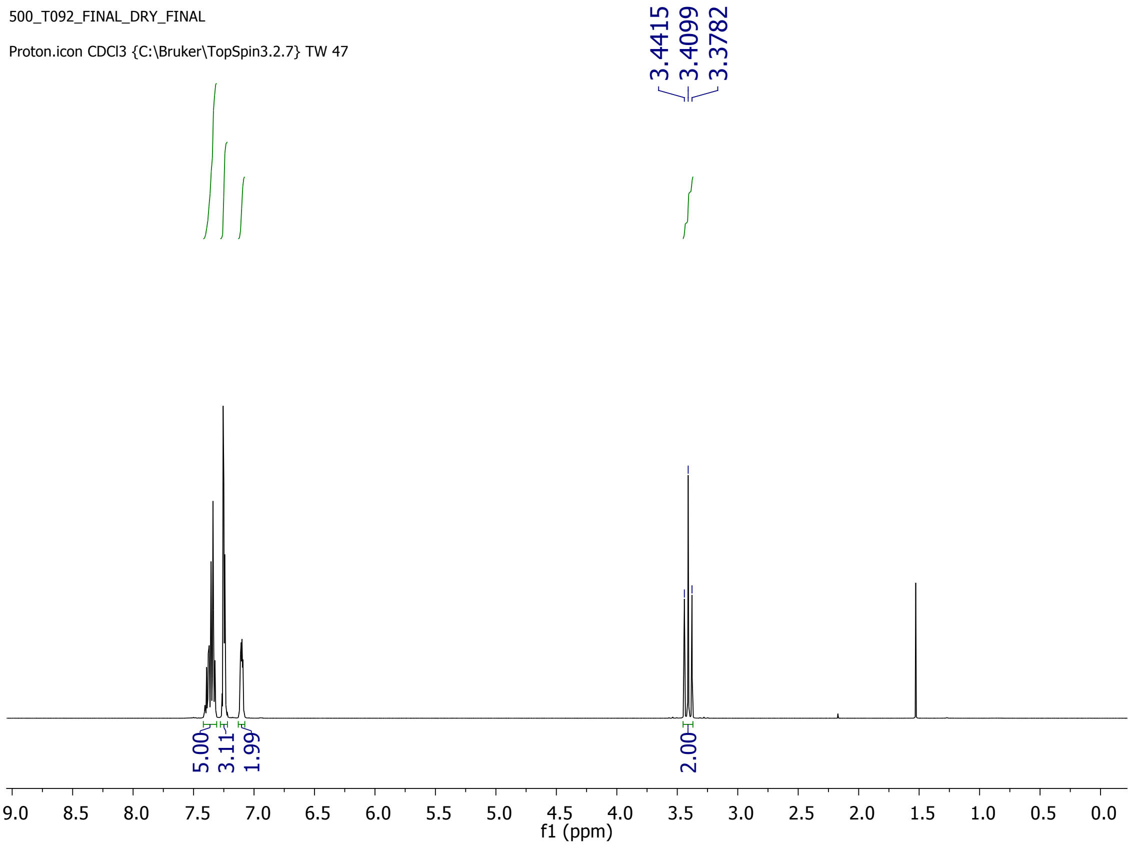

^{19}F NMR of 1,1-Difluoro-1,2-diphenylethane (5a)

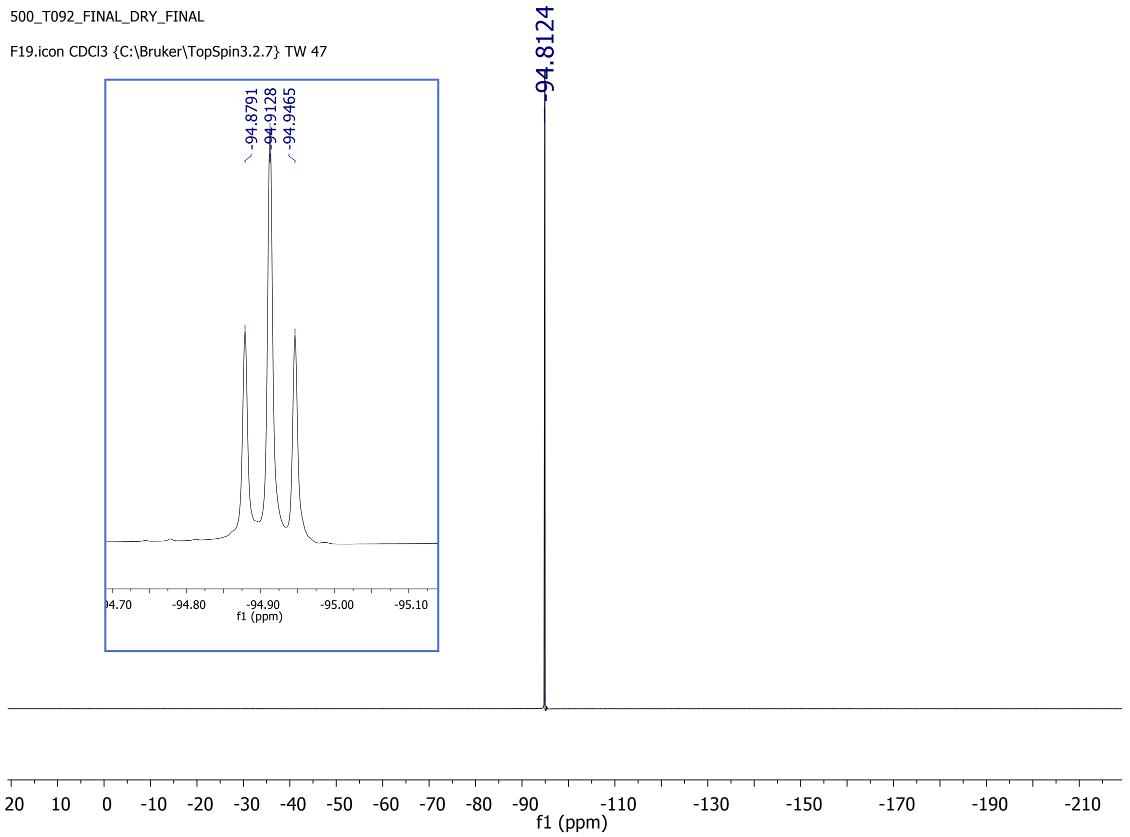

^{13}C NMR of 1,1-Difluoro-1,2-diphenylethane (5a)

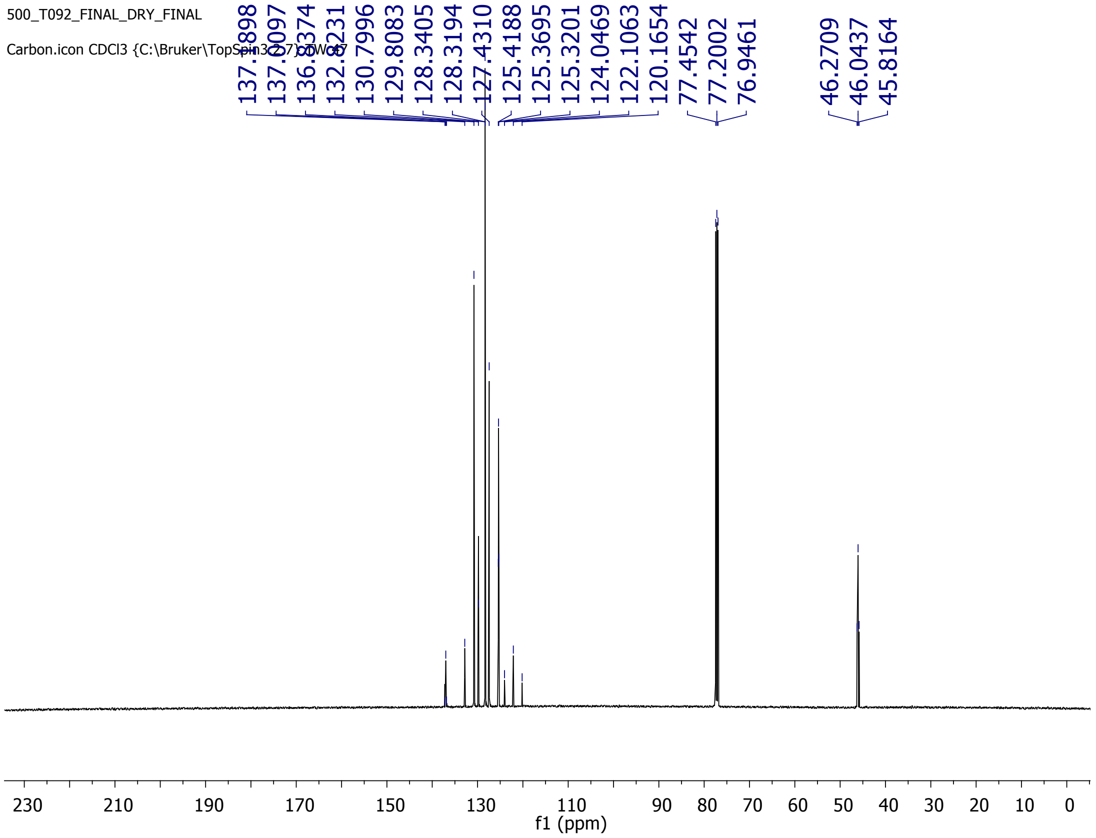

^{1}H NMR of 3-fluoro-3-(4-nitrophenyl)tetrahydrofuran (9a)

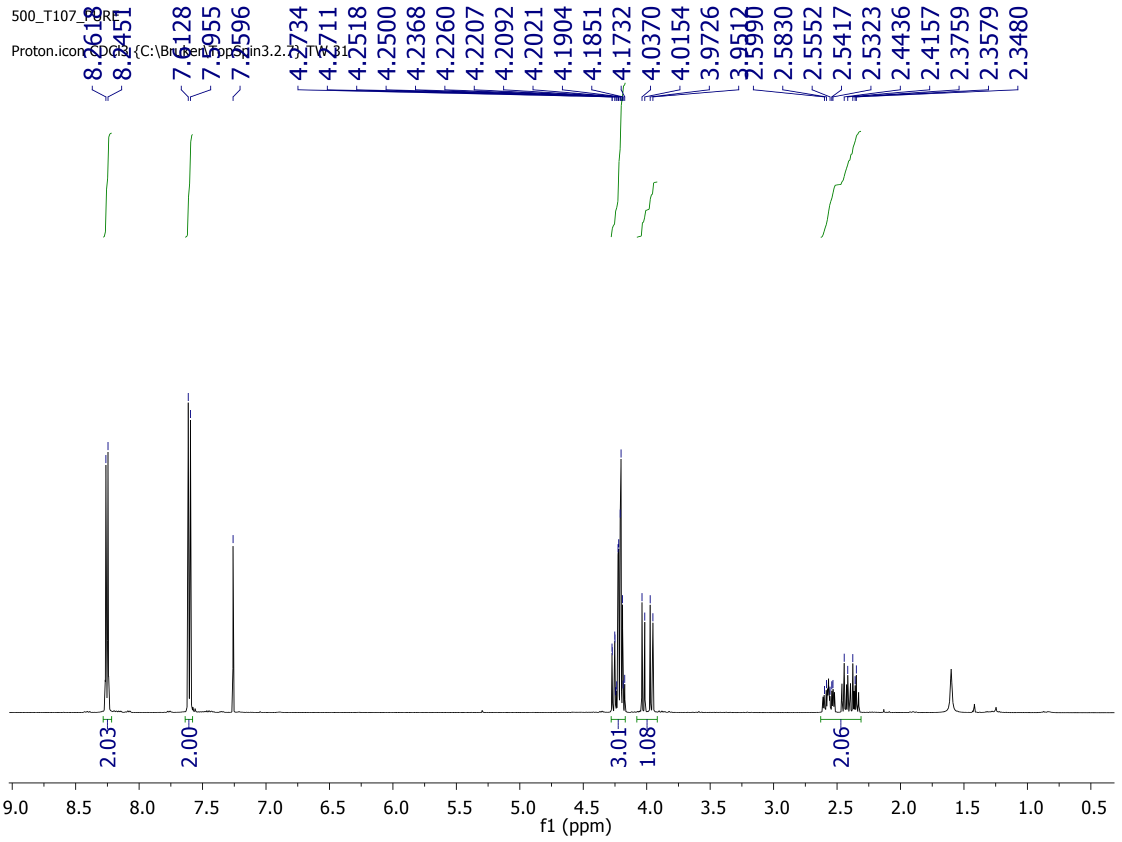

^{19}F NMR of 3-fluoro-3-(4-nitrophenyl)tetrahydrofuran (9a)

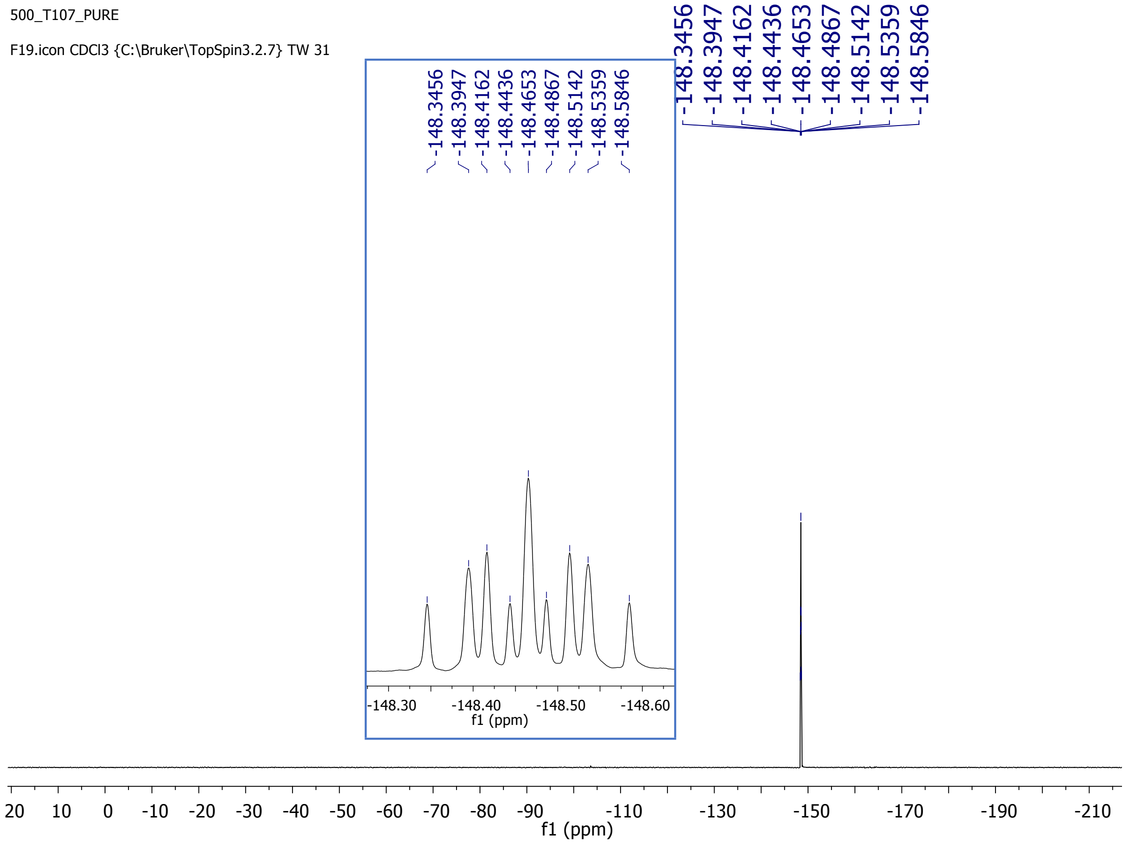

^{13}C NMR of 3-fluoro-3-(4-nitrophenyl)tetrahydrofuran (9a)

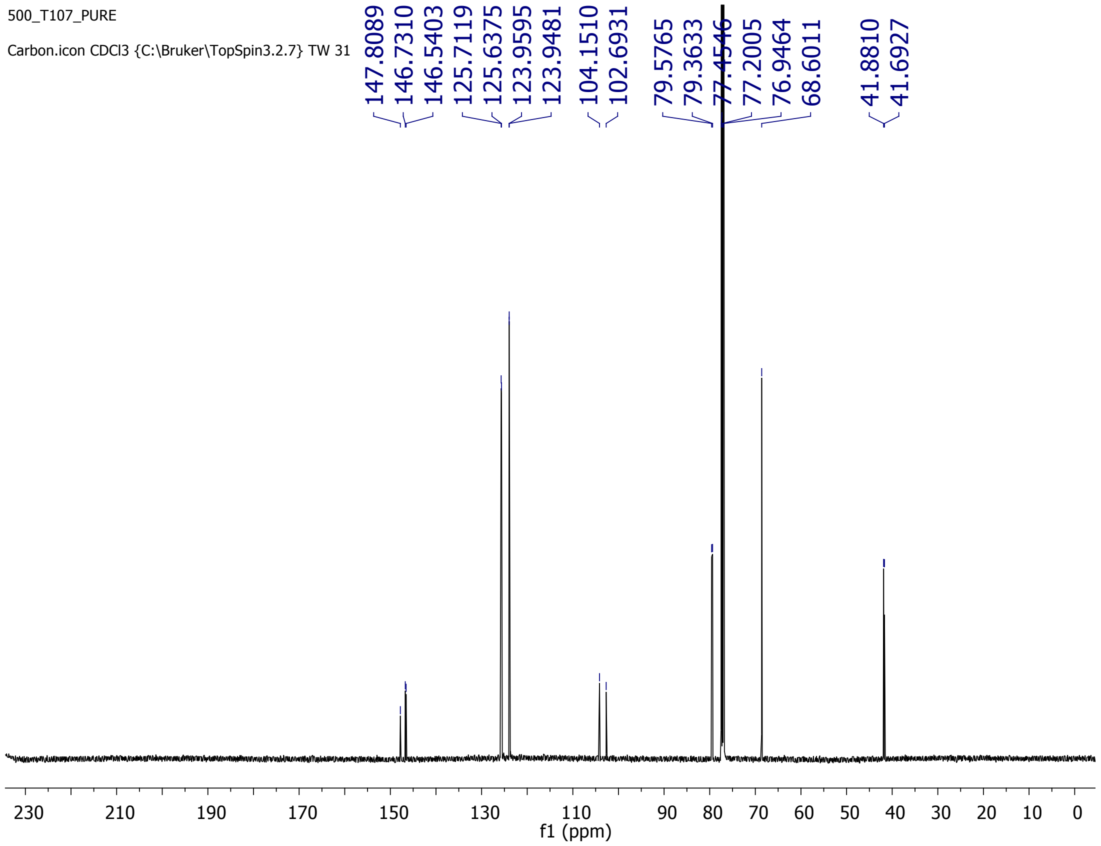

HRMS of 3-fluoro-3-(4-nitrophenyl)tetrahydrofuran (9a)

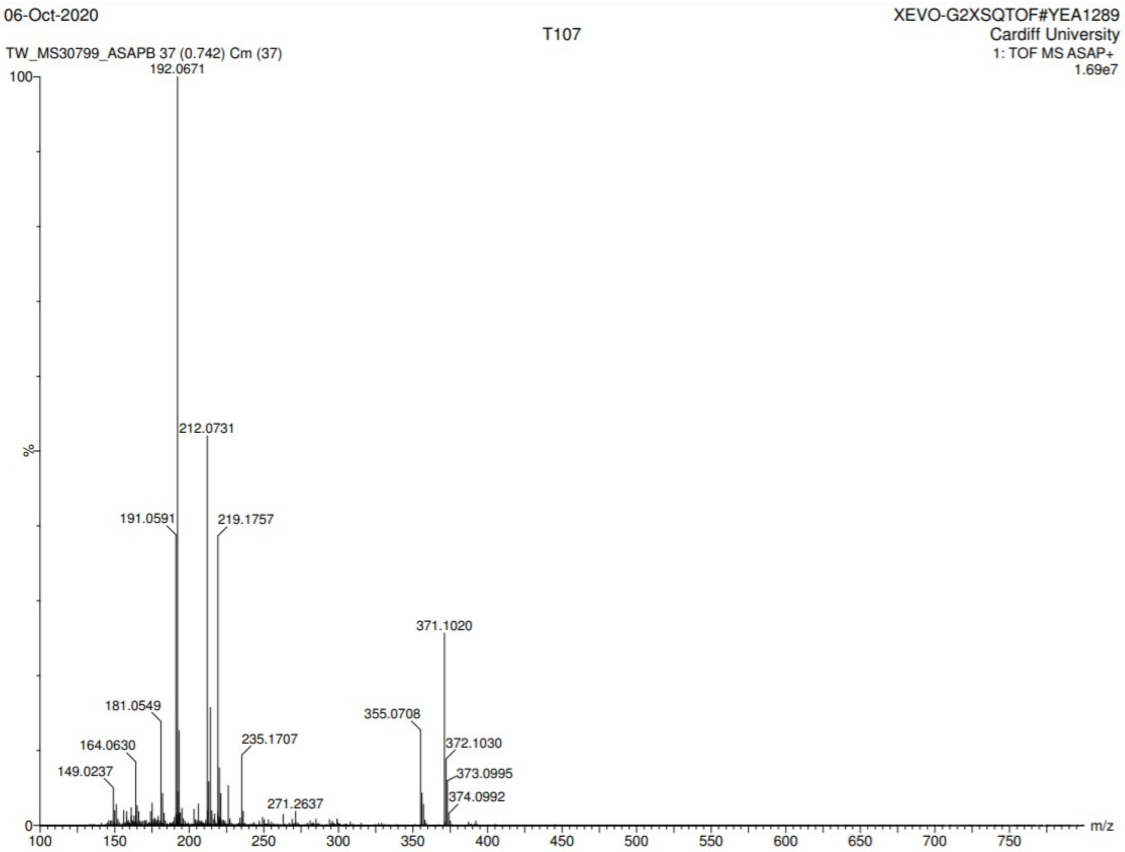

IR of 3-fluoro-3-(4-nitrophenyl)tetrahydrofuran (9a)

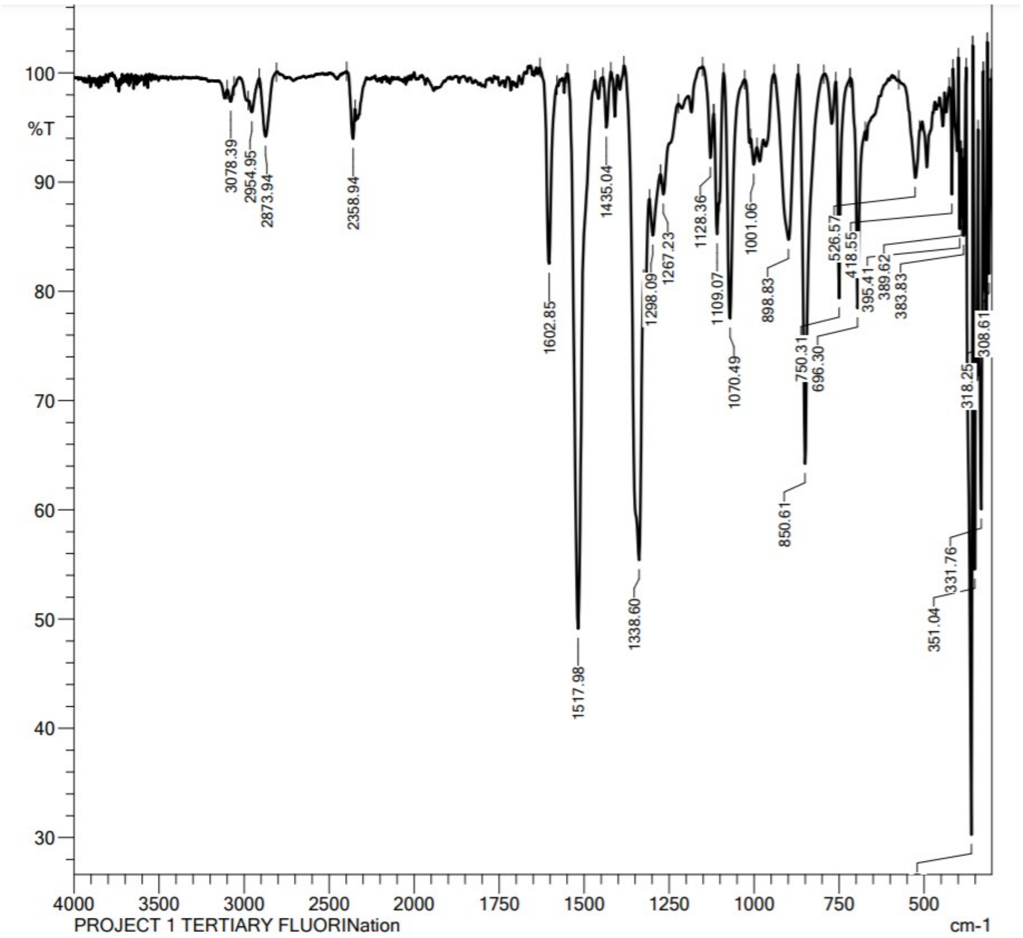

^{1}H NMR of 5-(Fluoromethyl)-3-phenyldihydrofuran-2(3H)-one (10a)

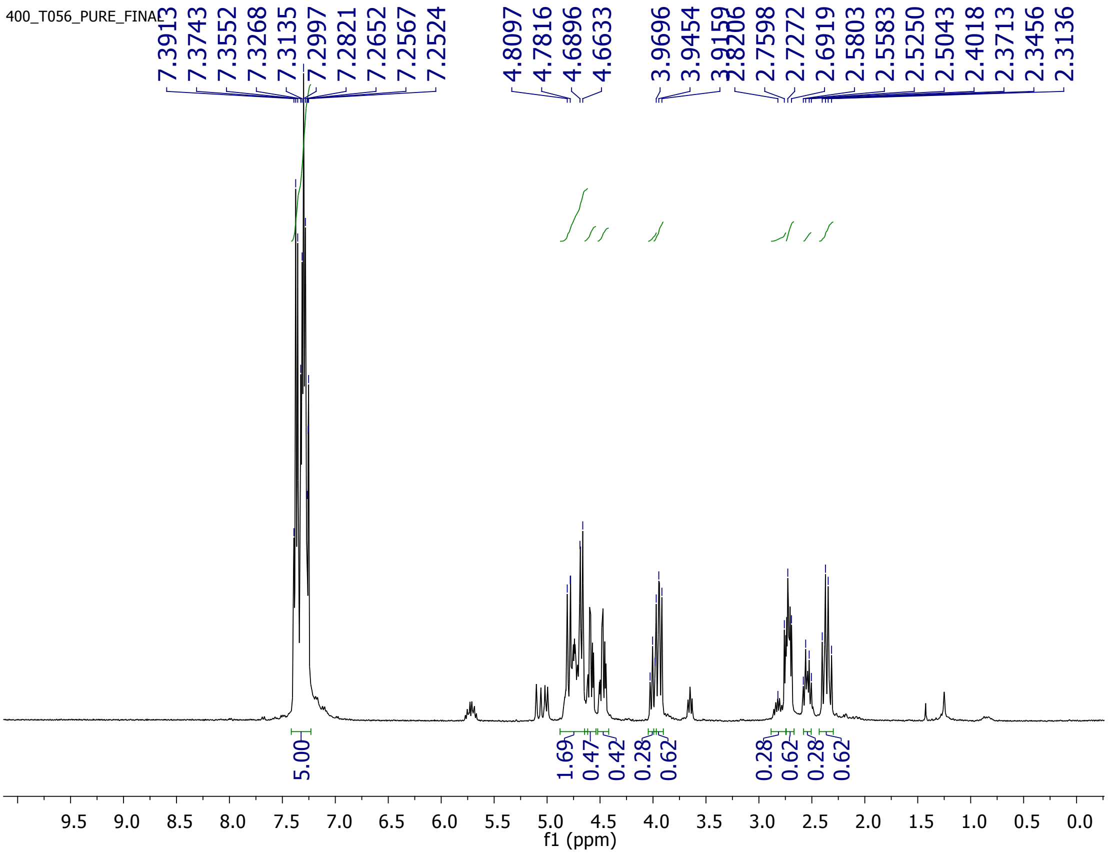

^{19}F NMR of 5-(Fluoromethyl)-3-phenyldihydrofuran-2(3H)-one (10a)

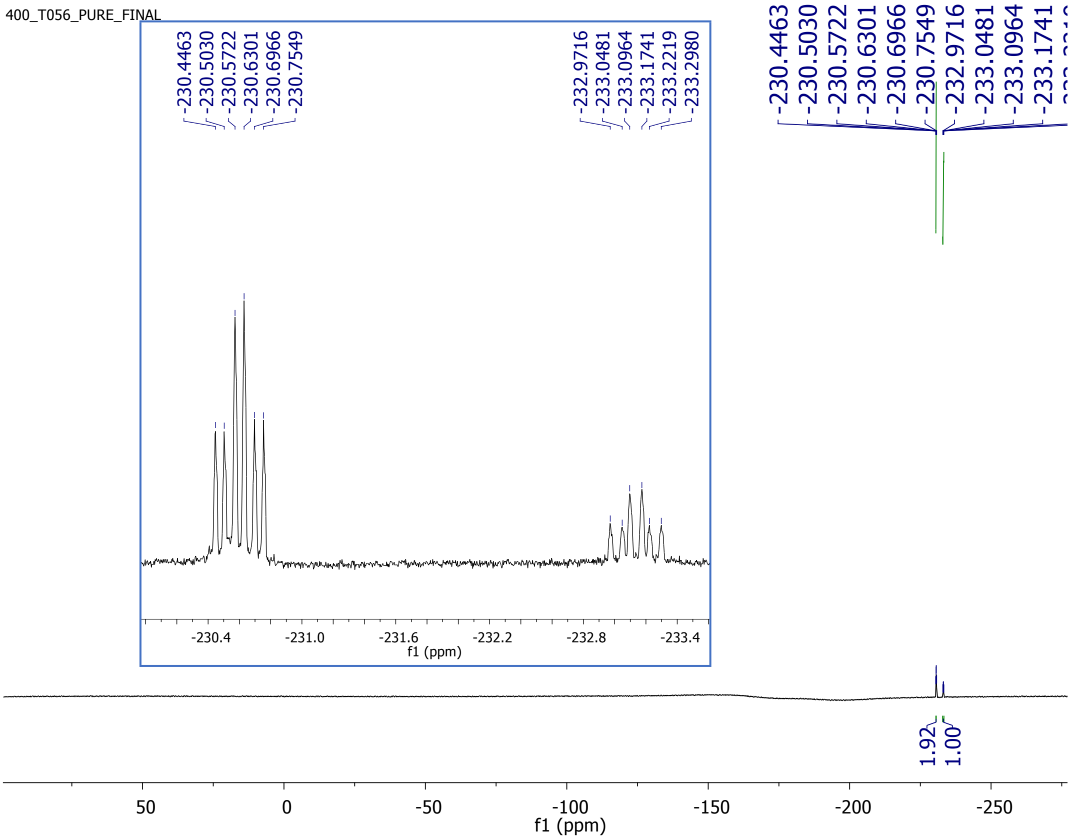

HRMS of 5-(Fluoromethyl)-3-phenyldihydrofuran-2(3H)-one (10a)

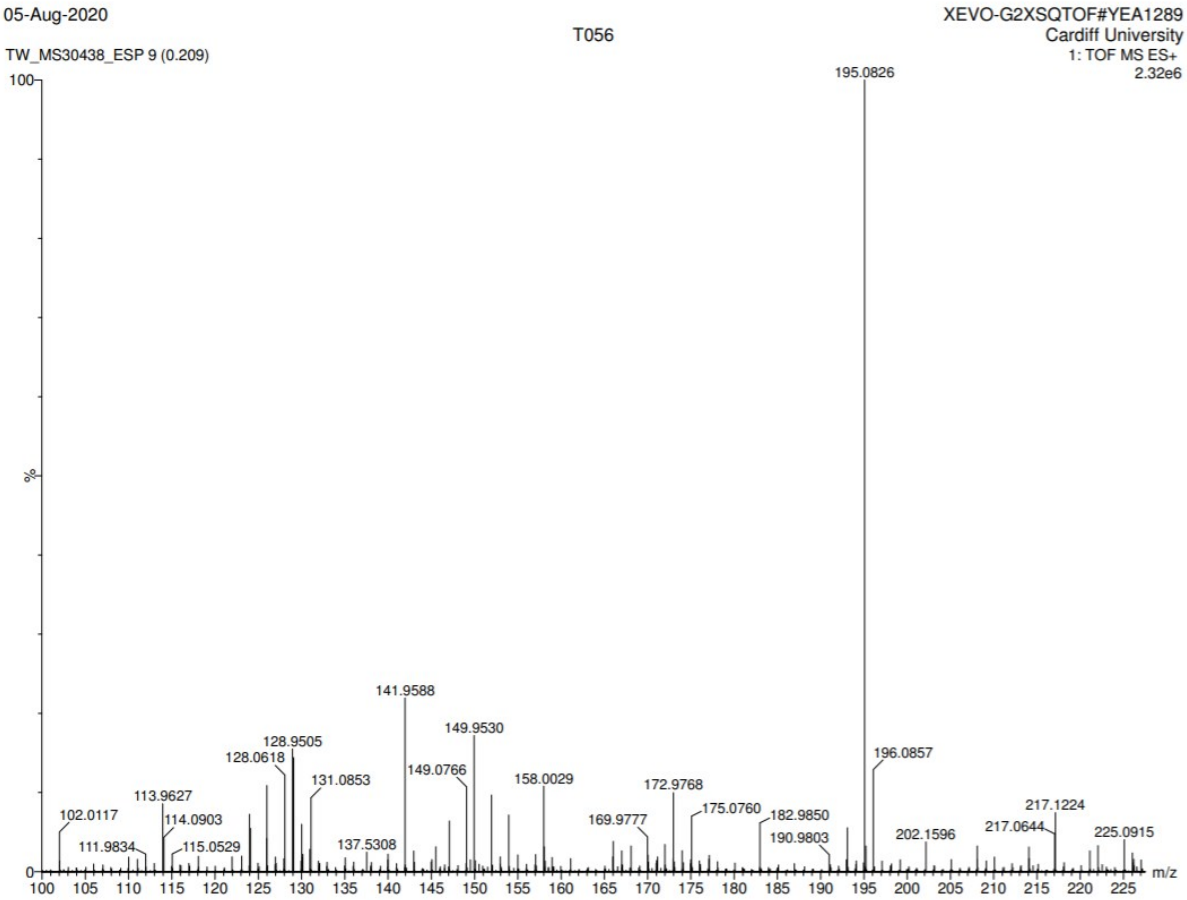

^{1}H NMR of (E)-(1-Fluoro-2-iodoethene-1,2-diyl)dibenzene (12a)

500_T135_PURE_PR1

Proton.icon CDCl3 {C:\Bruker\TopSpin3.2.7} TW 39

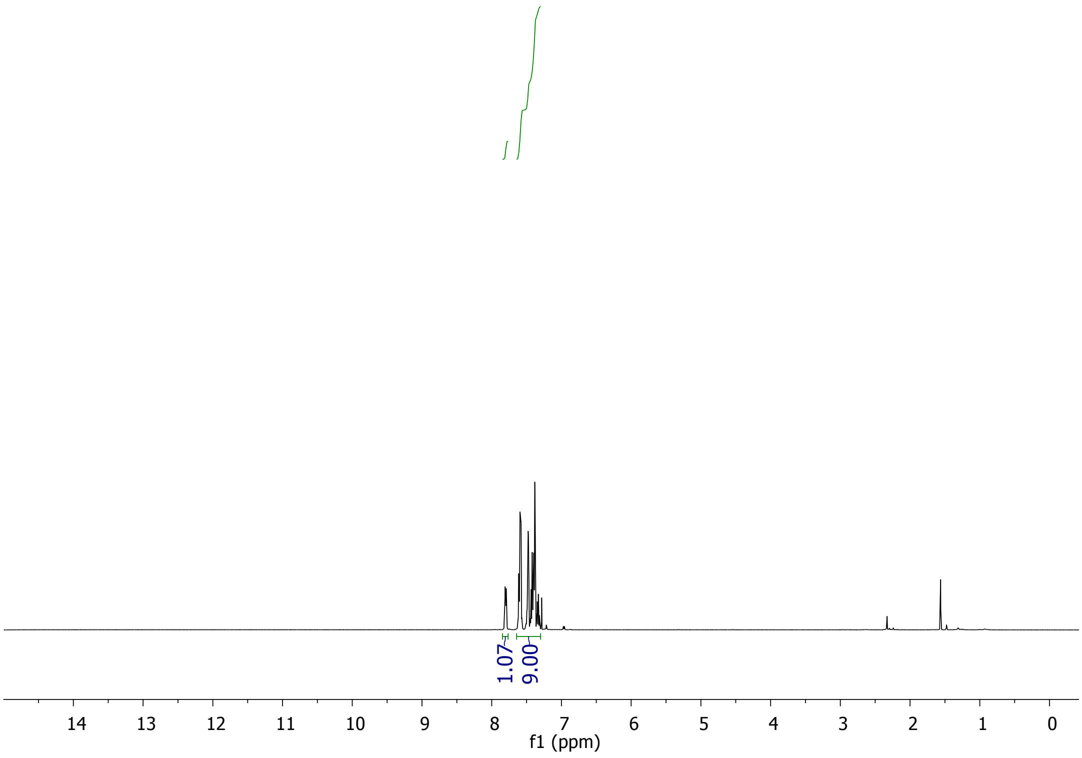

^{19}F NMR of (E)-(1-Fluoro-2-iodoethene-1,2-diyl)dibenzene (12a)

500_T135_PURE_PR1

F19.icon CDCl3 {C:\Bruker\TopSpin3.2.7} TW 39

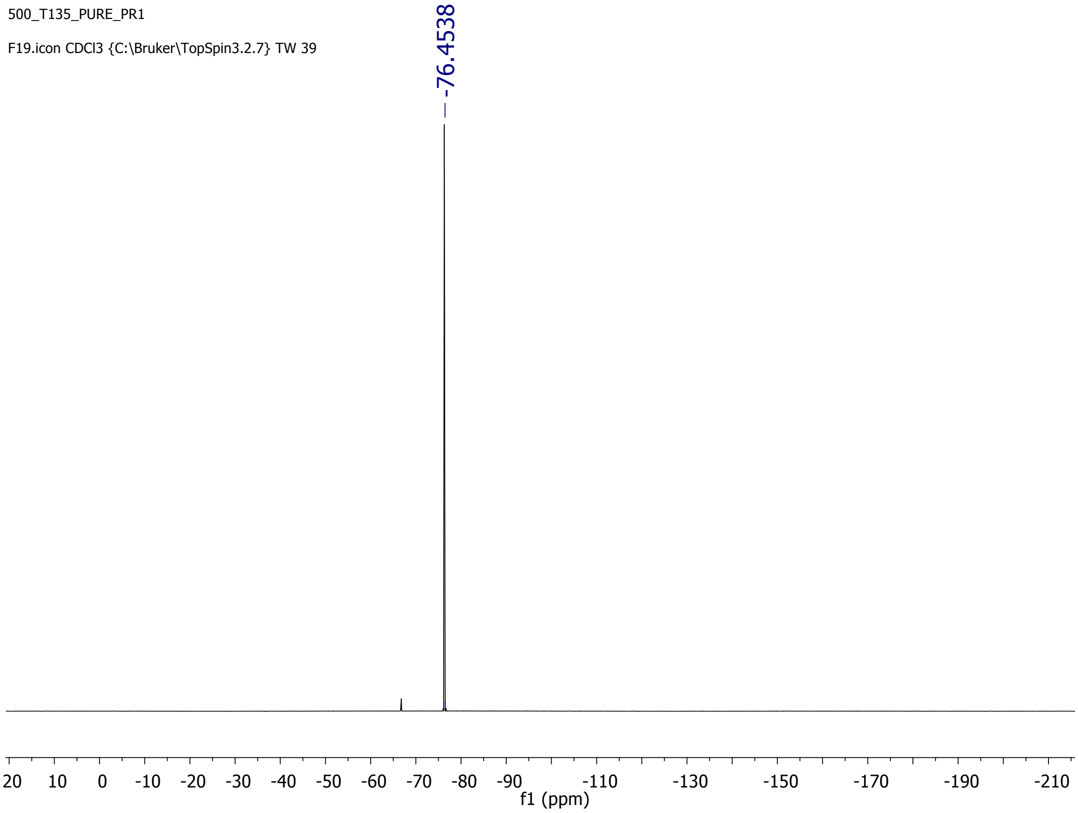

^{13}C NMR of (E)-(1-Fluoro-2-iodoethene-1,2-diyl)dibenzene (12a)

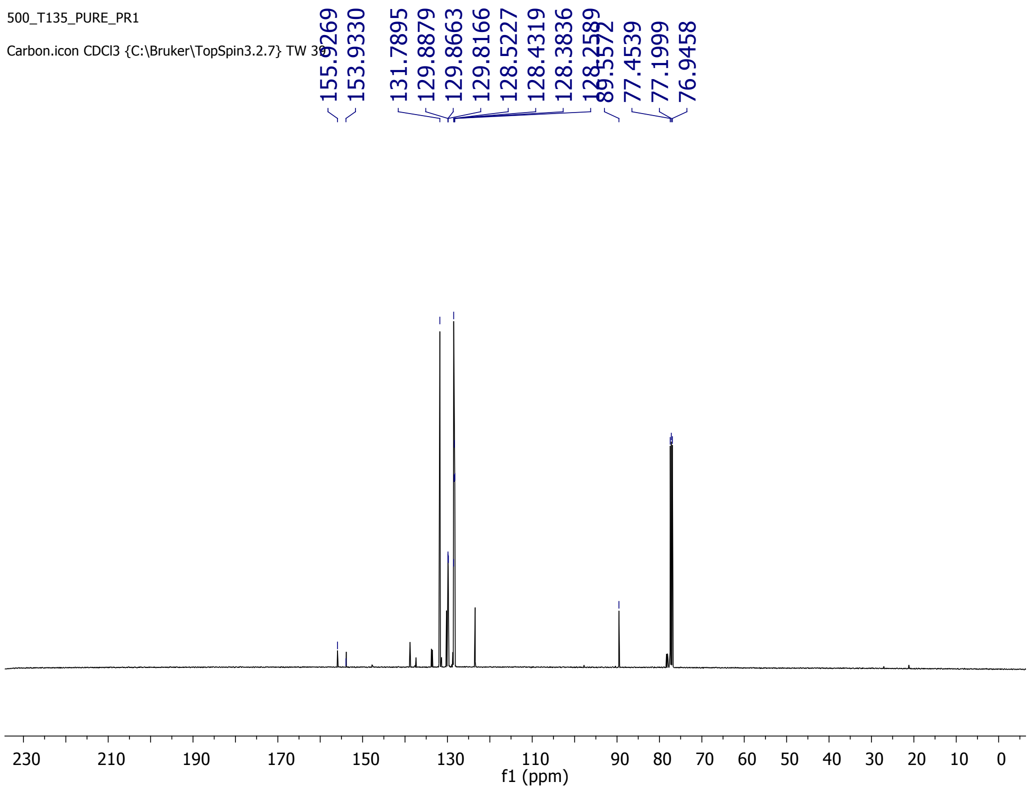

HRMS of (E)-(1-Fluoro-2-iodoethene-1,2-diyl)dibenzene (12a)

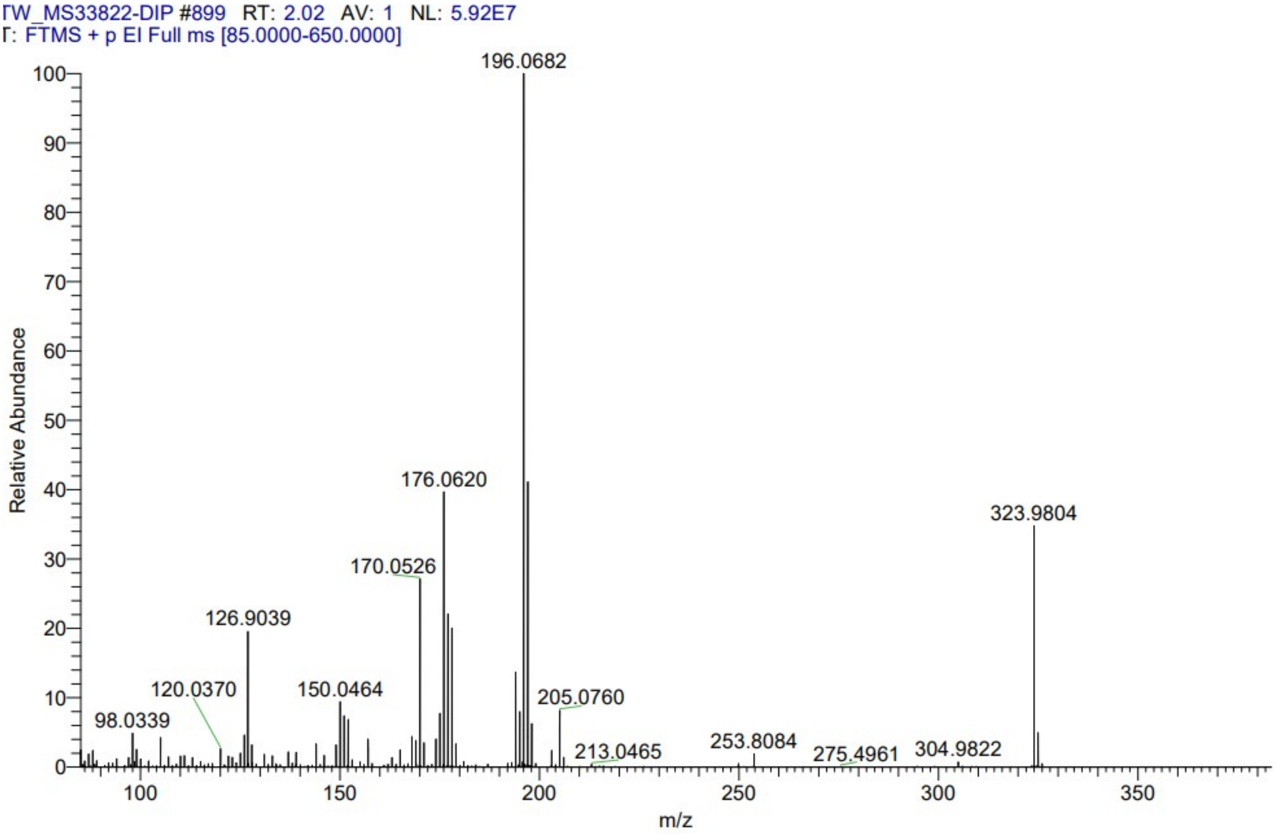

^{19}F NMR of (E)-(1-Fluoro-2-iodoprop-1-en-1-yl)benzene (12b)

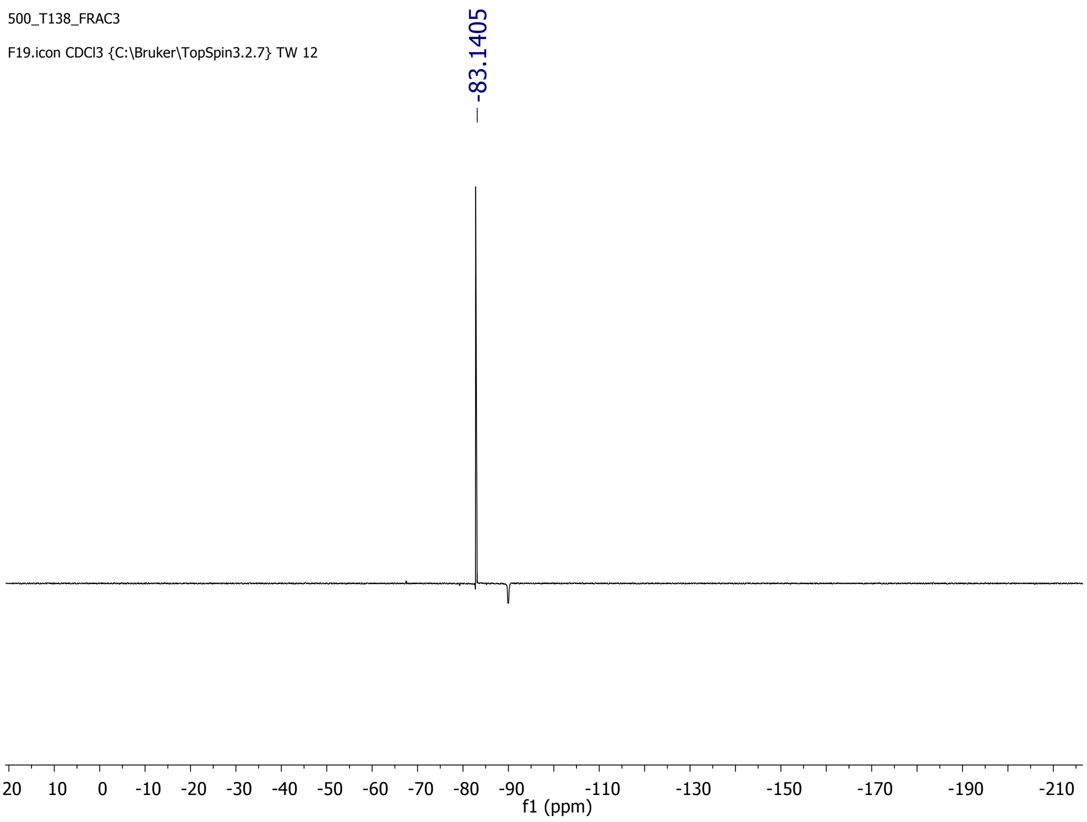

^{1}H NMR of (2-Fluoro-3-iodopropyl)benzene (12ca)

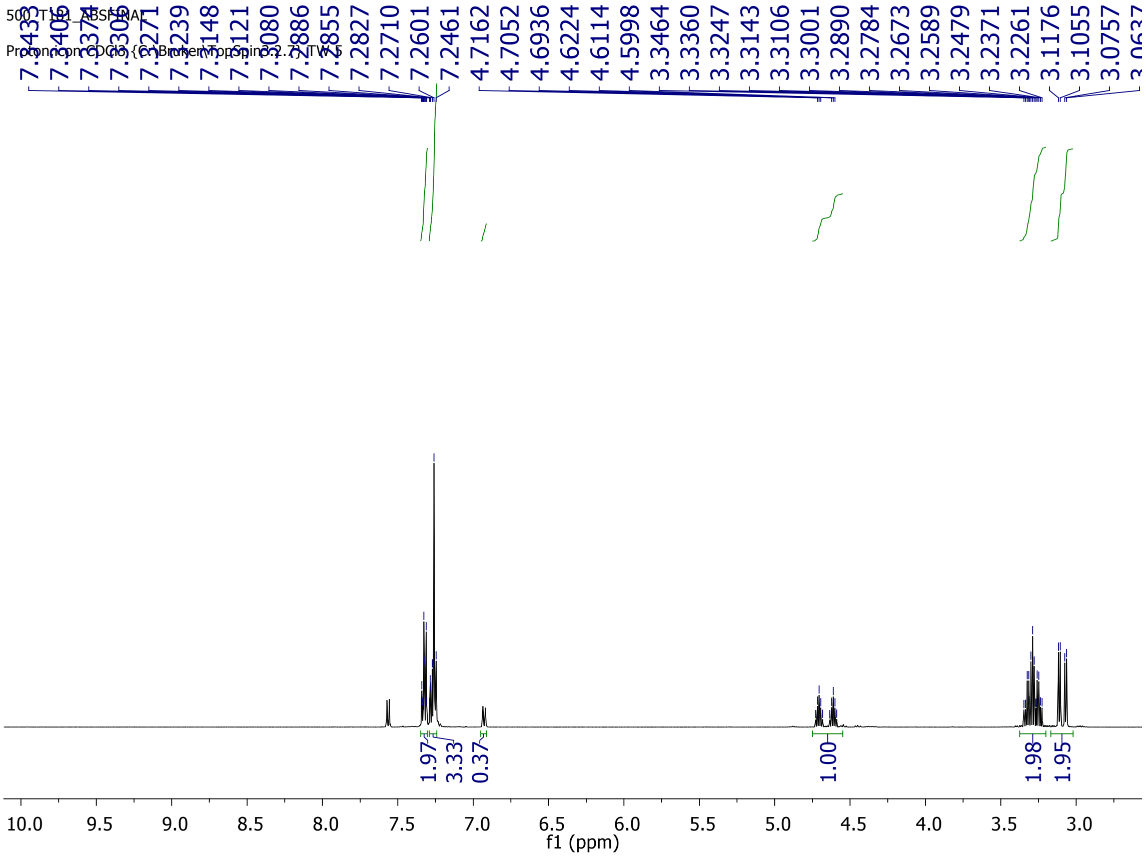

^{19}F NMR of (2-Fluoro-3-iodopropyl)benzene (12ca)

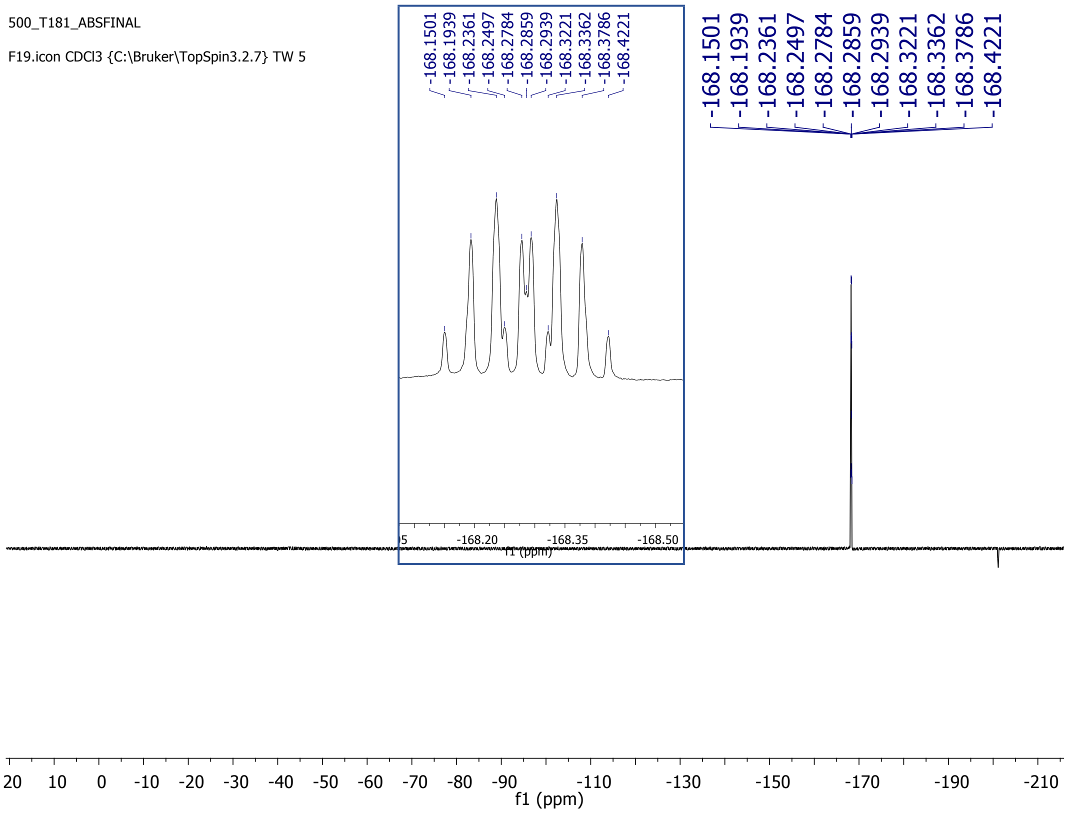

^{13}C NMR of (2-Fluoro-3-iodopropyl)benzene (12ca)

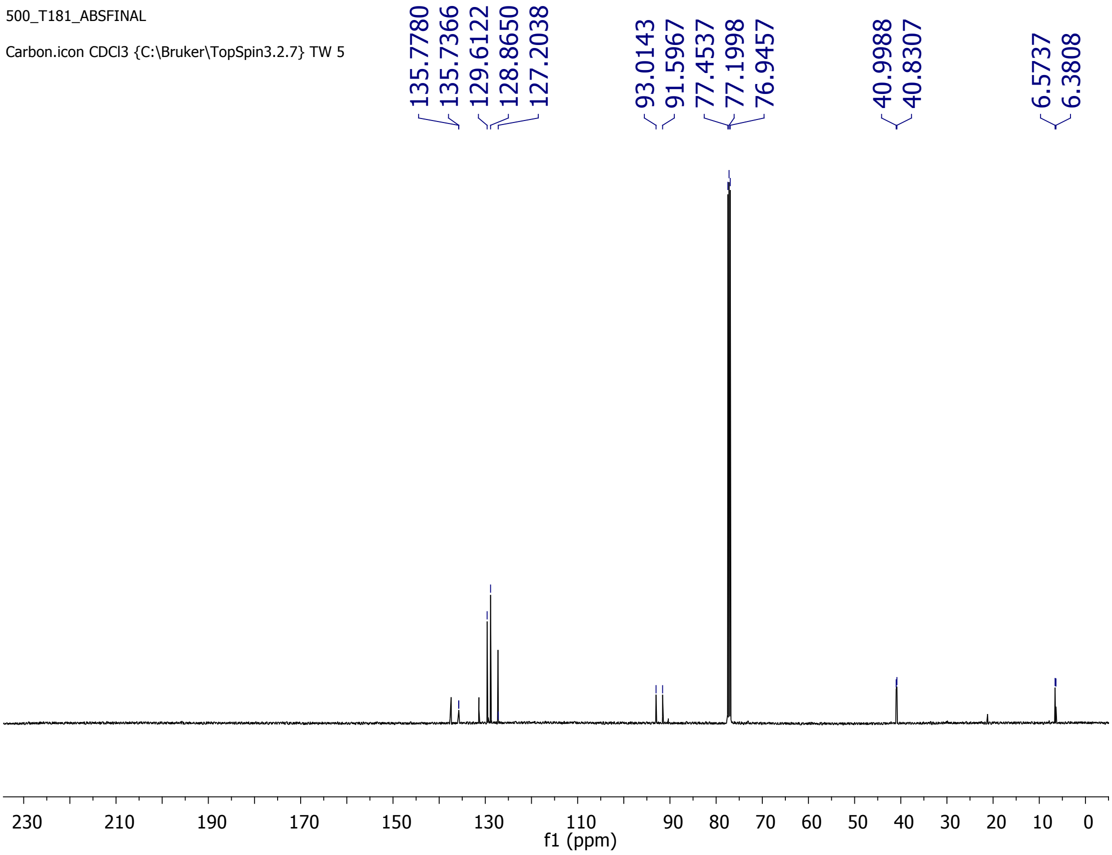

^{1}H NMR of (2,3-Difluoropropyl)benzene (12cb)

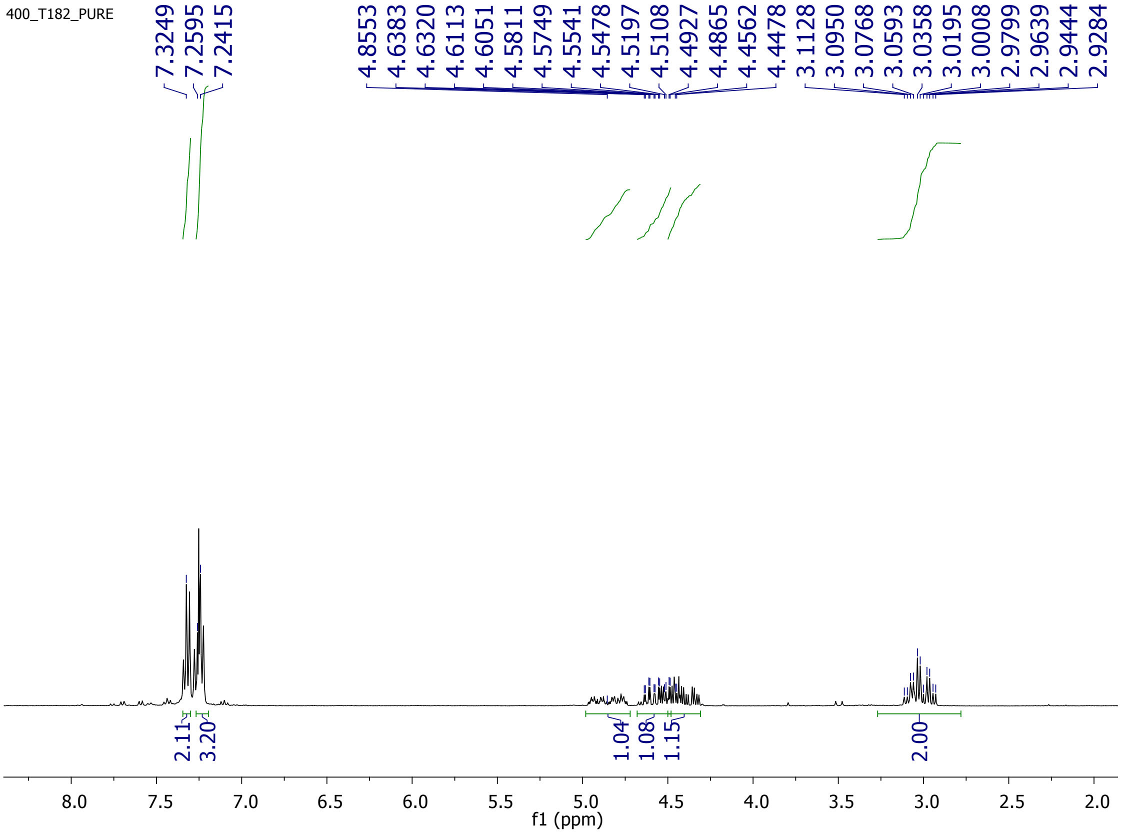

^{19}F NMR of (2,3-Difluoropropyl)benzene (12cb)

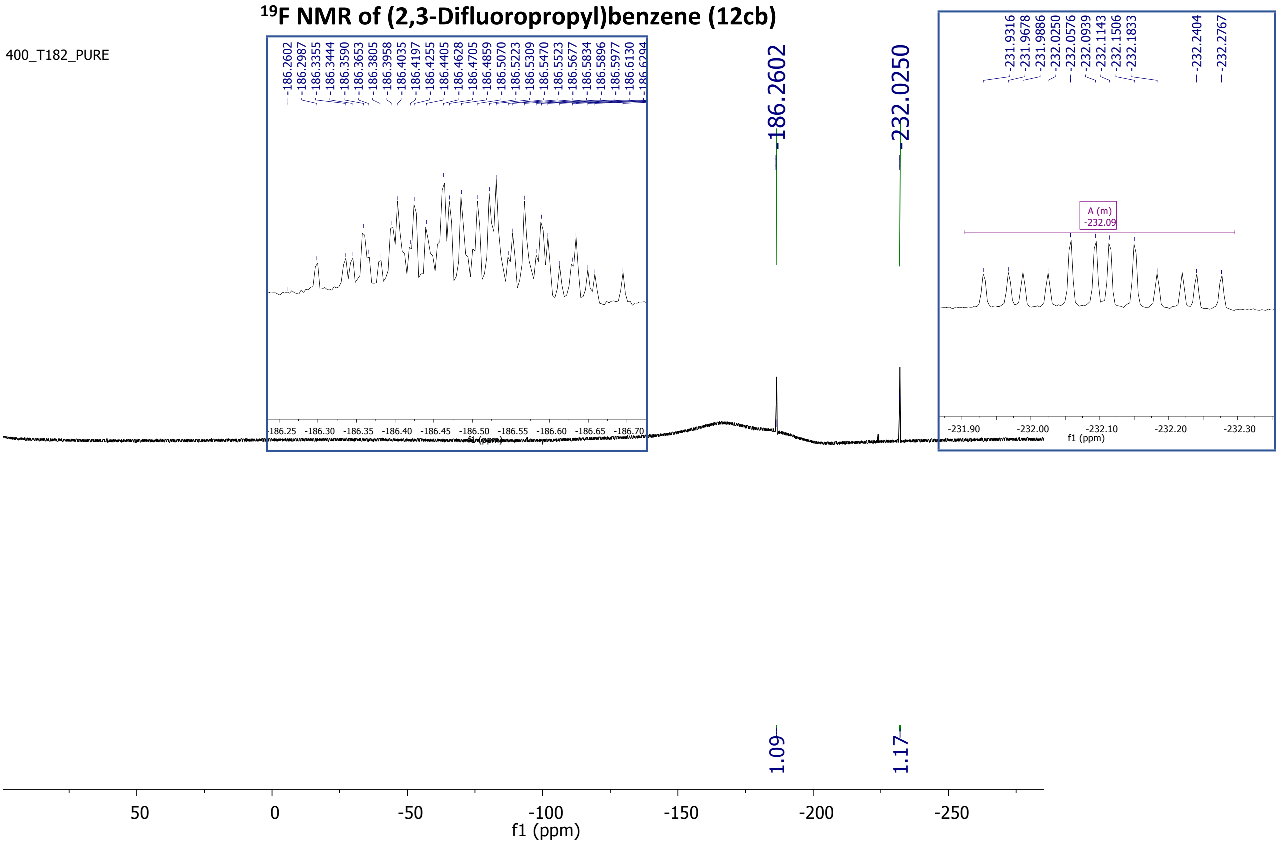

^{19}F NMR of (2R,3R,4S,5S,6R)-2-(Acetoxymethyl)-6-fluoro-5-iodotetrahydro-2H-pyran-3,4-diyl diacetate (12d)

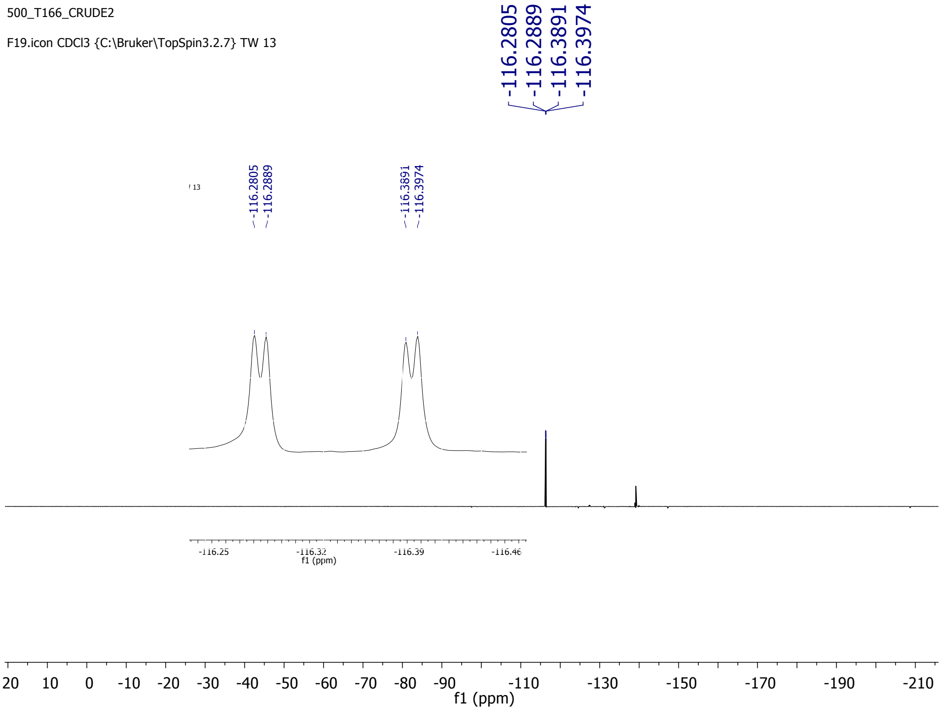

^{1}9F NMR of (2R,3R,4S,5R,6R)-2-(acetoxymethyl)-6-fluorotetrahydro-2H-pyran-3,4,5-triyl triacetate (13a)

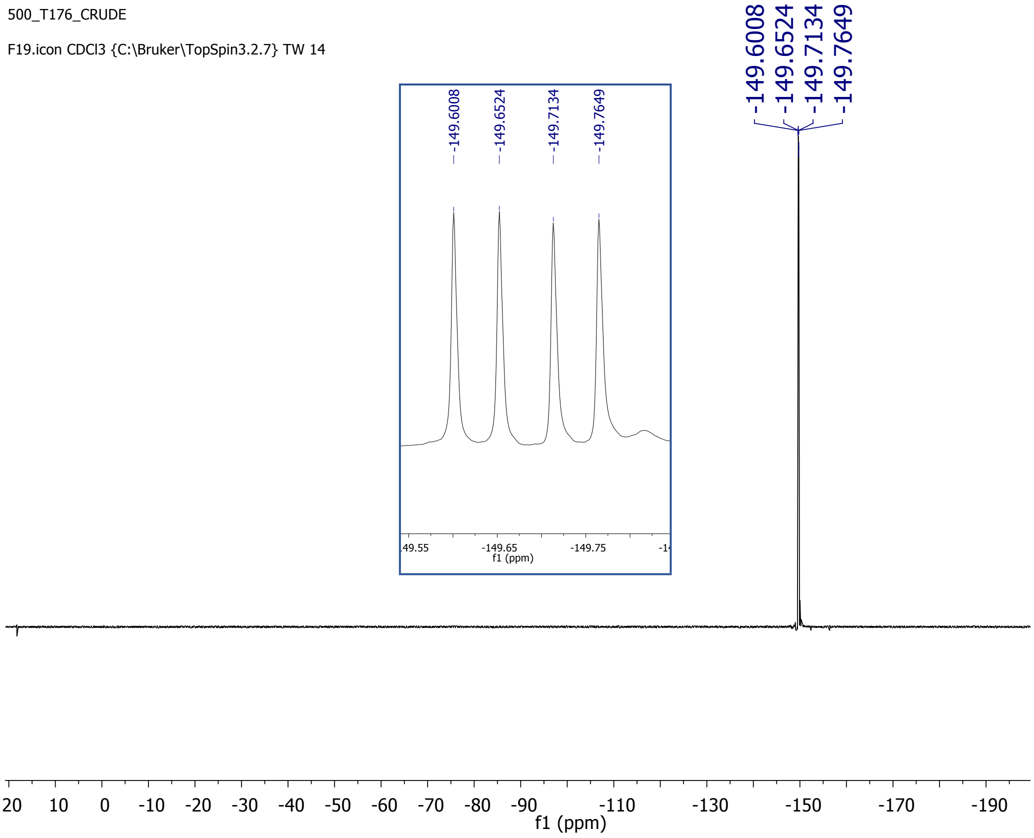

^{1}H NMR of 2-(4,5-Difluoropentyl)isoindoline-1,3-dione (14a)

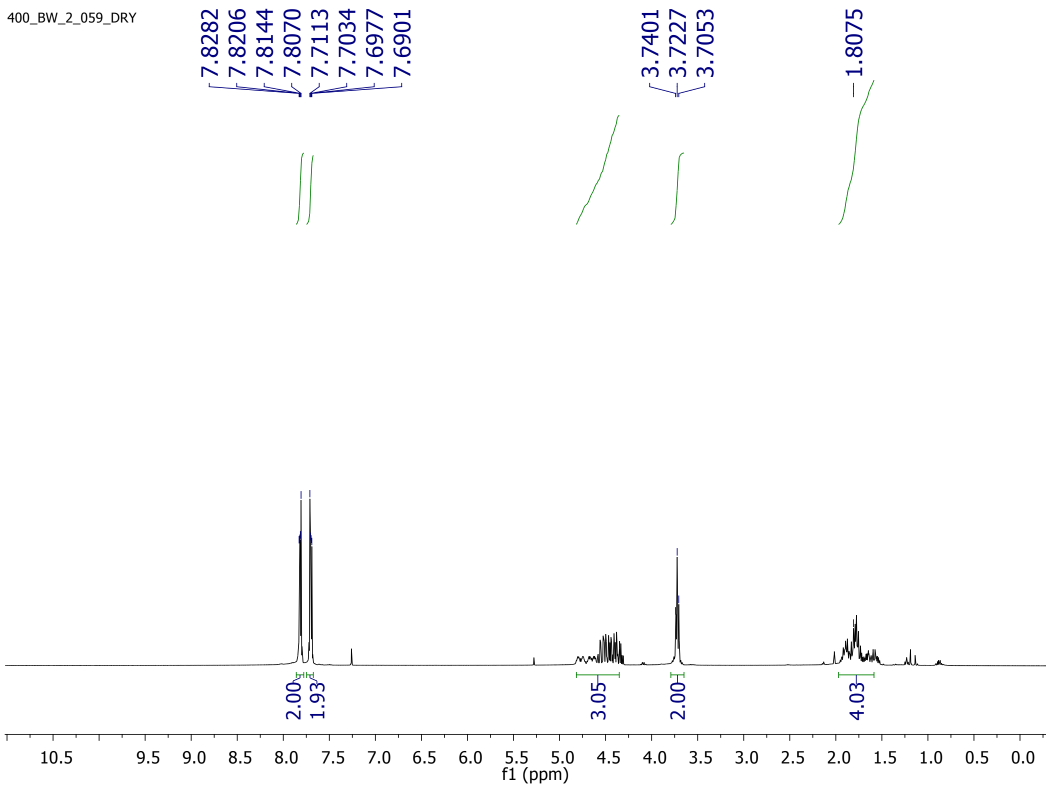

^{19}F NMR of 2-(4,5-Difluoropentyl)isoindoline-1,3-dione (14a)

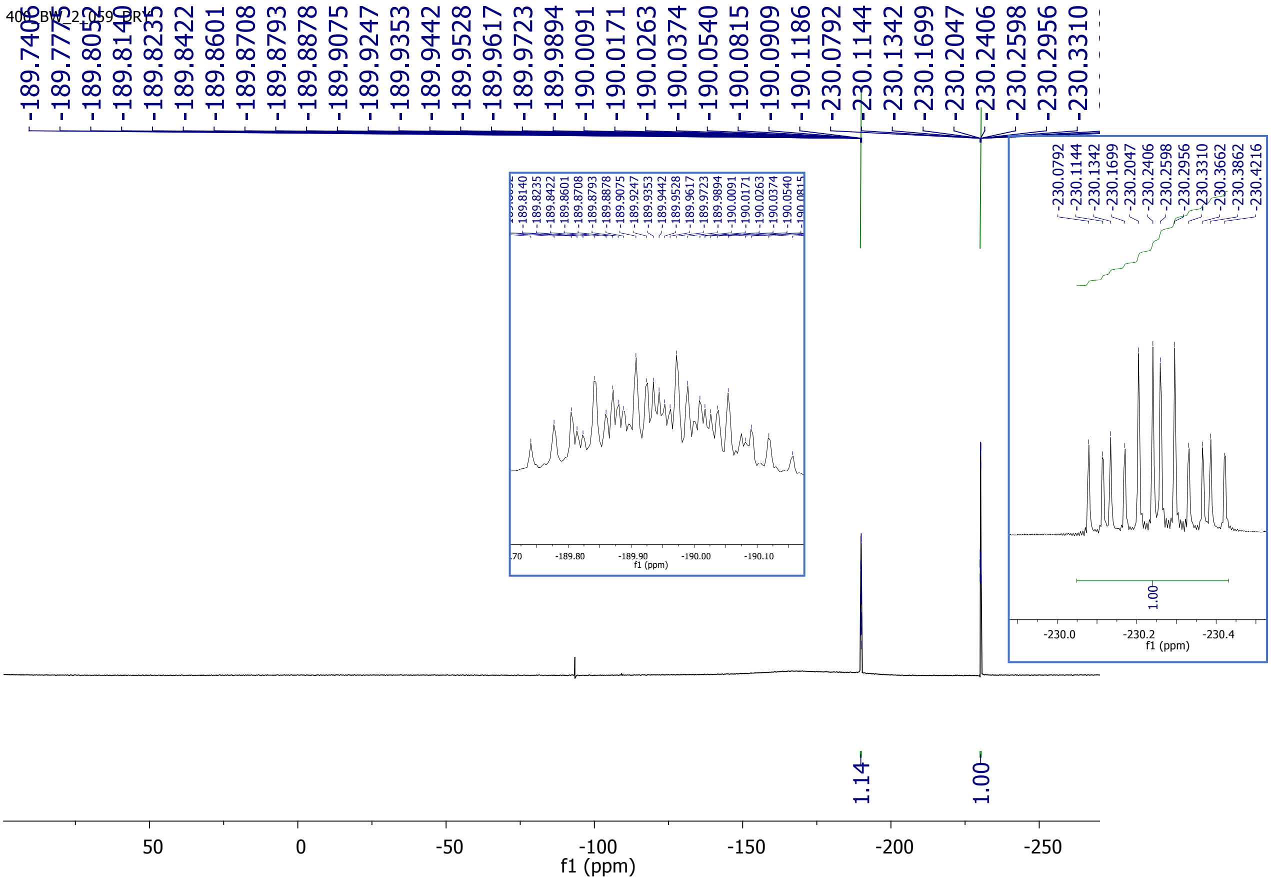

^{13}C NMR of 2-(4,5-Difluoropentyl)isoindoline-1,3-dione (14a)

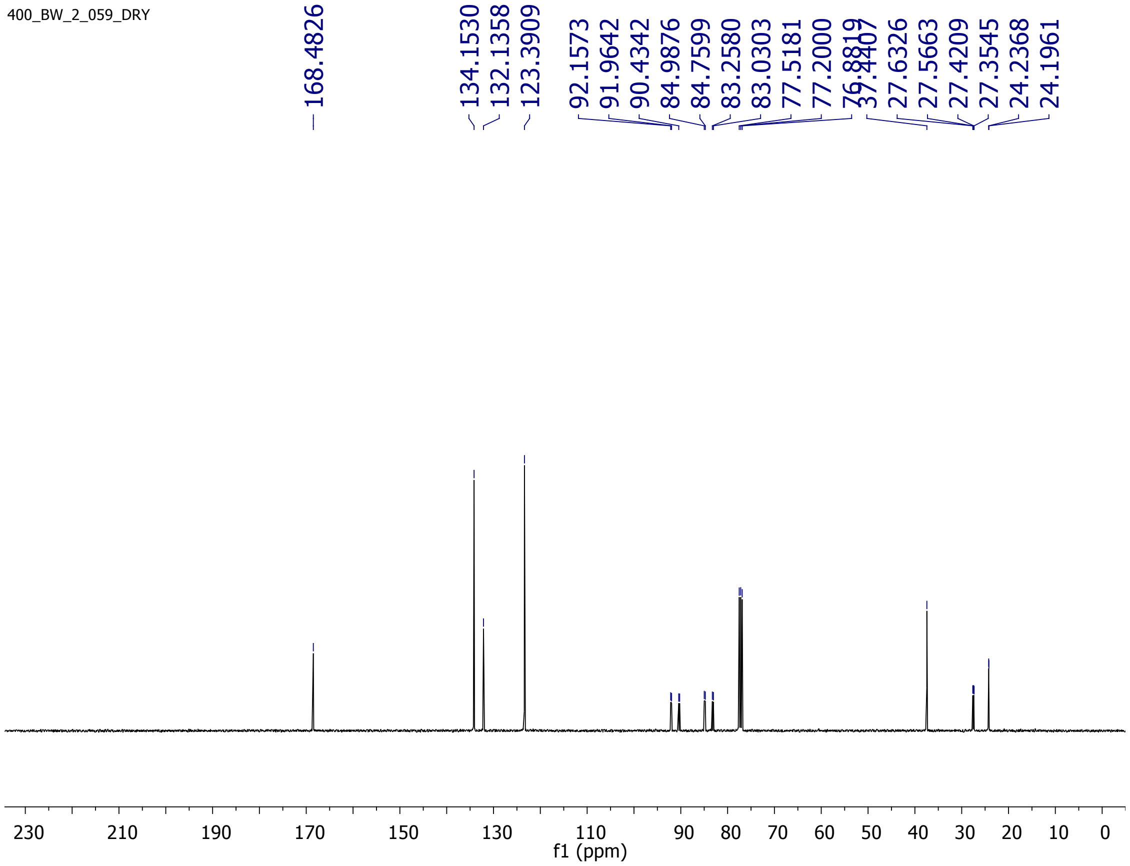

HRMS of 2-(4,5-Difluoropentyl)isoindoline-1,3-dione (14a)

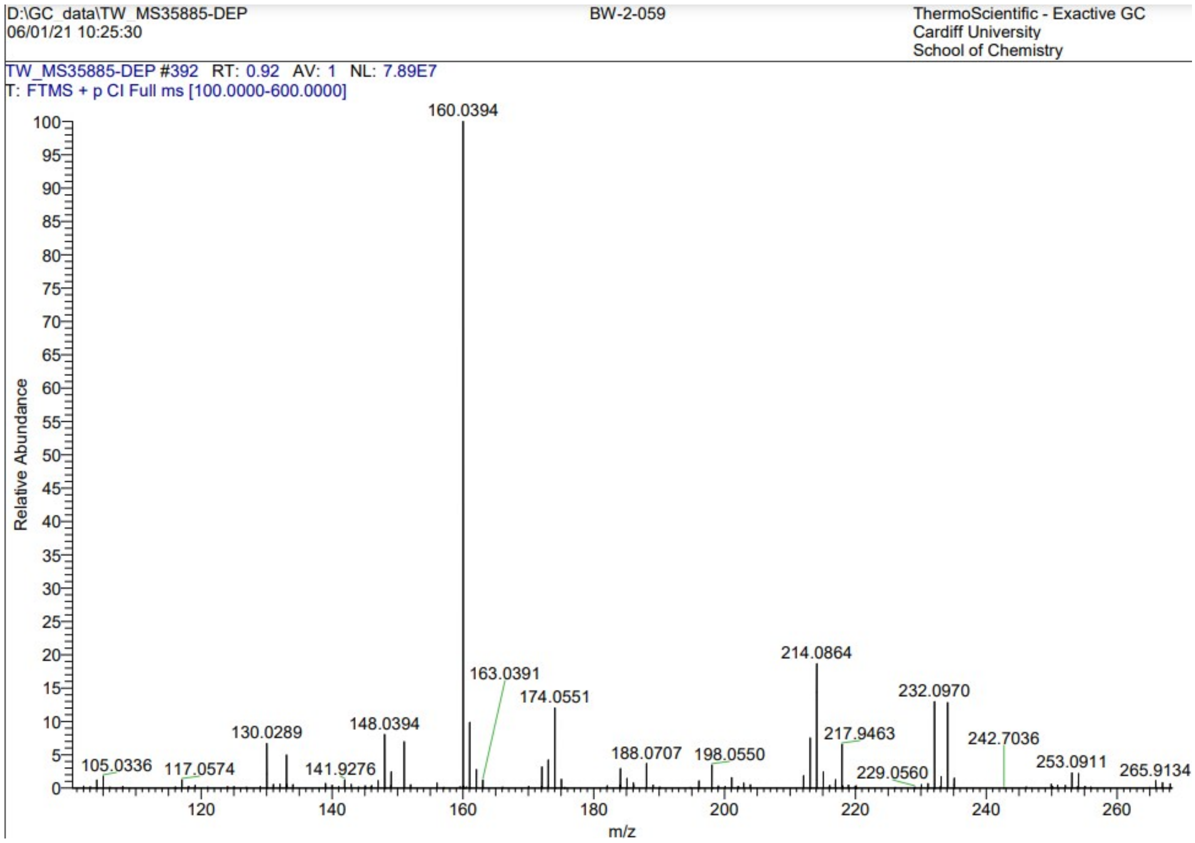

IR of 2-(4,5-Difluoropentyl)isoindoline-1,3-dione (14a)

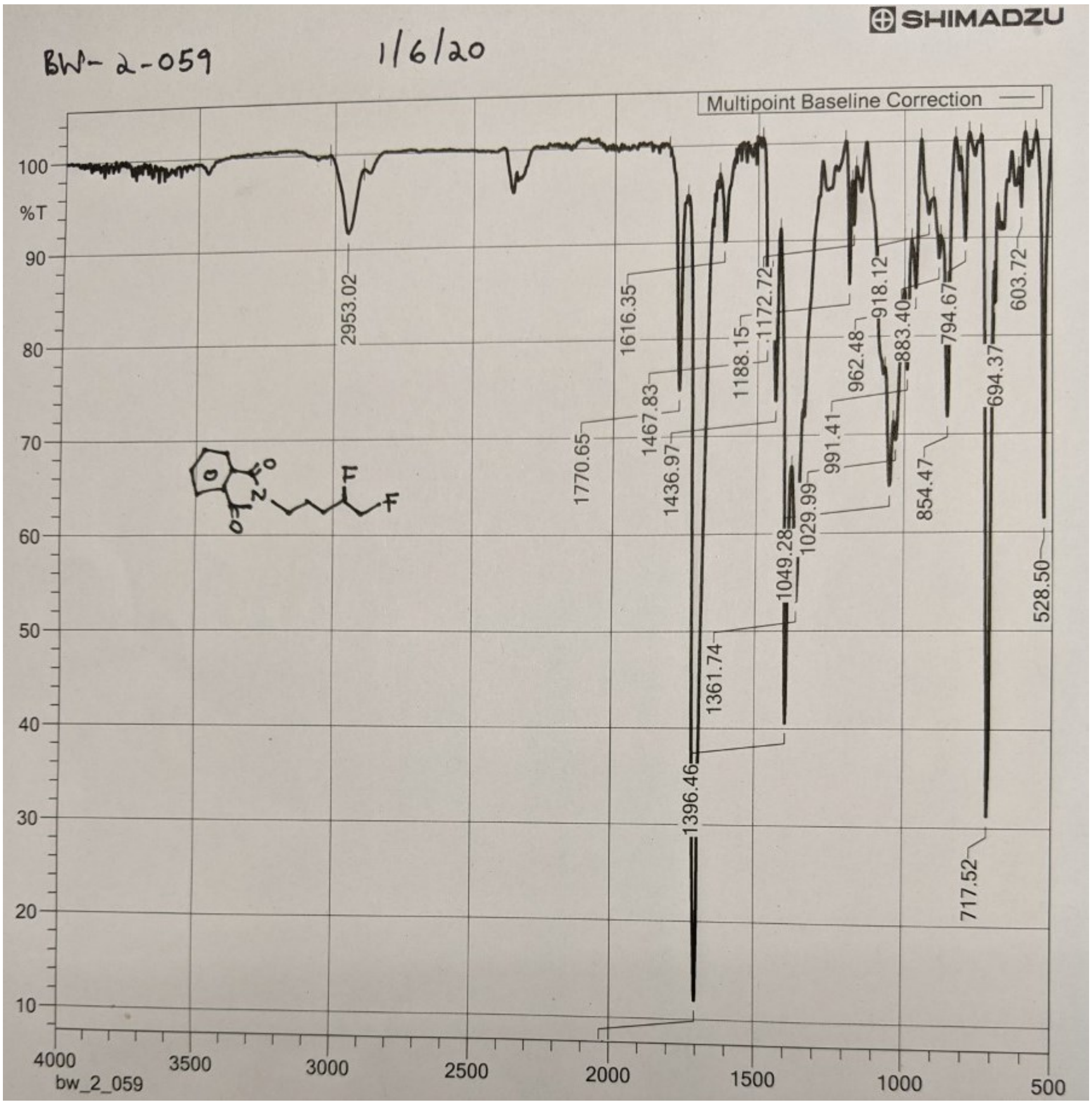

Supplement: SC-012-D1SC02123K-s001 [file SC-012-D1SC02123K-s001.pdf]
